# Bridged bicyclic peptides as potential drug scaffolds: synthesis, structure, protein binding and stability†
†Electronic supplementary information (ESI) available: Crystallographic data; spectroscopic data with ^1^H NMR spectra, HPLC profiles and MS spectra. CCDC 1063793 and 1063796–1063799. For ESI and crystallographic data in CIF or other electronic format see DOI: 10.1039/c5sc01699a


**DOI:** 10.1039/c5sc01699a

**Published:** 2015-07-13

**Authors:** Marco Bartoloni, Xian Jin, Maria José Marcaida, João Banha, Ivan Dibonaventura, Swathi Bongoni, Kathrin Bartho, Olivia Gräbner, Michael Sefkow, Tamis Darbre, Jean-Louis Reymond

**Affiliations:** a Department of Chemistry and Biochemistry , University of Berne , Freiestrasse 3 , 3012 Berne , Switzerland . Email: jean-louis.reymond@dcb.unibe.ch ; Fax: +41 31 631 80 57; b School of Life Sciences , Ecole Polytechnique de Lausanne , 1015 Lausanne , Switzerland; c caprotec bioanalytics GmbH , Berlin , Germany

## Abstract

Double cyclization of short linear peptides obtained by solid phase peptide synthesis was used to prepare bridged bicyclic peptides (BBPs) corresponding to the topology of bridged bicyclic alkanes such as norbornane. Diastereomeric norbornapeptides were investigated by ^1^H-NMR, X-ray crystallography and CD spectroscopy and found to represent rigid globular scaffolds stabilized by intramolecular backbone hydrogen bonds with scaffold geometries determined by the chirality of amino acid residues and sharing structural features of β-turns and α-helices. Proteome profiling by capture compound mass spectrometry (CCMS) led to the discovery of the norbornapeptide **27c** binding selectively to calmodulin as an example of a BBP protein binder. This and other BBPs showed high stability towards proteolytic degradation in serum.

## Abstract 2

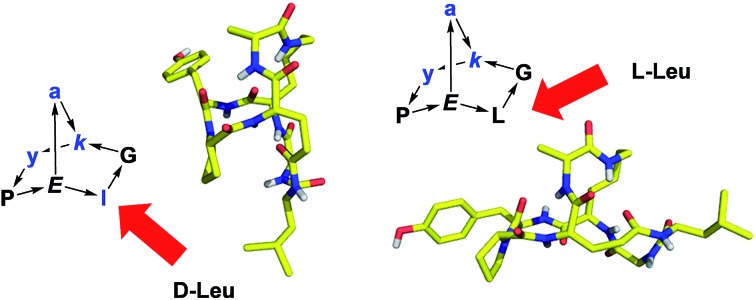
Diastereomeric norbornapeptides represent globular scaffolds with geometries determined by the chirality of amino acid residues and sharing structural features of β-turns and α-helices.

## Introduction

The recurring problem of high attrition rates in drug development has led to questioning whether the types of molecules investigated as drugs should be reconsidered from first principles.^1^ Among various approaches to address this problem, there has been a renewed interest in peptide-based drugs.^2^ Peptides are easily prepared by solid-phase peptide synthesis (SPPS),^3^ cover an intermediate size range otherwise difficult to access, and are suitable for targets with large binding sites such as protein–protein interaction interfaces.^4^ Linear peptides however have poor drug properties since they are rapidly degraded in serum,^5^ and show limited membrane penetration ability^6^ and often weak and unselective target binding due to conformational flexibility.^7^ These limitations can be overcome by rigidifying the structure by cyclization,^8^ which decreases the entropy penalty of binding, increases target selectivity, and may reduce or entirely suppress serum proteolysis.^9^ Cyclization may involve peptide bond formation to form a so-called homodetic cyclic peptide or macrolactonization to form depsipeptides,^10^ as well as disulfide or thioether bridge formation as in cyclotides^11^ and lantibiotics,^12^ and other types of connections between amino acid side-chains.^13^ Further modifications contributing to improved properties include the use of non-natural and d-amino acids^14^ and peptide bond *N*-methylation,^15^ all of which are found in various peptide natural products mostly of bacterial origin.

**Scheme 1:**
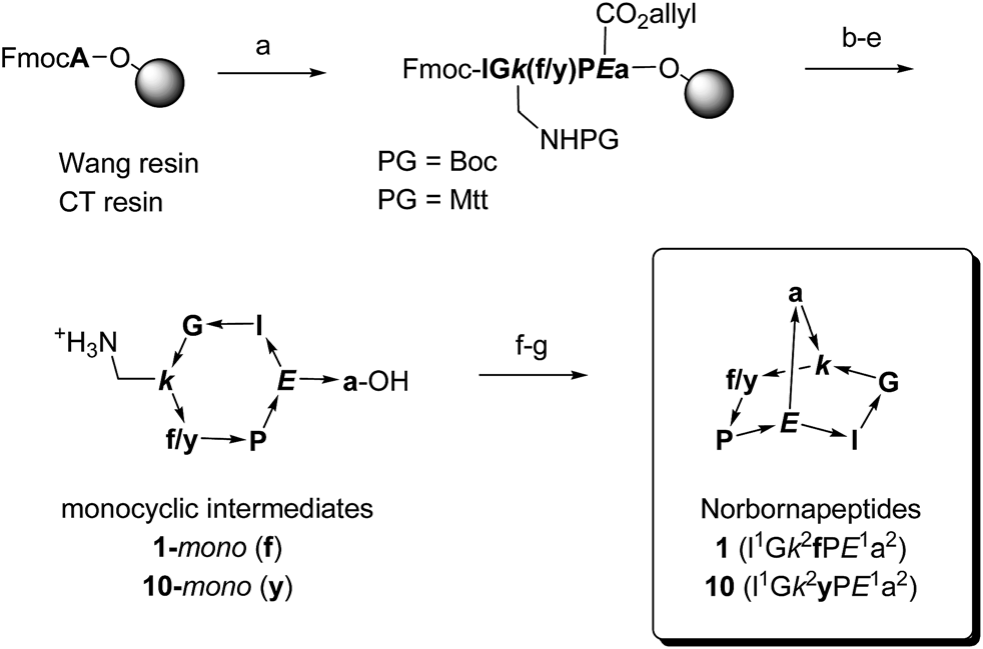
Synthetic schemes for bridged bicyclic peptides without protected side chains (**1–20**) or with Boc protected side chains (**21–26**), exemplified with norbornapeptides **1** and **10**. One letter codes are used for amino acids, upper case letters = l-amino acids, lower case letters = d-amino acids, italics = branching amino acids, arrow bonds indicate peptide bonds in C → N direction. Conditions: (a) Fmoc SPPS; (b) Pd(PPh_3_)_4_, PhSiH_3_, anh. CH_2_Cl_2_; (c) piperidine–DMF 1 : 4; (d) HATU, iPr_2_EtN, NMP–DMSO 4 : 1; (e) Wang resin–PG = Boc : CF_3_CO_2_H–iPr_3_SiH–H_2_O 94 : 5 : 1, chlorotrityl resin–PG = Mtt: 1% CF_3_CO_2_H in CH_2_Cl_2_; (f) HATU, iPr_2_EtN, CH_2_Cl_2_–DMF 20 : 1; (g) only for chlorotrityl resin–PG = Mtt route: CF_3_CO_2_H–CH_2_Cl_2_ 4 : 1.

Recently we proposed a strategy to expand the chemical space of bioactive peptides based on mathematical graphs.^16^ In analogy to small molecule graphs where graph nodes correspond to atoms and graph edges to covalent bonds,^17^ peptide graphs result from assigning graph nodes to amino acids and graph edges to peptide bonds. While only linear and cyclic peptides are obtained when using only monovalent (terminal) and divalent (intra-chain) nodes, introducing branching points in the peptide chains in form of diamino acids (*e.g.* lysine) or amino diacids (*e.g.* glutamic acid) leads to a diversity of possible polycyclic peptide topologies as potential new types of constrained peptides, in particular bridged bicyclic peptides (BBPs). BBPs represent a vast molecular class comprising up to 3.1 x 10^19^ possible members derived from 97 bicyclic graphs with up to 15 residues taken from 20 proteinogenic amino acids. Expanding from only a handful of examples in the literature prepared by solution phase synthesis,^18^ we recently showed that BBPs can be obtained by solid-phase peptide synthesis (SPPS) and on-resin cyclization to a monocyclic peptide, followed by a second cyclization in solution coupling the C-terminal carboxyl group with the ε-amino group of a lysine side-chain to form the final product, a strategy which is also followed for the present paper (Scheme 1).^19^ The bridgehead chirality was chosen such as to present the amino- and carboxyl-group on the same stereotopic face of the monocyclic peptide, which favoured the second cyclization. The X-ray crystal structure of a bicyclo[3.3.2]decapeptide established that BBPs cyclize to form a concave bicyclic system^20^ with the bridgehead amino acid H–C(α) proton pointing outwards.^21^

Herein we investigate the properties of BBPs as drug scaffolds. In drug design scaffolds are core structural elements, mostly polycyclic systems (*e.g.* benzofurans, steroids, benzodiazepines, *etc.*), that appear decorated with various substituents in drug molecules.^22^ Ideally, the scaffold chemistry is chosen such that substituents can be varied easily using a conserved synthetic approach.^23^ In the area of peptides, typical scaffold approaches include various α-helix mimetics such as polyphenyls on which residue side-chains can be grafted,^24^ cyclic peptides,^25^ and β-hairpin mimetics which can be incorporated into existing peptide sequences.^26^

We focus on diastereomers and side-chain analogs of norbornapeptide **1** which represents an expanded form of the parent hydrocarbon scaffold norbornane (Scheme 1). Structural studies by X-ray crystallography, ^1^H-NMR and CD spectroscopy show that these BBPs mostly exist as single conformers adopting a rigid structure stabilized by several intramolecular backbone hydrogen bonds. The relative orientation of side chains is determined by the chirality of the various residues as well as by interactions between side-chains within the constrained BBP geometry. A proteome profiling experiment by capture compound mass spectrometry (CCMS)^27^ using photoreactively labeled norbornapeptides is reported leading to the discovery of **27c** binding selectively to calmodulin as an example of a BBP protein binder. In contrast to linear peptides this and other BBPs are shown to be stable towards serum degradation.

## Results and discussion

### Synthesis of diastereomeric norbornapeptides

Diastereoisomers and side-chain analogs of norbornapeptide **1** were prepared by Fmoc-SPPS using either the original protocol on Wang resin, or a modified approach using a 2-chlorotrityl resin as solid support and a 4-methyltrityl (Mtt) protected lysine as the branching diamino acid. In both cases the first cyclization was carried out on the resin between the N-terminus and the side-chain carboxyl group of a glutamate residue released from its allyl ester by Palladium catalyzed deprotection. The second method on 2-chlorotrityl resin enabled the subsequent cleavage of the C-terminal carboxyl group from the resin and lysine side chain deprotection under weakly acidic conditions, and was suitable to carry out the second cyclisation in solution with sequences containing side-chain protected residues (Scheme 1). These procedures were used to prepare the diastereoisomeric series **1–18** and side-chain analogs **19–26** (Table 1).

**Table 1:** Synthesis of norbornapeptides (Scheme 1)

No.	Sequence^*a*^	mg	Yield^*b*^ (%)	[M + H]^+^ calc./obs.^*c*^
**1**	**l** ^1^ **G*k*** ^2^ **fP*E*** ^1^ **a** ^2^	34.9	82	725.40/725.40
**2**	l^1^G*k*^2^fP*E*^1^**A**^2^	25.9	60	725.40/725.40
**3**	l^1^G*k*^2^**F**P*E*^1^a^2^	11.4	56	725.40/725.40
**4**	**L** ^1^G*k*^2^fP*E*^1^a^2^	35.2	56	725.40/725.40
**5**	l^1^G*k*^2^**F**P*E*^1^**A**^2^	8.6	59	725.40/725.40
**6**	**L** ^1^G*k*^2^fP*E*^1^**A**^2^	22.4	61	725.40/725.40
**7**	**L** ^1^G*k*^2^**F**P*E*^1^a^2^	17.5	36	725.40/725.40
**8**	**L** ^1^G*k*^2^**F**P*E*^1^**A**^2^	34.3	81	725.40/725.40
**9**	**L** ^1^G***K***^2^**F**P*E*^1^**A**^2^	6.6	27	725.40/725.40
**10**	l^1^G*k*^2^**y**P*E*^1^a^2^	8.9	4*	741.39/741.39
**11**	l^1^G*k*^2^**y**P*E*^1^**A**^2^	10.9	59	741.39/741.42
**12**	l^1^G*k*^2^**Y**P*E*^1^a^2^	4.7	37	741.39/741.42
**13**	**L** ^1^G*k*^2^**y**P*E*^1^a^2^	21.8	73	741.39/741.40
**14**	l^1^G*k*^2^**Y**P*E*^1^**A**^2^	7.0	49	741.39/741.43
**15**	**L** ^1^G*k*^2^**y**P*E*^1^**A**^2^	20.5	42	741.39/741.39
**16**	**L** ^1^G*k*^2^**Y**P*E*^1^a^2^	18.1	72	741.39/741.41
**17**	**L** ^1^G*k*^2^**Y**P*E*^1^**A**^2^	7.3	15	741.39/741.45
**18**	**L** ^1^G***K***^2^**Y**P*E*^1^**A**^2^	13.8	37	741.39/741.40
**19**	**k** ^1^G*k*^2^fP*E*^1^a^2^	7.6	3*	740.41/740.41
**20**	l^1^**S***k*^2^fP*E*^1^a^2^	23.4	10*	755.41/755.41
**21**	l^1^G*k*^2^f**K***E*^1^a^2^	7.7	3*	756.44/756.44
**22**	**k** ^1^G*k*^2^**e**P*E*^1^**c**^2^	7.4	3*	754.36/754.36
**23**	**K** ^1^G*k*^2^fP*E*^1^**A**^2^	15.0	15*	740.41/740.41
**24**	**L** ^1^ **S** *k* ^2^fP*E*^1^**A**^2^	12.2	6*	755.41/755.41
**25**	**L** ^1^G*k*^2^f**K***E*^1^**A**^2^	12.0	5*	756.44/756.44
**26**	**K** ^1^G*k*^2^**e**P*E*^1^**C**^2^	5.3	3*	754.36/754.36

^*a*^One-letter code for amino acids, upper case = l-, lower case = d-amino acids, sequences are given as prepared by SPPS written from N- to C-terminus, superscript numbers indicate residues involved in amide bond ring closures in 1^st^ (^1^) and 2^nd^ (^2^) cyclization.

^*b*^Yields of HPLC purified products are given for the second cyclization reaction; asterisks mark total yields (SPPS and second cyclization). In all cases, yields are calculated for the corresponding trifluoroacetate salts.

^*c*^ESI-MS spectra (positive mode) were recorded on a LTQ OrbitrapXL hybrid ion trap-Orbitrap mass spectrometer.

### Structural studies

BBPs **1–18** were subjected to crystallisation screening either under protein crystallisation conditions (aqueous buffers with additives) or under small molecule crystallisation conditions (organic solvents). The protein crystallisation screen, which had succeeded earlier in the case of a bicyclo[3.3.2]decapeptide,21*b* did not yield crystals in the present study. On the other hand the tyrosine-containing norbornapeptide **10** yielded diffracting crystals in a water–trifluoroethanol–DMSO (7 : 2 : 1) mixture, and its stereoisomers **12**, **13** and **15** provided suitable crystals from methanol. Although solvent molecules and H-atoms were not always entirely resolved the structures were of sufficient quality to unambiguously describe the BBP structures. In all four cases, crystallisation was favoured by the formation of an intermolecular H-bond between the tyrosine side-chain hydroxyl groups (Tables S1–S20†).

**Fig. 1:**
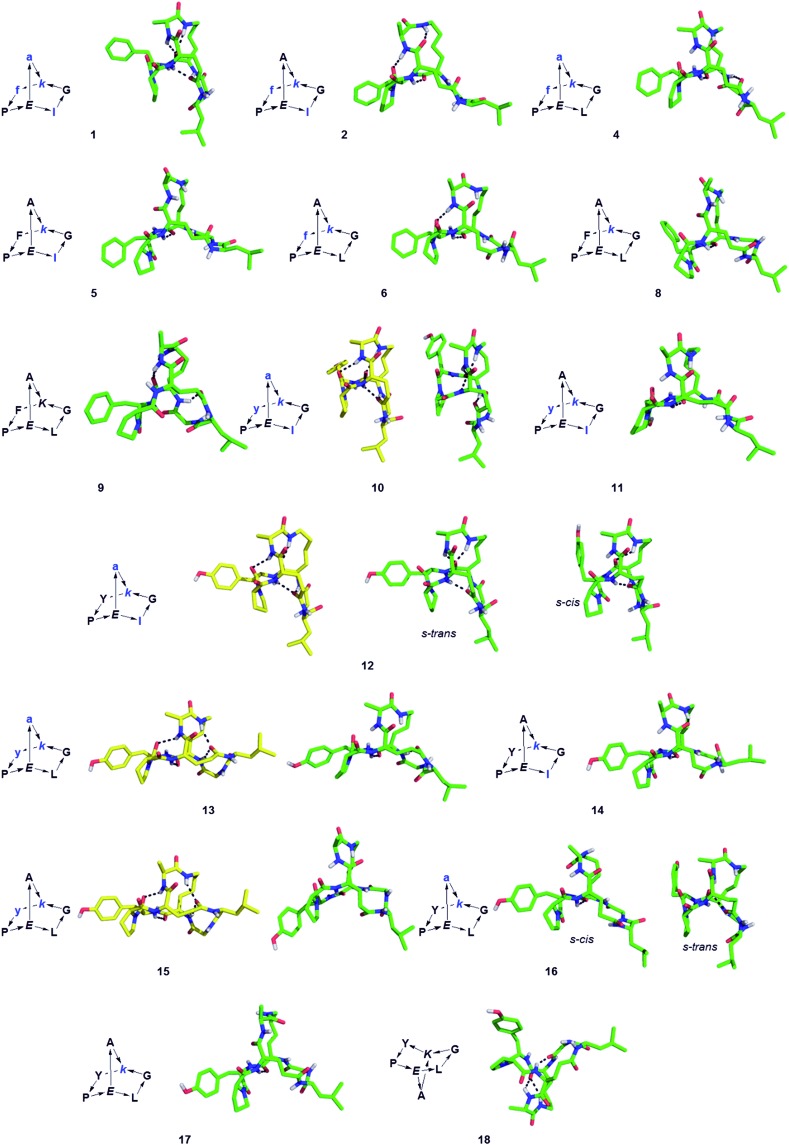
Structures of norbornapeptides determined by X-ray crystallography (yellow) or NMR (green). Intramolecular hydrogen bonds are marked as black dashed lines. BBP **12** crystallized solely in the s-*trans* conformation of the Tyr4-Pro5 peptide bond, while both conformations occur in solution for **12** and **16**.

The structure of BBPs **1–18** was furthermore established by NMR spectroscopy. Proton resonances in ^1^H NMR spectra were assigned from standard 1D and 2D experiments. The BBPs gave single sets of signals with the exception of **3**, **7**, **12** and **16** where two conformers appeared as distinct sets of signals which coalesced to a single set around 90 °C. Proton–proton distance constraints were extracted from 2D ROESY NMR spectra and used in a restrained molecular dynamics simulated annealing (rMDSA) using ff12SB force field from AMBER12 package^28^ for 1 ns starting from a 3D-model derived from the experimental X-ray structure of the BBP or its closest diastereoisomer. With the output structure from rMDSA, another 1 ns non-restrained simulation was implemented in explicit solvent. The averaged structure over the last 10 frames of the MD simulations was used as the final structure, which was checked for consistency by the presence of intramolecular hydrogen bonds identified as temperature independent amide protons in the NMR spectra of the phenylalanine series (**1–9**) mostly engaging the bridgehead lysine α-NH and bridgehead glutamate α-NH (Table 2). In the case of **5**, **8**, **9**, **14** and **18** the structure obtained after minimization starting from the closest X-ray structure was not consistent, however a satisfactory NMR structure was obtained starting the minimization from a model built using Maestro (version 8.5).^29^ For BBP **3** and **7** existing as two equilibrating phenylalanyl-proline s-*cis*/s-*trans* conformers the NMR signal could not be assigned well enough for a structure determination, however their tyrosine analogs **12** and **16** gave satisfactory structures for both conformers. For BBPs **9** and **18** with mismatched bridgehead chirality (both bridgehead residues as l-enantiomers) the NMR structures could be resolved with both possible bridge orientations. BBP **9** is shown with a “bridge-up” stereochemistry and BBP **18** with a “bridge-down” orientation corresponding to the solution with best fit to distance constraints. In total 22 different structures were obtained, including two pairs of equilibrating conformers (Fig. 1).

**Table 2:** Amide proton temperature coefficients indicating intramolecularly H-bonded protons in diastereomeric norbornapeptides **1–9**^*a*^

	**1**	**2**	**3** ^*b*^	**4**	**5**	**6**	**7** ^b^	**8**	**9**
l/d-Leu^1^-H_N_	7.3	8.1	**3.7**	7.7	7.8	8.5	6.8	7.8	7.2
Gly^2^-H_N_	7.9	7.7	4.4	6.8	7.0	7.9	6.2	7.6	7.9
l/d-Lys^3^-H_N_	8.8	**3.1**	**2.4**	**2.1**	**1.3**	**2.8**	**1.4**	**2.0**	**1.6**
l/d-Phe^4^-H_N_	6.6	7.2	5.2	5.3	10.0	8.0	6.3	8.8	9.2
l-Glu^6^-H_N_	**1.7**	**2.3**	**–1.0**	**1.2**	**1.2**	**1.2**	**1.8**	**1.5**	7.2
l/d-Ala^7^-H_N_	**3.0**	7.6	**0.3**	**1.4**	9.6	6.2	9.9	9.0	8.3
l/d-Lys^3^-H_Nε_	6.6	7.3	*n.d.*	4.3	6.6	6.6	*n.d.*	6.5	**2.0**

^*a*^Coefficients in –Δ*δ*/Δ*T* (ppb K^–1^). Fully H-bonded protons (boldface) have –Δ*δ*/Δ*T* < 4.0 ppb K^–1^ and partially H-bonded protons have –Δ*δ*/Δ*T* = 4.0–7.0 ppb K^–1^. *n.d.* = not determined. Low values indicating intramolecular H-bonds are highlighted in bold.

^*b*^Coefficient values given for the major conformer of the prolyl bond, *trans* for **3** and *cis* for **7**. Residue number according to the position in the parent linear precursor (Table 1).

**Fig. 2:**
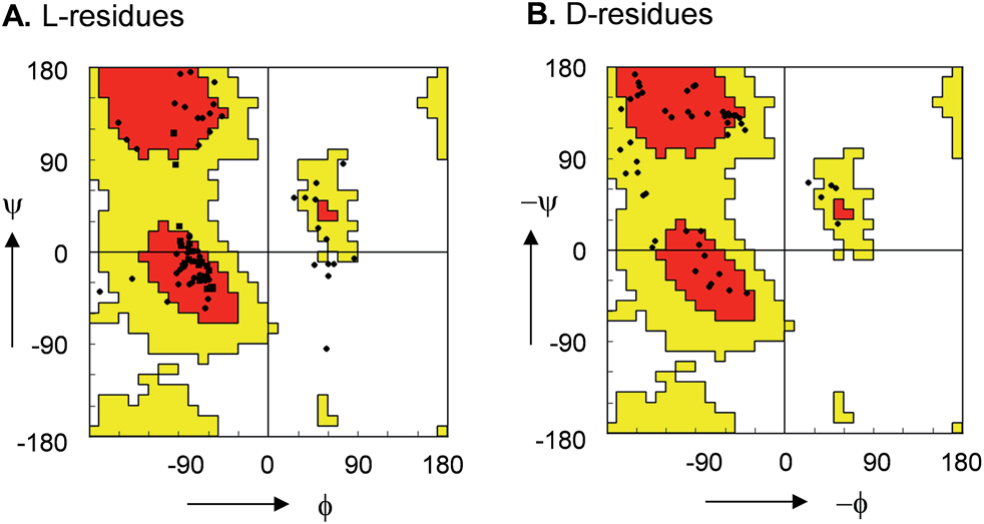
Ramachandran plot of peptide bond torsional angles observed in norbornapeptide structures for (A) l-residues and (B) d-residues. Preferred (red) and allowed (yellow) regions as observed in protein structures are highlighted. The (*φ*, *ψ*) torsional angles in the unallowed bottom right portion in (A) are at the α-carbon of l-leucine in **6**.

For **10**, **12**, **13** and **15** the structures determined by NMR were almost superimposable with their X-ray structure, with slight variations in the orientation of the peptide backbone probably reflecting a small degree of conformational flexibility. In all 22 structures determined (4 X-ray and 18 NMR structures) the peptide bonds occurred in the more stable s-*trans* conformation except for the occurrence of an s*-cis* arrangement in the secondary amide bonds phenylalanyl-proline of **5**, **8**, **9**, s*-cis* tyrosinyl-proline of **14**, **17**, **18**, and an s*-cis*/s*-trans* equilibrium in the tyrosinyl-proline bond of **12** and **16**. Almost all backbone torsional angles resided within allowed regions of the Ramachandran plot, providing further evidence that the bicyclic structure of the norbornapeptide did not induce unusual conformational constraints on the peptide chain despite its relatively constrained nature (Fig. 2).

**Fig. 3:**
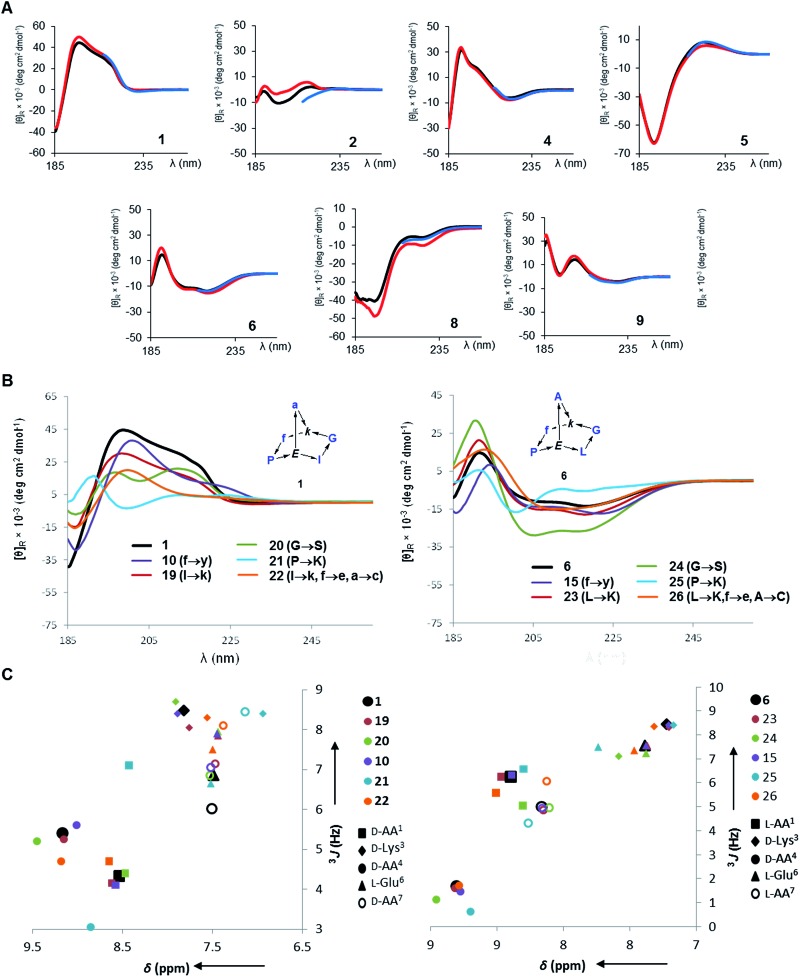
Circular dichroism and ^1^H-NMR of norbornapeptides. (A). CD spectra of **1** and diastereomers **2**, **4–6**, **8**, **9** (250 μg mL^–1^) in aq. 10 mM phosphate buffer pH = 7.0 (black), in 25% trifluoroethanol in aq. 10 mM phosphate buffer pH = 7.0 (red), and in aq. 6 M guanidinium chloride (blue). BBP **3** and **7** existing as equilibrating conformers were not considered. (B). CD spectra of **1**, **6** and their side-chain analogs (350 μg mL^–1^) in 10 mM aq. Phosphate pH 7.0. (C). ^1^H-NMR metrics on amide proton signals of norbornapeptides. (C). Chemical shift and ^3^*J* values for side-chain variations of **1** (left) and **6** (right) and their side-chain analogs **10**, **19–22** (to be compared to **1**) and **15**, **23–26** (to be compared to **6**).

In all determined structures at least two intramolecular backbone hydrogen bonds were observed, in particular involving the (α)NH of the bridgehead lysine and glutamate residues. These intramolecular hydrogen bonds contributed to scaffold rigidity, which was also evidenced by the existence of single conformers in solution with the exception of the proline s-*cis*/s-*trans* equilibria observed with **3**, **7**, **12** and **16** as discussed above. Further evidence for the conformational rigidity of norbornapeptides was provided by circular dichroism (CD) spectra. Each BBP gave a specific circular dichroism (CD) signature independent of the medium (water, 25% aqueous trifluoroethanol as folding inducer, or aq. 6 M guanidinium chloride as denaturant, Fig. 3A). Furthermore CD spectra of BBPs **19–26** having various amino acid side-chains but a conserved backbone and residue stereochemistry also gave comparable CD traces, showing that the backbone conformation was largely independent of the amino acid side-chains in the selected positions (Fig. 3B). The only exception concerned **21** and **25**, where the proline residue was substituted by a lysine, in line with the key structural role played by proline in peptide chains. The effect was confirmed by the similarities in the ^1^H NMR amide proton signal characteristics of these side-chain analogs (Fig. 3C).

**Fig. 4:**
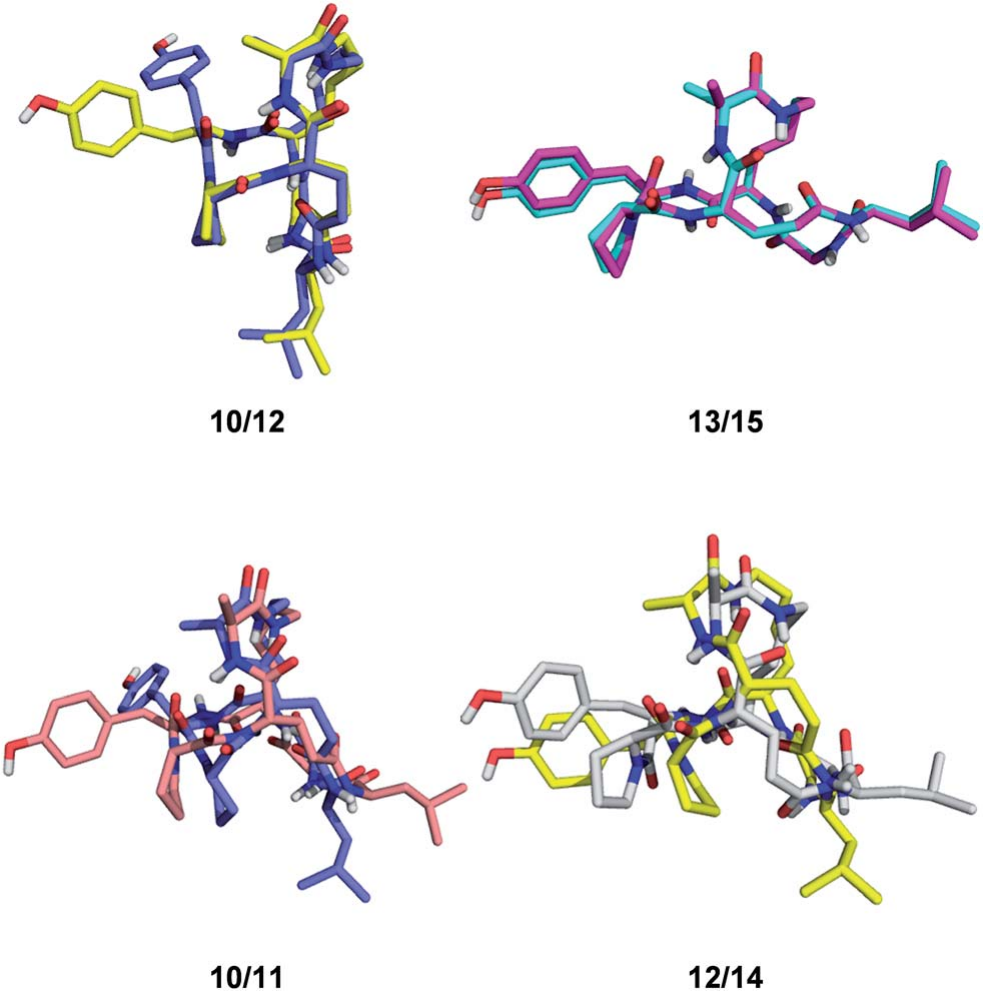
Effect of single residue stereochemical inversion on BBP structure. Pairwise alignment of α-carbons. *Blue*: **10**; *orange*: **11**; *yellow*: **12**; *cyan*: **13**; *grey*: **14**; *magenta*: **15**.

Despite of their rigidity the individual diastereomeric norbornapeptides displayed diverse scaffold geometries reflecting the influence of residue chirality on the overall structure. While for pairs **10**/**12** and **13**/**15** a single stereochemical inversion of phenylalanine respectively alanine only reoriented the side-chain without affecting the scaffold geometry as would occur in the parent norbornane scaffold, inversion of the bridging alanine in the pairs **10**/**11** and **12**/**14** induced a rearrangement of the peptide backbone such that the diastereomeric pairs had overall quite different geometries (Fig. 4). These backbone rearrangements were partly triggered by steric clashes between the bridge alanine and the tyrosine respectively leucine side chain, an effect resulting from the bridged nature of the BBP creating a 3D shape bringing side-chains close to one another, a situation reminiscent of folded proteins, but which is not observed in small monocyclic peptides which mostly adopt a planar shape.^30^

### Secondary structures of BBP

**Fig. 5:**
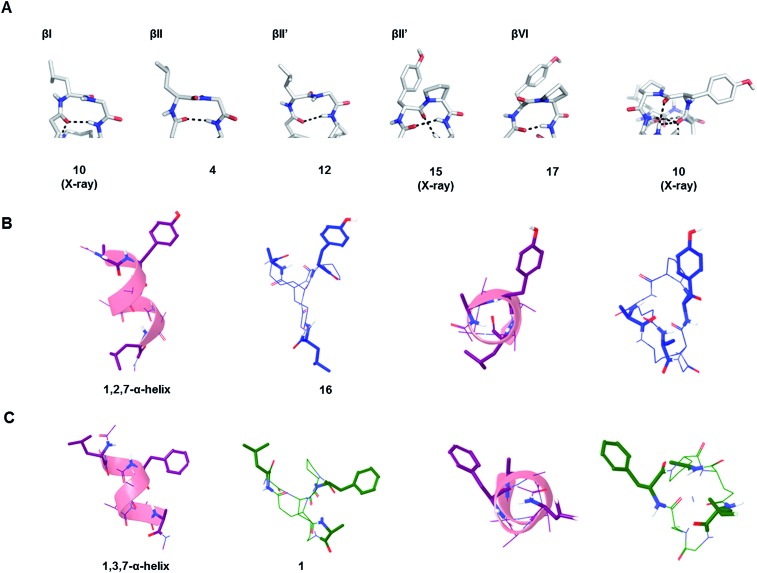
Similarity of BBP to secondary structures. (A). β-Turns in the leucine-loop and tyrosine-loop. The β-turn present in the tyrosine-loop of **10** is further stabilized by a hydrogen bond connection with the alanine-bridge, generating an unusual β-turn geometry. (B). Double turn α-helix with 1^st^, 2^nd^ and 7^th^ side chain highlighted and the corresponding BBP **16** shown in side-view (left) and top view (right). (C). Double turn α-helix with 1^st^, 3^rd^ and 7^th^ side chain highlighted and the corresponding BBP **1** shown in side-view (left) and top view (right).

The possible structural similarities between BBP scaffolds and secondary structure elements of peptides and proteins was analysed next. The dipeptide loops within the norbornapeptide displayed the geometry of various types of β-turns, with three cases of type I β-turn, fourteen cases of type II β-turns and six cases of type VI β-turn. In the case of BBP **10**, the tyrosine-containing loop adopted a geometry not documented in known β-turns, thus representing a new type of structural arrangement (Fig. 5A).

While the seven residues of a norbornapeptide are very different from the seven residues of α-helix double turn in terms of their topological connectivity, both backbones might present several of their amino acid side chains in comparable orientation. To test this hypothesis, all residue triplets in each of the norbornapeptide stereoisomers were compared pairwise with all possible residue triplets in a seven-residue α-helix double turn considering the relative positioning of the α-carbons, β-carbon atoms, or both simultaneously. The comparisons were carried out using an algorithm to optimize the spatial overlap between atoms measured by an atom-pair fitting function for small molecule comparisons.^31^ Thirty-five possible residue triplets in the α-helix were compared with the nine norbornapeptide diastereoisomers (Fig. S1†). The most similar triplet sets were observed between alanine, tyrosine, leucine in BBP **16** and the 1^st^, 2^nd^ and 7^th^ residues from the double turn of a α-helix, and between the corresponding residues in BBP **1** and 1^st^, 3^rd^ and 7^th^ residues from the double turn of a α-helix, suggesting that norbornapeptides might serve as stable analogs of α-helices for displaying three residues in these relative positions (Fig. 5B and C).

### Identification of a protein binding BBP

To test whether BBP might show specific binding to proteins, a proteomics based screening using capture compound mass spectroscopy (CCMS)^27^ was carried out as a proof-of-principle experiment. In CCMS proteins of a cell lysate binding specifically to a small molecule drug are identified as those proteins whose binding to a photoreactive, biotinylated analog of the drug is diminished or suppressed in the presence of the unlabeled drug as competitor. Proteins binding to the biotinylated drug are identified by removing them with streptavidin coated magnetic beads, washing, on-bead trypsin digestion and analysis of the resulting peptide fragments by LC/MSn.

**Scheme 2:**
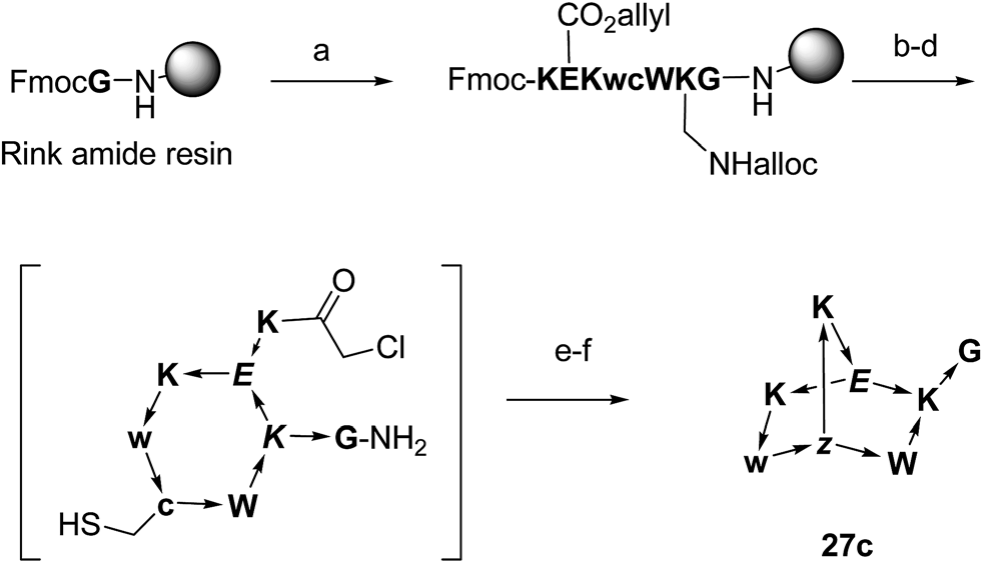
Synthesis of BBPs by ClAc ligation at the example of **27c**. One letter codes are used for amino acids, upper case letters = l-amino acids, lower case letters = d-amino acids, italics = branching amino acids, *z* = branching d-γ-thia-homoglutamate, arrow bonds indicate peptide bonds in C → N direction. Conditions: (a) Fmoc SPPS; (b) Pd(PPh_3_)_4_, PhSiH_3_, anh. CH_2_Cl_2_; (c) HATU, iPr_2_EtN, NMP–DMSO 4 : 1; (d) piperidine–DMF 1 : 4; (e) (ClCH_2_CO)_2_O, CH_2_Cl_2_; (f) CF_3_CO_2_H–iPr_3_SiH–H_2_O 94 : 5 : 1; (g) KI, iPr_2_EtN, CH_3_CN–H_2_O 1 : 1.

To obtain functionalized BBPs suitable for the CCMS experiment a modified synthesis was performed based on the chloroacetyl cysteine thioether (ClAc) ligation for the second cyclisation producing a BBP bearing a γ-thia-homoglutamate as the branching diamino acid (Scheme 2).^32^ In this ClAc approach to BBPs a linear peptide was synthesized by Fmoc-SPPS on a standard Rink-amide resin using an Alloc-protected lysine and a side-chain allyl protected glutamate to close the first cycle, and a cysteine to form the second cycle at the end of the synthesis. The first cyclisation was performed on-resin between the glutamate side-chain carboxyl group and the lysine side-chain amino group after Alloc/allyl deprotection. The N-terminal Fmoc group was then removed, the free N-terminus chloroacetylated, and the monocyclic peptide cleaved from the resin and deprotected by acidic treatment and purified by preparative HPLC. The second cyclisation was finally performed by ClAc ligation between the cysteine thiol and the N-terminal chloroacetyl group under basic conditions. This modified method gave BBPs of comparable structure to the double amide bond cyclization approach, but proved much more compatible with multiple protected side-chains as required for the CCMS experiment.

**Fig. 6:**
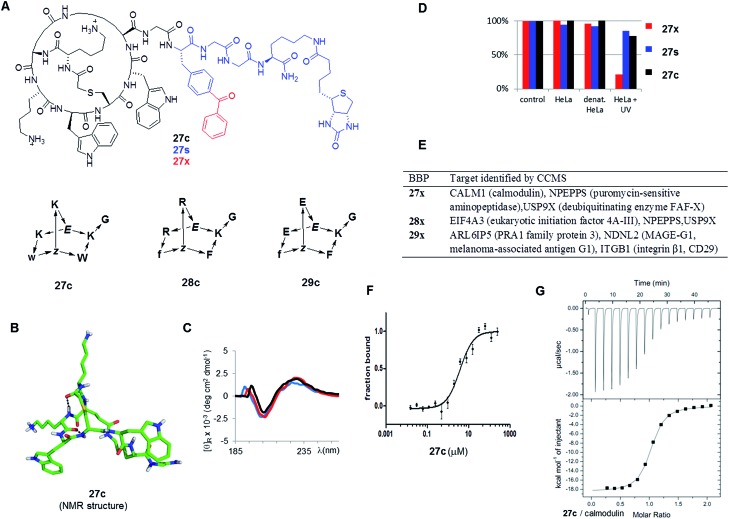
Identification of BBP protein binders. (A). Structure of the tested BBP. (B). Structure of **27c** determined by NMR. (C). Circular dichroism spectrum of **27c** in aq. 10 mM phosphate buffer pH = 7.0 (black), in 25% trifluoroethanol in aq. 10 mM phosphate buffer pH = 7.0 (red), and in aq. 6 M guanidinium chloride (blue). (D). Percentage of BBP **27x/s/c** detected by LC/MS of supernatant after incubation and centrifugation. Control = cell culture medium, 1 h, 4 °C; HeLa = HeLa cell lysate, 1 h, 37 °C; denat. HeLa = heat denaturated HeLa cell lysate, 1 h, 37 °C; HeLa + UV: HeLa cell lysate, irradiation with UV 310 nm for 10 min. Results were similar with **28x/s/c** and **29x/s/c** (data not shown) (E). Targets identified by CCMS. (F). MicroScale Thermophoresis of **27c** with CALM1 (calmodulin). (G). Isothermal titration calorimetry of **27c** with CALM1.

As possible protein binding BBPs three norbornapeptides **27c–29c** were prepared displaying a pair of aromatic residues (either phenylalanine or tryptophane) and two charged residues (glutamate, lysine or arginine) as side chains, hypothesizing that a combination of charged and aromatic residues might be favourable for protein binding. BBPs **27c–29c** contained glycine as an acyclic peptide portion, which was extended with the photoreactive benzoylphenylalanine in the photoreactive probes **27x–29x** or simply phenylalanine their non-photoreactive analogs **27s–29s** followed by a glycyl-glycine spacer and a biotinylated lysine (Fig. 6A and Table 3). The structure of BBP **27c** was determined by NMR and showed a compact bicyclic structure stabilized by intramolecular hydrogen bonds with a molecular shape comparable to a short stretch of an α-helix structure, presumably a favourable arrangement for protein binding (Fig. 6B). As for other norbornapeptides the CD spectrum of **27c** was unchanged in the presence of folding inducers or denaturants, suggesting conformational stability (Fig. 6C). The ability of the biotinylated probes **27x/s–29x/s** to bind to streptavidin was confirmed in a colorimetric HABA (4′-hydroxyazobenzene-2-carboxylic acid) displacement assay.

**Table 3:** Synthesis of BBP for CCMS experiments

No.	Length	Sequence^*a*^	mg	Yield^*b*^ (%)	[M + H]^+^ calc./obs.^*c*^
**27c**	8	**K** ^2^ ***E*** ^1^ **Kw*z*** ^2^ **WK** ^1^ **G**	19.0	11	1085.53/1085.52
**27x**	12	K^2^*E*^1^Kw*z*^2^WK^1^G**BGGK**_**(biot)**_	12.0	5	1804.85/1804.48
**27s**	12	K^2^*E*^1^Kw*z*^2^WK^1^G**FGGK**_**(biot)**_	12.3	5	1700.82/1700.47
**28c**	8	**R** ^2^ *E* ^1^ **Rf** *z* ^2^ **F**K^1^G	5.1	3	1063.52/1063.52
**28x**	12	**R** ^2^ *E* ^1^ **Rf** *z* ^2^ **F**K^1^G**BGGK**_**(biot)**_	7.9	3	892.42/892.42 (*z* = 2)
**28s**	12	**R** ^2^ *E* ^1^ **Rf** *z* ^2^ **F**K^1^G**FGGK**_**(biot)**_	10.5	4	839.91/840.50 (*z* = 2)
**29c**	8	**E** ^2^ *E* ^1^ **Ef** *z* ^2^ **F**K^1^G	1.2	1	1009.41/1009.41
**29x**	12	**E** ^2^ *E* ^1^ **Ef** *z* ^2^ **F**K^1^G**BGGK**_**(biot)**_	3.0	1	864.86/864.86 (*z* = 2)
**29s**	12	**E** ^2^ *E* ^1^ **Ef** *z* ^2^ **F**K^1^G**FGGK**_**(biot)**_	8.4	4	1624.69/1624.27

^*a*^BBP sequences as SMILES-like strings: superscript numbers indicate couple of residues involved in the first, second cyclization reactions. The C-termini are carboxamides CONH_2_. B = benzoylphenylalanine, K(Biot) = side-chain biotinylate lysine, *z* = branching γ-thia-homoglutamic acid.

^*b*^Yields are given as total yields (SPPS and second thioether ligation). In all cases, yields are calculated for the corresponding trifluoroacetate salts.

^*c*^ESI*-*MS positive mode spectra were recorded on a LTQ OrbitrapXL hybrid ion trap-Orbitrap mass spectrometer.

Incubation of the photoreactive BBPs **27x–29x** with a HeLa cell lysate under UV irradiation removed over 90% of the compounds from solution, while the non-photoreactive **27s–29s** and the parent BBP **27c–29c** were unaffected under the same conditions (Fig. 6D). Furthermore, the photoreactive BBPs **27x–29x** were unaffected without UV-irradiation, or with UV irradiation in buffer only, or with UV irradiation in presence of a thermally denatured HeLa cell lysate, indicating a specific cross-linking reactivity with protein components of the HeLa cell lysate. The CCMS experiment was carried by incubating HeLa cell lysates for 1 h at 4 °C with the photoreactive BBPs (**27x–29x**, 5 μM) or the unreactive phenylalanine analogs (**27s–29s**, 5 μM) with or without excess of the parent BBP as competitor (**27c–29c**, 240 μM), followed by a 10 min irradiation at 310 nm to allow for photo-cross-linking. The cross-linked products were then collected on streptavidin-coated magnetic beads, and analyzed by on-bead trypsin digestion followed by LC-MSn. MS data were analysed using MaxQuant^33^ using the human database from UniProtKB/Swiss-prot.21*b*

Focusing on proteins identified by at least two peptides with >99% confidence and significantly enriched in the **x** over **x** + **c** or in **s** over **s** + **c** experiments (>2-fold, triplicate experiments) revealed several potential protein binders for each of the three BBPs (Fig. 6E). Among the various hits, CALM1 (calmodulin) stood out as being specifically targeted by both **27x** and **27s**, suggesting that a covalent cross-link was not necessary. Furthermore, the related **28x** or **28s** with arginines replacing the lysine and phenylalanines replacing tryptophans did not indicate this target, suggesting specificity. The binding of **27c** to calmodulin was therefore investigated closer despite the fact that this protein is a relatively abundant component of the cell and a frequent hit in proteomics experiments. To test whether **27c** bound to calmodulin, the binding affinity was determined by microscale thermophoresis (MST, Fig. 6F).^34^ Indeed **27c** bound calmodulin with good affinity (*K*_D_ = 4.5 ± 1.1 μM). There was no measurable affinity for analog **28c** with arginine replacing lysines and phenylalanines replacing tryptophans, showing that binding was sequence specific, in line with the CCMS results where calmodulin was only indicated as a hit for **27c**. The measured affinity of **27c** was further confirmed by isothermal titration calorimetry (Fig. 6G), which gave a stronger binding affinity of *K*_D_ = 0.80 ± 0.05 μM. Binding was unchanged in the presence of excess Ca^2+^, but was reduced 25-fold in the presence of EDTA, indicating that **27c** interacted preferentially with calcium-bound calmodulin. The weaker binding observed by MST might reflect the much lower protein concentration used in MST (10 nM *vs.* 50 μM by ITC), or a perturbation by the fluorescence label coupled to calmodulin. ITC also indicated weak binding by analog BBP **28c** (*K*_D_ = 6.8 ± 0.6 μM).

Although the complex of calmodulin with **27c** did not yield to crystallization, we speculate that **27c** interacts with calmodulin by mimicking part of the α-helical IQ-motifs ((I/L/V)QXXXRXXXX(R/K)) found in the cardiac Ca_v_1.2 calcium channel binding with Ca^2+^-calmodulin.^35^ Although the **27c** consists of only eight residues, it contains sequence components comparable to the IQ motif. One of the tryptophans in **27c** might work as the first hydrophobic residue in IQ motif, while its C-terminal glycine amide could act similarly to the adjacent glutamine in the IQ motif. The two lysines might engage in further hydrophilic interactions and the remaining tryptophan can contribute more hydrophobicity.

### Pharmacokinetics

**Fig. 7:**
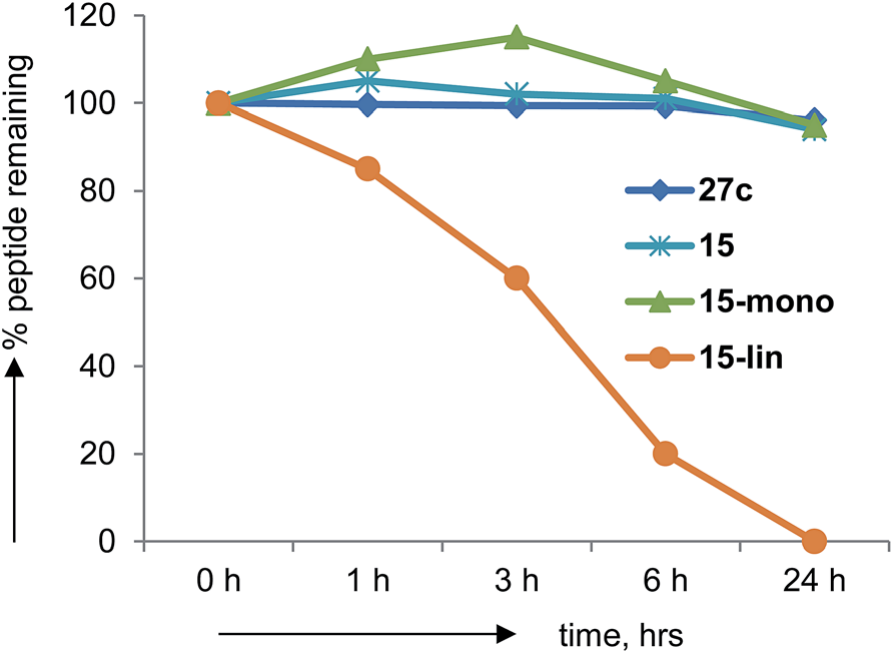
Human serum stability assays on norbornapeptide **27c**, **15** and its monocyclic and linear analogs.

Peptides generally suffer from rapid degradation by proteolysis in serum, and poor membrane permeability, which limits their use as drugs. The constrained nature of BBPs such as **27c** should limit sensitivity towards proteolysis. Indeed BBP **27c** was entirely stable upon incubation in human serum for 24 h at 37 °C. The same observation was made with several other BBPs including **15**, in which case the mono-cyclic analog obtained during synthesis was also stable, while its linear precursor was degraded (Fig. 7).

**Fig. 8:**
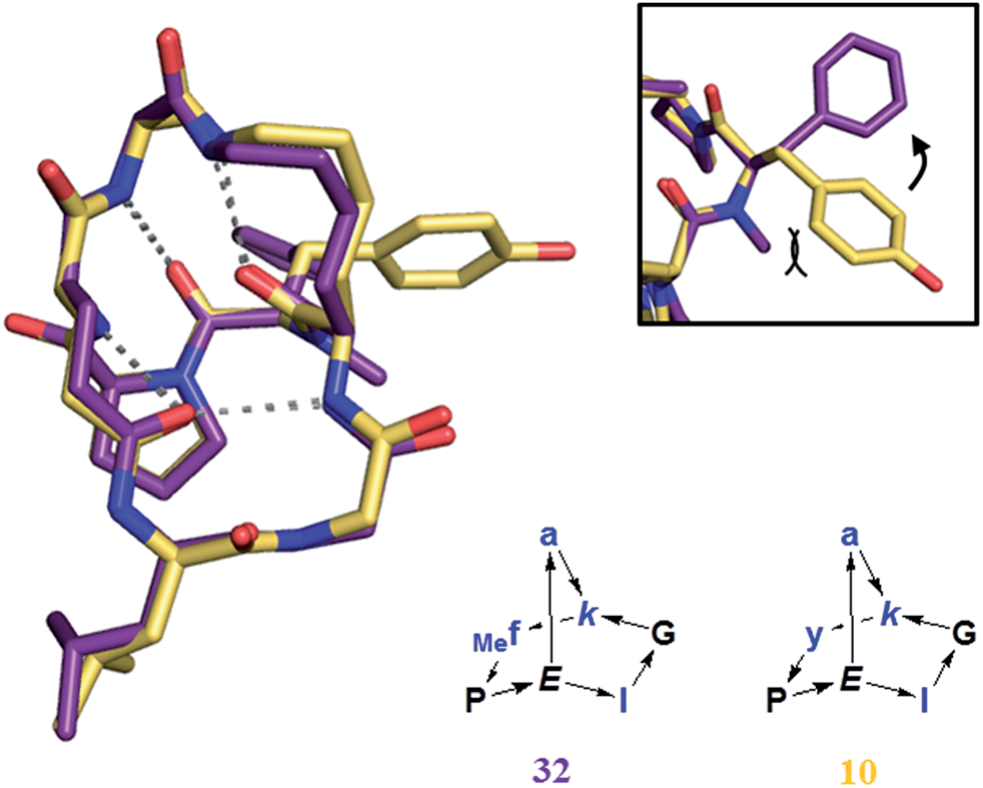
Crystal structure of **32** (purple), superimposed with **10** (yellow). *Frame*: detail of the orientation of the aromatic ring, tilted by the presence of the *N*-methyl group in **32**.

On the other hand BBPs showed the typical limitation of peptides in terms of membrane permeability when tested in a parallel artificial membrane permeation assay (PAMPA),^36^ with no measurable permeation through the membrane in comparison to the control peptide cyclosporine (data not shown). Cyclosporine is a cyclic peptide where all peptide bonds are *N*-methylated, which makes this compound much more hydrophobic than peptides bearing NH groups on their amides. Clearly, the formation of several intramolecular hydrogen bonds in BBPs was not sufficient to obtain significant membrane permeability. The possibility to increase BBP membrane permeation by *N*-methylation, in particular of backbone amide groups exposed to solvents25a,^37^ was investigated in the case of the water insoluble BBP **1**, focusing on the *N*-methylation of its solvent-exposed backbone amide at residues 1, 2 and 4. Single and double *N*-methyl derivatives **30–35** were obtained by attaching the *N*-methyl group at various positions during SPPS (Table 4).^6^ A crystal structure was obtained in the case of **32** showing that *N*-methylation did not affect the scaffold geometry and confirming methylation of a solvent exposed NH group (Fig. 8). However, none of these *N*-methylated BBP analogs showed measurable PAMPA permeability.

**Table 4:** Synthesis and yield for *N*-methylated norbornapeptides

BBP	Sequence	mg	Yield (%)	[M + H]^+^ calc.
**30**	_Me_l^1^G*k*^2^fP*E*^1^a^2^	2.2	1	739.41/739.41
**31**	l^1^_Me_G*k*^2^fP*E*^1^a^2^	6.2	3	739.41/739.41
**32**	l^1^G*k*^2^_Me_fP*E*^1^a^2^	4.3	2	739.41/739.41
**33**	_Me_l^1^_Me_G*k*^2^fP*E*^1^a^2^	11.5	5	753.43/753.43
**34**	_Me_l^1^G*k*^2^_Me_fP*E*^1^a^2^	13.2	5	753.43/753.43
**35**	l^1^_Me_G*k*^2^_Me_fP*E*^1^a^2^	11.1	5	753.43/753.43

## Conclusion

The experiments described above tested the potential of bridged bicyclic peptides (BBP) as a new type of scaffold for peptide-based drug design. A structural study focusing on norbornapeptides using X-ray crystallography, NMR and CD spectroscopy showed that BBP are rigid scaffolds for the display of amino acid side chains, however their precise geometry is influenced in non-trivial manner by residue stereochemistry. Selected dipeptide loops of the BBP mimic β-turns, while triplets of side-chains may adopt relative orientations comparable to those in α-helices. A proteomics based screening using capture compound mass spectrometry (CCMS) was used to identify **27c** as a binder for calmodulin, thus providing a proof-of-principle example that BBPs as small as norbornapeptide can show specific protein binding. This and other BBPs showed high stability towards serum proteolytic degradation. Future experiments will address the systematic exploration of BBP as protein binders.

## Experimental section

### Materials and reagents

All reagents were purchased either from Sigma Aldrich (St. Louis, MO, USA), Acros Organics (Thermo Fisher, Geel, Belgium) or Fluorochem (Hadfield, UK). Resins, coupling agents, amino acids and their derivatives were purchased from Advanced ChemTech (Louisville, KY, USA), Polypeptide Laboratories (Strasbourg, France), Iris Biotech (Marktredwitz, Germany), GLS Biochem (Shanghai, China), Rapp Polymere (Tübingen, Germany) or Novabiochem (Merck Biosciences, Läufelfingen, Switzerland).

### Analytics

Analytical RP-HPLC was performed with an Ultimate 3000 Rapid Separation LC System (DAD-3000RS diode array detector) using an Acclaim RSLC 120 C18 column (2.2 μm, 120 Å, 3 × 50 mm, flow 1.2 mL min^–1^) from Dionex (Sunnyvale, CA, USA). Data recording and processing was done with Dionex Chromeleon Management System (version 6.80). Preparative RP-HPLC was performed with a Waters Prep LC Controller System using a Dr. Maisch GmbH Reprospher column (C18-DE, 100 × 30 mm, particle size 5 μm, pore size 100 Å, flow rate 40 mL min^–1^). Compounds were detected by UV absorption at 214 nm using a Waters 486 Tunable Absorbance Detector. The following eluents were used for all RP-HPLC measurements: “A” (Milli-Q deionized H_2_O with 0.1% TFA); “D” (Milli-Q deionized H_2_O–HPLC-grade acetonitrile (10 : 90) with 0.1% TFA). LC-MS data were collected after coupling the analytical system described above with a LCQ Fleet Ion Trap mass spectrometer (Thermo Scientific, San Jose, CA, USA). LC-MS data recording and processing was done with Xcalibur (version 2.2, Thermo Scientific). High resolution MS spectra, recorded on a LTQ OrbitrapXL Hybrid Ion Trap-Orbitrap mass spectrometer (Thermo Scientific), were provided by the analytical service of the Department of Chemistry and Biochemistry at the University of Bern (group P.D. Dr. Stefan Schürch).

### Synthesis of monocyclic peptides on Wang resin

Peptide syntheses were performed manually in 10 mL polypropylene syringes (B. Braun, Melsungen, Germany) equipped with a polyethylene frit, a Teflon stopcock and stopper. DCM, NMP and MeOH were purchased in technical grade and used without prior distillation. Stirring of the reaction mixture at any given step described below was performed by attaching the closed syringe to a rotating axis. Wang resins pre-loaded with one amino acid were swollen in DCM for 10 min prior the start of the synthesis. At each step the Fmoc protecting group was removed with 6 mL of DMF–piperidine (4 : 1) for 10 min. After filtration the procedure was repeated and finally the resin was washed (2× each) with NMP, MeOH and DCM. For coupling, 3 eq. of Fmoc-protected amino acid, 3 eq. of PyBOP in 6 mL of NMP were added to the resin. 6 eq. of DIPEA were added and the reaction was stirred for 60 min (reaction times were prolonged to 120 min for couplings of bridgehead amino acids, which were performed with 2 eq. of both Fmoc-protected amino acid and HATU as coupling agent). The resin was then washed (2× each) with NMP, MeOH and DCM. After the last coupling, the polypropylene syringe was equipped with a septum and dried under vacuum for one hour. It was then swollen in dry DCM for 15 min under argon. To remove the allyl protecting group, solvent was removed, Pd(PPh_3_)_4_ (0.20 eq.) was dissolved in 6 mL of dry DCM and added to the resin under argon. Phenylsilane (20 eq.) was then added to the resin. The reaction was stirred under argon bubbling for 45 min. The reagents were then removed by filtration and the resin washed with DCM (6 mL, 2 × 15 min) and sodium diethylamino dithiocarboxylate (20 mM in DMF, 6 mL, 15 min). The last Fmoc protecting group was then removed as above. On resin cyclization was performed using HATU (3 eq.) and DIPEA (6 eq.) were added to the peptidyl-resins in 6 mL of NMP–DMSO (4 : 1) and the mixtures were stirred at room temperature for 2–6 h. The reagents were removed by filtration and the resins washed (2× each) with NMP, MeOH and DCM. The TNBS test was used to check the effectiveness of the cyclizations: if deemed necessary, the cyclization step was repeated. Cleavage was carried out by treating the resins with 6 mL of a TFA–TIPS–H_2_O (95 : 4 : 1) solution for 2 h. The peptide solutions were separated from the resin by filtration, evaporated and dried under high vacuum. The crudes were then dissolved in a H_2_O–CH_3_CN mixture, purified by preparative RP-HPLC and lyophilized again. Yields were calculated for the trifluoroacetate salts of the products.

### Synthesis of monocyclic peptides with Boc-protected side-chains on 2CT resin

Monocyclic peptides were synthesized on 2-chlorotrityl resin (0.68–1.63 mmol g^–1^). Elongation of the peptides by Fmoc SPPS, allyl ester deprotection and intramolecular cyclization were carried out with the same procedures described above on Wang resin. If the first amino acid was pre-loaded on the resin, the synthesis started with the coupling of the next amino acid. Otherwise, to load the first residue 2-chlorotrityl chloride resin was swollen in dry DCM for 10 min and the solvent was removed by filtration. 5 eq. of Fmoc-protected amino acid and 6 eq. DIPEA were dissolved in dry DCM (6 mL) and added to the resin. The reactions were stirred for 6–10 h at r.t., and the solution was removed by filtration. The resin was washed with DCM and then treated with DCM–MeOH–DIPEA (4 : 1 : 0.2) for 30 min, to saturate eventual unreacted chloride sites. The resin was then washed with NMP–MeOH–DCM (2× each) and the first Fmoc deprotection was performed. After SPPS and on-resin cyclization as above, cleavage was carried out by treating the resins with 6 mL of a AcOH–TFE–DCM (1 : 2 : 7) solution for 2 h. The peptide solutions were separated from the resin by filtration, evaporated and dried under high vacuum. To remove the Mtt protecting group, the product was redissolved in DCM (15 mL), TFA (150 μL) was added dropwise under stirring at r.t. (the solutions turned bright yellow). Reactions were monitored by RP-HPLC until complete disappearance of the starting materials. After 21 h the reactions were stopped by adding DIPEA (350 μL) and removing the solvent by evaporation. Alternatively cleavage and Mtt deprotection were carried out simultaneously by drying the resin under high vacuum for 2 h and then treating with consecutive batches of TFA–TIPS–DCM (1 : 0.5 : 98.5) (5 mL, 10 min, 5–7×). After the first 1–2 acidic treatments, the resins turn dark. The solutions were collected, neutralized with a slight excess of DIPEA and evaporated.

### Second cyclization

Bicyclic peptides were synthesized by subjecting monocyclic peptides to amide bond formation conditions in high dilution (0.5–1 mm). To a solution of PyBOP or HATU (3 eq.) and DIPEA (6 eq.) in DCM–DMF (20 : 1), a 5–10 mm solution of monocyclic peptide in DMF was slowly added dropwise at r.t. under vigorous stirring. The reaction was left stirring for 2 h at r.t. and monitored *via* RP-HPLC, until complete disappearance of the starting material. The reaction was then quenched by evaporation of the solvent. For the 2CT resin products, side chain protecting groups were removed as follows: the product was dissolved in CHCl_3_ (20 mL) and washed 3× with a saturated solution of KHSO_4_ and 1× with H_2_O; the organic phases were collected, dried over anhydrous Na_2_SO_4_ and evaporated. Then 8 mL of TFA–DCM (4 : 1) +0.1% TIPS was added to the remainder and the reactions were stirred at r.t. for 2 h. In both procedures, the crude was finally dissolved in a H_2_O–CH_3_CN mixture, purified by preparative RP-HPLC and lyophilized. Yields were calculated by considering the trifluoroacetate salts of monocyclic peptides as starting material.

### Synthesis using thioether ligation

BBPs **27–29** were synthesized by standard Fmoc-SPPS on TentaGel S RAM resin. Elongation of the sequences, Allyl/Alloc deprotection and the first on resin cyclization were carried out with the previously described procedures. The N-terminus was then chloroacetylated after the final Fmoc removal by treating the resin for 30 min with a solution of chloroacetic anhydride (10 eq.) in DCM (6 mL per syringe). The procedure was repeated once. The cleavage was then carried out by treating the resins with 6 mL of a TFA–TIPS–DODT–H_2_O (94 : 2.5 : 2.5 : 1) solution for 1 h or, alternatively, with a TFA–TIPS–H_2_O (95 : 4 : 1) solution previously purged with Ar (10 min). The peptide solutions were separated from the resin by filtration, evaporated and dried under high vacuum. The remainder was then redissolved in A and washed once with EA in a 100 mL separation funnel. The aqueous phase was then lyophilized. The effectiveness of the washing step was monitored by analytical RP-HPLC. The resulting crude monocyclic peptides were then subjected to thioether ligation conditions in high dilution. Potassium iodide (20 eq.) and DIPEA (50 eq.) were dissolved in 100 mL of H_2_O–CH_3_CN (1 : 1) and the solutions were purged with Ar for 10 min. N-term chloroacetylated monocyclic peptide crudes were dissolved in 5 mL of H_2_O–CH_3_CN (1 : 1), the solutions purged with Ar for 10 minutes and finally added dropwise to the first solution. The reactions were left under stirring at r.t. until complete disappearance of the starting material, as revealed by analytical RP-HPLC. The mixtures were then flash-frozen, lyophilized and purified by preparative RP-HPLC. Yields were calculated on trifluoroacetate salts of bicyclic peptides and by considering trifluoroacetate salts of monocyclic peptides as starting material.

### Synthesis of *N*-methylated BBPs

*N*-Methylated BBPs were prepared using Fmoc synthesis procedure on 2CT resin described above. *N*-Methylation was performed following the literature procedure^6^ at the desired position after Fmoc removal. The resin was treated with *o*-NBS and collidine and rotated for 30 minutes then washed with DCM. For *N*-methylation, DBU and NMP were added to the resin followed by dimethyl sulfate and the syringe rotated for 8 minutes then washed with DCM. For *o*-NBS deprotection, DBU and mercaptoethanol were added and rotated for 10 minutes. Then the resin was washed using NMP, Methanol and DCM. The chloranil test was used to follow the release of the *N*-methylated amine.

#### l^1^GkfPE^1^a (**1**-*mono*)

From Fmoc-d-Ala-Wang resin (400 mg, 0.44 mmol g^–1^), **1**-*mono* was obtained as foamy white solid after preparative RP-HPLC (50.6 mg, 59.1 μmol, 34%). Analytical RP-HPLC: *t*_R_ = 3.148 min (*A*/*D* 100 : 0 to 0 : 100 in 7.5 min, *λ* = 214 nm). MS (ESI+): C_36_H_54_N_8_O_9_ calc./obs. 743.41/743.41 [M + H]^+^.

#### l^1^G*k*^2^fP*E*^1^a^2^ (**1**)

Starting from **1**-*mono* (50.6 mg, 59.1 μmol), **1** was obtained as foamy white solid after preparative RP-HPLC (34.9 mg, 48.1 μmol, 82%). Analytical RP-HPLC: *t*_R_ = 3.31 min (*A*/*D* 100 : 0 to 0 : 100 in 7.5 min, *λ* = 214 nm). MS (ESI+): C_36_H_52_N_8_O_8_ calc./obs. 725.40/725.40 [M + H]^+^, 747.38/747.38 [M + Na]^+^. ^1^H-NMR assignment available in electronic format.

#### l^1^GkfPE^1^A (**2**-*mono*)

From Fmoc-Ala-Wang resin (300 mg, 0.57 mmol g^–1^), **2**-*mono* was obtained as foamy white solid after preparative RP-HPLC (37.1 mg, 43.3 μmol, 25%). Analytical RP-HPLC: *t*_R_ = 3.12 min (*A*/*D* 100 : 0 to 0 : 100 in 7.5 min, *λ* = 214 nm). MS (ESI+): C_36_H_54_N_8_O_9_ calc./obs. 743.41/743.41 [M + H]^+^.

#### l^1^G*k*^2^fP*E*^1^A^2^ (**2**)

Starting from **2**-*mono* (37.1 mg, 43.3 μmol), **2** was obtained as foamy white solid after preparative RP-HPLC (25.9 mg, 29.5 μmol, 60%). Analytical RP-HPLC: *t*_R_ = 3.31 min (*A*/*D* 100 : 0 to 0 : 100 in 7.5 min, *λ* = 214 nm). MS (ESI+): C_36_H_52_N_8_O_8_ calc./obs. 725.40/725.40 [M + H]^+^, 747.38/747.38 [M + Na]^+^. ^1^H-NMR assignment available in electronic format.

#### l^1^GkFPE^1^a (**3**-*mono*)

From Fmoc-d-Ala-Wang resin (400 mg, 0.44 mmol g^–1^), **3**-*mono* was obtained as foamy white solid after preparative RP-HPLC (23.9 mg, 27.9 μmol, 16%). Analytical RP-HPLC: *t*_R_ = 3.22 min (*A*/*D* 100 : 0 to 0 : 100 in 7.5 min, *λ* = 214 nm). MS (ESI+): C_36_H_54_N_8_O_9_ calc./obs. 743.41/743.41 [M + H]^+^.

#### l^1^G*k*^2^FP*E*^1^a^2^ (**3**)

Starting from **3**-*mono* (23.9 mg, 27.9 μmol), **3** was obtained as foamy white solid after preparative RP-HPLC (11.4 mg, 15.7 μmol, 56%). Analytical RP-HPLC: *t*_R_ = 3.52 min (*A*/*D* 100 : 0 to 0 : 100 in 7.5 min, *λ* = 214 nm). MS (ESI+): C_36_H_52_N_8_O_8_ calc./obs. 725.40/725.40 [M + H]^+^, 747.38/747.38 [M + Na]^+^.

#### L^1^GkfPE^1^a (**4**-*mono*)

From Fmoc-d-Ala-Wang resin (400 mg, 0.44 mmol g^–1^), **4**-*mono* was obtained as foamy white solid after preparative RP-HPLC (62.7 mg, 73.2 μmol, 42%). Analytical RP-HPLC: *t*_R_ = 3.20 min (*A*/*D* 100 : 0 to 0 : 100 in 7.5 min, *λ* = 214 nm). MS (ESI+): C_36_H_54_N_8_O_9_ calc./obs. 743.41/743.41 [M + H]^+^.

#### L^1^G*k*^2^fP*E*^1^a^2^ (**4**)

Starting from **4**-*mono* (62.7 mg, 73.2 μmol), **4** was obtained as foamy white solid after preparative RP-HPLC (35.2 mg, 48.6 μmol, 66%). Analytical RP-HPLC: *t*_R_ = 3.51 min (*A*/*D* 100 : 0 to 0 : 100 in 7.5 min, *λ* = 214 nm). MS (ESI+): C_36_H_52_N_8_O_8_ calc./obs. 725.40/725.40 [M + H]^+^, 747.38/747.38 [M + Na]^+^.

#### l^1^GkFPE^1^A (**5**-*mono*)

From Fmoc-Ala-Wang resin (300 mg, 0.57 mmol g^–1^), **5**-*mono* was obtained as foamy white solid after preparative RP-HPLC (17.2 mg, 20.1 μmol, 12%). Analytical RP-HPLC: *t*_R_ = 3.01 min (*A*/*D* 100 : 0 to 0 : 100 in 7.5 min, *λ* = 214 nm). MS (ESI+): C_36_H_54_N_8_O_9_ calc./obs. 743.41/743.41 [M + H]^+^.

#### l^1^G*k*^2^FP*E*^1^A^2^ (**5**)

Starting from **5**-*mono* (17.2 mg, 20.1 μmol), **5** was obtained as foamy white solid after preparative RP-HPLC (8.6 mg, 11.9 μmol, 59%). Analytical RP-HPLC: *t*_R_ = 3.03 min (*A*/*D* 100 : 0 to 0 : 100 in 7.5 min, *λ* = 214 nm). MS (ESI+): C_36_H_52_N_8_O_8_ calc./obs. 725.40/725.40 [M + H]^+^, 747.38/747.38 [M + Na]^+^.

#### L^1^GkfPE^1^A (**6**-*mono*)

From Fmoc-Ala-Wang resin (300 mg, 0.57 mmol g^–1^), **6**-*mono* was obtained as foamy white solid after preparative RP-HPLC (43.5 mg, 50.8 μmol, 30%). Analytical RP-HPLC: *t*_R_ = 2.92 min (*A*/*D* 100 : 0 to 0 : 100 in 7.5 min, *λ* = 214 nm). MS (ESI+): C_36_H_54_N_8_O_9_ calc./obs. 743.41/743.41 [M + H]^+^.

#### L^1^G*k*^2^fP*E*^1^A^2^ (**6**)

Starting from **6**-*mono* (43.5 mg, 50.8 μmol), **6** was obtained as foamy white solid after preparative RP-HPLC (22.4 mg, 30.9 μmol, 61%). Analytical RP-HPLC: *t*_R_ = 3.38 min (*A*/*D* 100 : 0 to 0 : 100 in 7.5 min, *λ* = 214 nm). MS (ESI+): C_36_H_52_N_8_O_8_ calc./obs. 725.40/725.40 [M + H]^+^, 747.38/747.38 [M + Na]^+^.

#### L^1^GkFPE^1^a (**7**-*mono*)

From Fmoc-d-Ala-Wang resin (400 mg, 0.44 mmol g^–1^), **7**-*mono* was obtained as foamy white solid after preparative RP-HPLC (58.1 mg, 67.8 μmol, 39%). Analytical RP-HPLC: *t*_R_ = 3.13 min (*A*/*D* 100 : 0 to 0 : 100 in 7.5 min, *λ* = 214 nm). MS (ESI+): C_36_H_54_N_8_O_9_ calc./obs. 743.41/743.41 [M + H]^+^.

#### L^1^G*k*^2^FP*E*^1^a^2^ (**7**)

Starting from **7**-*mono* (58.1 mg, 67.8 μmol), **7** was obtained as foamy white solid after preparative RP-HPLC (17.5 mg, 24.1 μmol, 36%). Analytical RP-HPLC: *t*_R_ = 3.47 min (*A*/*D* 100 : 0 to 0 : 100 in 7.5 min, *λ* = 214 nm). MS (ESI+): C_36_H_52_N_8_O_8_ calc./obs. 725.40/725.40 [M + H]^+^, 747.38/747.38 [M + Na]^+^.

#### L^1^GkFPE^1^A (**8**-*mono*)

From Fmoc-Ala-Wang resin (300 mg, 0.57 mmol g^–1^), **8**-*mono* was obtained as foamy white solid after preparative RP-HPLC (55.6 mg, 64.9 μmol, 38%). Analytical RP-HPLC: *t*_R_ = 2.86 min (*A*/*D* 100 : 0 to 0 : 100 in 7.5 min, *λ* = 214 nm). MS (ESI+): C_36_H_54_N_8_O_9_ calc./obs. 743.41/743.41 [M + H]^+^.

#### L^1^G*k*^2^FP*E*^1^A^2^ (**8**)

Starting from **8**-*mono* (50.2 mg, 59.1 μmol), **8** was obtained as foamy white solid after preparative RP-HPLC (34.3 mg, 47.3 μmol, 81%). Analytical RP-HPLC: *t*_R_ = 3.34 min (*A*/*D* 100 : 0 to 0 : 100 in 7.5 min, *λ* = 214 nm). MS (ESI+): C_36_H_52_N_8_O_8_ calc./obs. 725.40/725.40 [M + H]^+^, 747.38/747.38 [M + Na]^+^.

#### L^1^GKFPE^1^A (**9**-*mono*)

From Fmoc-Ala-Wang resin (300 mg, 0.57 mmol g^–1^), **9**-*mono* was obtained as foamy white solid after preparative RP-HPLC (43.5 mg, 59.1 μmol, 30%). Analytical RP-HPLC: *t*_R_ = 3.17 min (*A*/*D* 100 : 0 to 0 : 100 in 7.5 min, *λ* = 214 nm). MS (ESI+): C_36_H_54_N_8_O_9_ calc./obs. 743.41/743.41 [M + H]^+^.

#### L^1^G*K*^2^FP*E*^1^A^2^ (**9**)

Starting from **9**-*mono* (28.9 mg, 33.7 μmol), **9** was obtained as foamy white solid after preparative RP-HPLC (6.6 mg, 9.1 μmol, 27%). Analytical RP-HPLC: *t*_R_ = 2.98 min (*A*/*D* 100 : 0 to 0 : 100 in 7.5 min, *λ* = 214 nm). MS (ESI+): C_36_H_52_N_8_O_8_ calc./obs. 725.40/725.40 [M + H]^+^, 747.38/747.38 [M + Na]^+^.

#### l^1^G*k*^2^yP*E*^1^a^2^ (**10**)

From 2CT-Cl resin (200 mg, 1.3 mmol g^–1^), **10** was obtained as foamy white solid after preparative RP-HPLC (8.9 mg, 12.0 μmol, 4%). Analytical RP-HPLC: *t*_R_ = 2.87 min (*A*/*D* 100 : 0 to 0 : 100 in 7.5 min, *λ* = 214 nm). MS (ESI+): C_36_H_52_N_8_O_9_ calc./obs. 741.39/741.39 [M + H]^+^, 763.37/763.37 [M + Na]^+^, 779.35/779.35 [M + K]^+^, 390.17/390.17 [M + K + H]^2+^.

#### l^1^GkyPE^1^A (**11**-*mono*)

From Fmoc-Ala-Wang resin (300 mg, 0.57 mmol g^–1^), **11**-*mono* was obtained as foamy white solid after preparative RP-HPLC (22.9 mg, 26.2 μmol, 26%). Analytical RP-HPLC: *t*_R_ = 2.58 min (*A*/*D* 100 : 0 to 0 : 100 in 7.5 min, *λ* = 214 nm). MS (ESI+): C_36_H_54_N_8_O_10_ calc./obs. 759.40/759.45 [M + H]^+^.

#### l^1^G*k*^2^yP*E*^1^A^2^ (**11**)

Starting from **11**-*mono* (21.7 mg, 24.9 μmol), **11** was obtained as foamy white solid after preparative RP-HPLC (10.9 mg, 14.7 μmol, 59%). Analytical RP-HPLC: *t*_R_ = 2.79 min (*A*/*D* 100 : 0 to 0 : 100 in 7.5 min, *λ* = 214 nm). MS (ESI+): C_36_H_52_N_8_O_8_ calc./obs. 741.39/741.42 [M + H]^+^.

#### l^1^GkYPE^1^a (**12**-*mono*)

From Fmoc-d-Ala-Wang resin (300 mg, 0.44 mmol g^–1^), **12**-*mono* was obtained as foamy white solid after preparative RP-HPLC (16.3 mg, 18.7 μmol, 12%). Analytical RP-HPLC: *t*_R_ = 2.66 min (*A*/*D* 100 : 0 to 0 : 100 in 7.5 min, *λ* = 214 nm). MS (ESI+): C_36_H_54_N_8_O_10_ calc./obs. 759.40/759.64 [M + H]^+^.

#### l^1^G*k*^2^YP*E*^1^a^2^ (**12**)

Starting from **12**-*mono* (15.1 mg, 17.3 μmol), **12** was obtained as foamy white solid after preparative RP-HPLC (4.7 mg, 6.3 μmol, 37%). Analytical RP-HPLC: *t*_R_ = 2.97 min (*A*/*D* 100 : 0 to 0 : 100 in 7.5 min, *λ* = 214 nm). MS (ESI+): C_36_H_52_N_8_O_8_ calc./obs. 741.39/741.42 [M + H]^+^.

#### L^1^GkyPE^1^a (**13**-*mono*)

From Fmoc-d-Ala-Wang resin (300 mg, 0.44 mmol g^–1^), **13**-*mono* was obtained as foamy white solid after preparative RP-HPLC (36.8 mg, 42.2 μmol, 28%). Analytical RP-HPLC: *t*_R_ = 2.58 min (*A*/*D* 100 : 0 to 0 : 100 in 7.5 min, *λ* = 214 nm). MS (ESI+): C_36_H_54_N_8_O_10_ calc./obs. 759.40/759.48 [M + H]^+^.

#### L^1^G*k*^2^yP*E*^1^a^2^ (**13**)

Starting from **13***-mono* (35.0 mg, 40.1 μmol), **13** was obtained as foamy white solid after preparative RP-HPLC (21.8 mg, 29.4 μmol, 73%). Analytical RP-HPLC: *t*_R_ = 2.91 min (*A*/*D* 100 : 0 to 0 : 100 in 7.5 min, *λ* = 214 nm). MS (ESI+): C_36_H_52_N_8_O_8_ calc./obs. 741.39/741.40 [M + H]^+^.

#### l^1^GkYPE^1^A (**14**-*mono*)

From Fmoc-Ala-Wang resin (300 mg, 0.57 mmol g^–1^), **14**-*mono* was obtained as foamy white solid after preparative RP-HPLC (18.3 mg, 21.0 μmol, 14%). Analytical RP-HPLC: *t*_R_ = 2.56 min (*A*/*D* 100 : 0 to 0 : 100 in 7.5 min, *λ* = 214 nm). MS (ESI+): C_36_H_54_N_8_O_10_ calc./obs. 759.40/759.68 [M + H]^+^.

#### l^1^G*k*^2^YP*E*^1^A^2^ (**14**)

Starting from **14**-*mono* (17.0 mg, 19.5 μmol), **14** was obtained as foamy white solid after preparative RP-HPLC (7.0 mg, 9.5 μmol, 49%). Analytical RP-HPLC: *t*_R_ = 2.74 min (*A*/*D* 100 : 0 to 0 : 100 in 7.5 min, *λ* = 214 nm). MS (ESI+): C_36_H_52_N_8_O_8_ calc./obs. 741.39/741.43 [M + H]^+^.

#### L^1^Gky_(tBu)_PE^1^A (**15**-*mono*)

From H-l-Ala-2CT resin (350 mg, 0.68 mmol g^–1^), **15**-*mono* was obtained as foamy white solid after preparative RP-HPLC (60.7 mg, 65.3 μmol, 28%). Analytical RP-HPLC: *t*_R_ = 3.67 min (*A*/*D* 100 : 0 to 0 : 100 in 7.5 min, *λ* = 214 nm). MS (ESI+): C_40_H_62_N_8_O_10_ calc./obs. 815.47/815.47 [M + H]^+^.

#### L^1^G*k*^2^yP*E*^1^A^2^ (**15**)

Starting from **15**-*mono* (60.7 mg, 65.3 μmol), **15** was obtained as foamy white solid after preparative RP-HPLC (20.5 mg, 27.7 μmol, 42%). Analytical RP-HPLC: *t*_R_ = 3.02 min (*A*/*D* 100 : 0 to 0 : 100 in 7.5 min, *λ* = 214 nm). MS (ESI+): C_36_H_52_N_8_O_9_ calc./obs. 741.39/741.39 [M + H]^+^, 763.37/763.37 [M + Na]^+^.

#### L^1^GkYPE^1^a (**16**-*mono*)

From Fmoc-d-Ala-Wang resin (300 mg, 0.44 mmol g^–1^), **16**-*mono* was obtained as foamy white solid after preparative RP-HPLC (45.4 mg, 52.0 μmol, 35%). Analytical RP-HPLC: *t*_R_ = 2.53 min (*A*/*D* 100 : 0 to 0 : 100 in 7.5 min, *λ* = 214 nm). MS (ESI+): C_36_H_54_N_8_O_10_ calc./obs. 759.40/759.48 [M + H]^+^.

#### L^1^G*k*^2^YP*E*^1^a^2^ (**16**)

Starting from **16**-*mono* (29.7 mg, 34.0 μmol), **16** was obtained as foamy white solid after preparative RP-HPLC (18.1 mg, 24.4 μmol, 72%). Analytical RP-HPLC: *t*_R_ = 2.93 min (*A*/*D* 100 : 0 to 0 : 100 in 7.5 min, *λ* = 214 nm). MS (ESI+): C_36_H_52_N_8_O_8_ calc./obs. 741.39/741.41 [M + H]^+^.

#### L^1^GkYPE^1^A (**17**-*mono*)

From Fmoc-Ala-Wang resin (300 mg, 0.57 mmol g^–1^), **17**-*mono* was obtained as foamy white solid after preparative RP-HPLC (58.6 mg, 67.1 μmol, 45%). Analytical RP-HPLC: *t*_R_ = 2.48 min (*A*/*D* 100 : 0 to 0 : 100 in 7.5 min, *λ* = 214 nm). MS (ESI+): C_36_H_54_N_8_O_10_ calc./obs. 759.40/759.49 [M + H]^+^.

#### L^1^G*k*^2^YP*E*^1^A^2^ (**17**)

Starting from **17**-*mono* (56.8 mg, 65.1 μmol), **17** was obtained as foamy white solid after preparative RP-HPLC (7.3 mg, 9.9 μmol, 15%). Analytical RP-HPLC: *t*_R_ = 2.87 min (*A*/*D* 100 : 0 to 0 : 100 in 7.5 min, *λ* = 214 nm). MS (ESI+): C_36_H_52_N_8_O_8_ calc./obs. 741.39/741.45 [M + H]^+^.

#### L^1^GKYPE^1^A (**18**-*mono*)

From Fmoc-Ala-Wang resin (300 mg, 0.57 mmol g^–1^), **18**-*mono* was obtained as foamy white solid after preparative RP-HPLC (45.7 mg, 52.3 μmol, 35%). Analytical RP-HPLC: *t*_R_ = 2.66 min (*A*/*D* 100 : 0 to 0 : 100 in 7.5 min, *λ* = 214 nm). MS (ESI+): C_36_H_54_N_8_O_10_ calc./obs. 759.40/759.48 [M + H]^+^.

#### L^1^G*K*^2^YP*E*^1^A^2^ (**18**)

Starting from **18**-*mono* (43.8 mg, 50.2 μmol), **18** was obtained as foamy white solid after preparative RP-HPLC (13.8 mg, 18.6 μmol, 37%). Analytical RP-HPLC: *t*_R_ = 2.72 min (*A*/*D* 100 : 0 to 0 : 100 in 7.5 min, *λ* = 214 nm). MS (ESI+): C_36_H_52_N_8_O_8_ calc./obs. 741.39/741.40 [M + H]^+^.

#### k^1^G*k*^2^fP*E*^1^a^2^ (**19**)

From 2CT-Cl resin (200 mg, 1.3 mmol g^–1^), **19** was obtained as foamy white solid after preparative RP-HPLC (6.6 mg, 7.7 μmol, 2.4%). Analytical RP-HPLC: *t*_R_ = 2.78 min (*A*/*D* 100 : 0 to 0 : 100 in 7.5 min, *λ* = 214 nm). MS (ESI+): C_36_H_53_N_9_O_8_ calc./obs. 740.41/740.41 [M + H]^+^, 762.39/762.39 [M + Na]^+^, 389.68/389.68 [M + K + H]^2+^.

#### l^1^S*k*^2^fP*E*^1^a^2^ (**20**)

From 2CT-Cl resin (200 mg, 1.3 mmol g^–1^), **20** was obtained as foamy white solid after preparative RP-HPLC (23.4 mg, 31.0 μmol, 9.5%). Analytical RP-HPLC: *t*_R_ = 3.18 min (*A*/*D* 100 : 0 to 0 : 100 in 7.5 min, *λ* = 214 nm). MS (ESI+): C_37_H_54_N_8_O_9_ calc./obs. 755.41/755.41 [M + H]^+^, 777.39/777.39 [M + Na]^+^, 397.18/397.18 [M + K + H]^2+^, 793.36/793.36 [M + K]^+^, 389.19 [M + Na + H]^2+^.

#### l^1^G*k*^2^fK*E*^1^a^2^ (**21**)

From 2CT-Cl resin (200 mg, 1.3 mmol g^–1^), **21** was obtained as a white solid after preparative RP-HPLC (7.7 mg, 8.9 μmol, 2.7%). Analytical RP-HPLC: *t*_R_ = 2.69 min (*A*/*D* 100 : 0 to 0 : 100 in 7.5 min, *λ* = 214 nm). MS (ESI+): C_37_H_57_N_9_O_8_ calc./obs. 756.44/756.44 [M + H]^+^, 778.42/778.42 [M + Na]^+^.

#### k^1^G*k*^2^eP*E*^1^c^2^ (**22**)

From 2CT-Cl resin (200 mg, 1.3 mmol g^–1^), **22** was obtained as a white solid after preparative RP-HPLC (6.4 mg, 7.4 μmol, 2.3%). Analytical RP-HPLC: *t*_R_ = 1.92 min (*A*/*D* 100 : 0 to 0 : 100 in 7.5 min, *λ* = 214 nm). MS (ESI+): C_32_H_51_N_9_O_10_S calc./obs. 754.36/754.35 [M + H]^+^, 776.34/776.34 [M + Na]^+^, 396.65/396.65 [M + K + H]^2+^.

#### K_(Boc)_^1^GkfPE^1^A (**23**-*mono*)

From H-l-Ala-2CT resin (350 mg, 0.68 mmol g^–1^), **23**-*mono* was obtained as foamy white solid after preparative RP-HPLC (32.7 mg, 33.6 μmol, 14%). Analytical RP-HPLC: *t*_R_ = 3.53 min (*A*/*D* 100 : 0 to 0 : 100 in 7.5 min, *λ* = 214 nm). MS (ESI+): C_41_H_63_N_9_O_11_ calc./obs. 858.47/858.47 [M + H]^+^.

#### K^1^G*k*^2^fP*E*^1^A^2^ (**23**)

Starting from **23**-*mono* (32.7 mg, 33.6 μmol), **23** was obtained as glassy white solid after preparative RP-HPLC (15.0 mg, 17.6 μmol, 52%). Analytical RP-HPLC: *t*_R_ = 2.64 min (*A*/*D* 100 : 0 to 0 : 100 in 7.5 min, *λ* = 214 nm). MS (ESI+): C_36_H_53_N_9_O_8_ calc./obs. 740.41/740.41 [M + H]^+^, 370.71/370.71 [M+2H]^2+^.

#### L^1^S*k*^2^fP*E*^1^A^2^ (**24**)

From 2CT-Cl resin (200 mg, 1.3 mmol g^–1^), **24** was obtained as foamy white solid after preparative RP-HPLC (12.2 mg, 16.2 μmol, 6%). Analytical RP-HPLC: *t*_R_ = 3.16 min (*A*/*D* 100 : 0 to 0 : 100 in 7.5 min, *λ* = 214 nm). MS (ESI+): C_37_H_54_N_8_O_9_ calc./obs. 755.41/755.41 [M + H]^+^, 777.39/777.39 [M + Na]^+^.

#### L^1^G*k*^2^fK*E*^1^A^2^ (**25**)

From 2CT-Cl resin (200 mg, 1.3 mmol g^–1^) **25** was obtained as foamy white solid after preparative RP-HPLC (12.0 mg, 16.2 μmol, 5%). Analytical RP-HPLC: *t*_R_ = 2.56 min (*A*/*D* 100 : 0 to 0 : 100 in 7.5 min, *λ* = 214 nm). MS (ESI+): C_37_H_57_N_9_O_8_ calc./obs. 756.44/756.44 [M + H]^+^, 778.42/778.42 [M + Na]^+^.

#### K^1^G*k*^2^eP*E*^1^C^2^ (**26**)

From 2CT-Cl resin (200 mg, 1.3 mmol g^–1^), **26** was obtained as foamy white solid after preparative RP-HPLC (5.3 mg, 6.1 μmol, 2%). Analytical RP-HPLC: *t*_R_ = 1.91 min (*A*/*D* 100 : 0 to 0 : 100 in 7.5 min, *λ* = 214 nm). MS (ESI+): C_37_H_53_N_9_O_9_ calc./obs. 754.35/754.35 [M + H]^+^.

#### K^2^*E*^1^Kw*z*^2^WK^1^G (**27c**)

From TentaGel S RAM resin (500 mg, 0.26 mmol g^–1^), **27c** was obtained as foamy white solid after preparative RP-HPLC (19.0 mg, 17.5 μmol, 11%). Analytical RP-HPLC: *t*_R_ = 2.78 min (*A*/*D* 100 : 0 to 0 : 100 in 7.5 min, *λ* = 214 nm). MS (ESI+): C_52_H_72_N_14_O_10_S calc./obs. 1085.53/1085.52 [M + H]^+^.

#### K^2^*E*^1^Kw*z*^2^WK^1^GBGGK_(biot)_ (**27x**)

From TentaGel S RAM resin (500 mg, 0.26 mmol g^–1^), **27x** was obtained as foamy white solid after preparative RP-HPLC (12.0 mg, 6.7 μmol, 5%). Analytical RP-HPLC: *t*_R_ = 3.51 min (*A*/*D* 100 : 0 to 0 : 100 in 7.5 min, *λ* = 214 nm). MS (ESI+): C_88_H_117_N_21_O_17_S_2_ calc./obs. 1804.85/1804.48 [M + H]^+^.

#### K^2^*E*^1^Kw*z*^2^WK^1^GFGGK_(biot)_ (**27s**)

From TentaGel S RAM resin (500 mg, 0.26 mmol g^–1^), **27s** was obtained as foamy white solid after preparative RP-HPLC (12.3 mg, 7.2 μmol, 5%). Analytical RP-HPLC: *t*_R_ = 3.22 min (*A*/*D* 100 : 0 to 0 : 100 in 7.5 min, *λ* = 214 nm). MS (ESI+): C_81_H_113_N_21_O_16_S_2_ calc./obs. 1700.82/1700.47 [M + H]^+^.

#### R^2^*E*^1^Rf*z*^2^FK^1^G (**28c**)

From TentaGel S RAM resin (500 mg, 0.26 mmol g^–1^), **29c** was obtained as foamy white solid after preparative RP-HPLC (5.1 mg, 4.8 μmol, 3%). Analytical RP-HPLC: *t*_R_ = 2.75 min (*A*/*D* 100 : 0 to 0 : 100 in 7.5 min, *λ* = 214 nm). MS (ESI+): C_48_H_70_N_16_O_10_S calc./obs. 1063.52/1063.52 [M + H]^+^.

#### R^2^*E*^1^Rf*z*^2^FK^1^GBGGK_(biot)_ (**28x**)

From TentaGel S RAM resin (500 mg, 0.26 mmol g^–1^), **29x** was obtained as foamy white solid after preparative RP-HPLC (7.9 mg, 4.4 μmol, 3%). Analytical RP-HPLC: *t*_R_ = 3.59 min (*A*/*D* 100 : 0 to 0 : 100 in 7.5 min, *λ* = 214 nm). MS (ESI+): C_84_H_115_N_23_O_17_S_2_ calc./obs. 892.42/892.42 [M + 2H]^2+^.

#### R^2^*E*^1^Rf*z*^2^FK^1^GFGGK_(biot)_ (**28s**)

From TentaGel S RAM resin (500 mg, 0.26 mmol g^–1^), **29s** was obtained as foamy white solid after preparative RP-HPLC (10.5 mg, 6.3 μmol, 4%). Analytical RP-HPLC: *t*_R_ = 3.20 min (*A*/*D* 100 : 0 to 0 : 100 in 7.5 min, *λ* = 214 nm). MS (ESI+): C_77_H_111_N_23_O_16_S_2_ calc./obs. 839.91/840.50 [M + 2H]^2+^.

#### E^2^*E*^1^Ef*z*^2^FK^1^G (**29c**)

From TentaGel S RAM resin (500 mg, 0.26 mmol g^–1^), **28c** was obtained as glassy white solid after preparative RP-HPLC (1.2 mg, 1.2 μmol, 1%). Analytical RP-HPLC: *t*_R_ = 3.16 min (*A*/*D* 100 : 0 to 0 : 100 in 7.5 min, *λ* = 214 nm). MS (ESI+): C_46_H_60_N_10_O_14_S calc./obs. 1009.41/1009.41 [M + H]^+^.

#### E^2^*E*^1^Ef*z*^2^FK^1^GBGGK_(biot)_ (**29x**)

From TentaGel S RAM resin (500 mg, 0.26 mmol g^–1^), **28x** was obtained as glassy white solid after preparative RP-HPLC (3.0 mg, 1.7 μmol, 1%). Analytical RP-HPLC: *t*_R_ = 3.94 min (*A*/*D* 100 : 0 to 0 : 100 in 7.5 min, *λ* = 214 nm). MS (ESI+): C_82_H_105_N_17_O_21_S_2_ calc./obs. 864.86/864.86 [M + 2H]^2+^.

#### E^2^*E*^1^Ef*z*^2^FK^1^GFGGK_(biot)_ (**29s**)

From TentaGel S RAM resin (500 mg, 0.26 mmol g^–1^), **28s** was obtained as glassy white solid after preparative RP-HPLC (8.4 mg, 5.2 μmol, 4%). Analytical RP-HPLC: *t*_R_ = 3.59 min (*A*/*D* 100 : 0 to 0 : 100 in 7.5 min, *λ* = 214 nm). MS (ESI+): C_75_H_101_N_17_O_20_S_2_ calc./obs. 1624.69/1624.27 [M + H]^+^.

#### _Me_l^1^G*k*^2^fP*E*^1^a^2^ (**30**)

From CT resin (200 mg, 1.63 mmol g^–1^), **30** was obtained as yellow solid after preparative RP-HPLC (2.2 mg, 2.9 μmol, 1%). Analytical RP-HPLC: *t*_R_ = 3.610 min (*A*/*D* 100 : 0 to 0 : 100 in 7.5 min, *λ* = 214 nm). MS (ESI+): C_37_H_54_N_8_O_8_ calc./obs. 739.41/739.41 [M + H]^+^.

#### l^1^_Me_G*k*^*2*^fP*E*^*1*^a^2^ (**31**)

From CT resin (200 mg, 1.63 mmol g^–1^), **31** was obtained as yellow solid after preparative RP-HPLC (6.2 mg, 8.4 μmol, 3%). Analytical RP-HPLC: *t*_R_ = 3.595 min (*A*/*D* 100 : 0 to 0 : 100 in 7.5 min, *λ* = 214 nm). MS (ESI+): C_37_H_54_N_8_O_8_ calc./obs. 739.41/739.41 [M + H]^+^.

#### l^1^G*k*^2^_Me_fP*E*^1^a^2^ (**32**)

From CT resin (200 mg, 1.63 mmol g^–1^), **32** was obtained as yellow solid after preparative RP-HPLC (4.3 mg, 5.8 μmol, 2%). Analytical RP-HPLC: *t*_R_ = 3.587 min (*A*/*D* 100 : 0 to 0 : 100 in 7.5 min, *λ* = 214 nm). MS (ESI+): C_37_H_54_N_8_O_9_ calc./obs. 739.41/739.41 [M + H]^+^.

#### _Me_l^1^_Me_G*k*^2^fP*E*^1^a^2^ (**33**)

From CT resin (200 mg, 1.63 mmol g^–1^), **33** was obtained as yellow solid after preparative RP-HPLC (11.5 mg, 15.3 μmol, 5%). Analytical RP-HPLC: *t*_R_ = 3.880 min (*A*/*D* 100 : 0 to 0 : 100 in 7.5 min, *λ* = 214 nm). MS (ESI+): C_38_H_56_N_8_O_8_ cal./obs. 753.43/753.43 [M + H]^+^.

#### _Me_l^1^G*k*^2^_Me_fP*E*^1^a^2^ (**34**)

From CT resin (200 mg, 1.63 mmol g^–1^), **34** was obtained as yellow solid after preparative RP-HPLC (11 mg, 14.6 μmol, 4.5%). Analytical RP-HPLC: *t*_R_ = 3.765 min (*A*/*D* 100 : 0 to 0 : 100 in 7.5 min, *λ* = 214 nm). MS (ESI+): C_38_H_56_N_8_O_8_ calc./obs. 753.43/753.43 [M + H]^+^.

#### l^1^_Me_G*k*^2^_Me_fP*E*^1^a^2^ (**35**)

From CT resin (200 mg, 1.63 mmol g^–1^), **35** was obtained as yellow solid after preparative RP-HPLC (13.2 mg, 17.5 μmol, 5.3%). Analytical RP-HPLC: *t*_R_ = 3.80 min (*A*/*D* 100 : 0 to 0 : 100 in 7.5 min, *λ* = 214 nm). MS (ESI+): C_38_H_56_N_8_O_8_ calc./obs. 775.41/775.41 [M + Na]^+^.

### X-ray crystallography

All measurements were performed on an Oxford Diffraction SuperNova area-detector diffractometer (Oxford Diffraction Ltd., Yarnton, Oxfordshire, UK) using mirror optics monochromated Mo *K*α radiation and Al filtered.^38^ The unit cell constants and an orientation matrix for data collection were obtained from a least-squares refinement of the setting angles of reflections. Data reduction was performed using CrysAlisPro (version 1.171.34.44). The intensities were corrected for Lorentz and polarization effects, and an absorption correction based on the multi-scan method using SCALE3 ABSPACK in CrysAlisPro was applied. Data collection and refinement parameters are given for each crystal structure in the ESI.† The structures were solved by direct methods using SHELXS-97, which revealed the positions of all non-hydrogen atoms, which were refined anisotropically. Refinement of the structure was carried out on *F*^2^ using full-matrix least-squares procedures, which minimized the function Σ*w*(*F*_o_^2^ – *F*_c_^2^)^2^. The weighting scheme was based on counting statistics and included a factor to downweight the intense reflections. All calculations were performed using SHELXL-97.†

### NMR studies

NMR data were acquired at a temperature of 298 K using a Bruker AvanceII 500 MHz NMR spectrometer equipped with an inverse dual channel (^1^H, X) *z*-gradient probehead (BBI), or on a Bruker AvanceII 400 MHz NMR spectrometer equipped with an inverse dual channel (^1^H, X) z-gradient probehead (BBI). Proton resonances were assigned using TOCSY and HSQC data. All NMR data were processed using TopSpin (version 3.0). 1D ^1^H-NMR data were acquired with 16 to 64 transients into 32k data points over a ppm width of 12 ppm using a W5 sequence to eliminate the water resonance.^39^ A relaxation delay of 6 s was applied between transients. 2D ^1^H-TOCSY and ROESY NMR data were acquired over a frequency width of 12 ppm in both *F*_2_ and *F*_1_ into 2k complex data points in *F*_2_ using 128 to 256 *t*_1_ increments depending on the sample. A relaxation delay of 2 s between transients was used for all experiments. ^1^H-TOCSY data were recorded using 8 or 32 transients, ^1^H-ROESY using 32 to 96 transients, depending on the sample. Water suppression was achieved using a WATERGATE routine after the final read pulse.^40^ The 2D TOCSY NMR data were acquired with a spin-lock time of 70 ms. 2D ROESY NMR (Tr-ROESY scheme)^41^ data were acquired with a mixing time of 150 ms. Data were processed using standard apodizing functions prior to Fourier transformation. 2D ^1^H–^13^C HSQC NMR data were acquired, with ^13^C decoupling during the acquisition period, over an *F*_2_ frequency width of 12 ppm into 2k complex data points. 16 to 32 transients were accumulated for each of 128 *t*_1_ increments over an *F*_1_ frequency width of 180 ppm centered at 90 ppm. Phase-sensitive data were acquired in a sensitivity-improved manner using an echo-antiecho acquisition mode. 2D ^1^H–^13^C HMBC NMR data were acquired over an *F*_2_ frequency width of 12 ppm into 2k complex data points. 64 to 96 transients were accumulated for each of 128 *t*_1_ increments over an *F*_1_ frequency width of 200 ppm centered at 100 ppm. Phase-sensitive data were acquired in a sensitivity-improved manner using an echo-antiecho acquisition mode.

### Molecular dynamics

BBP starting structures were experimental X-ray structure or their closest diastereoisomers built using Maestro (version 8.5). The compounds **5**, **8**, **9**, **14** and **18** were built with the peptide building dictionary interface in Maestro. Parameters for bridgehead lysine and glutamic acid were obtained from natural amino acid building blocks based on transferability of the AMBER force field ff99SB. The final topologies, coordinates and parameters for refinement were then built using the Xleap module from AMBER12 package. All minimization and MD calculations were performed by the SANDER module of AMBER 12.0. There were two parts of MD simulations. The first one was a restraint MD simulated annealing (rMDSA) performed with generalized-Born implicit solvent model (GB).^42^ A cutoff of 8 Å was used for the non-bonded interactions. The simulation started from a 500-step steepest descent energy minimization, which followed by a period of 1 ns simulated annealing using NOE distance restraints (Table S21†). The protocol involved: 5 ps of heating from 0 K to 600 K, 0.99 ns of slow cooling from 600 K to 100 K, and the last 10 ps of cooling to 0 K. The second MD simulation was an off-restraint simulation starting with the output structure of the first rMDSA above as an input with solvent corresponding to the NMR experiment: parameterized DMSO^43^ for **1**, **4**, **10**, **12**, **13**, and TIP3P^44^ water for all other BBPs. The simulations used Langevin dynamics for temperature regulation.^45^ Hydrogen bonds were constrained with the SHAKE constraint algorithm,^46^ and the long-range electrostatic interactions were handled by the particle-mesh Ewald (PME) procedure.^47^ In addition, the non-bonded interactions used a cutoff of 8 Å. In the second simulation, two stages of minimization approaches were carried out. The first stage involved only ions and solvent molecular with 500 steps of steepest descent minimization and 500 steps with conjugate gradient algorithm. The second stage minimized the entire system with 2500 steps. Next approach was 20 ps MD with weak restraints on the peptide. During this approach, the system was heated from 0 K to 300 K at constant volume. Finally, 1 ns MD on the entire solute and solvent was performed using constant pressure periodic boundary and 300 K constant temperature.

### Circular dichroism (CD)

Experiments were measured on a Jasco J-715 Spectropolarimeter using Hellma Suprasil R 110-QS 0.1 cm cuvettes. For each peptide, the measurements were performed in phosphate buffer (PB, pH = 7.0, 10 mm), 25% 2,2,2-trifluoroethanol (TFE) in PB (pH = 7.0, 10 mm), or in guanidinium chloride 6 m. The concentration of the peptides was 0.25 mg mL^–1^ and each sample was measured *via* 3 accumulations. The scan rate was 10 nm min^–1^, pitch 0.5 nm, response 16 s and bandwidth 1.0 nm. The nitrogen flow was kept >5 L min^–1^ throughout all measurements. Before each measurement, the cuvettes were washed successively with 1 m HCl, milli-Q H_2_O and PB. The baseline was recorded under the same conditions and subtracted automatically.

### Capture compound mass spectrometry

Capture experiments were carried out in PCR strips according to the general CCMS procedure.^48^ 400 μg of HeLa cell lysate at 10 μg μL^–1^ was pipetted for each set in triplicate. To the competition samples, 240 μM of BBP-competitor was added as a final concentration and incubated for 30 min at 4 °C. Assay and competition samples were then incubated with BBP-capture compounds or BBP-scaffold controls at a final concentration of 5 μM for 1 hour at 4 °C on a rotating axis and irradiated for 10 min at 310 nm in a caproBox™ (caprotec bioanalytics GmbH) to promote covalent cross-linking. Streptavidin-coated magnetic beads (Invitrogen) were added to the samples and further rotated for 1 hour at 4 °C. The beads were collected using a caproMag (caprotec bioanalytics GmbH) and washed 4 times with washing buffer (caprotec bionanalytics GmbH) followed by 3 times washing with 80% acetonitrile and lastly resuspended in MS-grade water. For tryptic digestion, the magnetic beads were incubated with trypsin in 50 mM ammonium bicarbonate for 16 hours at 37 °C. The supernatant was recovered, evaporated and stored at –20 °C until mass spectrometry analysis.

Mass Spectrometry: Tryptic digests were analyzed by online nanoflow liquid chromatography tandem MS (LC-MSn) on an UltiMate 3000 RSLCnano System (Dionex, part of Thermo Fisher Scientific, Germany) coupled to a LTQ-Orbitrap Velos instrument (Thermo Fisher Scientific, Germany) through a Proxeon nanoelectrospray ion source (Proxeon, part of Thermo Fisher Scientific, Germany). For chromatographic separation samples were first loaded on a reversed phase (RP) precolumn (Acclaim PepMap100, 5 μm, 100 Å, 100 μm i.d. × 20 mm) and separated on a RP analytical column (Acclaim PepMap RSLC C18, 2 μm, 100 Å, 75 μm i.d. × 150 mm, Dionex, part of Thermo Fisher Scientific, Germany) performing a 96 min gradient (5–45 % acetonitrile, 0.1% formic acid). MS detection was performed in the data-dependent mode allowing to automatically switch between Orbitrap-MS and LTQ-MS/MS acquisition in a top 20 configuration at 60 K resolution for a full scan with subsequent collision induced dissociation (CID) fragmentation. Full scan MS spectra (from *m*/*z* 300–2000) were acquired in the Orbitrap analyzer after accumulation to a target value of 1 × 10^6^ in the linear ion trap. The most intense ions (up to twenty, depending on signal intensity) with charge state ≥2 were sequentially isolated at a target value of 5000 and fragmented in the linear ion trap using low energy CID with normalized collision energy of 35%. Target ions already mass selected for CID were dynamically excluded for the duration of 60 s. The minimal signal required for MS2 was 1000 counts. An activation *q* of 0.25 and an activation time of 10 ms were applied for MS2 acquisitions. All MS/MS data were analyzed using Andromeda implemented in MaxQuant.^33^ Automated database searching against the human UniProtKB/Swiss-Prot database was performed with 6 ppm precursor tolerance, 0.5 Da fragment ion tolerance, full trypsin specificity allowing for up to 2 missed cleavages and methionine oxidation as variable modification. The maximum false discovery rates were set to 0.01 both on protein and peptide level, the maximum PEP to 1, and 7 amino acids were required as minimum peptide length. The label free quantification option was selected with a maximal retention time window of 2 min for the alignment between LC-MS/MS runs.

### MicroScale thermophoresis

Human calmodulin (Enzo Life Sciences, Lausen, Switzerland) was labeled using the RED-NHS Labeling kit (NanoTemper Technologies). The labeling reaction was performed according to the manufacturer's instructions in the supplied labeling buffer applying a concentration of 20 μM protein (molar dye : protein ratio ≈ 2 : 1) at RT for 30 min. Unreacted dye was removed with the supplied dye removal columns equilibrated with MST buffer (50 mm TrisHCl pH 7.5, 150 mm NaCl, 10 mm MgCl_2_). The label : protein ratio was determined using photometry at 650 and 280 nm. Thereby, a ratio of 0.8 was typically achieved. The labeled calmodulin was adjusted to 20 nM with MST buffer. **27c** was dissolved in MST buffer and a series of 16 1 : 1 dilutions was prepared in the identical buffer, producing ligand concentrations ranging from 30.5 nM to 500 μM.

For thermophoresis, each ligand dilution was mixed with one volume of labeled calmodulin, which leads to a final concentration of fluorescently labeled calmodulin of 10 nM and final ligand concentrations ranging from 15.3 nM to 250 μM. After 10 min incubation, followed by centrifugation at 10 000 × *g* for 10 min, approximately 4 μL of each solution was filled into Monolith NT Standard Treated Capillaries (NanoTemper Technologies GmbH, Germany). Thermophoresis was measured using a Monolith NT.115 instrument (NanoTemper Technologies GmbH) at an ambient temperature of 25 °C with 5 s/30 s/5 s laser off/on/off times, respectively. Instrument parameters were adjusted with 15% LED power and 40% MST power. Data of three independently pipetted measurements were analyzed (NT.Analysis software version 1.5.41, NanoTemper Technologies) using the signal from Thermophoresis + T-Jump. **28c** was used as negative control.

### Isothermal titration microcalorimetry

ITC experiments were performed using a MicroCal ITC200 from GE Healthcare. **27c** and **28c** were dissolved in water to a concentration of 0.7 and 0.6 mM, respectively. Calmodulin was dissolved in water at 50 μM. Optionally CaCl_2_ (final concentration 500 μM) or EDTA (final concentration 1 mM) were added. The peptide solution was injected into the calmodulin solution (2.5 μL of peptide per injection at an interval of 180 s, a total of 15 injections into the cell volume of 200 μL, with stirring speed of 800 rpm, at 25 °C). The calmodulin solutions had a pH of 6.5 before and after titration. The experimental data were fitted to a theoretical titration curve (“one set of sites” model) using software supplied by Microcal, with *n* (number of binding sites per monomer), Δ*H* (binding enthalpy, kcal mol^–1^) and *Ka* (association constant, M^–1^), as adjustable parameters. Thermodynamic parameters were calculated from the Gibbs free energy equation, Δ*G* = Δ*H* – *T*Δ*S* = –*RT* ln *K*_*a*_, where Δ*G*, Δ*H*, and Δ*S* are the changes in free energy, enthalpy, and entropy of binding, respectively. *T* is the absolute temperature, and *R* = 1.98 cal mol^–1^ K^–1^.

### Serum stability assays

Human serum was diluted 1 : 4 in DMEM. Selected peptide was diluted in TRIS buffer to a concentration of 400 μM. Aliquots of peptide solution (50 μL) were added to aliquots of serum (50 μL) in sterile Eppendorf tubes, to reach a peptide concentration of 200 μM during the assay. Samples were incubated at 37 celsius degree under gentle stirring (350 rpm). Different samples (triplicate) were quenched at different time points (0/1/3/6/24 h) by precipitating serum proteins through the addition of ZnSO_4_·7H_2_O (0.1 M, 100 μL) and cooling down in ice bath. Protein precipitates were pelleted under centrifugation and the supernatants were sampled and evaporated to dryness in a centrifugal evaporator. Samples were resuspended in a H_2_O/ACN (4 : 1) mixture and centrifuged again to remove residual protein precipitate. Supernatants were then sampled and analysed by LC-MS. Experiment controls included a precipitation control for each peptide, to test their resistance to the protein precipitation conditions, and serum blanks, to check reproducibility over different serum batches. Two peaks originating from DMEM, surviving the incubation in serum and the protein precipitation conditions were used as internal standard.

### PAMPA assays

The assays were performed according to the manufacturer's protocol (Millipore, Billerica, MA, USA). Two (acceptor and donor) multi-well plates were used. To the acceptor wells, 300 μL buffer (5% DMSO in PBS, pH 7.4) was added. Donor plate consists of PVDF filter onto which 5 μL of lecithine in 5% dodecane was applied as an artificial membrane then immediately 150 μL sample in buffer (0.2 mM) was added. Both plates were incubated for 16 h at room temperature and the solution from acceptor plate was analyzed by LCMS. Equilibrium solutions are the mixture of volumes (*i.e.*, 150 μL sample in buffer and 300 μL buffer mixed manually) added to the donor and acceptor wells. Later, these were analyzed by LCMS. Both compounds and equilibrium solution in triplicates were analyzed by LCMS. Carbamazepine is used as control and analyzed parallel with compounds. Permeability of compounds is calculated using the following formula. Calibration of Carbamazepine resulted in a linear curve and permeability value showed a good correlation with theoretical permeability value (Millipore) validating the assay. We did not observe any norbornapeptide or *N*-methylated norbornapeptides transmission into the acceptor plate.

## Supplementary Material

Supplementary information

Crystal structure data
