# Supplementary material for: Bridged bicyclic peptides as potential drug scaffolds: synthesis, structure, protein binding and stability
Source: Chem Sci. 2015 Jul 13;6(10):5473–90. doi: 10.1039/c5sc01699a (PMC5949603; doi:10.1039/c5sc01699a)

Electronic Supplementary Material (ESI) for Chemical Science.
This journal is © The Royal Society of Chemistry 2015

Supporting information

Crystallographic data

Table S1. Crystal data and structure refinement for **10** (CCDC 1063797)

Empirical formula	C36 H52 N8 O9	
Formula weight	740.86	
Temperature	123(2) K	
Wavelength	0.71073 Å	
Crystal system	Monoclinic	
Space group	I 1 2 1	
Unit cell dimensions	a = 16.9971(11) Å	α= 90°.
	b = 9.2681(4) Å	β= 99.408(6)°.
	c = 25.8496(17) Å	γ = 90°.
Volume	4017.3(4) $Å^3$	
Z	4	
Density (calculated)	1.225 Mg/m^3	
Absorption coefficient	0.089 mm^{-1}	
F(000)	1584	
Crystal size	0.2789 x 0.1267 x 0.0162 mm^3	
Theta range for data collection	1.56 to 26.56°.	
Index ranges	-20<=h<=21, -11<=k<=11, -31<=l<=30	
Reflections collected	12542	
Independent reflections	6184 [R(int) = 0.0843]	
Completeness to theta = 25.00°	89.3 %	
Absorption correction	Semi-empirical from equivalents	
Max. and min. transmission	1.00000 and 0.77965	
Refinement method	Full-matrix least-squares on F^2	
Data / restraints / parameters	6184 / 5 / 469	
Goodness-of-fit on F^2	0.981	
Final R indices [I>2sigma(I)]	R1 = 0.0892, wR2 = 0.2133	
R indices (all data)	R1 = 0.1457, wR2 = 0.2361	
Absolute structure parameter	1(3)	
Largest diff. peak and hole	0.598 and -0.330 e.$Å^{-3}$	

Table S2. Atomic coordinates (x 10^4) and equivalent isotropic displacement parameters ($Å^2$x 10^3) for **10**. U(eq) is defined as one third of the trace of the orthogonalized U^{ij} tensor.

	x	y	z	U(eq)
C(1)	6792(8)	5714(16)	9998(4)	93(5)
C(2)	6287(7)	6160(15)	9480(3)	83(4)
C(3)	6771(5)	6187(8)	9033(3)	31(2)
C(4)	6393(4)	6951(8)	8536(3)	23(2)
C(5)	6857(4)	6678(9)	8081(3)	26(2)
C(6)	6860(4)	5541(9)	7245(3)	28(2)
C(7)	6488(4)	6474(7)	6778(3)	22(2)
C(8)	5540(5)	8346(8)	6429(3)	27(2)
C(9)	5994(5)	9631(8)	6271(3)	24(2)
C(10)	5547(4)	10316(8)	5757(3)	26(2)
C(11)	5837(5)	11812(8)	5645(3)	30(2)
C(12)	5834(5)	12914(10)	6087(3)	36(2)
C(13)	4603(5)	14010(8)	6308(3)	24(2)
C(14)	3891(5)	13728(9)	6579(3)	29(2)
C(15)	3260(5)	12941(10)	6192(3)	33(2)

C(16)	4645(5)	13318(8)	7441(3)	24(2)
C(17)	4897(4)	12294(8)	7904(3)	24(2)
C(18)	5790(5)	12009(9)	7926(3)	33(2)
C(19)	6155(5)	10996(8)	8386(3)	29(2)
C(20)	6041(5)	9432(9)	8241(3)	29(2)
C(21)	3790(5)	10788(10)	8075(3)	32(2)
C(22)	3901(5)	8302(9)	8479(3)	33(2)
C(23)	3802(6)	6760(9)	8245(3)	38(2)
C(24)	3919(5)	7033(8)	7690(3)	34(2)
C(25)	3294(4)	9018(8)	7086(3)	21(2)
C(26)	3343(4)	8041(8)	6605(3)	20(2)
C(27)	4737(5)	8777(8)	6583(3)	20(2)
C(28)	2880(5)	8707(8)	6105(3)	25(2)
C(29)	2848(5)	7677(8)	5647(3)	27(2)
C(30)	3327(5)	7840(9)	5276(3)	30(2)
C(31)	3280(5)	6960(9)	4837(4)	39(2)
C(32)	2762(5)	5796(8)	4801(3)	26(2)
C(33)	2303(5)	5590(9)	5171(3)	32(2)
C(34)	2339(5)	6504(8)	5595(3)	31(2)
C(35)	5544(11)	5120(20)	9360(8)	166(8)
C(36)	3467(5)	9223(7)	8040(3)	27(2)
N(2)	6365(4)	8503(6)	8644(2)	25(2)
N(3)	5092(4)	12887(7)	6310(2)	31(2)
N(4)	6529(4)	5774(7)	7718(2)	26(2)
N(5)	4464(3)	10977(6)	7862(2)	21(1)
N(7)	4174(3)	7777(7)	6551(2)	21(1)
N(11)	5982(3)	7499(7)	6858(2)	27(2)
N(22)	4080(4)	12863(7)	7057(2)	29(2)
N(102)	3592(4)	8462(6)	7561(2)	21(1)
O(1)	5727(3)	8982(6)	7821(2)	29(1)
O(2)	4974(3)	14529(6)	7436(2)	34(1)
O(3)	4633(3)	10047(6)	6737(2)	30(1)
O(4)	6678(3)	6256(6)	6345(2)	33(1)
O(5)	2684(3)	4873(6)	4377(2)	35(1)
O(6)	2968(3)	10201(6)	7039(2)	28(1)
O(7)	4697(3)	15171(6)	6115(2)	33(1)
O(55)	3523(4)	11713(7)	8333(3)	55(2)
O(101)	7506(3)	7306(6)	8083(2)	37(2)

Table S3. Bond lengths [Å] and angles [°] for **10**

Bond lengths [Å]	
C(1)-C(2)	1.524(12)
C(2)-C(3)	1.524(11)
C(2)-C(35)	1.580(15)
C(3)-C(4)	1.514(9)
C(4)-N(2)	1.467(9)
C(4)-C(5)	1.540(11)
C(5)-O(101)	1.247(9)
C(5)-N(4)	1.313(9)
C(6)-N(4)	1.445(9)
C(6)-C(7)	1.534(11)
C(7)-O(4)	1.233(9)
C(7)-N(11)	1.321(9)
C(8)-N(11)	1.462(9)
C(8)-C(9)	1.511(10)
C(8)-C(27)	1.536(11)
C(9)-C(10)	1.553(10)
C(10)-C(11)	1.516(11)
C(11)-C(12)	1.533(11)
C(12)-N(3)	1.472(9)
C(13)-O(7)	1.208(8)
C(13)-N(3)	1.332(10)
C(13)-C(14)	1.516(11)
C(14)-N(22)	1.463(10)
C(14)-C(15)	1.526(11)
C(16)-O(2)	1.254(8)
C(16)-N(22)	1.333(10)
C(16)-C(17)	1.532(11)
C(17)-N(5)	1.420(9)
C(17)-C(18)	1.532(11)
C(18)-C(19)	1.562(11)
C(19)-C(20)	1.502(11)
C(20)-O(1)	1.204(9)
C(20)-N(2)	1.393(10)
C(21)-O(55)	1.218(10)

C(21)-N(5)	1.361(9)
C(21)-C(36)	1.548(11)
C(22)-C(36)	1.513(11)
C(22)-C(23)	1.551(12)
C(23)-C(24)	1.503(12)
C(24)-N(102)	1.454(9)
C(25)-O(6)	1.226(8)
C(25)-N(102)	1.350(9)
C(25)-C(26)	1.552(10)
C(26)-N(7)	1.462(9)
C(26)-C(28)	1.529(10)
C(27)-O(3)	1.265(9)
C(27)-N(7)	1.325(9)
C(28)-C(29)	1.515(11)
C(29)-C(30)	1.365(10)
C(29)-C(34)	1.382(11)
C(30)-C(31)	1.390(11)
C(31)-C(32)	1.386(11)
C(32)-C(33)	1.342(10)
C(32)-O(5)	1.381(9)
C(33)-C(34)	1.379(11)
C(36)-N(102)	1.471(9)

Angles [°]

C(3)-C(2)-C(1)	111.9(9)
C(3)-C(2)-C(35)	111.9(11)
C(1)-C(2)-C(35)	109.0(12)
C(4)-C(3)-C(2)	116.4(7)
N(2)-C(4)-C(3)	108.7(6)
N(2)-C(4)-C(5)	110.0(6)
C(3)-C(4)-C(5)	111.8(6)
O(101)-C(5)-N(4)	124.9(7)
O(101)-C(5)-C(4)	118.9(7)
N(4)-C(5)-C(4)	116.2(6)
N(4)-C(6)-C(7)	114.4(6)
O(4)-C(7)-N(11)	122.3(7)
O(4)-C(7)-C(6)	119.1(6)
N(11)-C(7)-C(6)	118.6(7)
N(11)-C(8)-C(9)	114.0(7)
N(11)-C(8)-C(27)	108.1(6)

C(9)-C(8)-C(27)	112.3(6)
C(8)-C(9)-C(10)	110.7(6)
C(11)-C(10)-C(9)	114.1(6)
C(10)-C(11)-C(12)	115.2(7)
N(3)-C(12)-C(11)	112.9(7)
O(7)-C(13)-N(3)	125.0(7)
O(7)-C(13)-C(14)	120.9(7)
N(3)-C(13)-C(14)	114.0(7)
N(22)-C(14)-C(13)	113.8(6)
N(22)-C(14)-C(15)	109.0(7)
C(13)-C(14)-C(15)	107.9(7)
O(2)-C(16)-N(22)	122.9(7)
O(2)-C(16)-C(17)	119.6(7)
N(22)-C(16)-C(17)	117.5(7)
N(5)-C(17)-C(18)	110.6(6)
N(5)-C(17)-C(16)	113.4(6)
C(18)-C(17)-C(16)	106.6(6)
C(17)-C(18)-C(19)	113.6(7)
C(20)-C(19)-C(18)	111.8(7)
O(1)-C(20)-N(2)	121.5(7)
O(1)-C(20)-C(19)	125.4(7)
N(2)-C(20)-C(19)	113.0(7)
O(55)-C(21)-N(5)	122.9(7)
O(55)-C(21)-C(36)	122.2(7)
N(5)-C(21)-C(36)	114.2(7)
C(36)-C(22)-C(23)	102.7(6)
C(24)-C(23)-C(22)	101.2(6)
N(102)-C(24)-C(23)	105.5(7)
O(6)-C(25)-N(102)	121.9(7)
O(6)-C(25)-C(26)	121.6(7)
N(102)-C(25)-C(26)	116.4(6)
N(7)-C(26)-C(28)	111.0(6)
N(7)-C(26)-C(25)	110.7(6)
C(28)-C(26)-C(25)	110.8(6)
O(3)-C(27)-N(7)	122.2(7)
O(3)-C(27)-C(8)	120.0(6)
N(7)-C(27)-C(8)	117.8(6)
C(29)-C(28)-C(26)	110.8(6)
C(30)-C(29)-C(34)	117.1(7)

C(30)-C(29)-C(28)	122.0(7)
C(34)-C(29)-C(28)	120.9(7)
C(29)-C(30)-C(31)	123.2(8)
C(32)-C(31)-C(30)	117.5(8)
C(33)-C(32)-O(5)	119.1(7)
C(33)-C(32)-C(31)	120.1(7)
O(5)-C(32)-C(31)	120.8(7)
C(32)-C(33)-C(34)	121.6(7)
C(33)-C(34)-C(29)	120.4(7)
N(102)-C(36)-C(22)	104.0(6)
N(102)-C(36)-C(21)	113.6(6)
C(22)-C(36)-C(21)	111.1(7)
C(20)-N(2)-C(4)	119.1(6)
C(13)-N(3)-C(12)	124.0(7)
C(5)-N(4)-C(6)	121.5(6)
C(21)-N(5)-C(17)	122.7(7)
C(27)-N(7)-C(26)	125.2(6)
C(7)-N(11)-C(8)	122.3(6)
C(16)-N(22)-C(14)	119.2(7)
C(25)-N(102)-C(24)	129.0(6)
C(25)-N(102)-C(36)	119.8(6)
C(24)-N(102)-C(36)	110.3(6)

Table S4. Anisotropic displacement parameters ($Å^2 x 10^3$) for **10**. The anisotropic displacement factor exponent takes the form: $-2\pi^2[h^2a^{*2}U^{11} + ... + 2 h k a^* b^* U^{12}]$

	U^{11}	U^{22}	U^{33}	U^{23}	U^{13}	U^{12}
C(1)	145(12)	105(11)	21(6)	15(6)	-6(7)	3(10)
C(2)	121(11)	109(10)	20(5)	36(6)	18(6)	54(9)
C(3)	37(5)	14(4)	38(5)	7(4)	-1(4)	1(4)
C(4)	21(4)	18(4)	25(4)	-1(4)	-9(3)	-6(3)
C(5)	18(4)	29(5)	30(5)	-10(4)	-2(3)	-8(4)
C(6)	26(4)	28(5)	28(5)	4(4)	-2(4)	-7(4)
C(7)	16(4)	13(4)	40(5)	3(4)	13(4)	4(3)
C(8)	39(5)	11(4)	29(5)	6(4)	0(4)	6(4)
C(9)	31(4)	13(4)	29(4)	-5(4)	5(4)	-3(3)
C(10)	27(4)	22(4)	26(4)	1(4)	0(4)	4(4)
C(11)	21(4)	31(5)	38(5)	9(4)	7(4)	-1(4)

C(12)	36(5)	37(5)	36(5)	5(4)	8(4)	5(4)
C(13)	23(4)	11(4)	38(5)	6(4)	4(4)	-3(3)
C(14)	29(5)	25(5)	29(5)	6(4)	-4(4)	0(4)
C(15)	42(5)	37(5)	19(4)	5(4)	1(4)	7(4)
C(16)	25(4)	20(4)	24(4)	-4(4)	-6(4)	-7(3)
C(17)	29(4)	9(4)	31(5)	-5(3)	-6(4)	3(3)
C(18)	40(5)	20(4)	38(5)	-5(4)	3(4)	-10(4)
C(19)	26(4)	30(5)	26(5)	-7(4)	-8(4)	12(4)
C(20)	33(5)	29(5)	23(5)	5(4)	1(4)	7(4)
C(21)	26(5)	49(6)	21(4)	-7(4)	2(4)	-17(4)
C(22)	35(5)	41(6)	21(4)	1(4)	-7(4)	-16(4)
C(23)	44(5)	33(5)	34(5)	20(4)	2(4)	-1(4)
C(24)	47(5)	9(4)	42(5)	8(4)	-7(4)	9(4)
C(25)	19(4)	18(4)	25(4)	-2(4)	1(3)	4(3)
C(27)	27(4)	11(4)	22(4)	-3(3)	0(3)	-9(3)
C(28)	30(5)	14(4)	28(5)	1(3)	-7(4)	6(4)
C(29)	36(5)	16(4)	29(5)	5(4)	8(4)	6(4)
C(30)	41(5)	22(4)	27(4)	-13(4)	8(4)	-10(4)
C(31)	39(5)	39(5)	39(5)	-5(5)	10(4)	0(5)
C(32)	35(5)	27(4)	15(4)	-9(4)	-5(4)	-2(4)
C(33)	34(5)	27(5)	36(5)	-6(4)	9(4)	-18(4)
C(34)	44(5)	28(5)	21(4)	6(4)	7(4)	-17(4)
C(36)	40(5)	12(4)	29(5)	-7(4)	11(4)	-6(4)
N(2)	32(4)	23(4)	20(3)	-3(3)	0(3)	-1(3)
N(3)	40(4)	17(3)	35(4)	6(3)	9(3)	-3(3)
N(4)	34(4)	17(3)	27(4)	-1(3)	7(3)	-3(3)
N(5)	22(3)	22(3)	20(3)	-9(3)	6(3)	-4(3)
N(7)	19(3)	22(3)	19(3)	4(3)	-2(3)	3(3)
N(11)	31(4)	35(4)	14(3)	12(3)	-1(3)	7(3)
N(22)	34(4)	20(3)	33(4)	17(3)	2(3)	10(3)
N(102)	22(3)	16(3)	24(4)	5(3)	1(3)	-8(3)
O(1)	29(3)	26(3)	28(3)	0(3)	-7(3)	2(2)
O(2)	23(3)	22(3)	52(4)	0(3)	-8(3)	0(3)
O(3)	26(3)	30(3)	36(3)	-8(3)	11(2)	-7(3)
O(4)	29(3)	31(3)	39(4)	6(3)	9(3)	5(3)
O(5)	41(3)	35(3)	30(3)	-11(3)	7(3)	-14(3)
O(6)	28(3)	19(3)	34(3)	-10(3)	2(2)	0(2)
O(7)	26(3)	21(3)	51(4)	6(3)	7(3)	2(3)
O(55)	59(4)	52(4)	64(5)	-37(4)	41(4)	-19(3)

O(101)	33(3)	32(3)	44(4)	7(3)	-1(3)	-4(3)

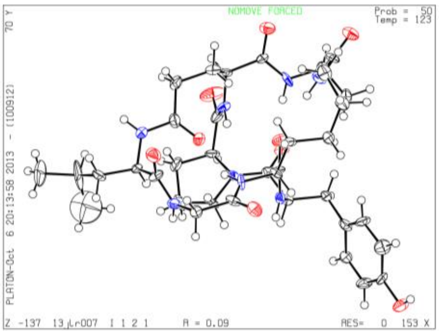

Table S5. Crystal data and structure refinement for **12** (CCDC 1063799)

Empirical formula	C39 H64 N8 O12	
Formula weight	836.98	
Temperature	173(2) K	
Wavelength	0.71069 Å	
Crystal system	Monoclinic	
Space group	P 21	
Unit cell dimensions	a = 9.769(5) Å	α= 90.000(5)°.
	b = 13.318(5) Å	β= 103.191(5)°.
	c = 17.543(5) Å	γ = 90.000(5)°.
Volume	2222.2(15) Å3	
Z	2	
Density (calculated)	1.251 Mg/m^3	
Absorption coefficient	0.093 mm^{-1}	
F(000)	900	
Crystal size	0.3124 x 0.1804 x 0.1192 mm^3	
Theta range for data collection	1.939 to 27.235°.	
Index ranges	-12<=h<=12, -16<=k<=16, -21<=l<=21	
Reflections collected	17813	
Independent reflections	8517 [R(int) = 0.0286]	
Completeness to theta = 25.000°	99.8 %	
Absorption correction	Gaussian	
Max. and min. transmission	1 and 0.786	
Refinement method	Full-matrix least-squares on F^2	
Data / restraints / parameters	8517 / 1 / 539	
Goodness-of-fit on F^2	1.039	
Final R indices [I>2sigma(I)]	R1 = 0.0571, wR2 = 0.1425	
R indices (all data)	R1 = 0.0658, wR2 = 0.1496	
Absolute structure parameter	-0.2(5)	
Extinction coefficient	n/a	
Largest diff. peak and hole	0.662 and -0.667 e.$Å^{-3}$	

Table S6. Atomic coordinates (x 10^4) and equivalent isotropic displacement parameters ($Å^2$x 10^3) for **12**. U(eq) is defined as one third of the trace of the orthogonalized U^{ij} tensor.

	x	y	z	U(eq)
C(1)	9374(5)	-520(3)	4279(2)	29(1)
C(2)	10437(5)	174(3)	4580(2)	28(1)
C(3)	10193(5)	1190(3)	4457(2)	27(1)

C(4)	8910(4)	1555(3)	4028(2)	24(1)
C(5)	7870(5)	858(3)	3734(3)	31(1)
C(6)	8077(5)	-171(4)	3861(3)	38(1)
C(7)	8625(4)	2658(3)	3901(2)	24(1)
C(8)	9771(4)	3249(3)	3631(2)	23(1)
C(9)	10179(4)	2767(3)	2919(2)	22(1)
C(10)	7821(4)	3165(3)	2006(2)	29(1)
C(11)	7481(5)	3144(3)	1115(3)	31(1)
C(12)	8342(5)	2269(4)	915(3)	33(1)
C(13)	9694(4)	2261(3)	1563(2)	25(1)
C(14)	10935(4)	2749(3)	1317(2)	25(1)
C(15)	12327(4)	4264(3)	1284(2)	25(1)
C(16)	13710(4)	4157(3)	1888(2)	26(1)
C(17)	14914(4)	3700(3)	3206(3)	29(1)
C(18)	14703(5)	2942(4)	3820(3)	43(1)
C(19)	15417(4)	4703(3)	3605(2)	29(1)
C(21)	14790(5)	6277(4)	4164(3)	36(1)
C(22)	13709(5)	7095(4)	3912(3)	35(1)
C(23)	12229(4)	6830(3)	4048(3)	29(1)
C(24)	11058(5)	6772(3)	3313(3)	28(1)
C(25)	9874(4)	6088(3)	3427(2)	24(1)
C(26)	10298(4)	4980(3)	3410(2)	23(1)
C(27)	7538(5)	6847(3)	2987(3)	28(1)
C(28)	6185(5)	6923(4)	2358(3)	36(1)
C(30)	6783(5)	6672(3)	1102(3)	33(1)
C(31)	6774(4)	5948(3)	436(2)	28(1)
C(32)	5703(5)	6256(4)	-305(3)	37(1)
C(33)	5544(7)	5487(5)	-949(4)	61(2)
C(34)	4739(7)	5923(7)	-1732(4)	73(2)
C(35)	9301(5)	5612(3)	828(2)	27(1)
C(36)	4789(13)	4576(7)	-758(6)	142(6)
C(45)	11985(5)	5392(3)	1192(3)	31(1)
C(48)	10682(5)	5645(4)	564(3)	43(1)
N(1)	9282(4)	2787(2)	2213(2)	24(1)
N(2)	11171(3)	3722(2)	1483(2)	22(1)
N(3)	13653(4)	3780(3)	2587(2)	25(1)
N(4)	8180(4)	5911(3)	280(2)	31(1)
N(5)	6139(4)	6340(3)	1658(2)	32(1)
N(6)	9358(4)	4298(2)	3520(2)	23(1)

N(7)	14436(4)	5359(3)	3692(2)	32(1)
N(12)	8555(4)	6248(2)	2851(2)	26(1)
O(1)	9669(4)	-1506(2)	4424(2)	41(1)
O(2)	11347(3)	2361(2)	3007(2)	28(1)
O(3)	11470(3)	4730(2)	3313(2)	33(1)
O(4)	11678(3)	2243(2)	986(2)	39(1)
O(5)	14821(3)	4448(2)	1739(2)	35(1)
O(6)	16683(3)	4850(3)	3857(2)	39(1)
O(7)	7347(4)	7502(3)	1144(2)	48(1)
O(8)	9204(3)	5336(2)	1479(2)	29(1)
O(9)	7623(3)	7333(2)	3598(2)	36(1)
C(1S)	13185(11)	31(8)	3431(6)	105(3)
C(2S)	9690(30)	9504(11)	1959(11)	307(19)
C(3S)	12330(20)	8198(11)	1338(17)	247(14)
O(1S)	12701(8)	565(4)	2727(4)	103(2)
O(2S)	9119(10)	9100(9)	1224(12)	292(10)
O(3S)	10850(40)	8298(13)	664(7)	510(30)

Table S7. Bond lengths [Å] and angles [°] for **12**.

Bond lengths [Å]	
C(1)-O(1)	1.356(5)
C(1)-C(6)	1.391(7)
C(1)-C(2)	1.400(6)
C(2)-C(3)	1.382(6)
C(3)-C(4)	1.394(6)
C(4)-C(5)	1.386(6)
C(4)-C(7)	1.503(6)
C(5)-C(6)	1.395(6)
C(7)-C(8)	1.529(6)
C(8)-N(6)	1.455(5)
C(8)-C(9)	1.535(5)
C(9)-O(2)	1.240(5)
C(9)-N(1)	1.343(5)
C(10)-N(1)	1.479(6)
C(10)-C(11)	1.522(6)
C(11)-C(12)	1.525(6)

C(12)-C(13)	1.534(6)
C(13)-N(1)	1.471(5)
C(13)-C(14)	1.523(6)
C(14)-O(4)	1.229(5)
C(14)-N(2)	1.336(5)
C(15)-N(2)	1.449(5)
C(15)-C(16)	1.522(6)
C(15)-C(45)	1.539(6)
C(16)-O(5)	1.235(5)
C(16)-N(3)	1.337(5)
C(17)-N(3)	1.449(5)
C(17)-C(18)	1.524(6)
C(17)-C(19)	1.535(6)
C(19)-O(6)	1.230(5)
C(19)-N(7)	1.332(6)
C(21)-N(7)	1.472(6)
C(21)-C(22)	1.511(7)
C(22)-C(23)	1.559(6)
C(23)-C(24)	1.518(6)
C(24)-C(25)	1.520(6)
C(25)-N(12)	1.460(5)
C(25)-C(26)	1.534(6)
C(26)-O(3)	1.241(5)
C(26)-N(6)	1.337(5)
C(27)-O(9)	1.239(5)
C(27)-N(12)	1.337(5)
C(27)-C(28)	1.520(6)
C(28)-N(5)	1.444(6)
C(30)-O(7)	1.230(6)
C(30)-N(5)	1.350(6)
C(30)-C(31)	1.515(7)
C(31)-N(4)	1.461(5)
C(31)-C(32)	1.526(6)
C(32)-C(33)	1.507(8)
C(33)-C(36)	1.498(12)
C(33)-C(34)	1.532(8)
C(35)-O(8)	1.225(5)
C(35)-N(4)	1.343(6)
C(35)-C(48)	1.523(6)

C(45)-C(48)	1.520(7)
C(1S)-O(1S)	1.411(11)
C(2S)-O(2S)	1.39(2)
C(3S)-O(3S)	1.65(3)
Angles [°]	
O(1)-C(1)-C(6)	123.4(4)
O(1)-C(1)-C(2)	117.5(4)
C(6)-C(1)-C(2)	119.1(4)
C(3)-C(2)-C(1)	120.1(4)
C(2)-C(3)-C(4)	121.8(4)
C(5)-C(4)-C(3)	117.4(4)
C(5)-C(4)-C(7)	120.3(4)
C(3)-C(4)-C(7)	122.3(4)
C(4)-C(5)-C(6)	122.1(4)
C(1)-C(6)-C(5)	119.6(4)
C(4)-C(7)-C(8)	115.1(3)
N(6)-C(8)-C(7)	109.6(3)
N(6)-C(8)-C(9)	114.1(3)
C(7)-C(8)-C(9)	112.5(3)
O(2)-C(9)-N(1)	120.8(4)
O(2)-C(9)-C(8)	118.9(4)
N(1)-C(9)-C(8)	120.4(3)
N(1)-C(10)-C(11)	102.6(3)
C(10)-C(11)-C(12)	104.3(4)
C(11)-C(12)-C(13)	105.2(3)
N(1)-C(13)-C(14)	113.1(3)
N(1)-C(13)-C(12)	103.3(3)
C(14)-C(13)-C(12)	113.2(3)
O(4)-C(14)-N(2)	122.9(4)
O(4)-C(14)-C(13)	119.6(4)
N(2)-C(14)-C(13)	117.5(3)
N(2)-C(15)-C(16)	113.7(3)
N(2)-C(15)-C(45)	110.4(3)
C(16)-C(15)-C(45)	107.6(3)
O(5)-C(16)-N(3)	122.3(4)
O(5)-C(16)-C(15)	120.4(4)
N(3)-C(16)-C(15)	117.3(4)
N(3)-C(17)-C(18)	110.5(4)
N(3)-C(17)-C(19)	114.0(3)

C(18)-C(17)-C(19)	109.6(4)
O(6)-C(19)-N(7)	123.1(4)
O(6)-C(19)-C(17)	119.5(4)
N(7)-C(19)-C(17)	117.3(4)
N(7)-C(21)-C(22)	112.1(4)
C(21)-C(22)-C(23)	113.7(4)
C(24)-C(23)-C(22)	115.3(4)
C(23)-C(24)-C(25)	111.6(4)
N(12)-C(25)-C(24)	113.6(3)
N(12)-C(25)-C(26)	109.1(3)
C(24)-C(25)-C(26)	110.9(3)
O(3)-C(26)-N(6)	121.5(4)
O(3)-C(26)-C(25)	121.5(4)
N(6)-C(26)-C(25)	117.0(3)
O(9)-C(27)-N(12)	124.1(4)
O(9)-C(27)-C(28)	118.0(4)
N(12)-C(27)-C(28)	117.9(4)
N(5)-C(28)-C(27)	115.8(4)
O(7)-C(30)-N(5)	121.9(5)
O(7)-C(30)-C(31)	122.8(4)
N(5)-C(30)-C(31)	115.3(4)
N(4)-C(31)-C(30)	109.0(4)
N(4)-C(31)-C(32)	110.0(4)
C(30)-C(31)-C(32)	111.6(4)
C(33)-C(32)-C(31)	112.9(4)
C(36)-C(33)-C(32)	110.8(7)
C(36)-C(33)-C(34)	109.2(6)
C(32)-C(33)-C(34)	111.1(5)
O(8)-C(35)-N(4)	122.2(4)
O(8)-C(35)-C(48)	123.7(4)
N(4)-C(35)-C(48)	114.1(4)
C(48)-C(45)-C(15)	114.7(4)
C(45)-C(48)-C(35)	115.2(4)
C(9)-N(1)-C(13)	118.0(3)
C(9)-N(1)-C(10)	128.5(3)
C(13)-N(1)-C(10)	113.0(3)
C(14)-N(2)-C(15)	122.4(3)
C(16)-N(3)-C(17)	120.6(3)
C(35)-N(4)-C(31)	121.6(4)

C(30)-N(5)-C(28)	120.5(4)
C(26)-N(6)-C(8)	119.6(3)
C(19)-N(7)-C(21)	121.7(4)
C(27)-N(12)-C(25)	122.5(4)

Table S8. Anisotropic displacement parameters (Å^{2}x 10^3) for **12**. The anisotropic displacement factor exponent takes the form: $-2\pi^2$[$h^2a^{*2}U^{11}$ + ... + 2 h k a* b* U^{12}]

	U^{11}	U^{22}	U^{33}	U^{23}	U^{13}	U^{12}
C(1)	37(3)	25(2)	24(2)	2(2)	4(2)	2(2)
C(2)	29(2)	30(2)	25(2)	7(2)	5(2)	4(2)
C(3)	27(2)	30(2)	24(2)	2(2)	4(2)	-3(2)
C(4)	25(2)	25(2)	22(2)	1(2)	8(2)	-1(2)
C(5)	25(2)	31(2)	34(2)	0(2)	-1(2)	2(2)
C(6)	35(3)	30(2)	42(3)	0(2)	-3(2)	-5(2)
C(7)	25(2)	25(2)	24(2)	3(2)	10(2)	1(2)
C(8)	23(2)	24(2)	24(2)	2(2)	7(2)	3(2)
C(9)	24(2)	17(2)	27(2)	2(2)	9(2)	-1(2)
C(10)	25(2)	30(2)	32(2)	-5(2)	9(2)	1(2)
C(11)	26(2)	35(2)	29(2)	-3(2)	4(2)	1(2)
C(12)	29(2)	37(2)	32(2)	-9(2)	5(2)	-4(2)
C(13)	27(2)	24(2)	25(2)	-5(2)	10(2)	-3(2)
C(14)	25(2)	30(2)	21(2)	-4(2)	7(2)	-2(2)
C(15)	23(2)	30(2)	24(2)	-1(2)	10(2)	-1(2)
C(16)	24(2)	22(2)	32(2)	-4(2)	9(2)	1(2)
C(17)	19(2)	36(2)	30(2)	2(2)	5(2)	4(2)
C(18)	35(3)	48(3)	43(3)	15(2)	5(2)	1(2)
C(19)	23(2)	41(2)	24(2)	3(2)	8(2)	3(2)
C(21)	25(2)	48(3)	36(2)	-13(2)	7(2)	-3(2)
C(22)	31(2)	34(2)	41(3)	-4(2)	14(2)	-3(2)
C(23)	26(2)	34(2)	29(2)	-5(2)	8(2)	0(2)
C(24)	29(2)	24(2)	31(2)	3(2)	8(2)	0(2)
C(25)	24(2)	25(2)	25(2)	-2(2)	7(2)	-1(2)
C(26)	24(2)	24(2)	20(2)	0(2)	4(2)	1(2)
C(27)	27(2)	23(2)	36(2)	1(2)	11(2)	-4(2)
C(28)	27(2)	41(3)	39(3)	-1(2)	7(2)	7(2)
C(30)	26(2)	30(2)	39(3)	7(2)	2(2)	1(2)
C(31)	19(2)	31(2)	32(2)	10(2)	2(2)	0(2)

C(32)	22(2)	45(3)	39(3)	9(2)	0(2)	5(2)
C(33)	54(4)	62(4)	51(3)	-11(3)	-19(3)	13(3)
C(34)	60(4)	113(6)	40(3)	-4(3)	0(3)	30(4)
C(35)	27(2)	25(2)	28(2)	6(2)	4(2)	0(2)
C(36)	194(12)	69(5)	107(7)	10(5)	-82(8)	-46(7)
C(45)	25(2)	29(2)	40(2)	7(2)	9(2)	-3(2)
C(48)	37(3)	51(3)	44(3)	24(2)	15(2)	7(2)
N(1)	25(2)	24(2)	24(2)	-2(1)	9(1)	1(1)
N(2)	22(2)	24(2)	22(2)	-1(1)	9(1)	0(1)
N(3)	18(2)	32(2)	27(2)	0(2)	7(1)	1(1)
N(4)	26(2)	37(2)	29(2)	11(2)	6(2)	3(2)
N(5)	22(2)	39(2)	34(2)	2(2)	5(2)	-3(2)
N(6)	20(2)	23(2)	26(2)	-1(1)	9(1)	1(1)
N(7)	18(2)	41(2)	36(2)	-7(2)	5(2)	0(2)
N(12)	25(2)	24(2)	27(2)	-2(1)	2(1)	2(1)
O(1)	47(2)	24(2)	47(2)	0(1)	-1(2)	4(2)
O(2)	27(2)	28(2)	31(2)	4(1)	11(1)	6(1)
O(3)	27(2)	27(2)	52(2)	-2(1)	21(1)	1(1)
O(4)	37(2)	37(2)	50(2)	-17(2)	24(2)	-6(2)
O(5)	23(2)	42(2)	43(2)	9(2)	12(1)	-2(1)
O(6)	19(2)	50(2)	47(2)	-10(2)	4(1)	1(2)
O(7)	54(2)	32(2)	59(2)	6(2)	13(2)	-8(2)
O(8)	27(2)	32(2)	28(2)	7(1)	8(1)	4(1)
O(9)	36(2)	30(2)	45(2)	-12(2)	14(2)	0(1)
C(1S)	103(7)	99(7)	116(7)	31(6)	32(6)	31(6)
C(2S)	470(40)	101(10)	215(17)	-81(11)	-210(20)	109(16)
C(3S)	310(20)	89(9)	450(40)	-59(15)	310(30)	-52(13)
O(1S)	132(5)	70(4)	123(5)	-2(3)	60(4)	32(4)
O(2S)	88(6)	177(10)	580(30)	206(15)	5(10)	-56(6)
O(3S)	1230(80)	196(15)	103(9)	17(8)	180(20)	250(30)

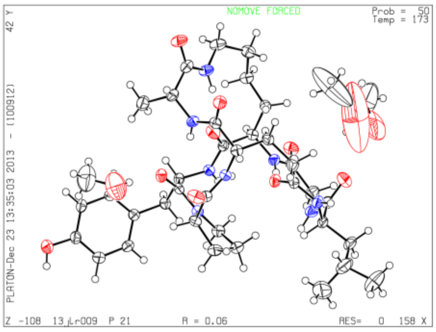

Table S9. Crystal data and structure refinement for **13** (CCDC 1063798)

Empirical formula	C39 H64 N8 O12	
Formula weight	836.98	
Temperature	173(2) K	
Wavelength	0.71073 Å	
Crystal system	Monoclinic	
Space group	P 21	
Unit cell dimensions	a = 17.7309(7) Å	α= 90°.
	b = 9.6245(3) Å	β= 107.755(4)°.
	c = 26.2360(10) Å	γ = 90°.
Volume	4263.9(3) $Å^3$	
Z	4	
Density (calculated)	1.304 Mg/m^3	
Absorption coefficient	0.097 mm^{-1}	
F(000)	1800	
Crystal size	0.3919 x 0.0532 x 0.0426 mm3	
Theta range for data collection	1.630 to 26.702°.	
Index ranges	-20<=h<=22, -12<=k<=11, -32<=l<=31	
Reflections collected	40797	
Independent reflections	15995 [R(int) = 0.0729]	
Completeness to theta = 25.000°	99.8 %	
Absorption correction	Gaussian	
Max. and min. transmission	1 and 0.788	
Refinement method	Full-matrix least-squares on F^2	
Data / restraints / parameters	15995 / 20 / 1084	
Goodness-of-fit on F^2	1.066	
Final R indices [I>2sigma(I)]	R1 = 0.0918, wR2 = 0.2165	
R indices (all data)	R1 = 0.1477, wR2 = 0.2530	
Absolute structure parameter	-1.1(7)	
Extinction coefficient	n/a	
Largest diff. peak and hole	0.567 and -0.542 e.$Å^{-3}$	

Table S10. Atomic coordinates (x 10^4) and equivalent isotropic displacement parameters ($Å^2$x 10^3) for **13**. U(eq) is defined as one third of the trace of the orthogonalized U^{ij} tensor.

	x	y	z	U(eq)
C(015)	3927(5)	11868(10)	-433(4)	32(2)
C(43)	9328(6)	1084(10)	4175(4)	38(2)
C(44)	8825(6)	1427(10)	4547(4)	40(2)
C(62)	8554(8)	7422(10)	4443(5)	57(3)
C(72)	699(9)	2883(16)	3376(6)	93(5)
C(73)	2120(9)	8060(20)	-1126(5)	114(8)
C(101)	4284(5)	11548(10)	-1234(3)	28(2)
C(102)	4810(6)	10495(10)	-982(4)	36(2)

C(103)	3859(6)	12201(11)	-957(4)	38(2)
C(104)	4886(5)	10140(11)	-458(4)	37(2)
C(106)	4456(5)	10823(10)	-169(3)	28(2)
C(107)	4557(5)	10445(10)	399(3)	30(2)
C(108)	4117(5)	9093(9)	463(3)	27(2)
C(109)	3240(5)	9266(11)	172(3)	31(2)
C(110)	3321(7)	7407(15)	-478(5)	73(4)
C(111)	8897(5)	1250(10)	3604(4)	33(2)
C(112)	2119(7)	8783(15)	-614(4)	60(3)
C(113)	1478(7)	8294(15)	-381(4)	56(3)
C(114)	1852(5)	5163(10)	2612(4)	34(2)
C(115)	1382(6)	6078(12)	1726(5)	44(3)
C(116)	906(6)	5918(11)	1156(4)	45(3)
C(117)	1412(6)	6250(12)	773(5)	51(3)
C(118)	1060(6)	7280(11)	332(4)	39(2)
C(119)	760(6)	8607(11)	549(4)	34(2)
C(120)	1036(5)	10989(10)	877(4)	32(2)
C(121)	1171(6)	12177(11)	533(4)	41(2)
C(122)	1445(5)	11246(10)	1470(4)	27(2)
C(123)	2019(6)	10224(10)	2358(3)	35(2)
C(124)	2904(5)	10527(10)	2544(4)	31(2)
C(125)	3426(5)	9341(10)	2469(3)	31(2)
C(126)	3394(5)	9156(10)	1881(4)	31(2)
C(127)	3902(5)	7928(9)	1788(4)	26(2)
C(128)	3808(5)	7923(9)	1193(3)	25(2)
C(129)	3676(5)	4192(9)	2221(4)	28(2)
C(130)	2717(6)	5009(10)	2641(4)	34(2)
C(131)	1643(6)	4022(11)	2973(4)	44(3)
C(132)	836(7)	4167(14)	3060(5)	58(3)
C(133)	705(7)	5479(15)	3320(5)	70(4)
C(140)	4068(5)	5479(9)	2081(3)	22(2)
C(141)	2626(11)	6750(20)	-925(7)	119(8)
C(201)	5507(5)	6680(10)	6212(3)	27(2)
C(202)	5996(5)	7316(11)	5976(4)	37(2)
C(203)	5984(5)	6984(10)	5471(4)	36(2)
C(204)	5477(5)	5964(10)	5166(3)	29(2)
C(205)	4983(5)	5317(11)	5418(4)	33(2)
C(206)	4991(5)	5645(10)	5930(3)	28(2)
C(207)	5451(5)	5599(10)	4608(3)	32(2)

C(208)	5951(5)	4318(10)	4585(3)	26(2)
C(209)	6808(5)	4596(10)	4912(3)	28(2)
C(210)	6690(6)	2930(13)	5620(4)	51(3)
C(211)	7358(7)	2480(20)	6118(6)	119(8)
C(212)	7870(7)	3744(18)	6270(5)	82(5)
C(213)	7885(5)	4321(11)	5743(3)	37(2)
C(214)	8582(5)	3760(11)	5587(4)	32(2)
C(215)	9056(5)	2686(10)	4900(4)	31(2)
C(216)	9289(5)	3929(10)	4613(4)	34(2)
C(217)	8933(6)	6247(10)	4225(4)	35(2)
C(218)	8298(5)	5121(10)	2768(3)	31(2)
C(219)	7408(5)	5408(10)	2536(3)	28(2)
C(220)	6863(5)	4270(10)	2629(3)	28(2)
C(221)	6826(5)	4236(9)	3207(3)	26(2)
C(222)	6331(5)	3035(9)	3316(3)	26(2)
C(223)	6375(5)	3107(10)	3903(3)	22(2)
C(224)	6207(5)	585(9)	3028(3)	24(2)
C(225)	6627(5)	-693(10)	2920(4)	33(2)
C(226)	7678(6)	164(10)	2580(4)	39(2)
C(227)	8545(5)	370(10)	2681(4)	32(2)
C(228)	8860(6)	-706(11)	2363(4)	44(3)
C(229)	9704(6)	-442(13)	2357(5)	54(3)
C(230)	9758(7)	873(14)	2049(6)	75(4)
C(231)	10008(8)	-1666(14)	2118(6)	78(4)
C(232)	8707(5)	6299(10)	3619(4)	31(2)
N(2)	1268(4)	9671(8)	693(3)	28(2)
N(18)	1334(4)	5142(9)	2082(3)	36(2)
N(105)	2883(4)	4357(8)	2241(3)	25(2)
N(110)	3613(4)	6628(8)	1948(3)	30(2)
N(121)	4234(4)	8862(8)	1031(3)	26(2)
N(132)	1654(4)	10121(8)	1779(3)	27(2)
N(201)	5907(4)	4043(8)	4029(3)	25(2)
N(202)	8730(4)	4919(8)	4431(3)	29(2)
N(203)	8437(4)	3010(9)	5139(3)	29(2)
N(204)	9004(4)	325(9)	3248(3)	35(2)
N(205)	8576(4)	5137(8)	3347(3)	27(2)
N(206)	7444(4)	-508(8)	2955(3)	30(2)
N(207)	6650(4)	1720(8)	3199(3)	27(2)
O(101)	4210(4)	11955(8)	-1749(2)	35(2)

N(112)	1654(5)	7627(9)	73(3)	41(2)
N(111)	2937(5)	8499(10)	-266(3)	43(2)
O(104)	778(5)	8619(13)	-638(3)	85(3)
O(105)	3236(4)	5519(8)	3029(3)	43(2)
O(106)	1840(4)	7095(8)	1876(3)	48(2)
O(108)	4755(3)	5427(8)	2076(2)	32(1)
O(109)	3301(4)	7187(8)	873(2)	34(2)
O(110)	1523(4)	12435(7)	1647(3)	41(2)
O(111)	99(4)	8606(8)	600(3)	45(2)
O(122)	2840(3)	10145(7)	324(2)	30(1)
O(201)	5529(4)	7072(8)	6718(2)	33(2)
O(203)	7208(3)	5416(8)	4744(2)	30(1)
N(211)	7101(4)	3942(8)	5379(3)	29(2)
O(205)	9258(4)	4009(9)	5873(3)	46(2)
O(206)	9940(4)	3972(8)	4551(3)	48(2)
O(207)	8682(4)	7448(7)	3402(3)	41(2)
O(208)	6852(3)	2401(7)	4251(2)	31(1)
O(209)	8423(3)	2229(7)	3437(3)	39(2)
O(210)	7190(4)	626(9)	2172(3)	48(2)
O(211)	5491(3)	532(7)	2957(2)	32(1)
C(1S)	3201(8)	3649(15)	757(5)	72(4)
C(2S)	5063(6)	7198(11)	3315(4)	50(3)
C(3S)	5322(7)	1982(12)	1704(5)	55(3)
C(4S)	8571(6)	9305(12)	563(5)	58(3)
C(5S)	6972(6)	9539(11)	4242(5)	61(3)
C(6S)	726(12)	9860(19)	-1734(9)	300
O(1S)	3245(5)	2362(10)	1075(3)	67(2)
O(2S)	4851(4)	5779(7)	3322(3)	38(2)
O(3S)	5525(4)	564(8)	1660(3)	50(2)
O(4S)	8284(10)	8032(17)	707(7)	173(6)
O(5S)	7167(8)	9317(13)	4798(7)	167(7)
O(6S)	167(16)	8870(30)	-1621(8)	341(15)

Table S11. Bond lengths [Å] and angles [°] for **13.**

Bond lengths [Å]	
C(015)-C(103)	1.381(12)
C(015)-C(106)	1.406(12)

C(43)-C(111)	1.469(13)
C(43)-C(44)	1.544(12)
C(44)-C(215)	1.505(13)
C(62)-C(217)	1.513(14)
C(72)-C(132)	1.547(16)
C(73)-C(112)	1.511(17)
C(73)-C(141)	1.54(3)
C(101)-C(103)	1.350(12)
C(101)-O(101)	1.376(10)
C(101)-C(102)	1.399(12)
C(102)-C(104)	1.383(13)
C(104)-C(106)	1.392(12)
C(106)-C(107)	1.493(12)
C(107)-C(108)	1.553(11)
C(108)-N(121)	1.457(11)
C(108)-C(109)	1.517(12)
C(109)-O(122)	1.246(10)
C(109)-N(111)	1.333(11)
C(110)-N(111)	1.452(14)
C(110)-C(141)	1.552(17)
C(111)-O(209)	1.250(10)
C(111)-N(204)	1.345(11)
C(112)-N(111)	1.485(14)
C(112)-C(113)	1.522(14)
C(113)-O(104)	1.259(14)
C(113)-N(112)	1.303(14)
C(114)-N(18)	1.413(12)
C(114)-C(130)	1.519(12)
C(114)-C(131)	1.568(12)
C(115)-O(106)	1.256(12)
C(115)-N(18)	1.318(12)
C(115)-C(116)	1.484(15)
C(116)-C(117)	1.572(14)
C(117)-C(118)	1.506(14)
C(118)-N(112)	1.457(11)
C(118)-C(119)	1.556(13)
C(119)-O(111)	1.220(10)
C(119)-N(2)	1.340(11)
C(120)-N(2)	1.462(11)

C(120)-C(121)	1.520(12)
C(120)-C(122)	1.522(12)
C(122)-O(110)	1.227(10)
C(122)-N(132)	1.335(11)
C(123)-N(132)	1.464(11)
C(123)-C(124)	1.523(12)
C(124)-C(125)	1.520(12)
C(125)-C(126)	1.535(12)
C(126)-C(127)	1.551(11)
C(127)-N(110)	1.462(10)
C(127)-C(128)	1.517(12)
C(128)-O(109)	1.248(10)
C(128)-N(121)	1.329(10)
C(129)-N(105)	1.431(10)
C(129)-C(140)	1.520(10)
C(130)-O(105)	1.245(11)
C(130)-N(105)	1.332(11)
C(131)-C(132)	1.523(14)
C(132)-C(133)	1.486(16)
C(140)-O(108)	1.224(9)
C(140)-N(110)	1.350(10)
C(201)-C(202)	1.354(12)
C(201)-O(201)	1.371(10)
C(201)-C(206)	1.401(12)
C(202)-C(203)	1.356(13)
C(203)-C(204)	1.404(13)
C(204)-C(205)	1.394(12)
C(204)-C(207)	1.493(12)
C(205)-C(206)	1.374(12)
C(207)-C(208)	1.531(11)
C(208)-N(201)	1.462(10)
C(208)-C(209)	1.524(12)
C(209)-O(203)	1.231(10)
C(209)-N(211)	1.333(11)
C(210)-N(211)	1.471(12)
C(210)-C(211)	1.534(15)
C(211)-C(212)	1.50(2)
C(212)-C(213)	1.498(15)
C(213)-N(211)	1.472(11)

C(213)-C(214)	1.517(12)
C(214)-O(205)	1.229(10)
C(214)-N(203)	1.334(11)
C(215)-N(203)	1.454(11)
C(215)-C(216)	1.536(12)
C(216)-O(206)	1.215(10)
C(216)-N(202)	1.352(11)
C(217)-N(202)	1.474(11)
C(217)-C(232)	1.517(13)
C(218)-N(205)	1.446(11)
C(218)-C(219)	1.533(12)
C(219)-C(220)	1.528(11)
C(220)-C(221)	1.537(11)
C(221)-C(222)	1.530(11)
C(222)-N(207)	1.457(10)
C(222)-C(223)	1.520(11)
C(223)-O(208)	1.241(10)
C(223)-N(201)	1.332(10)
C(224)-O(211)	1.228(9)
C(224)-N(207)	1.340(10)
C(224)-C(225)	1.509(11)
C(225)-N(206)	1.434(11)
C(226)-O(210)	1.236(11)
C(226)-N(206)	1.344(11)
C(226)-C(227)	1.492(13)
C(227)-N(204)	1.462(12)
C(227)-C(228)	1.538(12)
C(228)-C(229)	1.524(14)
C(229)-C(231)	1.509(15)
C(229)-C(230)	1.519(16)
C(232)-O(207)	1.238(10)
C(232)-N(205)	1.309(11)
C(1S)-O(1S)	1.483(15)
C(2S)-O(2S)	1.418(11)
C(3S)-O(3S)	1.425(12)
C(4S)-O(4S)	1.421(11)
C(5S)-O(5S)	1.410(18)
C(6S)-O(6S)	1.465(13)

Angles [°]

C(103)-C(015)-C(106)	119.9(8)
C(111)-C(43)-C(44)	113.6(8)
C(215)-C(44)-C(43)	117.4(7)
C(112)-C(73)-C(141)	103.3(12)
C(103)-C(101)-O(101)	119.6(8)
C(103)-C(101)-C(102)	119.0(8)
O(101)-C(101)-C(102)	121.4(8)
C(104)-C(102)-C(101)	119.8(9)
C(101)-C(103)-C(015)	122.3(9)
C(102)-C(104)-C(106)	121.3(9)
C(104)-C(106)-C(015)	117.7(8)
C(104)-C(106)-C(107)	120.6(8)
C(015)-C(106)-C(107)	121.7(8)
C(106)-C(107)-C(108)	113.6(7)
N(121)-C(108)-C(109)	109.7(7)
N(121)-C(108)-C(107)	108.6(6)
C(109)-C(108)-C(107)	109.0(7)
O(122)-C(109)-N(111)	122.2(9)
O(122)-C(109)-C(108)	120.6(7)
N(111)-C(109)-C(108)	117.1(8)
N(111)-C(110)-C(141)	103.1(11)
O(209)-C(111)-N(204)	118.6(9)
O(209)-C(111)-C(43)	121.6(8)
N(204)-C(111)-C(43)	119.9(8)
N(111)-C(112)-C(73)	101.3(11)
N(111)-C(112)-C(113)	113.9(9)
C(73)-C(112)-C(113)	114.8(10)
O(104)-C(113)-N(112)	122.4(9)
O(104)-C(113)-C(112)	116.2(11)
N(112)-C(113)-C(112)	121.3(11)
N(18)-C(114)-C(130)	113.1(7)
N(18)-C(114)-C(131)	112.4(7)
C(130)-C(114)-C(131)	108.8(8)
O(106)-C(115)-N(18)	119.1(10)
O(106)-C(115)-C(116)	120.7(9)
N(18)-C(115)-C(116)	120.2(9)
C(115)-C(116)-C(117)	111.3(8)
C(118)-C(117)-C(116)	116.2(8)
N(112)-C(118)-C(117)	108.7(8)

N(112)-C(118)-C(119)	111.0(8)
C(117)-C(118)-C(119)	111.6(8)
O(111)-C(119)-N(2)	123.7(9)
O(111)-C(119)-C(118)	119.1(8)
N(2)-C(119)-C(118)	117.1(8)
N(2)-C(120)-C(121)	110.2(7)
N(2)-C(120)-C(122)	112.6(7)
C(121)-C(120)-C(122)	111.8(8)
O(110)-C(122)-N(132)	123.2(8)
O(110)-C(122)-C(120)	120.2(8)
N(132)-C(122)-C(120)	116.5(7)
N(132)-C(123)-C(124)	115.5(7)
C(125)-C(124)-C(123)	115.1(7)
C(124)-C(125)-C(126)	112.3(7)
C(125)-C(126)-C(127)	113.7(7)
N(110)-C(127)-C(128)	110.8(7)
N(110)-C(127)-C(126)	109.6(6)
C(128)-C(127)-C(126)	106.2(7)
O(109)-C(128)-N(121)	122.2(8)
O(109)-C(128)-C(127)	121.7(7)
N(121)-C(128)-C(127)	115.6(7)
N(105)-C(129)-C(140)	116.4(7)
O(105)-C(130)-N(105)	123.0(8)
O(105)-C(130)-C(114)	119.0(8)
N(105)-C(130)-C(114)	117.9(8)
C(132)-C(131)-C(114)	115.6(9)
C(133)-C(132)-C(131)	115.6(9)
C(133)-C(132)-C(72)	111.2(10)
C(131)-C(132)-C(72)	108.4(11)
O(108)-C(140)-N(110)	123.3(7)
O(108)-C(140)-C(129)	120.0(7)
N(110)-C(140)-C(129)	116.7(7)
C(73)-C(141)-C(110)	100.0(12)
C(202)-C(201)-O(201)	119.0(8)
C(202)-C(201)-C(206)	119.1(8)
O(201)-C(201)-C(206)	121.9(8)
C(201)-C(202)-C(203)	121.1(9)
C(202)-C(203)-C(204)	122.5(9)
C(205)-C(204)-C(203)	115.3(8)

C(205)-C(204)-C(207)	121.6(8)
C(203)-C(204)-C(207)	123.1(8)
C(206)-C(205)-C(204)	122.6(9)
C(205)-C(206)-C(201)	119.3(8)
C(204)-C(207)-C(208)	112.5(7)
N(201)-C(208)-C(209)	109.6(6)
N(201)-C(208)-C(207)	109.3(6)
C(209)-C(208)-C(207)	109.1(7)
O(203)-C(209)-N(211)	121.8(8)
O(203)-C(209)-C(208)	119.7(8)
N(211)-C(209)-C(208)	118.6(8)
N(211)-C(210)-C(211)	101.4(9)
C(212)-C(211)-C(210)	104.2(11)
C(213)-C(212)-C(211)	103.6(11)
N(211)-C(213)-C(212)	103.2(9)
N(211)-C(213)-C(214)	115.0(7)
C(212)-C(213)-C(214)	111.3(8)
O(205)-C(214)-N(203)	122.3(8)
O(205)-C(214)-C(213)	119.3(8)
N(203)-C(214)-C(213)	118.4(8)
N(203)-C(215)-C(44)	109.9(7)
N(203)-C(215)-C(216)	113.3(7)
C(44)-C(215)-C(216)	112.8(7)
O(206)-C(216)-N(202)	123.5(9)
O(206)-C(216)-C(215)	120.1(9)
N(202)-C(216)-C(215)	116.4(8)
N(202)-C(217)-C(62)	108.9(8)
N(202)-C(217)-C(232)	113.3(7)
C(62)-C(217)-C(232)	111.4(8)
N(205)-C(218)-C(219)	113.2(7)
C(220)-C(219)-C(218)	115.6(7)
C(219)-C(220)-C(221)	113.0(7)
C(222)-C(221)-C(220)	113.5(7)
N(207)-C(222)-C(223)	110.5(7)
N(207)-C(222)-C(221)	109.6(6)
C(223)-C(222)-C(221)	107.5(6)
O(208)-C(223)-N(201)	121.6(7)
O(208)-C(223)-C(222)	122.2(7)
N(201)-C(223)-C(222)	116.1(7)

O(211)-C(224)-N(207)	123.9(7)
O(211)-C(224)-C(225)	119.0(7)
N(207)-C(224)-C(225)	117.1(7)
N(206)-C(225)-C(224)	115.7(7)
O(210)-C(226)-N(206)	121.1(9)
O(210)-C(226)-C(227)	121.2(8)
N(206)-C(226)-C(227)	117.7(9)
N(204)-C(227)-C(226)	113.6(7)
N(204)-C(227)-C(228)	110.5(7)
C(226)-C(227)-C(228)	109.9(8)
C(229)-C(228)-C(227)	114.5(9)
C(231)-C(229)-C(230)	110.0(9)
C(231)-C(229)-C(228)	110.5(10)
C(230)-C(229)-C(228)	111.4(9)
O(207)-C(232)-N(205)	122.4(9)
O(207)-C(232)-C(217)	118.2(8)
N(205)-C(232)-C(217)	119.3(7)
C(119)-N(2)-C(120)	121.6(7)
C(115)-N(18)-C(114)	121.6(8)
C(130)-N(105)-C(129)	122.5(8)
C(140)-N(110)-C(127)	122.7(7)
C(128)-N(121)-C(108)	121.0(7)
C(122)-N(132)-C(123)	121.9(7)
C(223)-N(201)-C(208)	121.5(7)
C(216)-N(202)-C(217)	120.5(7)
C(214)-N(203)-C(215)	121.7(8)
C(111)-N(204)-C(227)	122.3(7)
C(232)-N(205)-C(218)	122.0(7)
C(226)-N(206)-C(225)	123.0(8)
C(224)-N(207)-C(222)	123.6(7)
C(113)-N(112)-C(118)	122.2(9)
C(109)-N(111)-C(110)	128.2(9)
C(109)-N(111)-C(112)	119.3(9)
C(110)-N(111)-C(112)	112.4(8)
C(209)-N(211)-C(210)	127.0(7)
C(209)-N(211)-C(213)	120.4(8)
C(210)-N(211)-C(213)	112.3(7)

Table S12. Anisotropic displacement parameters ($Å^2$x 10^3) for **13**. The anisotropic displacement factor exponent takes

the form: $-2\pi^2[h^2a^{*2}U^{11} + ... + 2 h k a^* b^* U^{12}]$

	U^{11}	U^{22}	U^{33}	U^{23}	U^{13}	U^{12}
C(015)	35(5)	33(5)	33(6)	3(4)	18(4)	6(4)
C(43)	36(6)	24(5)	59(7)	-3(4)	22(5)	4(4)
C(44)	38(6)	23(5)	69(7)	-1(4)	34(5)	1(4)
C(62)	87(9)	18(5)	68(8)	-6(5)	27(7)	-4(5)
C(72)	120(13)	96(11)	86(11)	-6(8)	63(10)	-59(10)
C(73)	82(12)	230(20)	33(8)	-50(12)	23(8)	-79(14)
C(101)	27(5)	29(5)	26(5)	0(4)	4(4)	-11(4)
C(102)	45(6)	29(5)	38(6)	3(4)	16(5)	1(4)
C(103)	36(6)	41(5)	41(6)	6(5)	17(5)	8(4)
C(104)	37(5)	35(5)	38(6)	9(4)	11(5)	6(4)
C(106)	27(5)	25(4)	33(5)	-4(4)	9(4)	-6(4)
C(107)	28(5)	32(5)	31(5)	0(4)	11(4)	-9(4)
C(108)	25(5)	19(4)	39(5)	-3(4)	13(4)	-6(3)
C(109)	31(5)	41(5)	25(5)	-7(4)	13(4)	-16(4)
C(110)	68(8)	95(10)	67(9)	-61(8)	39(7)	-36(7)
C(111)	29(5)	14(4)	61(7)	2(4)	22(5)	-2(4)
C(112)	53(7)	102(10)	21(6)	-11(6)	5(5)	-27(7)
C(113)	43(7)	105(10)	25(6)	-29(6)	17(5)	-40(7)
C(114)	31(5)	28(5)	45(6)	-8(4)	16(5)	-5(4)
C(115)	29(6)	46(6)	70(8)	-3(5)	31(5)	9(5)
C(116)	29(5)	41(6)	66(8)	18(5)	13(5)	-3(4)
C(117)	43(6)	36(6)	80(9)	-1(5)	27(6)	-4(5)
C(118)	36(5)	50(6)	36(6)	-15(5)	17(5)	-11(5)
C(119)	35(6)	40(5)	28(5)	-11(4)	11(4)	-8(4)
C(120)	30(5)	30(5)	36(5)	-4(4)	9(4)	5(4)
C(121)	43(6)	37(5)	40(6)	14(4)	8(5)	4(5)
C(122)	19(4)	28(5)	38(5)	-5(4)	13(4)	-2(4)
C(123)	53(6)	29(5)	27(5)	-2(4)	19(4)	7(4)
C(124)	38(5)	26(5)	28(5)	-2(4)	9(4)	-2(4)
C(125)	37(5)	29(5)	28(5)	-1(4)	10(4)	1(4)
C(126)	36(5)	23(4)	38(5)	0(4)	16(4)	2(4)
C(127)	28(5)	16(4)	41(5)	2(4)	20(4)	-3(3)
C(128)	32(5)	13(4)	30(5)	0(3)	8(4)	1(4)
C(129)	30(5)	22(4)	31(5)	-2(4)	9(4)	-3(4)
C(130)	37(6)	29(5)	42(6)	3(4)	17(5)	-10(4)

C(131)	52(7)	35(5)	54(7)	-1(5)	28(5)	-10(5)
C(132)	40(6)	81(9)	59(7)	-4(6)	26(6)	-25(6)
C(133)	49(7)	91(10)	89(10)	-21(8)	50(7)	-10(7)
C(140)	25(4)	12(4)	33(5)	-2(3)	16(4)	-6(3)
C(141)	130(16)	159(17)	98(13)	-106(13)	80(12)	-102(14)
C(201)	24(5)	29(5)	27(5)	-6(4)	5(4)	7(4)
C(202)	30(5)	41(5)	41(6)	-9(5)	14(5)	-4(4)
C(203)	33(5)	34(5)	46(6)	-4(4)	18(5)	-3(4)
C(204)	28(5)	28(5)	30(5)	5(4)	8(4)	14(4)
C(205)	30(5)	32(5)	39(6)	-6(4)	13(4)	1(4)
C(206)	24(5)	27(4)	36(6)	-3(4)	13(4)	4(4)
C(207)	29(5)	35(5)	30(5)	-2(4)	8(4)	10(4)
C(208)	23(5)	26(4)	29(5)	6(4)	10(4)	6(4)
C(209)	25(5)	31(5)	30(5)	-5(4)	11(4)	8(4)
C(210)	31(6)	71(7)	51(7)	41(6)	10(5)	10(5)
C(211)	32(7)	240(20)	96(11)	130(13)	29(7)	39(10)
C(212)	32(7)	170(15)	45(8)	31(9)	16(6)	28(9)
C(213)	35(6)	52(6)	19(5)	-11(4)	1(4)	5(5)
C(214)	29(5)	38(5)	29(5)	6(4)	9(4)	-1(4)
C(215)	30(5)	33(5)	32(5)	12(4)	10(4)	4(4)
C(216)	27(5)	37(5)	36(5)	-6(4)	8(4)	-9(4)
C(217)	33(5)	20(4)	45(6)	0(4)	1(4)	-9(4)
C(218)	27(5)	28(5)	39(6)	7(4)	10(4)	2(4)
C(219)	27(5)	28(4)	28(5)	6(4)	9(4)	-2(4)
C(220)	26(5)	32(5)	23(5)	0(4)	4(4)	3(4)
C(221)	20(4)	22(4)	36(5)	-2(4)	9(4)	-1(3)
C(222)	20(4)	18(4)	41(5)	-4(4)	11(4)	-4(3)
C(223)	14(4)	28(4)	22(5)	0(4)	3(4)	-3(4)
C(224)	37(5)	14(4)	25(5)	1(3)	16(4)	-4(4)
C(225)	40(6)	22(4)	37(5)	1(4)	12(4)	-2(4)
C(226)	54(6)	26(5)	45(6)	6(4)	29(5)	16(5)
C(227)	46(6)	20(4)	41(6)	11(4)	27(5)	12(4)
C(228)	49(6)	32(5)	63(7)	5(5)	35(6)	4(5)
C(229)	45(7)	66(7)	62(8)	10(6)	33(6)	16(6)
C(230)	55(8)	88(10)	106(11)	26(8)	58(8)	-1(7)
C(231)	69(9)	78(9)	108(11)	8(8)	59(9)	32(7)
C(232)	21(5)	21(5)	50(6)	7(4)	11(4)	-1(4)
N(2)	26(4)	26(4)	31(4)	-4(3)	8(3)	-5(3)
N(18)	39(5)	22(4)	55(5)	7(4)	26(4)	-6(3)

N(105)	28(4)	17(3)	35(4)	1(3)	16(3)	2(3)
N(110)	26(4)	26(4)	42(5)	4(3)	17(4)	2(3)
N(121)	25(4)	20(4)	34(4)	-1(3)	9(3)	-6(3)
N(132)	27(4)	21(4)	33(4)	-3(3)	10(3)	2(3)
N(201)	20(4)	27(4)	27(4)	1(3)	6(3)	7(3)
N(202)	25(4)	21(4)	36(5)	4(3)	3(3)	-5(3)
N(203)	25(4)	39(4)	25(4)	5(3)	9(3)	7(3)
N(204)	36(4)	23(4)	58(6)	7(4)	31(4)	9(3)
N(205)	25(4)	18(4)	36(4)	6(3)	7(3)	1(3)
N(206)	36(4)	19(3)	41(5)	0(3)	21(4)	7(3)
N(207)	21(4)	23(4)	38(5)	-5(3)	12(3)	-1(3)
O(101)	33(4)	45(4)	29(4)	7(3)	12(3)	0(3)
N(112)	40(5)	52(5)	36(5)	-18(4)	19(4)	-20(4)
N(111)	42(5)	60(5)	31(5)	-21(4)	16(4)	-24(4)
O(104)	48(6)	162(10)	39(5)	-2(5)	6(4)	-34(6)
O(105)	41(4)	43(4)	49(4)	-15(3)	21(3)	-5(3)
O(106)	47(4)	19(3)	88(6)	-5(3)	34(4)	-8(3)
O(108)	30(3)	32(3)	39(4)	5(3)	19(3)	5(3)
O(109)	39(4)	29(3)	36(4)	-1(3)	14(3)	-4(3)
O(110)	52(4)	19(3)	49(4)	-9(3)	11(3)	0(3)
O(111)	32(4)	44(4)	66(5)	-13(3)	25(4)	-5(3)
O(122)	25(3)	32(3)	31(4)	-3(3)	3(3)	0(3)
O(201)	29(4)	42(4)	30(4)	-7(3)	12(3)	-4(3)
O(203)	23(3)	29(3)	35(4)	9(3)	6(3)	-2(3)
N(211)	27(4)	34(4)	25(4)	7(3)	7(3)	9(3)
O(205)	29(4)	66(5)	41(4)	-6(3)	7(3)	-9(3)
O(206)	34(4)	38(4)	79(5)	7(4)	25(4)	-5(3)
O(207)	44(4)	18(3)	61(5)	10(3)	16(3)	-2(3)
O(208)	27(3)	28(3)	36(4)	0(3)	8(3)	6(3)
O(209)	30(4)	15(3)	75(5)	0(3)	20(3)	2(3)
O(210)	43(4)	56(5)	49(5)	20(4)	23(4)	13(4)
O(211)	27(3)	26(3)	43(4)	-4(3)	10(3)	-8(3)
C(1S)	58(8)	76(9)	85(10)	-21(8)	29(7)	-13(7)
C(2S)	57(7)	40(6)	48(7)	9(5)	8(5)	-8(5)
C(3S)	55(7)	40(6)	60(8)	-2(5)	4(6)	3(5)
C(4S)	31(6)	41(6)	113(10)	3(6)	35(6)	7(5)
C(5S)	38(7)	31(6)	110(11)	13(6)	15(7)	-4(5)
O(1S)	69(6)	64(5)	60(6)	-17(4)	8(5)	9(5)
O(2S)	29(3)	34(3)	41(4)	7(3)	-3(3)	3(3)

O(3S)	49(5)	44(4)	46(5)	-6(3)	-2(3)	-6(3)
O(4S)	131(14)	202(17)	167(15)	-18(13)	20(10)	-36(12)
O(5S)	179(14)	88(9)	300(20)	64(11)	177(15)	24(9)

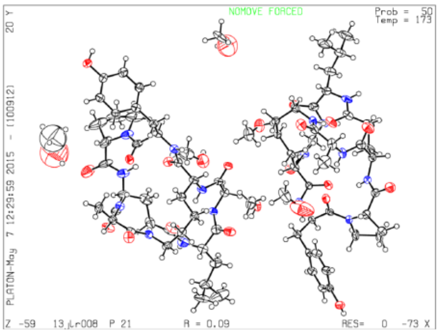

Table S13 Crystal data and structure refinement for **15** (CCDC 1063796)

Empirical formula	C40 H68 N8 O13	
Formula weight	869.02	
Temperature	123(2) K	
Wavelength	0.71073 Å	
Crystal system	Monoclinic	
Space group	P 1 21 1	
Unit cell dimensions	a = 13.3658(2) Å	α= 90°.
	b = 9.48640(10) Å	β= 104.2040(10)°.
	c = 17.7634(2) Å	γ = 90°.
Volume	2183.42(5) $Å^3$	
Z	2	
Density (calculated)	1.322 Mg/m^3	
Absorption coefficient	0.099 mm^{-1}	
F(000)	936	
Crystal size	0.3069 x 0.1396 x 0.0564 mm^3	
Theta range for data collection	1.57 to 27.31°.	
Index ranges	-16<=h<=16, -12<=k<=12, -22<=l<=22	
Reflections collected	33579	
Independent reflections	8949 [R(int) = 0.0296]	
Completeness to theta = 25.00°	100.0 %	
Absorption correction	Gaussian	
Max. and min. transmission	1.351 and 1.074	
Refinement method	Full-matrix least-squares on F^2	
Data / restraints / parameters	8949 / 11 / 553	
Goodness-of-fit on F^2	1.080	
Final R indices [I>2sigma(I)]	R1 = 0.0575, wR2 = 0.1487	
R indices (all data)	R1 = 0.0623, wR2 = 0.1518	
Absolute structure parameter	0.6(11)	
Largest diff. peak and hole	0.997 and -0.359 e.$Å^{-3}$	

Table S14. Atomic coordinates (x 10^4) and equivalent isotropic displacement parameters ($Å^2$x 10^3) for **15**. U(eq) is defined as one third of the trace of the orthogonalized U^{ij} tensor.

	x	y	z	U(eq)
C(1)	12416(2)	2280(3)	5623(2)	22(1)
C(2)	11948(2)	3320(3)	5104(2)	26(1)
C(3)	10919(2)	3654(3)	5032(2)	28(1)
C(4)	10331(2)	2959(3)	5469(2)	23(1)

C(5)	10821(2)	1945(3)	5996(2)	24(1)
C(6)	11846(2)	1586(3)	6076(2)	26(1)
C(7)	9205(2)	3328(3)	5378(2)	23(1)
C(8)	9051(2)	4685(3)	5816(2)	21(1)
C(9)	9638(2)	4513(3)	6665(2)	25(1)
C(10)	10854(3)	6471(5)	6542(2)	45(1)
C(11)	11688(4)	7120(7)	7193(3)	75(2)
C(12)	12131(3)	5856(7)	7687(3)	68(2)
C(13)	11177(3)	4946(5)	7698(2)	42(1)
C(14)	10712(3)	5324(4)	8379(2)	40(1)
C(15)	9309(2)	6381(4)	8839(2)	27(1)
C(16)	8775(2)	5046(4)	9028(2)	27(1)
C(17)	8197(3)	2628(3)	8597(2)	28(1)
C(18)	8787(3)	1944(4)	9357(2)	41(1)
C(19)	7047(3)	2498(3)	8481(2)	28(1)
C(20)	5353(2)	3635(4)	8120(2)	29(1)
C(21)	4934(2)	3342(3)	7248(2)	26(1)
C(22)	5131(2)	4513(3)	6710(2)	22(1)
C(23)	6248(2)	4598(3)	6645(2)	21(1)
C(25)	6480(2)	5840(3)	6154(2)	19(1)
C(26)	7647(2)	5824(3)	6208(2)	20(1)
C(27)	5876(2)	8298(3)	6022(2)	20(1)
C(28)	5591(2)	9612(3)	6429(2)	24(1)
C(29)	4828(2)	8706(3)	7446(2)	26(1)
C(30)	4942(3)	8501(4)	8317(2)	30(1)
C(31)	4299(3)	9600(4)	8623(2)	39(1)
C(32)	4244(3)	9351(6)	9463(2)	52(1)
C(33)	3805(4)	10643(7)	9777(3)	71(2)
C(34)	3631(4)	8049(6)	9552(2)	63(1)
C(35)	6724(3)	7641(3)	8657(2)	30(1)
C(36)	7814(3)	7893(4)	9103(2)	37(1)
C(37)	8569(3)	7622(4)	8588(2)	36(1)
N(1)	7961(2)	4878(3)	5759(1)	21(1)
N(2)	10487(2)	5271(3)	6930(2)	29(1)
N(3)	9858(2)	6134(3)	8231(2)	31(1)
N(4)	8562(2)	4052(3)	8485(1)	25(1)
N(5)	6475(2)	3658(3)	8366(1)	25(1)
N(6)	6178(2)	7158(2)	6455(1)	21(1)
N(7)	5591(2)	9409(3)	7235(2)	24(1)

N(8)	6010(2)	8573(3)	8769(2)	29(1)
O(1)	13415(2)	1872(2)	5700(1)	26(1)
O(2)	9360(2)	3602(2)	7073(1)	24(1)
O(3)	11131(2)	4884(4)	9027(1)	50(1)
O(4)	8532(2)	4951(3)	9655(1)	39(1)
O(5)	6665(2)	1297(2)	8475(1)	37(1)
O(6)	8243(2)	6567(2)	6685(1)	26(1)
O(7)	5820(2)	8374(2)	5321(1)	25(1)
O(8)	4078(2)	8210(3)	6981(1)	32(1)
O(9)	6472(2)	6646(2)	8200(1)	34(1)
C(1S)	8471(4)	136(6)	6902(3)	70(1)
O(1S)	7907(2)	1390(3)	6735(2)	52(1)
O(2S)	10795(3)	-194(4)	8695(2)	79(1)
C(2S)	11015(4)	1109(6)	8398(3)	59(1)
C(3S)	6598(3)	1766(4)	4794(2)	37(1)
O(3S)	6731(2)	3189(2)	4601(1)	35(1)
O(4S)	6836(9)	9850(13)	10265(7)	60(4)
C(4S)	6510(30)	9030(40)	10841(18)	138(13)

Table S15. Bond lengths [Å] and angles [°] for **15**.

Bond lengths [Å]	
C(1)-O(1)	1.365(4)
C(1)-C(2)	1.389(4)
C(1)-C(6)	1.400(4)
C(2)-C(3)	1.387(4)
C(3)-C(4)	1.397(4)
C(4)-C(5)	1.389(4)
C(4)-C(7)	1.516(4)
C(5)-C(6)	1.385(4)
C(7)-C(8)	1.543(4)
C(8)-N(1)	1.448(4)
C(8)-C(9)	1.528(4)
C(9)-O(2)	1.242(4)
C(9)-N(2)	1.328(4)
C(10)-N(2)	1.475(5)
C(10)-C(11)	1.525(6)
C(11)-C(12)	1.518(9)

C(12)-C(13)	1.544(6)
C(13)-N(2)	1.480(5)
C(13)-C(14)	1.533(5)
C(14)-O(3)	1.223(5)
C(14)-N(3)	1.347(5)
C(15)-N(3)	1.465(4)
C(15)-C(16)	1.530(5)
C(15)-C(37)	1.531(5)
C(16)-O(4)	1.237(4)
C(16)-N(4)	1.329(4)
C(17)-N(4)	1.466(4)
C(17)-C(19)	1.505(5)
C(17)-C(18)	1.531(4)
C(19)-O(5)	1.248(4)
C(19)-N(5)	1.327(4)
C(20)-N(5)	1.455(4)
C(20)-C(21)	1.537(4)
C(21)-C(22)	1.531(4)
C(22)-C(23)	1.528(4)
C(23)-C(25)	1.542(4)
C(25)-N(6)	1.456(3)
C(25)-C(26)	1.539(4)
C(26)-O(6)	1.232(4)
C(26)-N(1)	1.335(4)
C(27)-O(7)	1.231(3)
C(27)-N(6)	1.331(4)
C(27)-C(28)	1.535(4)
C(28)-N(7)	1.446(4)
C(29)-O(8)	1.225(4)
C(29)-N(7)	1.345(4)
C(29)-C(30)	1.530(4)
C(30)-N(8)	1.457(4)
C(30)-C(31)	1.534(5)
C(31)-C(32)	1.531(5)
C(32)-C(34)	1.511(7)
C(32)-C(33)	1.523(6)
C(35)-O(9)	1.236(4)
C(35)-N(8)	1.351(4)
C(35)-C(36)	1.498(5)

C(36)-C(37)	1.540(5)
C(1S)-O(1S)	1.400(6)
O(2S)-C(2S)	1.403(6)
C(3S)-O(3S)	1.414(4)
O(4S)-C(4S)	1.438(18)

Angles [°]

O(1)-C(1)-C(2)	122.5(3)
O(1)-C(1)-C(6)	117.8(3)
C(2)-C(1)-C(6)	119.7(3)
C(3)-C(2)-C(1)	119.9(3)
C(2)-C(3)-C(4)	121.4(3)
C(5)-C(4)-C(3)	117.7(3)
C(5)-C(4)-C(7)	121.6(3)
C(3)-C(4)-C(7)	120.7(3)
C(4)-C(5)-C(6)	122.1(3)
C(5)-C(6)-C(1)	119.3(3)
C(4)-C(7)-C(8)	112.9(2)
N(1)-C(8)-C(9)	110.2(2)
N(1)-C(8)-C(7)	109.0(2)
C(9)-C(8)-C(7)	108.0(2)
O(2)-C(9)-N(2)	121.4(3)
O(2)-C(9)-C(8)	119.7(3)
N(2)-C(9)-C(8)	118.6(3)
N(2)-C(10)-C(11)	102.9(4)
C(12)-C(11)-C(10)	103.2(4)
C(11)-C(12)-C(13)	104.1(4)
N(2)-C(13)-C(14)	113.4(3)
N(2)-C(13)-C(12)	101.6(3)
C(14)-C(13)-C(12)	112.0(3)
O(3)-C(14)-N(3)	123.3(3)
O(3)-C(14)-C(13)	118.9(3)
N(3)-C(14)-C(13)	117.8(3)
N(3)-C(15)-C(16)	112.0(3)
N(3)-C(15)-C(37)	108.3(3)
C(16)-C(15)-C(37)	113.4(3)
O(4)-C(16)-N(4)	122.9(3)
O(4)-C(16)-C(15)	119.9(3)
N(4)-C(16)-C(15)	117.2(3)
N(4)-C(17)-C(19)	114.8(3)

N(4)-C(17)-C(18)	113.2(3)
C(19)-C(17)-C(18)	111.9(3)
O(5)-C(19)-N(5)	122.5(3)
O(5)-C(19)-C(17)	118.6(3)
N(5)-C(19)-C(17)	118.9(3)
N(5)-C(20)-C(21)	113.5(3)
C(22)-C(21)-C(20)	115.0(3)
C(23)-C(22)-C(21)	114.0(2)
C(22)-C(23)-C(25)	114.7(2)
N(6)-C(25)-C(26)	110.7(2)
N(6)-C(25)-C(23)	109.6(2)
C(26)-C(25)-C(23)	107.4(2)
O(6)-C(26)-N(1)	123.4(3)
O(6)-C(26)-C(25)	120.8(2)
N(1)-C(26)-C(25)	115.5(2)
O(7)-C(27)-N(6)	124.5(3)
O(7)-C(27)-C(28)	117.9(3)
N(6)-C(27)-C(28)	117.6(2)
N(7)-C(28)-C(27)	114.8(2)
O(8)-C(29)-N(7)	123.6(3)
O(8)-C(29)-C(30)	119.6(3)
N(7)-C(29)-C(30)	116.7(3)
N(8)-C(30)-C(29)	113.1(3)
N(8)-C(30)-C(31)	109.4(3)
C(29)-C(30)-C(31)	110.3(3)
C(32)-C(31)-C(30)	113.9(4)
C(34)-C(32)-C(33)	110.6(4)
C(34)-C(32)-C(31)	112.6(3)
C(33)-C(32)-C(31)	110.4(4)
O(9)-C(35)-N(8)	120.6(3)
O(9)-C(35)-C(36)	122.8(3)
N(8)-C(35)-C(36)	116.6(3)
C(35)-C(36)-C(37)	110.7(3)
C(15)-C(37)-C(36)	115.2(3)
C(26)-N(1)-C(8)	120.2(2)
C(9)-N(2)-C(10)	127.3(3)
C(9)-N(2)-C(13)	119.9(3)
C(10)-N(2)-C(13)	112.8(3)
C(14)-N(3)-C(15)	120.0(3)

C(16)-N(4)-C(17)	125.1(3)
C(19)-N(5)-C(20)	123.1(3)
C(27)-N(6)-C(25)	123.8(2)
C(29)-N(7)-C(28)	121.7(3)
C(35)-N(8)-C(30)	121.3(3)

Table S16. Anisotropic displacement parameters ($Å^2$x 10^3) for **15**. The anisotropic displacement factor exponent takes the form: $-2\pi^2$[$h^2a^{*2}U^{11}$ + ... + 2 h k a* b* U^{12}]

	U^{11}	U^{22}	U^{33}	U^{23}	U^{13}	U^{12}
C(1)	20(1)	24(2)	23(1)	-7(1)	7(1)	-1(1)
C(2)	30(2)	24(2)	28(1)	2(1)	17(1)	-1(1)
C(3)	30(2)	26(2)	31(2)	1(1)	13(1)	6(1)
C(4)	21(2)	27(2)	23(1)	-5(1)	10(1)	0(1)
C(5)	25(2)	25(2)	26(1)	-3(1)	12(1)	-3(1)
C(6)	26(2)	27(2)	24(1)	3(1)	7(1)	4(1)
C(7)	21(1)	24(2)	27(1)	-5(1)	10(1)	1(1)
C(8)	22(1)	20(1)	25(1)	-2(1)	13(1)	-2(1)
C(9)	24(2)	26(2)	28(2)	-9(1)	14(1)	-4(1)
C(10)	44(2)	58(3)	44(2)	-21(2)	28(2)	-34(2)
C(11)	58(3)	110(5)	67(3)	-44(3)	35(3)	-62(3)
C(12)	28(2)	130(5)	48(2)	-33(3)	13(2)	-33(3)
C(13)	23(2)	72(3)	33(2)	-16(2)	9(1)	-10(2)
C(14)	22(2)	60(2)	38(2)	-22(2)	7(1)	-11(2)
C(15)	24(2)	38(2)	22(1)	-10(1)	11(1)	-8(1)
C(16)	23(2)	37(2)	21(1)	0(1)	4(1)	-1(1)
C(17)	36(2)	26(2)	22(1)	4(1)	8(1)	-1(1)
C(18)	43(2)	40(2)	37(2)	15(2)	5(2)	10(2)
C(19)	41(2)	25(2)	17(1)	4(1)	5(1)	-2(1)
C(20)	30(2)	32(2)	28(2)	5(1)	11(1)	-5(1)
C(21)	21(1)	29(2)	28(2)	2(1)	5(1)	-6(1)
C(22)	20(1)	24(1)	22(1)	1(1)	3(1)	-2(1)
C(23)	24(2)	15(1)	26(1)	2(1)	10(1)	2(1)
C(25)	23(1)	17(1)	20(1)	-1(1)	9(1)	1(1)
C(26)	27(2)	16(1)	18(1)	3(1)	9(1)	2(1)
C(27)	17(1)	21(1)	27(1)	1(1)	14(1)	-2(1)
C(28)	26(2)	18(1)	34(2)	1(1)	15(1)	2(1)
C(29)	32(2)	23(2)	29(2)	-6(1)	18(1)	-2(1)

C(30)	34(2)	33(2)	29(2)	-6(1)	16(1)	-9(1)
C(31)	33(2)	48(2)	42(2)	-20(2)	19(2)	-8(2)
C(32)	31(2)	93(3)	34(2)	-30(2)	13(2)	-7(2)
C(33)	51(3)	106(4)	62(3)	-49(3)	30(2)	-9(3)
C(34)	54(3)	107(4)	36(2)	-11(2)	25(2)	-19(3)
C(35)	50(2)	23(2)	20(1)	0(1)	17(1)	-1(1)
C(36)	43(2)	42(2)	29(2)	-7(2)	13(2)	7(2)
C(37)	46(2)	35(2)	32(2)	-4(1)	21(2)	-5(2)
N(1)	22(1)	22(1)	21(1)	0(1)	9(1)	0(1)
N(2)	22(1)	40(2)	28(1)	-11(1)	11(1)	-7(1)
N(3)	26(1)	40(2)	28(1)	-9(1)	13(1)	-10(1)
N(4)	27(1)	32(1)	18(1)	1(1)	6(1)	-2(1)
N(5)	27(1)	24(1)	23(1)	2(1)	4(1)	-9(1)
N(6)	28(1)	18(1)	20(1)	-1(1)	12(1)	4(1)
N(7)	26(1)	20(1)	29(1)	-3(1)	15(1)	-2(1)
N(8)	34(1)	33(2)	25(1)	-9(1)	16(1)	-5(1)
O(1)	21(1)	31(1)	28(1)	0(1)	9(1)	-1(1)
O(2)	26(1)	28(1)	22(1)	0(1)	9(1)	-1(1)
O(3)	32(1)	87(2)	28(1)	-18(1)	2(1)	4(1)
O(4)	48(2)	49(2)	24(1)	-4(1)	17(1)	-5(1)
O(5)	48(2)	24(1)	38(1)	6(1)	8(1)	-9(1)
O(6)	27(1)	23(1)	29(1)	-7(1)	10(1)	-4(1)
O(7)	29(1)	25(1)	24(1)	6(1)	12(1)	5(1)
O(8)	34(1)	38(1)	29(1)	-4(1)	16(1)	-9(1)
O(9)	53(2)	21(1)	29(1)	0(1)	15(1)	4(1)
C(1S)	81(4)	64(3)	71(3)	-8(3)	31(3)	-4(3)
O(1S)	56(2)	38(2)	54(2)	5(1)	0(1)	-17(1)
O(2S)	103(3)	81(3)	45(2)	-1(2)	5(2)	-32(2)
C(2S)	57(3)	70(3)	49(2)	17(2)	9(2)	11(2)
C(3S)	36(2)	30(2)	39(2)	-3(2)	1(2)	-3(2)
O(3S)	38(1)	31(1)	30(1)	-2(1)	-1(1)	-3(1)

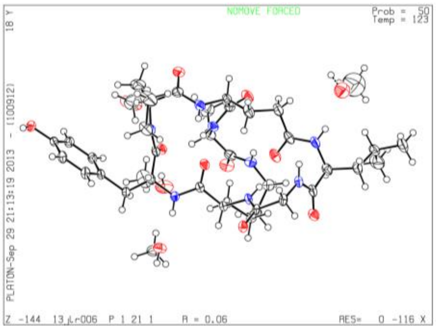

Table S17. Crystal data and structure refinement for **32** (CCDC 1063793).

Empirical formula	C74 H112 N16 O18	
Formula weight	1513.79	
Temperature	120(2) K	
Wavelength	0.71073 Å	
Crystal system	Monoclinic	
Space group	P 21	
Unit cell dimensions	a = 9.2715(4) Å	α= 90°.
	b = 36.7577(15) Å	β= 90.098(4)°.
	c = 11.3129(5) Å	γ = 90°.
Volume	3855.4(3) $Å^3$	
Z	2	
Density (calculated)	1.304 Mg/m^3	
Absorption coefficient	0.094 mm^{-1}	
F(000)	1624	
Crystal size	? x ? x ? mm^3	
Theta range for data collection	1.800 to 24.713°.	
Index ranges	-10<=h<=10, -41<=k<=43, -13<=l<=13	
Reflections collected	26472	
Independent reflections	11905 [R(int) = 0.1025]	
Completeness to theta = 25.242°	94.0 %	
Refinement method	Full-matrix least-squares on F^2	
Data / restraints / parameters	11905 / 481 / 964	
Goodness-of-fit on F^2	1.063	
Final R indices [I>2sigma(I)]	R1 = 0.1570, wR2 = 0.3787	
R indices (all data)	R1 = 0.2072, wR2 = 0.4110	
Absolute structure parameter	1.8(10)	
Extinction coefficient	0.020(3)	
Largest diff. peak and hole	0.824 and -0.597 e.$Å^{-3}$	

Table S18. Atomic coordinates (x 10^4) and equivalent isotropic displacement parameters ($Å^2$x 10^3) for **32**. U(eq) is defined as one third of the trace of the orthogonalized U^{ij} tensor.

	x	y	z	U(eq)
C(101)	2820(40)	3706(10)	7160(30)	77(10)
C(102)	3610(40)	3714(11)	6140(40)	89(11)
C(103)	5000(40)	3897(11)	6270(40)	91(11)
C(104)	5510(40)	4015(9)	7280(30)	71(9)
C(105)	4650(40)	3984(10)	8230(30)	77(10)
C(106)	3170(40)	3851(10)	8140(30)	79(10)
C(107)	7050(20)	4164(6)	7450(20)	33(5)
C(108)	7320(20)	4465(6)	8300(20)	36(5)

C(109)	6430(20)	4827(5)	8068(17)	19(4)
C(110)	7160(20)	5087(6)	10020(17)	25(5)
C(111)	6300(20)	5344(6)	10758(18)	29(5)
C(112)	5720(20)	5622(6)	9845(18)	26(5)
C(113)	5490(20)	5378(5)	8706(16)	20(4)
C(114)	5580(18)	5595(5)	7571(15)	11(4)
C(115)	7400(20)	5854(5)	6185(17)	22(4)
C(116)	8980(20)	5977(5)	6344(16)	19(4)
C(117)	9270(20)	6204(5)	7430(16)	18(4)
C(118)	9481(18)	5984(5)	8576(16)	16(4)
C(119)	9700(20)	6017(6)	10671(17)	24(5)
C(120)	9830(20)	6278(5)	11704(17)	23(4)
C(121)	8680(20)	6570(6)	11810(18)	25(5)
C(122)	7170(20)	6409(6)	11720(18)	27(5)
C(123)	8850(30)	6786(7)	12970(20)	41(6)
C(124)	11060(20)	5790(6)	10630(17)	26(5)
C(125)	12180(20)	5198(6)	11010(19)	33(5)
C(126)	12200(20)	4953(5)	9940(17)	21(4)
C(127)	11210(20)	4825(6)	7984(18)	25(5)
C(128)	11880(20)	5018(6)	6905(17)	29(5)
C(129)	11940(20)	4757(6)	5825(18)	33(5)
C(130)	12310(20)	4946(6)	4720(20)	32(5)
C(131)	11260(20)	5224(6)	4358(18)	30(5)
C(132)	8750(20)	5085(5)	3606(18)	23(4)
C(133)	7130(20)	5064(6)	4000(19)	30(5)
C(134)	6720(20)	4668(6)	4260(20)	35(5)
C(135)	9530(20)	4761(6)	7770(19)	28(5)
C(136)	9510(20)	4333(7)	9550(20)	47(7)
C(137)	7240(20)	5628(6)	5026(17)	21(4)
C(201)	8180(40)	9096(11)	7530(40)	90(11)
C(202)	9490(30)	9203(8)	7130(30)	59(7)
C(203)	10640(40)	9018(9)	7430(30)	73(9)
C(204)	10670(30)	8736(7)	8220(20)	43(6)
C(205)	9330(30)	8669(8)	8650(30)	52(7)
C(206)	8200(30)	8830(8)	8400(30)	59(8)
C(207)	12100(30)	8538(7)	8620(20)	42(6)
C(208)	12360(20)	8227(6)	7728(19)	28(5)
C(209)	11410(20)	7889(5)	7998(17)	22(4)
C(210)	12160(20)	7615(6)	6045(16)	26(5)

C(211)	11240(20)	7336(6)	5340(20)	35(5)
C(212)	10679(17)	7076(5)	6289(14)	10(3)
C(213)	10415(19)	7316(5)	7349(16)	20(4)
C(214)	10620(20)	7126(7)	8510(20)	39(6)
C(215)	12405(18)	6892(5)	9919(15)	14(4)
C(216)	12233(19)	7079(6)	11066(17)	22(4)
C(217)	12120(20)	7668(6)	12086(19)	30(5)
C(218)	11840(30)	8054(7)	11850(20)	48(7)
C(219)	13770(20)	7631(6)	12540(20)	36(6)
C(220)	16160(20)	7472(6)	11820(20)	31(5)
C(221)	17370(20)	7759(7)	11430(20)	36(6)
C(222)	17000(20)	7941(6)	10291(19)	34(5)
C(223)	16900(20)	7692(6)	9264(19)	32(5)
C(224)	16230(20)	7882(6)	8137(16)	23(4)
C(225)	17220(20)	7760(6)	6229(19)	33(5)
C(226)	17250(20)	7516(6)	5090(19)	28(5)
C(227)	16020(20)	6926(6)	5528(18)	27(5)
C(228)	14690(20)	6719(6)	5439(16)	22(4)
C(229)	14790(20)	6441(6)	4374(17)	25(5)
C(230)	13630(20)	6152(6)	4309(19)	29(5)
C(231)	13870(20)	5936(6)	3102(17)	26(5)
C(232)	12160(20)	6308(6)	4365(17)	28(5)
C(233)	14339(19)	6713(5)	7592(16)	17(4)
C(234)	14170(20)	6516(6)	8692(18)	34(5)
C(235)	13950(20)	6729(6)	9806(18)	28(5)
C(236)	14630(20)	7954(6)	8359(17)	23(4)
C(237)	14560(30)	8350(7)	6570(20)	39(6)
N(101)	8846(18)	4542(5)	8516(16)	34(5)
N(102)	6405(18)	5085(5)	8870(15)	30(4)
N(103)	11290(18)	5046(5)	9044(13)	24(4)
N(104)	10986(17)	5443(5)	11082(13)	25(4)
N(105)	9467(15)	6187(5)	9552(14)	26(4)
N(106)	6950(20)	5622(5)	7173(15)	34(5)
N(107)	6977(18)	5278(5)	5066(16)	30(4)
N(108)	9659(17)	5104(5)	4449(15)	29(4)
N(201)	13849(17)	8159(5)	7520(14)	25(4)
N(202)	16377(16)	7661(5)	7073(13)	23(4)
N(203)	16012(17)	7257(4)	5038(15)	26(4)
N(204)	14436(16)	6518(4)	6541(15)	20(4)

N(205)	12032(17)	7082(4)	8916(14)	20(4)
N(206)	11433(17)	7624(4)	7224(14)	21(4)
N(207)	11920(16)	7427(5)	11038(15)	26(4)
N(208)	14721(18)	7601(5)	11684(14)	27(4)
O(1W)	20080(30)	8292(8)	4700(30)	118(10)
O(3W)	15190(40)	4325(9)	11240(30)	125(10)
O(101)	13040(14)	4690(4)	9905(13)	34(4)
O(102)	12194(15)	5895(4)	10120(14)	37(4)
O(103)	4579(15)	5754(4)	7110(12)	28(3)
O(104)	7611(13)	5795(4)	4081(11)	23(3)
O(105)	9009(15)	5083(4)	2566(11)	29(3)
O(106)	5645(16)	4838(4)	7153(14)	37(4)
O(107)	8945(15)	4907(4)	6930(12)	32(4)
O(108)	9507(15)	5661(4)	8601(11)	26(3)
O(201)	12598(18)	6931(5)	12010(13)	39(4)
O(202)	14001(16)	7620(5)	13582(12)	35(4)
O(203)	9571(15)	6960(4)	8965(12)	32(4)
O(204)	10683(15)	7877(4)	8889(12)	28(3)
O(205)	13901(15)	7801(4)	9152(11)	30(4)
O(206)	14523(14)	7058(4)	7548(12)	28(3)
O(207)	18028(17)	8020(4)	6200(13)	37(4)
O(208)	17184(14)	6796(4)	6000(12)	25(3)

Table S19. Bond lengths [Å] and angles [°] for **32**.

Bond lengths [Å]	
C(101)-C(106)	1.27(5)
C(101)-C(102)	1.37(5)
C(102)-C(103)	1.46(5)
C(103)-C(104)	1.31(5)
C(104)-C(105)	1.35(4)
C(104)-C(107)	1.54(4)
C(105)-C(106)	1.46(5)
C(107)-C(108)	1.49(3)
C(108)-N(101)	1.46(3)
C(108)-C(109)	1.59(3)
C(109)-O(106)	1.26(2)
C(109)-N(102)	1.31(3)
C(110)-N(102)	1.48(3)

C(110)-C(111)	1.50(3)
C(111)-C(112)	1.55(3)
C(112)-C(113)	1.58(3)
C(113)-N(102)	1.38(3)
C(113)-C(114)	1.51(2)
C(114)-O(103)	1.21(2)
C(114)-N(106)	1.35(2)
C(115)-N(106)	1.47(3)
C(115)-C(116)	1.54(3)
C(115)-C(137)	1.56(3)
C(116)-C(117)	1.51(3)
C(117)-C(118)	1.54(3)
C(118)-O(108)	1.19(2)
C(118)-N(105)	1.33(3)
C(119)-N(105)	1.43(3)
C(119)-C(124)	1.51(3)
C(119)-C(120)	1.52(3)
C(120)-C(121)	1.52(3)
C(121)-C(122)	1.52(3)
C(121)-C(123)	1.54(3)
C(124)-O(102)	1.26(2)
C(124)-N(104)	1.38(3)
C(125)-N(104)	1.43(3)
C(125)-C(126)	1.51(3)
C(126)-O(101)	1.24(2)
C(126)-N(103)	1.36(3)
C(127)-N(103)	1.45(3)
C(127)-C(128)	1.54(3)
C(127)-C(135)	1.59(3)
C(128)-C(129)	1.56(3)
C(129)-C(130)	1.47(3)
C(130)-C(131)	1.47(3)
C(131)-N(108)	1.55(2)
C(132)-O(105)	1.20(2)
C(132)-N(108)	1.27(3)
C(132)-C(133)	1.57(3)
C(133)-N(107)	1.45(3)
C(133)-C(134)	1.53(3)
C(135)-O(107)	1.22(2)

C(135)-N(101)	1.33(3)
C(136)-N(101)	1.53(3)
C(137)-O(104)	1.28(2)
C(137)-N(107)	1.31(3)
C(201)-C(202)	1.35(5)
C(201)-C(206)	1.38(5)
C(202)-C(203)	1.31(4)
C(203)-C(204)	1.36(4)
C(204)-C(205)	1.36(4)
C(204)-C(207)	1.58(4)
C(205)-C(206)	1.24(4)
C(207)-C(208)	1.54(3)
C(208)-N(201)	1.42(3)
C(208)-C(209)	1.55(3)
C(209)-O(204)	1.22(2)
C(209)-N(206)	1.31(3)
C(210)-N(206)	1.50(2)
C(210)-C(211)	1.55(3)
C(211)-C(212)	1.53(3)
C(212)-C(213)	1.51(2)
C(213)-N(206)	1.48(2)
C(213)-C(214)	1.50(3)
C(214)-O(203)	1.26(3)
C(214)-N(205)	1.39(3)
C(215)-N(205)	1.38(2)
C(215)-C(216)	1.48(3)
C(215)-C(235)	1.56(2)
C(216)-O(201)	1.24(2)
C(216)-N(207)	1.31(3)
C(217)-C(218)	1.47(3)
C(217)-N(207)	1.49(3)
C(217)-C(219)	1.62(3)
C(219)-O(202)	1.20(3)
C(219)-N(208)	1.31(3)
C(220)-N(208)	1.42(3)
C(220)-C(221)	1.60(3)
C(221)-C(222)	1.49(3)
C(222)-C(223)	1.48(3)
C(223)-C(224)	1.58(3)

C(224)-N(202)	1.46(3)
C(224)-C(236)	1.53(3)
C(225)-O(207)	1.21(3)
C(225)-N(202)	1.29(3)
C(225)-C(226)	1.57(3)
C(226)-N(203)	1.49(3)
C(227)-O(208)	1.29(3)
C(227)-N(203)	1.34(3)
C(227)-C(228)	1.45(3)
C(228)-N(204)	1.47(2)
C(228)-C(229)	1.58(3)
C(229)-C(230)	1.51(3)
C(230)-C(232)	1.48(3)
C(230)-C(231)	1.60(3)
C(233)-O(206)	1.28(2)
C(233)-N(204)	1.39(2)
C(233)-C(234)	1.45(3)
C(234)-C(235)	1.50(3)
C(236)-O(205)	1.25(2)
C(236)-N(201)	1.41(3)
C(237)-N(201)	1.44(2)

Angles [°]

C(106)-C(101)-C(102)	126(4)
C(101)-C(102)-C(103)	114(4)
C(104)-C(103)-C(102)	124(4)
C(103)-C(104)-C(105)	117(4)
C(103)-C(104)-C(107)	124(3)
C(105)-C(104)-C(107)	119(3)
C(104)-C(105)-C(106)	122(4)
C(101)-C(106)-C(105)	116(4)
C(108)-C(107)-C(104)	120(2)
N(101)-C(108)-C(107)	114.5(19)
N(101)-C(108)-C(109)	111.6(17)
C(107)-C(108)-C(109)	115.1(18)
O(106)-C(109)-N(102)	122.3(18)
O(106)-C(109)-C(108)	117.4(17)
N(102)-C(109)-C(108)	119.8(18)
N(102)-C(110)-C(111)	103.9(16)
C(110)-C(111)-C(112)	103.3(16)

C(111)-C(112)-C(113)	102.4(16)
N(102)-C(113)-C(114)	119.4(15)
N(102)-C(113)-C(112)	104.6(16)
C(114)-C(113)-C(112)	112.7(16)
O(103)-C(114)-N(106)	122.5(17)
O(103)-C(114)-C(113)	125.2(15)
N(106)-C(114)-C(113)	112.0(16)
N(106)-C(115)-C(116)	111.0(16)
N(106)-C(115)-C(137)	107.5(16)
C(116)-C(115)-C(137)	110.5(15)
C(117)-C(116)-C(115)	115.5(16)
C(116)-C(117)-C(118)	114.7(16)
O(108)-C(118)-N(105)	122.6(18)
O(108)-C(118)-C(117)	123.2(18)
N(105)-C(118)-C(117)	113.9(17)
N(105)-C(119)-C(124)	109.9(15)
N(105)-C(119)-C(120)	114.7(18)
C(124)-C(119)-C(120)	108.1(17)
C(119)-C(120)-C(121)	117.0(17)
C(120)-C(121)-C(122)	111.5(17)
C(120)-C(121)-C(123)	111.1(17)
C(122)-C(121)-C(123)	110.5(17)
O(102)-C(124)-N(104)	119.5(19)
O(102)-C(124)-C(119)	122.8(19)
N(104)-C(124)-C(119)	117.4(16)
N(104)-C(125)-C(126)	115.8(19)
O(101)-C(126)-N(103)	124.0(17)
O(101)-C(126)-C(125)	120.0(18)
N(103)-C(126)-C(125)	115.9(17)
N(103)-C(127)-C(128)	112.2(17)
N(103)-C(127)-C(135)	104.9(15)
C(128)-C(127)-C(135)	109.9(17)
C(127)-C(128)-C(129)	110.7(18)
C(130)-C(129)-C(128)	113(2)
C(131)-C(130)-C(129)	114.3(18)
C(130)-C(131)-N(108)	114.6(18)
O(105)-C(132)-N(108)	126.9(19)
O(105)-C(132)-C(133)	118.1(18)
N(108)-C(132)-C(133)	115.0(18)

N(107)-C(133)-C(134)	109.4(17)
N(107)-C(133)-C(132)	107.7(17)
C(134)-C(133)-C(132)	109.9(18)
O(107)-C(135)-N(101)	123(2)
O(107)-C(135)-C(127)	119.2(19)
N(101)-C(135)-C(127)	117.4(18)
O(104)-C(137)-N(107)	123.4(19)
O(104)-C(137)-C(115)	114.7(17)
N(107)-C(137)-C(115)	120.9(17)
C(202)-C(201)-C(206)	116(4)
C(203)-C(202)-C(201)	119(3)
C(202)-C(203)-C(204)	126(3)
C(205)-C(204)-C(203)	111(3)
C(205)-C(204)-C(207)	125(2)
C(203)-C(204)-C(207)	124(3)
C(206)-C(205)-C(204)	127(3)
C(205)-C(206)-C(201)	120(3)
C(208)-C(207)-C(204)	106.5(19)
N(201)-C(208)-C(207)	113.1(18)
N(201)-C(208)-C(209)	116.1(17)
C(207)-C(208)-C(209)	112.0(17)
O(204)-C(209)-N(206)	122.4(18)
O(204)-C(209)-C(208)	120.5(18)
N(206)-C(209)-C(208)	117.1(17)
N(206)-C(210)-C(211)	102.9(16)
C(212)-C(211)-C(210)	104.0(17)
C(213)-C(212)-C(211)	104.3(15)
N(206)-C(213)-C(214)	111.0(17)
N(206)-C(213)-C(212)	105.5(13)
C(214)-C(213)-C(212)	113.8(17)
O(203)-C(214)-N(205)	123(2)
O(203)-C(214)-C(213)	119(2)
N(205)-C(214)-C(213)	117.3(17)
N(205)-C(215)-C(216)	117.4(16)
N(205)-C(215)-C(235)	110.9(15)
C(216)-C(215)-C(235)	110.6(15)
O(201)-C(216)-N(207)	120.5(19)
O(201)-C(216)-C(215)	121.3(18)
N(207)-C(216)-C(215)	117.2(18)

C(218)-C(217)-N(207)	114.2(19)
C(218)-C(217)-C(219)	107.8(19)
N(207)-C(217)-C(219)	108.6(17)
O(202)-C(219)-N(208)	127(2)
O(202)-C(219)-C(217)	118.7(17)
N(208)-C(219)-C(217)	114(2)
N(208)-C(220)-C(221)	114.1(18)
C(222)-C(221)-C(220)	111.6(18)
C(223)-C(222)-C(221)	115(2)
C(222)-C(223)-C(224)	112.6(18)
N(202)-C(224)-C(236)	109.0(15)
N(202)-C(224)-C(223)	112.4(17)
C(236)-C(224)-C(223)	109.0(15)
O(207)-C(225)-N(202)	128(2)
O(207)-C(225)-C(226)	114.5(19)
N(202)-C(225)-C(226)	117(2)
N(203)-C(226)-C(225)	112.3(17)
O(208)-C(227)-N(203)	120.7(19)
O(208)-C(227)-C(228)	122.9(18)
N(203)-C(227)-C(228)	116.2(19)
C(227)-C(228)-N(204)	110.0(17)
C(227)-C(228)-C(229)	109.9(15)
N(204)-C(228)-C(229)	109.4(16)
C(230)-C(229)-C(228)	116.6(17)
C(232)-C(230)-C(229)	112.3(18)
C(232)-C(230)-C(231)	111.3(16)
C(229)-C(230)-C(231)	106.8(17)
O(206)-C(233)-N(204)	118.0(16)
O(206)-C(233)-C(234)	122.9(18)
N(204)-C(233)-C(234)	118.9(17)
C(233)-C(234)-C(235)	118(2)
C(234)-C(235)-C(215)	113.3(18)
O(205)-C(236)-N(201)	116.6(17)
O(205)-C(236)-C(224)	124.2(18)
N(201)-C(236)-C(224)	118.5(16)
C(135)-N(101)-C(108)	118.5(17)
C(135)-N(101)-C(136)	126.9(17)
C(108)-N(101)-C(136)	114.5(17)
C(109)-N(102)-C(113)	118.7(18)

C(109)-N(102)-C(110)	127.1(18)
C(113)-N(102)-C(110)	113.9(17)
C(126)-N(103)-C(127)	120.3(16)
C(124)-N(104)-C(125)	121.8(17)
C(118)-N(105)-C(119)	119.3(18)
C(114)-N(106)-C(115)	124.8(18)
C(137)-N(107)-C(133)	119.2(18)
C(132)-N(108)-C(131)	126.7(18)
C(236)-N(201)-C(208)	118.5(15)
C(236)-N(201)-C(237)	121.7(17)
C(208)-N(201)-C(237)	119.0(17)
C(225)-N(202)-C(224)	120.8(19)
C(227)-N(203)-C(226)	124.0(18)
C(233)-N(204)-C(228)	118.5(15)
C(215)-N(205)-C(214)	124.3(16)
C(209)-N(206)-C(213)	119.7(15)
C(209)-N(206)-C(210)	128.3(16)
C(213)-N(206)-C(210)	110.8(15)
C(216)-N(207)-C(217)	122.1(18)
C(219)-N(208)-C(220)	126(2)

Table S20. Anisotropic displacement parameters (Å^{2}x 10^3) for **32**. The anisotropic displacement factor exponent takes the form: $-2\pi^2[h^2a^{*2}U^{11} + ... + 2hka^*b^*U^{12}]$

	U^{11}	U^{22}	U^{33}	U^{23}	U^{13}	U^{12}
C(101)	76(14)	73(14)	81(14)	-3(12)	1(12)	-9(11)
C(102)	85(15)	91(16)	92(16)	-12(12)	7(12)	6(12)
C(103)	83(15)	100(16)	89(16)	-7(12)	-8(12)	-11(12)
C(104)	75(13)	58(13)	79(14)	-11(11)	10(11)	-3(11)
C(105)	77(14)	83(15)	70(14)	5(12)	8(11)	-6(11)
C(106)	70(14)	85(15)	84(15)	7(12)	-2(12)	-6(12)
C(107)	37(10)	24(9)	38(10)	-4(8)	9(8)	2(8)
C(108)	34(10)	37(10)	37(10)	10(9)	-12(8)	2(8)
C(109)	19(8)	17(9)	21(9)	-3(8)	-10(7)	-5(7)
C(110)	23(9)	29(9)	23(9)	6(8)	5(7)	4(7)
C(111)	27(9)	37(10)	24(9)	11(8)	4(8)	-5(8)
C(112)	27(9)	26(9)	24(9)	4(8)	-3(7)	-10(7)
C(113)	21(8)	25(9)	14(8)	-1(7)	3(7)	2(7)

C(114)	11(4)	11(4)	10(4)	0(1)	0(1)	0(1)
C(115)	20(8)	24(9)	21(9)	11(8)	-7(7)	5(7)
C(116)	24(6)	21(7)	12(6)	8(5)	0(5)	0(5)
C(117)	20(8)	18(9)	15(8)	1(7)	-3(7)	6(7)
C(118)	8(7)	21(9)	18(8)	2(7)	5(6)	1(6)
C(119)	18(8)	31(10)	22(9)	4(8)	6(7)	-11(7)
C(120)	27(9)	23(9)	19(8)	5(8)	3(7)	-10(7)
C(121)	25(9)	34(10)	17(8)	-3(8)	-2(7)	3(8)
C(122)	19(9)	39(12)	22(10)	-7(9)	-6(8)	-6(8)
C(123)	48(13)	45(14)	29(12)	2(11)	1(10)	9(11)
C(124)	31(9)	29(9)	18(8)	-8(8)	16(7)	-13(8)
C(125)	31(9)	37(10)	31(9)	6(9)	10(8)	7(8)
C(126)	17(8)	27(9)	19(8)	-11(8)	0(7)	-1(7)
C(127)	20(8)	29(9)	28(9)	-4(8)	-1(7)	4(7)
C(128)	34(9)	34(10)	18(8)	-7(8)	3(8)	1(8)
C(129)	29(9)	43(11)	25(9)	-6(9)	-6(8)	9(8)
C(130)	24(9)	41(10)	32(10)	2(9)	1(8)	-6(8)
C(131)	29(9)	40(10)	20(9)	-7(8)	5(8)	-12(8)
C(132)	23(8)	20(9)	25(9)	-1(8)	-7(7)	4(7)
C(133)	24(9)	35(10)	30(9)	2(8)	1(8)	-5(8)
C(134)	29(11)	46(13)	30(11)	3(10)	8(9)	-20(10)
C(135)	30(9)	27(10)	26(9)	-8(8)	-7(8)	8(8)
C(136)	22(11)	50(14)	68(16)	19(13)	11(11)	3(10)
C(137)	22(6)	21(7)	19(7)	4(6)	-7(5)	5(5)
C(201)	89(15)	89(16)	92(16)	3(12)	6(12)	1(12)
C(202)	45(11)	58(12)	73(13)	15(11)	6(10)	14(10)
C(203)	75(14)	77(14)	68(14)	14(11)	7(11)	1(11)
C(204)	47(11)	35(11)	48(11)	-1(10)	1(9)	3(9)
C(205)	47(11)	57(12)	52(12)	4(10)	3(10)	1(10)
C(206)	58(12)	54(12)	65(13)	2(11)	1(10)	-3(10)
C(207)	37(10)	43(11)	46(11)	-11(9)	3(9)	-5(9)
C(208)	26(9)	31(10)	29(9)	7(8)	-12(8)	1(8)
C(209)	29(9)	16(9)	22(9)	3(8)	1(8)	1(7)
C(210)	36(9)	27(9)	16(8)	5(8)	5(7)	-3(8)
C(211)	33(9)	35(10)	38(10)	11(9)	9(8)	-10(8)
C(212)	9(6)	10(6)	11(6)	3(5)	-4(5)	1(5)
C(213)	8(7)	32(9)	19(8)	3(8)	7(7)	4(7)
C(214)	35(10)	50(11)	31(10)	-7(9)	10(8)	-3(9)
C(215)	10(7)	15(8)	16(8)	-4(7)	-3(6)	4(6)

C(216)	14(8)	31(10)	20(9)	1(8)	8(7)	5(7)
C(217)	24(9)	41(10)	25(9)	1(9)	-6(8)	-1(8)
C(218)	52(14)	56(15)	36(13)	-17(12)	-2(11)	-6(12)
C(219)	31(10)	34(10)	44(11)	-3(9)	21(9)	6(8)
C(220)	21(9)	36(10)	37(10)	1(9)	16(8)	4(8)
C(221)	35(10)	44(11)	30(10)	-1(9)	-4(8)	-11(8)
C(222)	23(9)	44(11)	35(10)	-2(9)	1(8)	-20(8)
C(223)	28(9)	34(10)	35(10)	-7(9)	8(8)	11(8)
C(224)	27(9)	27(9)	16(8)	3(8)	-10(7)	-7(7)
C(225)	38(10)	36(10)	25(9)	4(8)	-6(8)	9(9)
C(226)	23(9)	33(10)	29(9)	-8(8)	4(8)	0(7)
C(227)	31(9)	27(9)	23(9)	2(8)	20(8)	0(8)
C(228)	23(8)	28(9)	13(8)	0(7)	-3(7)	7(7)
C(229)	26(9)	29(9)	19(8)	-5(8)	-16(7)	-10(7)
C(230)	32(9)	32(10)	23(9)	-1(8)	-18(8)	4(8)
C(231)	28(9)	33(10)	17(9)	-3(8)	-6(7)	-17(8)
C(232)	24(10)	45(13)	16(9)	-7(9)	11(8)	3(9)
C(233)	16(8)	19(9)	15(8)	2(7)	-2(7)	-12(7)
C(234)	38(10)	38(10)	26(9)	2(9)	-2(8)	0(8)
C(235)	27(9)	32(10)	25(9)	1(8)	1(8)	5(8)
C(236)	23(9)	28(9)	18(8)	4(8)	-5(7)	-7(7)
C(237)	36(12)	51(14)	29(11)	15(10)	21(10)	14(10)
N(101)	27(10)	37(11)	37(11)	21(9)	-13(8)	2(8)
N(102)	33(10)	30(10)	27(10)	10(9)	8(8)	-5(8)
N(103)	37(8)	22(8)	12(7)	-5(7)	6(6)	15(7)
N(104)	16(8)	48(12)	10(8)	7(8)	0(6)	3(8)
N(105)	8(8)	53(12)	15(8)	9(8)	-3(6)	-8(7)
N(106)	38(11)	47(12)	17(9)	-5(9)	14(8)	-3(9)
N(107)	25(9)	37(11)	29(10)	1(9)	0(8)	2(8)
N(108)	17(7)	47(9)	23(8)	0(8)	-7(6)	2(7)
N(201)	30(8)	25(8)	20(8)	18(7)	7(6)	1(7)
N(202)	19(8)	37(10)	13(8)	1(8)	2(7)	-3(7)
N(203)	19(9)	25(10)	35(10)	0(8)	1(7)	6(7)
N(204)	17(8)	9(8)	35(10)	0(7)	13(7)	3(6)
N(205)	24(6)	15(6)	20(6)	4(5)	-2(5)	-11(5)
N(206)	30(8)	15(7)	19(7)	10(7)	3(6)	2(6)
N(207)	14(7)	36(9)	29(8)	2(7)	-4(6)	-7(6)
N(208)	30(10)	33(10)	18(9)	-3(8)	17(7)	0(8)
O(101)	15(7)	52(10)	34(9)	-2(8)	-2(6)	13(7)

O(102)	29(8)	38(9)	43(10)	11(8)	14(7)	3(7)
O(103)	34(8)	22(8)	27(8)	10(6)	10(6)	-8(6)
O(104)	11(6)	41(9)	17(7)	14(6)	1(5)	-12(6)
O(105)	39(9)	36(9)	12(7)	8(7)	-17(6)	-10(7)
O(106)	36(9)	36(9)	39(10)	-15(8)	4(7)	1(7)
O(107)	32(8)	40(9)	23(8)	15(7)	0(6)	7(7)
O(108)	42(9)	19(8)	16(7)	2(6)	2(6)	-6(6)
O(201)	47(10)	44(10)	26(8)	12(8)	-4(7)	-6(8)
O(202)	39(9)	48(10)	18(8)	4(8)	-7(6)	0(7)
O(203)	28(8)	52(10)	15(7)	-5(7)	-8(6)	-22(7)
O(204)	31(8)	31(8)	22(8)	3(7)	10(6)	2(6)
O(205)	29(8)	49(10)	12(7)	1(7)	11(6)	-2(7)
O(206)	21(7)	41(10)	22(8)	9(7)	3(6)	4(6)
O(207)	43(10)	39(10)	28(8)	-3(7)	-8(7)	-16(7)
O(208)	23(7)	24(8)	28(8)	5(7)	1(6)	-1(6)

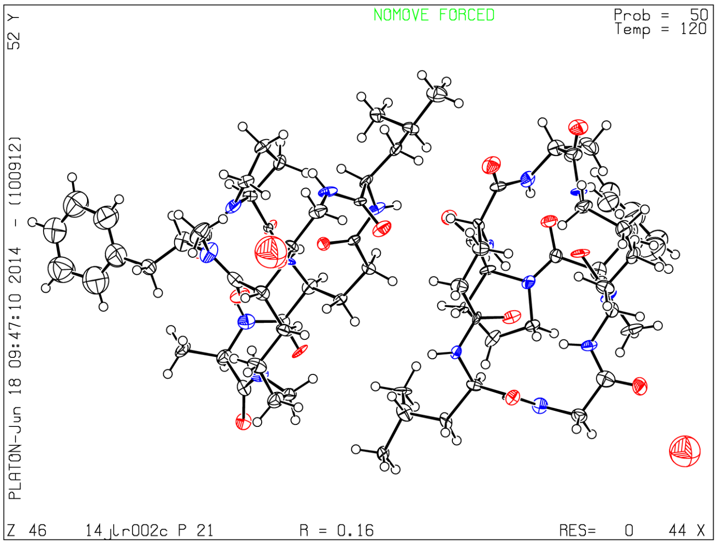

Comparing BBP with α-helices

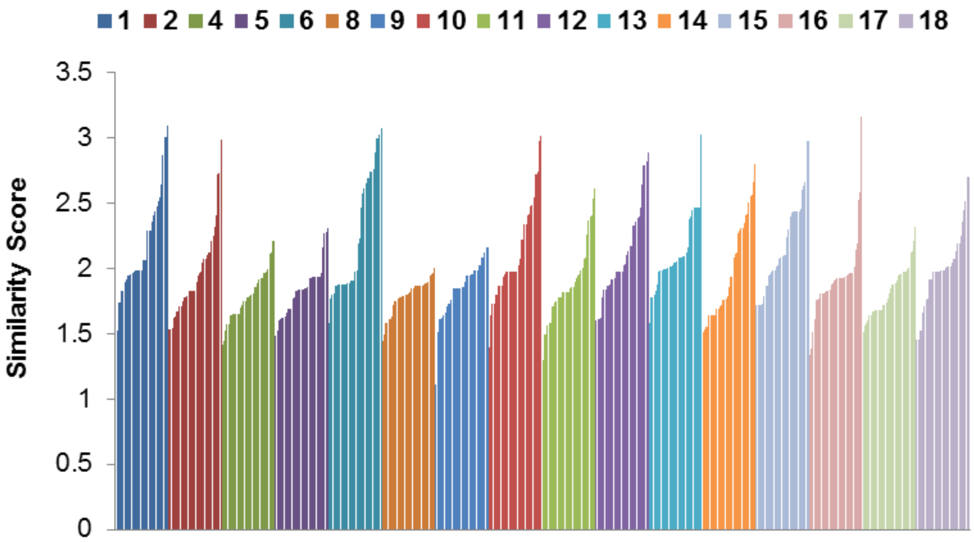

Figure S1. Similarity scores from 35 residue triplets from α-helix and 18 norbornapeptides. BBP **10**, **12**, **13** and **15** uses X-ray structures. BBP **16** uses the *s-cis* conformation which is the majority conformation in the solution.

Distance constraints from NMR

Table S21. H-H distances obtained with ROESY experiments.

1

Atom A	Atom B	Upper Distance Restraint (Å)
**LEU-NH**	LEU-Hα	2.3
**LEU-Hα**	GLY-NH	2.2
**GLY-NH**	GLY-Hα	2.5
**GLY-Hα**	LYS-NH	3.4
**GLY-NH**	LYS-NH	2.3
**LYS-NH**	LYS-Hα	2.6
**LYS-NHZ**	ALA-Hα	3.0
**PHE-NH**	PHE-Hα	2.0

2

Atom A	Atom B	Upper Distance Restraint (Å)
**GLY-NH**	LYS-NH	2.5
**PHE-NH**	GLU-NH	3.5
**PHE-NH**	ALA-NH	3.9
**GLY-NH**	LEU-NH	3.3
**LEU-NH**	LYS-NH	3.6
**ALA-NH**	GLU-NH	2.8
**ALA-NH**	LYS-NHZ	2.8
**PHE-NH**	LYS-Hα	2.3
**PHE-NH**	PHE-Hα	2.1
**GLY-NH**	GLY-Hα	2.4
**GLY-NH**	LEU-Hα	2.2
**ALA-NH**	ALA-Hα	2.3
**ALA-NH**	GLU-Hα	2.4
**ALA-NH**	PRO-Hα	3.8
**LYS-NHZ**	ALA-Hα	2.4
**GLU-NH**	GLU-Hα	2.7
**GLU-NH**	PRO-Hα	3.0
**GLU-NH**	PHE-Hα	3.6
**LYS-NH**	LEU-Hα	3.6
**LYS-NH**	LYS-Hα	2.6

4

Atom A	Atom B	Upper Distance Restraint (Å)
**GLY-NH**	LYS-NH	2.5
**LYS-NHZ**	ALA-NH	3.2
**GLY-NH**	GLY-Hα	2.5
**GLY-NH**	LEU-Hα	2.2
**LEU-NH**	LEU-Hα	2.8
**ALA-NH**	GLU-Hα	3.3
**GLU-NH**	GLU-Hα	2.8
**LYS-NH**	GLY-Hα	3.3
**LYS-NH**	LYS-Hα	2.8

**LYS-NHZ**	ALA-Hα	2.7

5

Atom A	Atom B	Upper Distance Restraint (Å)
**GLY-NH**	LYS-NH	2.3
**ALA-NH**	LYS-NHZ	2.6
**GLY-NH**	LEU-NH	2.9
**GLY-NH**	GLY-Hα	2.1
**GLY-NH**	LEU-Hα	1.9
**GLU-NH**	PRO-Hα	2.8
**GLU-NH**	GLU-Hα	2.4
**GLU-NH**	PHE-Hα	2.1
**ALA-NH**	ALA-Hα	2.4
**ALA-NH**	GLU-Hα	2.0
**LYS-NHZ**	ALA-Hα	2.2
**LYS-NH**	LYS-Hα	2.3
**LYS-NH**	GLY-Hα	2.9

6

Atom A	Atom B	Upper Distance Restraint (Å)
**GLY-NH**	LYS-NH	2.2
**GLY-NH**	LEU-NH	2.8
**ALA-NH**	LYS-NHZ	2.5
**PHE-NH**	PHE-Hβ	2.2
**PHE-NH**	PHE-Hα	1.8
**GLY-NH**	LEU-Hα	1.9
**LEU-NH**	LEU-Hα	2.4
**ALA-NH**	ALA-Hα	2.1
**ALA-NH**	GLU-Hα	2.1
**LYS-NHZ**	GLU-Hβ	2.8
**LYS-NHZ**	LYS-Hε	2.3
**LYS-NHZ**	ALA-Hα	2.2
**LYS-NH**	LYS-Hα	2.3

8

Atom A	Atom B	Upper Distance Restraint (Å)
**LYS-NH**	PHE-Hα	4.0
**LYS-Hα**	GLU-NH	3.7
**GLY-NH**	LYS-Hα	2.4
**LYS-NHZ**	ALA-NH	2.7
**PRO-Hα**	GLU-NH	3.0
**GLU-NH**	GLU-Hα	2.6
**PHE-Hα**	GLU-NH	2.3
**GLY-NH**	GLY-Hα	2.3
**LEU-NH**	LEU-Hα	2.0
**ALA-NH**	ALA-Hα	2.5
**GLU-Hα**	ALA-NH	2.2
**LYS-NHZ**	ALA-Hα	2.5
**GLY-Hα**	LYS-NH	2.7

**LYS-NH**	LYS-Hα	2.2
**PHE-Hα**	PRO-Hα	2.0

9

Atom A	Atom B	Upper Distance Restraint (Å)
**GLY-NH**	LYS-NH	2.6
**GLY-NH**	LEU-NH	3.0
**GLU-NH**	ALA-NH	2.9
**GLY-NH**	GLY-Hα	2.5
**GLY-NH**	LEU-Hα	2.6
**GLU-NH**	GLU-Hα	2.2
**PHE-NH**	LYS-Hα	2.4
**LEU-NH**	LEU-Hα	3.7
**LYS-NHZ**	LYS-Hε	2.6
**ALA-NH**	ALA-Hα	2.6
**LYS-NH**	GLY-Hα	3.0
**LYS-NH**	LYS-Hα	2.7

10

Atom A	Atom B	Upper Distance Restraint (Å)
**TYR-NH**	GLU-NH	3.6
**TYR-NH**	LYS-NH	2.8
**GLY-NH**	LYS-NH	2.2
**LYS-NH**	GLU-NH	3.0
**TYR-NH**	PRO-Hδ	2.9
**TYR-NH**	TYR-Hα	2.1
**TYR-NH**	LYS-Hα	2.0
**GLY-NH**	GLY-Hα	2.4
**GLY-NH**	LEU-Hα	2.3
**LEU-NH**	GLU-Hγ	2.2
**LEU-NH**	LEU-Hα	2.7
**LYS-NH**	GLY-Hα	3.3
**LYS-NH**	LYS-Hα	2.5
**ALA-NH**	LYS-Hε	3.2
**ALA-NH**	GLU-Hα	3.2
**ALA-NH**	ALA-Hα	2.6
**GLU-NH**	GLU-Hα	2.7
**GLU-NH**	ALA-Hα	2.9
**LYS-NHZ**	LYS-Hε	2.4
**LYS-NHZ**	ALA-Hα	3.0

11

Atom A	Atom B	Upper Distance Restraint (Å)
**GLY-NH**	LYS-NH	2.3
**GLY-NH**	LEU-NH	2.8
**ALA-NH**	GLU-NH	2.6
**ALA-NH**	LYS-NHZ	2.5
**GLY-NH**	GLY-Hα	2.2
**GLY-NH**	LEU-Hα	2.0

**TYR-NH**	TYR-Hα	1.8
**ALA-NH**	ALA-Hα	2.2
**ALA-NH**	GLU-Hα	2.1
**LEU-NH**	LEU-Hα	2.3
**LYS-NHZ**	LYS-Hε	2.3
**LYS-NHZ**	ALA-Hα	2.2
**GLU-NH**	PRO-Hδ	2.5
**GLU-NH**	GLU-Hα	2.5
**GLU-NH**	PRO-Hα	2.6
**LYS-NH**	PRO-Hδ	2.9
**LYS-NH**	GLY-Hα	2.9
**LYS-NH**	LYS-Hα	2.3
**TYR-Hα**	PRO-Hδ	2.0

12 (trans)

Atom A	Atom B	Upper Distance Restraint (Å)
**TYR-NH**	TYR-Hδ	2.6
**TYR-NH**	ALA-NH	3.6
**TYR-NH**	LYS-NH	2.8
**GLY-NH**	LYS-NH	2.2
**LYS-NH**	GLU-NH	2.9
**TYR-NH**	TYR-Hα	2.3
**TYR-NH**	LYS-Hα	1.9
**GLY-NH**	LEU-Hα	2.1
**GLY-NH**	GLY-Hα	2.5
**GLY-NH**	TYR-Hα	2.9
**LEU-NH**	LEU-Hα	2.7
**LYS-NH**	LEU-Hα	2.9
**LYS-NH**	GLY-Hα	2.9
**LYS-NH**	LYS-Hα	2.4
**GLU-NH**	GLU-Hα	2.4
**ALA-NH**	ALA-Hα	2.3
**LYS-NHZ**	ALA-Hα	2.8
**TYR-NH**	PRO-Hδ	2.6
**LEU-NH**	GLU-Hγ	2.3

12 (cis)

Atom A	Atom B	Upper Distance Restraint (Å)
**TYR-Hα**	PRO-Hα	1.8
**TYR-NH**	LYS-NH	2.6
**TYR-NH**	TYR-Hδ	2.5
**GLU-NH**	LYS-NH	2.8
**GLU-NH**	ALA-NH	2.2
**GLY-NH**	LYS-NH	2.0
**ALA-NH**	LYS-NHZ	2.0
**TYR-NH**	LYS-Hα	1.9
**TYR-NH**	TYR-Hα	2.1
**GLU-NH**	GLU-Hα	2.3
**GLU-NH**	LUE-Hα	2.9

**GLU-NH**	TYR-Hα	2.2
**GLY-NH**	LEU-Hα	1.7
**LEU-NH**	LEU-Hα	2.2
**ALA-NH**	GLU-Hα	2.5
**ALA-NH**	ALA-Hα	2.3
**ALA-NH**	TYR-Hα	2.3
**LYS-NHZ**	ALA-Hα	2.2
**TYR-Hδ**	PRO-Hα	2.5
**TYR-Hδ**	TYR-Hα	2.1
**LYS-NH**	LYS-Hα	2.3
**LEU-NH**	GLU-Hγ	2.3

13

Atom A	Atom B	Upper Distance Restraint (Å)
**GLY-NH**	LYS-NH	2.8
**TYR-NH**	TYR-Hα	2.8
**TYR-NH**	LYS-Hα	2.4
**GLY-NH**	LEU-Hα	2.6
**LEU-NH**	LEU-Hα	3.1
**GLU-NH**	GLU-Hα	2.3
**LYS-NHZ**	ALA-Hα	3.1

14

Atom A	Atom B	Upper Distance Restraint (Å)
**GLY-NH**	LYS-NH	2.4
**ALA-NH**	LYS-NHZ	2.7
**GLY-NH**	GLY-Hα	2.3
**GLY-NH**	LEU-Hα	2.0
**TYR-NH**	LYS-Hα	2.0
**GLU-NH**	PRO-Hδ	3.0
**GLU-NH**	GLU-Hα	2.5
**GLU-NH**	TYR-Hα	2.0
**ALA-NH**	ALA-Hα	2.5
**ALA-NH**	GLU-Hα	2.1
**LEU-NH**	LEU-Hα	2.5
**LYS-NHZ**	ALA-Hα	2.3
**LYS-NH**	GLY-Hα	2.9
**LYS-NH**	LYS-Hα	2.5
**TYR-Hα**	PRO-Hα	1.8

15

Atom A	Atom B	Upper Distance Restraint (Å)
**GLY-NH**	LYS-NH	1.9
**LEU-NH**	LYS-NH	2.3
**ALA-NH**	GLU-NH	2.4
**ALA-NH**	LYS-NHZ	2.4
**TYR-NH**	TYR-Hβ	2.0
**TYR-NH**	TYR-Hα	1.7
**GLY-NH**	GLY-Hα	1.8

**GLY-NH**	LEU-Hα	1.8
**LEU-NH**	GLU-Hβ	2.7
**LEU-NH**	GLU-Hγ	2.0
**LEU-NH**	LEU-Hα	2.1
**ALA-NH**	GLU-Hβ	2.3
**ALA-NH**	GLU-Hγ	2.6
**ALA-NH**	ALA-Hα	1.9
**ALA-NH**	GLU-Hα	1.9
**LYS-NHZ**	LYS-Hβ	2.5
**LYS-NHZ**	LYS-Hε	2.0
**LYS-NHZ**	ALA-Hα	2.0
**GLU-NH**	LEU-Hα	2.3
**GLU-NH**	PRO-Hα	2.3
**GLU-NH**	GLU-Hα	2.0
**LYS-NH**	LYS-Hα	1.7

16 (cis)

Atom A	Atom B	Upper Distance Restraint (Å)
**GLU-NH**	LYS-NH	3.3
**GLY-NH**	LYS-NH	3.0
**TYR-NH**	LYS-Hα	2.5
**GLU-NH**	TYR-Hα	2.1
**GLY-NH**	GLY-Hα	3.2
**GLY-NH**	LEU-Hα	2.7
**LEU-NH**	GLU-Hγ	2.7
**LEU-NH**	LEU-Hα	3.1
**TYR-Hδ**	TYR-Hα	2.3
**LYS-NH**	GLY-Hα	3.3
**LYS-NH**	LYS-Hα	2.6
**TYR-Hδ**	PRO-Hδ	2.8
**TYR-Hα**	PRO-Hα	2.0

16 (trans)

Atom A	Atom B	Upper Distance Restraint (Å)
**TYR-NH**	LYS-NHZ	3.4
**GLY-NH**	LYS-NH	3.1
**TYR-NH**	TYR-Hα	3.3
**TYR-NH**	LYS-Hα	2.5
**GLY-NH**	LEU-Hα	2.9
**LYS-NH**	LYS-Hα	2.8
**ALA-NH**	ALA-Hα	2.6
**GLU-NH**	GLU-Hα	2.6
**TYR-Hδ**	TYR-Hα	2.4

17

Atom A	Atom B	Upper Distance Restraint (Å)
**GLY-NH**	LYS-NH	2.3
**LYS-NHZ**	ALA-NH	2.7
**TYR-NH**	TYR-Hα	2.6

**TYR-NH**	LYS-Hα	2.4
**GLU-NH**	GLU-Hα	2.7
**GLY-NH**	LEU-Hα	2.2
**ALA-NH**	GLU-Hα	2.2
**LYS-NHZ**	ALA-Hα	2.6
**LYS-NH**	GLY-Hα	2.7
**TYR-Hα**	PRO-Hα	1.9

18

Atom A	Atom B	Upper Distance Restraint (Å)
**GLY-NH**	LYS-NH	2.5
**GLY-NH**	LEU-NH	2.8
**GLU-NH**	ALA-NH	2.8
**GLY-NH**	GLY-Hα	2.3
**GLY-NH**	LEU-Hα	2.3
**GLU-NH**	TYR-Hα	3.3
**GLU-NH**	GLU-Hα	2.1
**TYR-NH**	LYS-Hα	2.3
**TYR-NH**	TYR-Hα	2.6
**LEU-NH**	LEU-Hα	2.6
**LYS-NHZ**	ALA-Hα	2.9
**ALA-NH**	ALA-Hα	2.5
**LYS-NH**	GLY-Hα	2.9
**LYS-NH**	LYS-Hα	2.7
**LYS-NHZ**	LYS-Hε	2.5

27c

Atom A	Atom B	Upper Distance Restraint (Å)
**LYS-Hα**	TRP-HZ	2.8
**LYS-Hβ**	CYS-Hα	3.8
**GLU-Hγ**	LYS-Hα	3.0
**LYS-Hα**	TRP-Hε	2.9
**CYS-Hβ**	LYS-Hα	2.9
**TRP-Hε**	CYS-Hα	2.7

Synthesis and spectra of synthesized products

l^{1}G*k*^{2}fP*E*^{1}a^{2} (1).

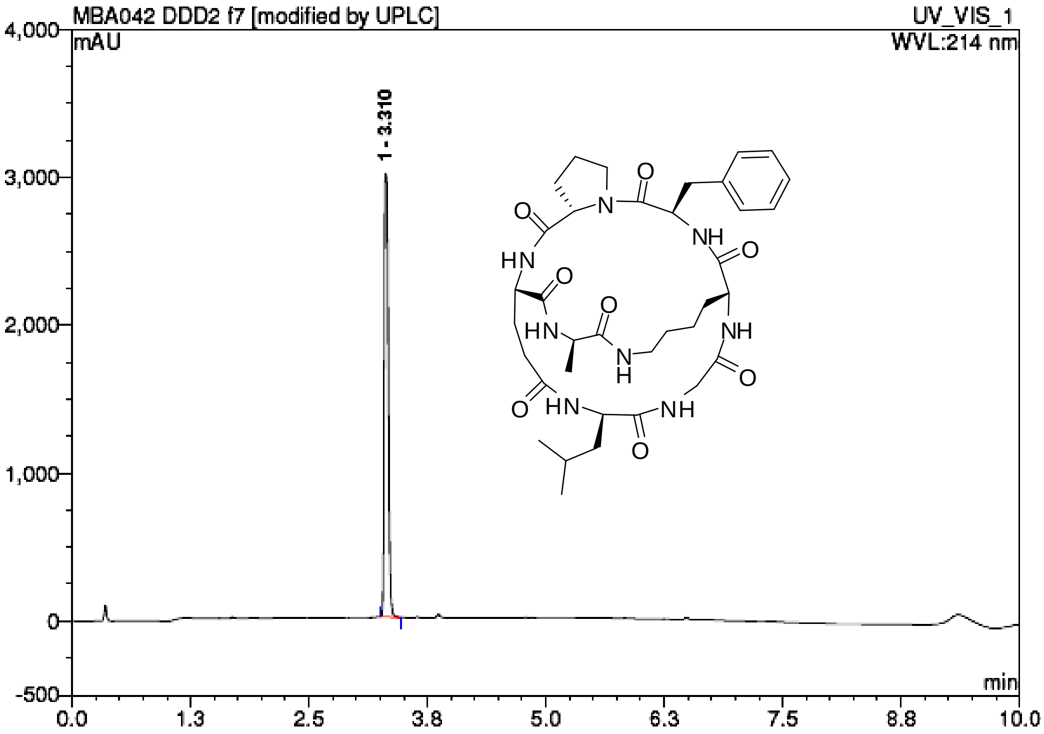

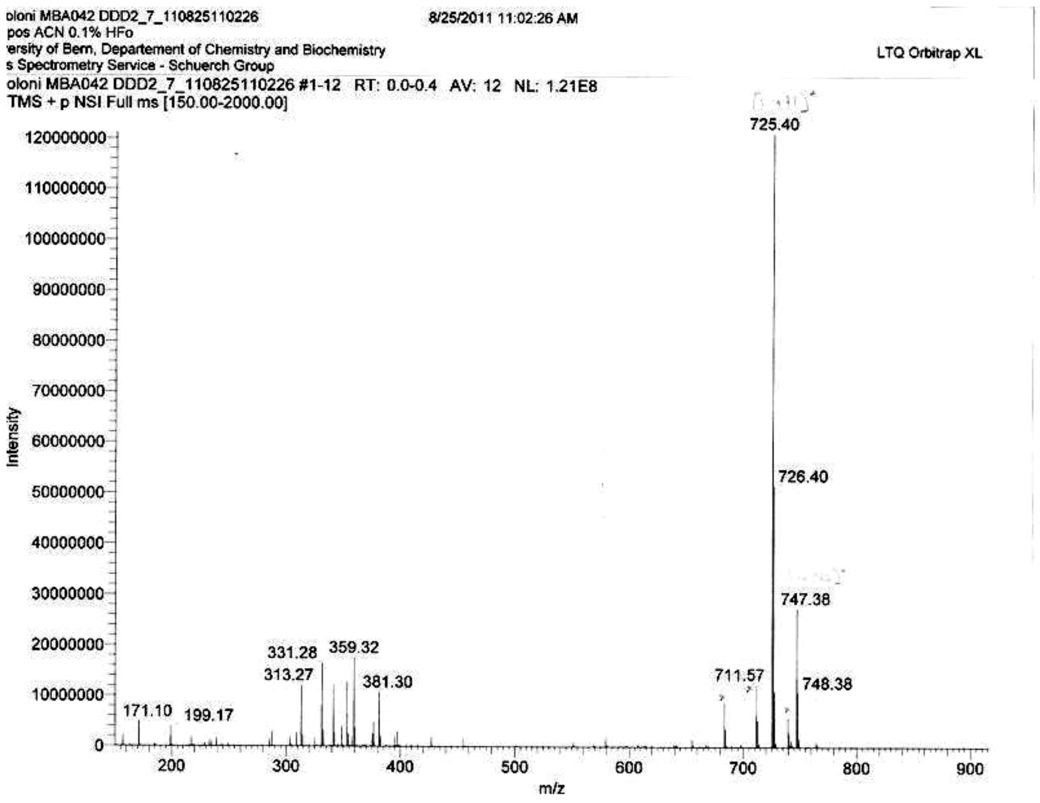

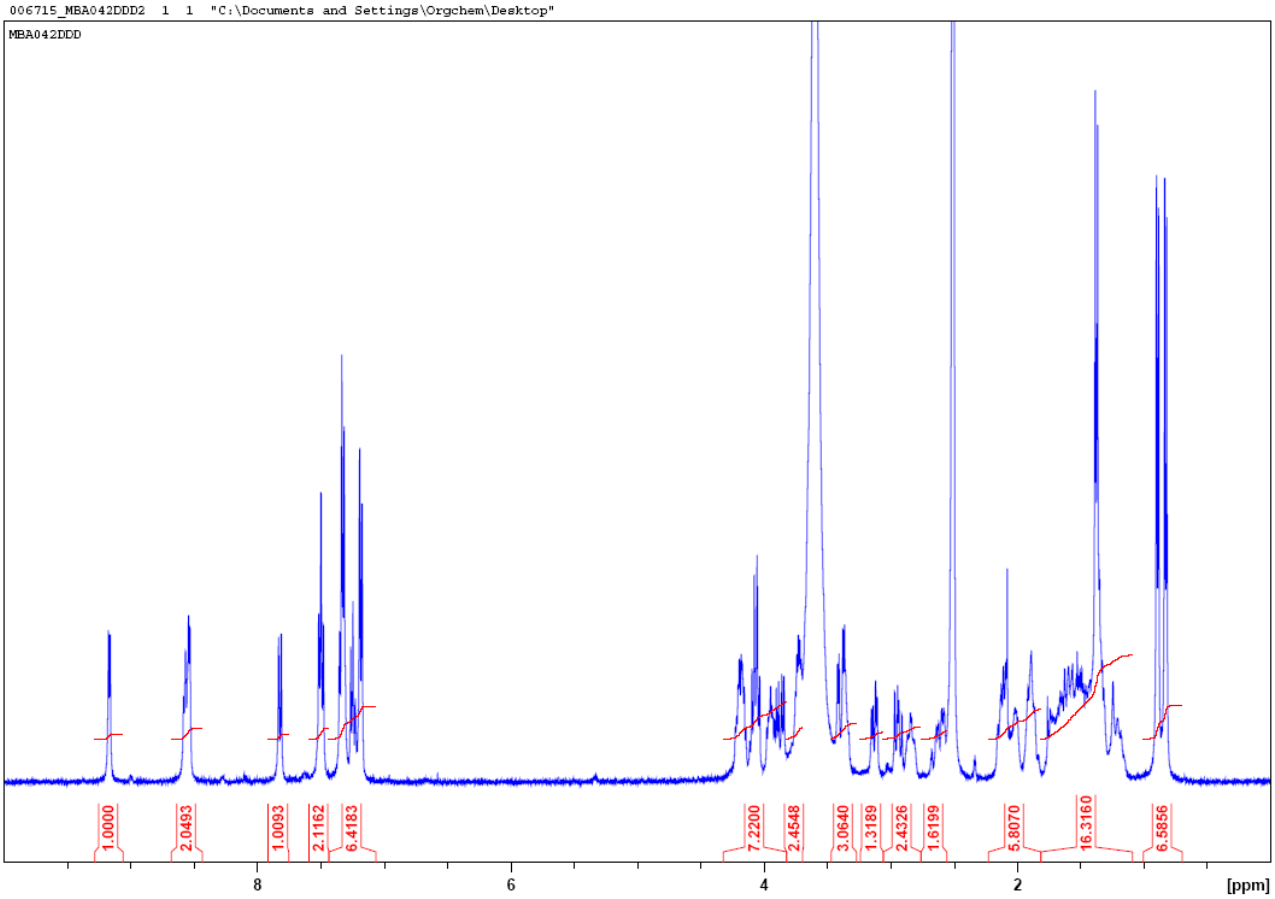

residue	proton	δ (ppm)	J (Hz)
^{1}H-NMR (400 MHz, DMSO-d_6)			
leu[1]	NH	8.54	^{3}J(NH–αCH) = 4.33
	αCH	3.74	
	β'CH	1.53	
	β"CH	1.36	
	γCH	1.66	
	δ'CH_3	0.90	
	δ"CH_3	0.83	
Gly[2]	NH	8.57	^{3}J(NH–α'CH) = 4.70
	α'CH	3.88	^{3}J(NH–α"CH) = 7.34
	α"CH	3.40	
lys[3]	αNH	7.82	^{3}J(NH–αCH) = 8.48
	αCH	4.21	
	βCH_2	1.44	
	γ'CH	1.49	
	γ"CH	1.21	
	δ'CH	1.58	
	δ"CH	1.35	
	ε'CH	3.57	
	ε"CH	2.84	
	εNH	7.18	
phe[4]	NH	9.17	^{3}J(NH–αCH) = 5.41
	αCH	4.17	
	β'CH	3.13	
	β"CH	2.96	
	2,6H	7.19	
	3,5H	7.34	
	4H	7.25	
Pro[5]	αCH	4.06	
	β'CH	2.11	
	β"CH	1.61	
	γ'CH	1.90	
	γ"CH	1.74	
	δ'CH	3.65	
	δ"CH	3.36	
Glu[6]	NH	7.49	^{3}J(NH–αCH) = 6.86
	αCH	3.95	
	β'CH	2.02	
	β"CH	1.90	
	γ'CH	2.62	
	γ"CH	2.13	
ala[7]	NH	7.51	^{3}J(NH–αCH) = 6.02
	αCH	4.09	
	βCH_3	1.38	

l^{1}G*k*^{2}fP*E*^{1}A^2 (2).

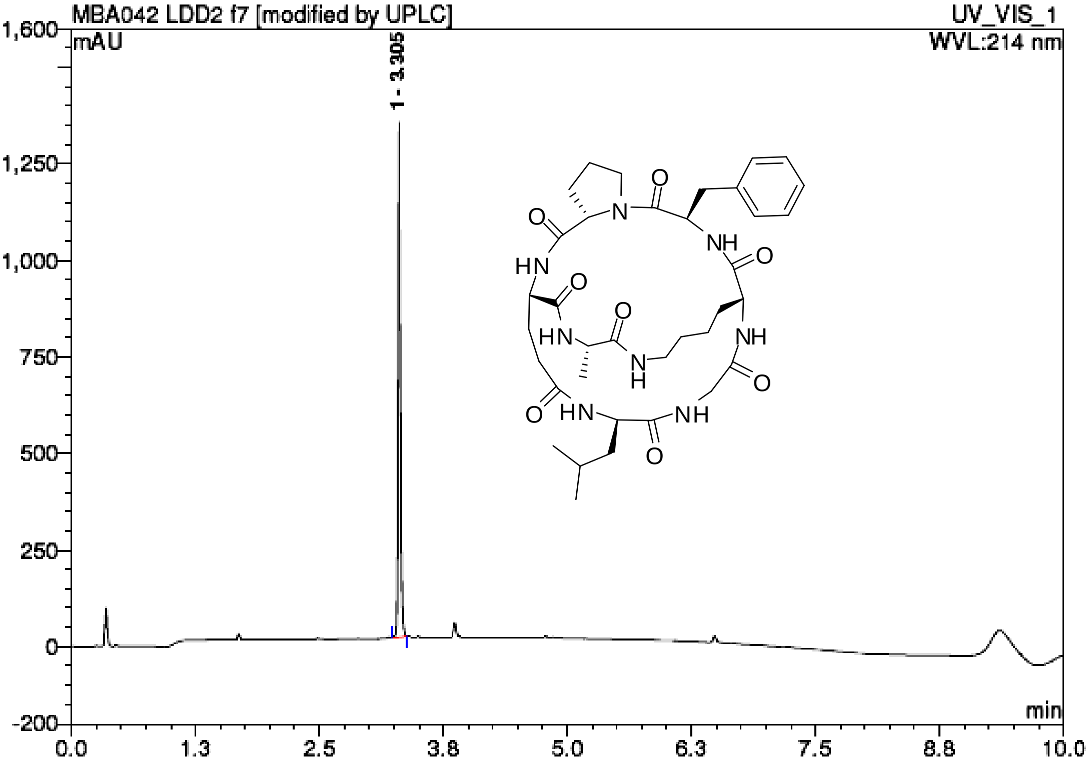

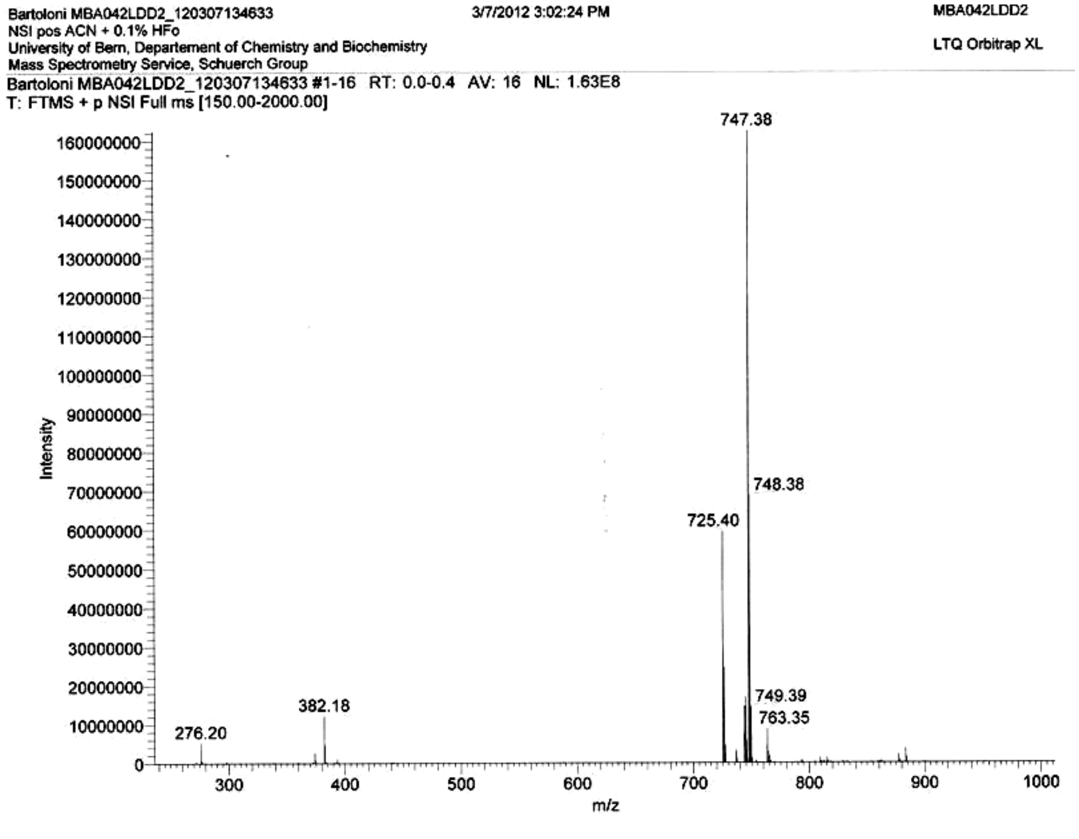

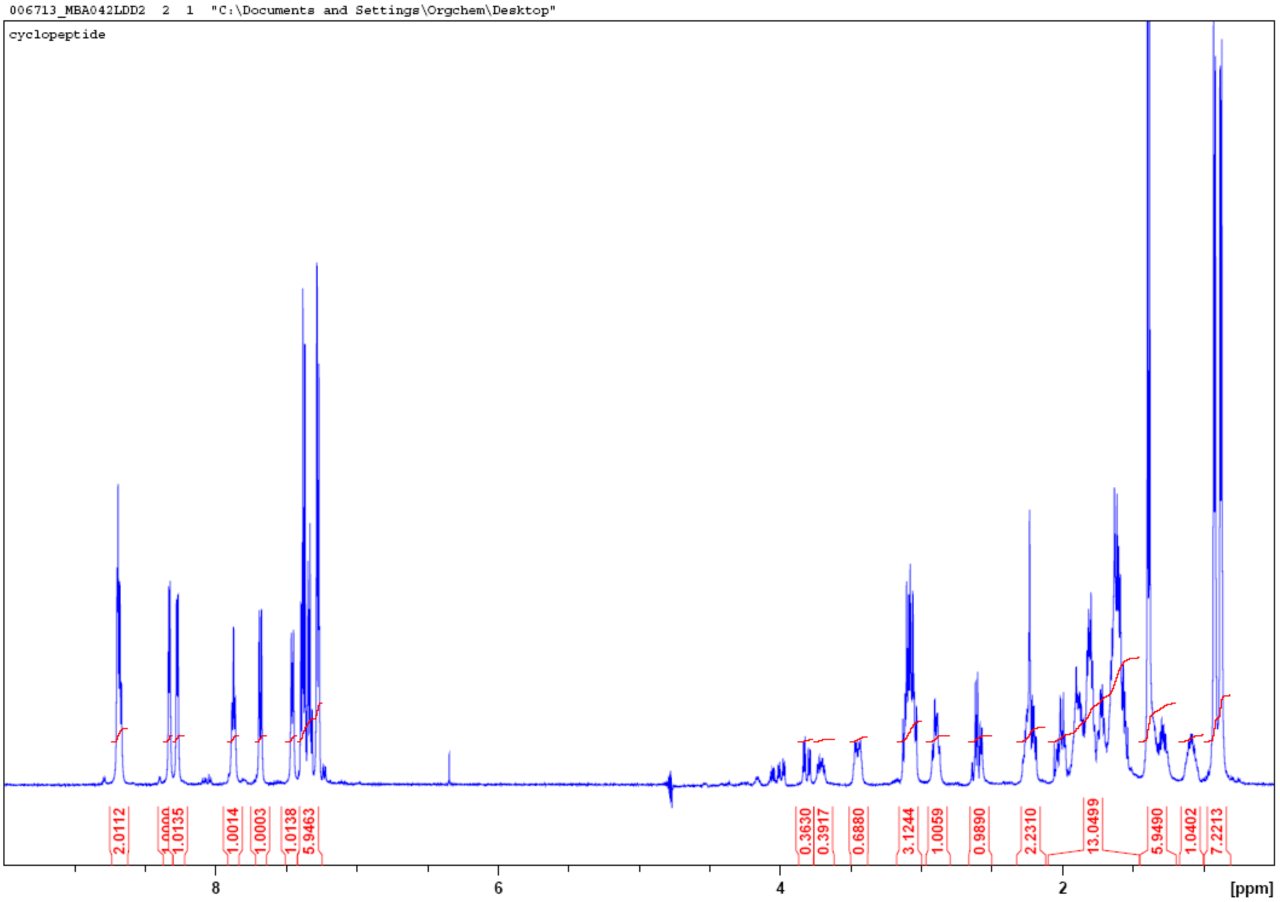

^{1}H-NMR (500 MHz, H_2O/D_2O 9:1)

residue	***proton***	***δ (ppm)***	***J (Hz)***
leu[1]	NH	8.34	^3J(NH–αCH) = 5.43
	αCH	4.17	
	$βCH_2$	1.61	
	γCH	1.61	
	δ'CH_3	0.93	
	δ"CH_3	0.88	
Gly[2]	NH	8.69	^3J(NH–α'CH) = 5.04
	α'CH	3.99	^3J(NH–α"CH) = 7.22
	α"CH	3.81	
lys[3]	αNH	7.48	^3J(NH–αCH) = 7.61
	αCH	4.42	
	β'CH	1.86	
	β"CH	1.64	
	γ'CH	1.29	
	γ"CH	1.09	
	δ'CH	1.62	
	δ"CH	1.38	
	ε'CH	3.44	
	ε"CH	3.08	^3J(NH–ε'CH) = 5.04
	εNH	7.89	^3J(NH–ε"CH) = 7.52
phe[4]	NH	8.71	^3J(NH–αCH) = 3.14
	αCH	4.53	
	$βCH_2$	3.09	
	2,6H	7.28	
	3,5H	7.38	
	4H	7.34	
Pro[5]	αCH	4.37	
	β'CH	1.80	
	β"CH	1.72	
	γ'CH	1.90	
	γ"CH	1.81	
	δ'CH	3.71	
	δ"CH	2.90	
Glu[6]	NH	7.70	^3J(NH–αCH) = 8.03
	αCH	4.26	
	β'CH	2.23	
	β"CH	2.01	
	γ'CH	2.59	
	γ"CH	2.21	
Ala[7]	NH	8.28	^3J(NH–αCH) = 5.17
	αCH	4.06	
	$βCH_3$	1.39	

l[1]G*k*[2]FP*E*[1]a[2] (3).

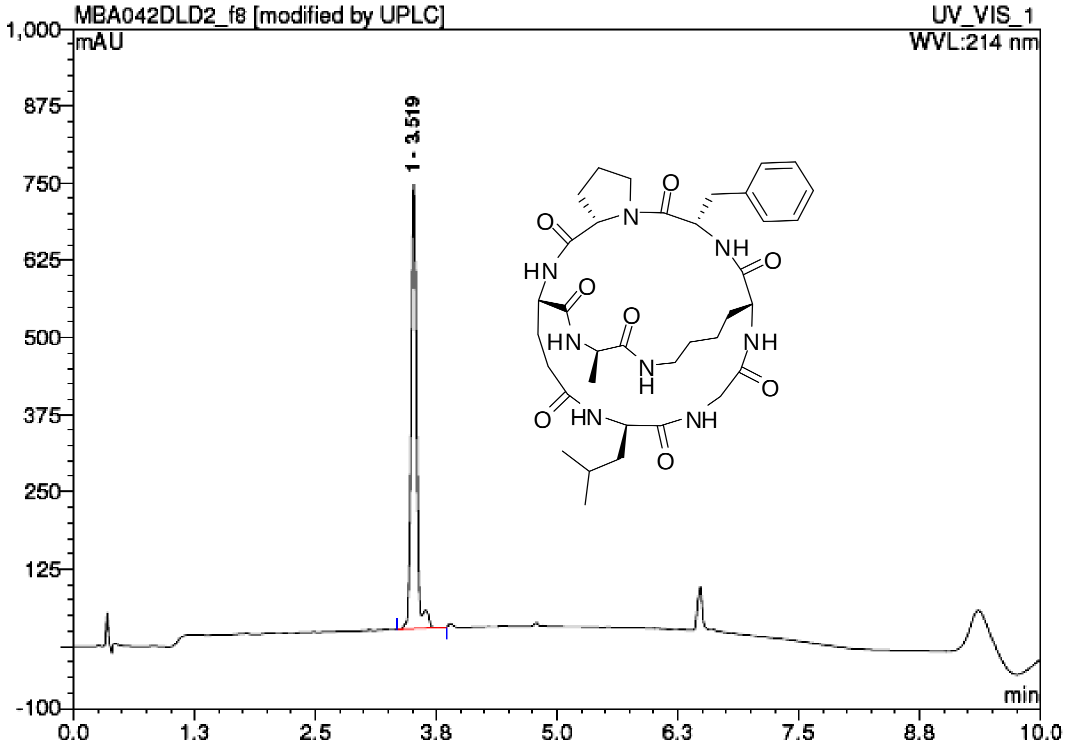

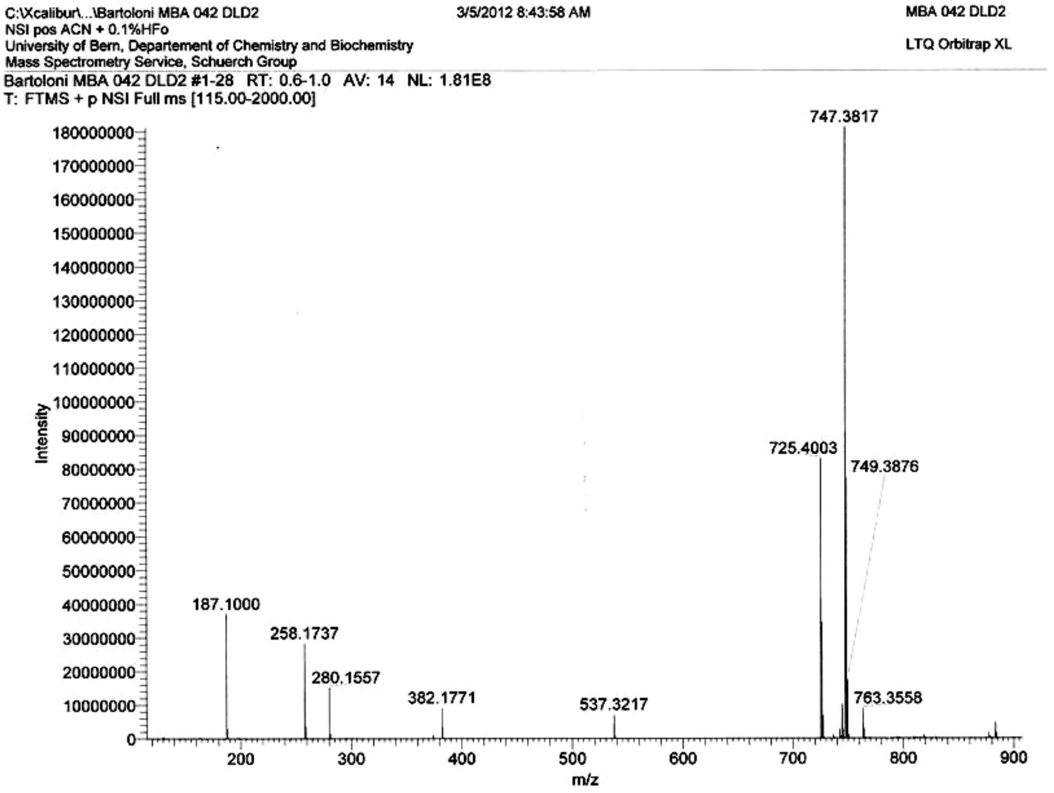

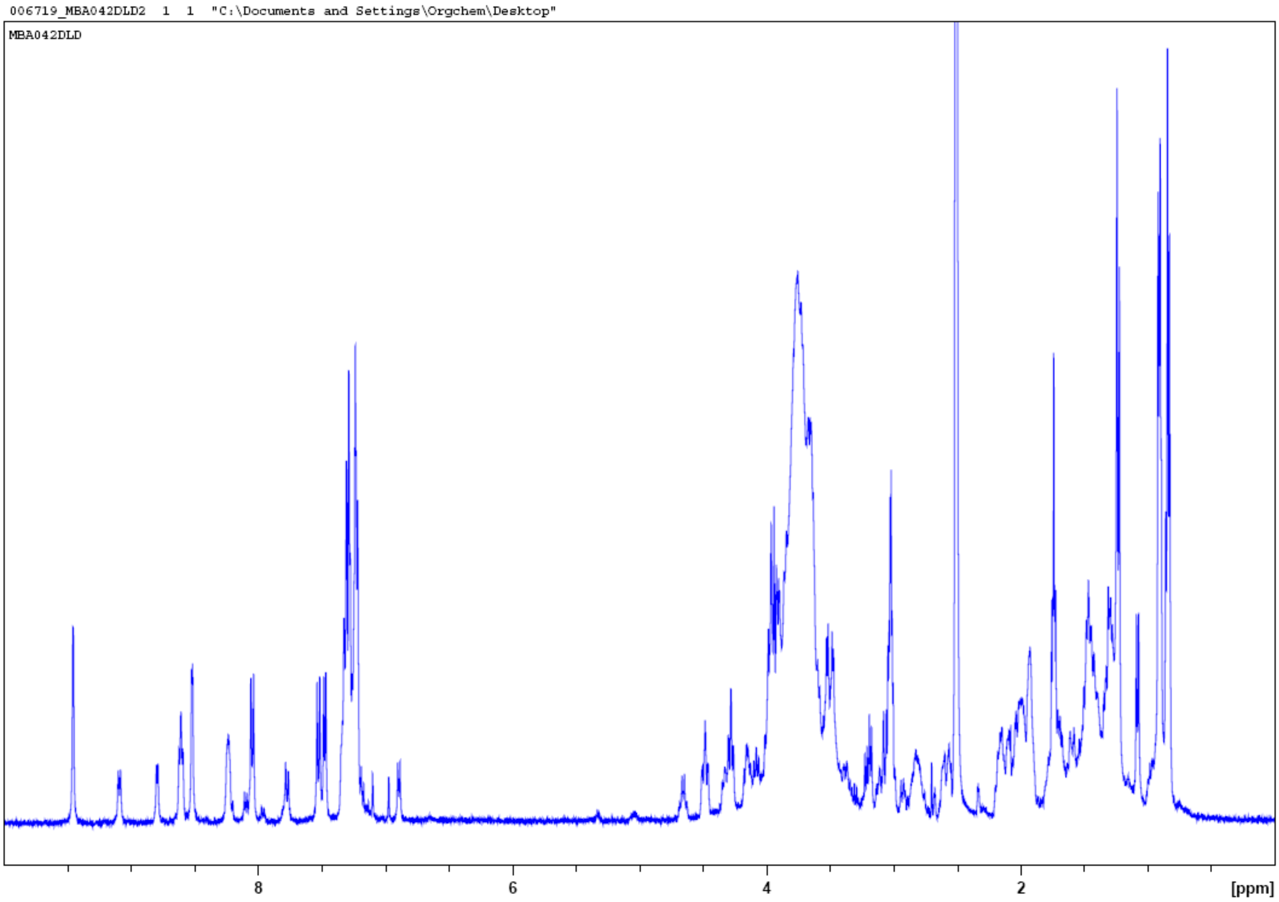

**^{1}H-NMR (400 MHz, DMSO-d_6)**				
residue	***proton***	***δ (ppm) conform. A***	***δ (ppm) conform. B***	***J (Hz)***
leu[1]	NH	8.52	8.25	^3J(NH–αCH) = 3.00 (A)<br>^3J(NH–αCH) = 4.00 (B)
Gly[2]	NH	8.61	8.23	^3J(NH–α'CH) = 4.09 (A)<br>^3J(NH–α"CH) = 8.09 (A)
lys[3]	αNH	8.05	6.90	^3J(NH–αCH) = 8.18 (A)<br>^3J(NH–αCH) = 7.17 (B)
Phe[4]	NH	9.46	9.09	^3J(NH–αCH) = 1.08 (A)<br>^3J(NH–αCH) = 6.77 (B)
Glu[6]	NH	7.53	8.80	^3J(NH–αCH) = 8.67 (A)<br>^3J(NH–αCH) = 4.88 (B)
ala[7]	NH	7.48	7.78	^3J(NH–αCH) = 6.47 (A)<br>^3J(NH–αCH) = 8.51 (B)

L^{1}G*k*^{2}fP*E*^{1}a^2 (4).

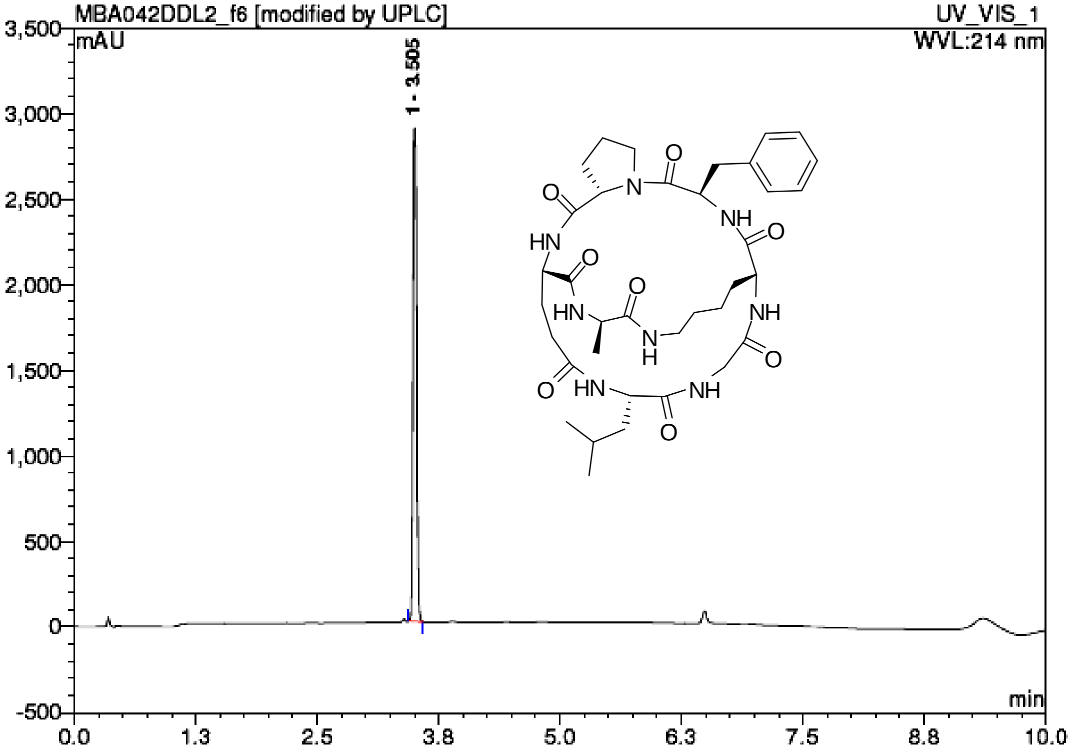

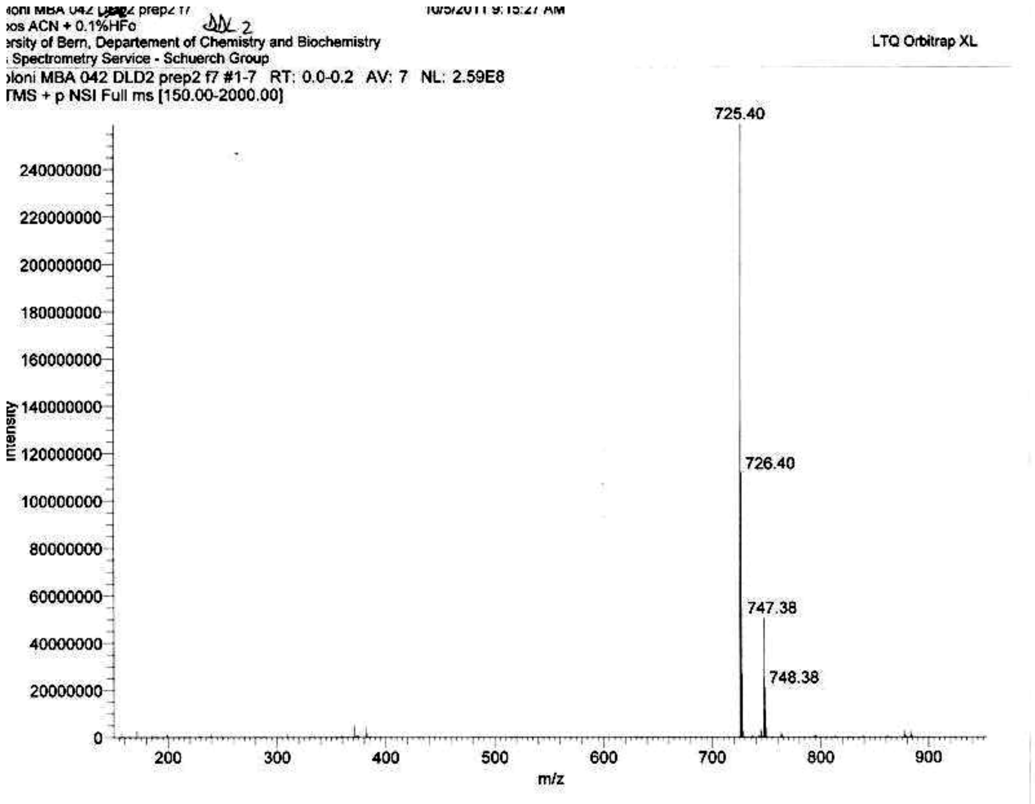

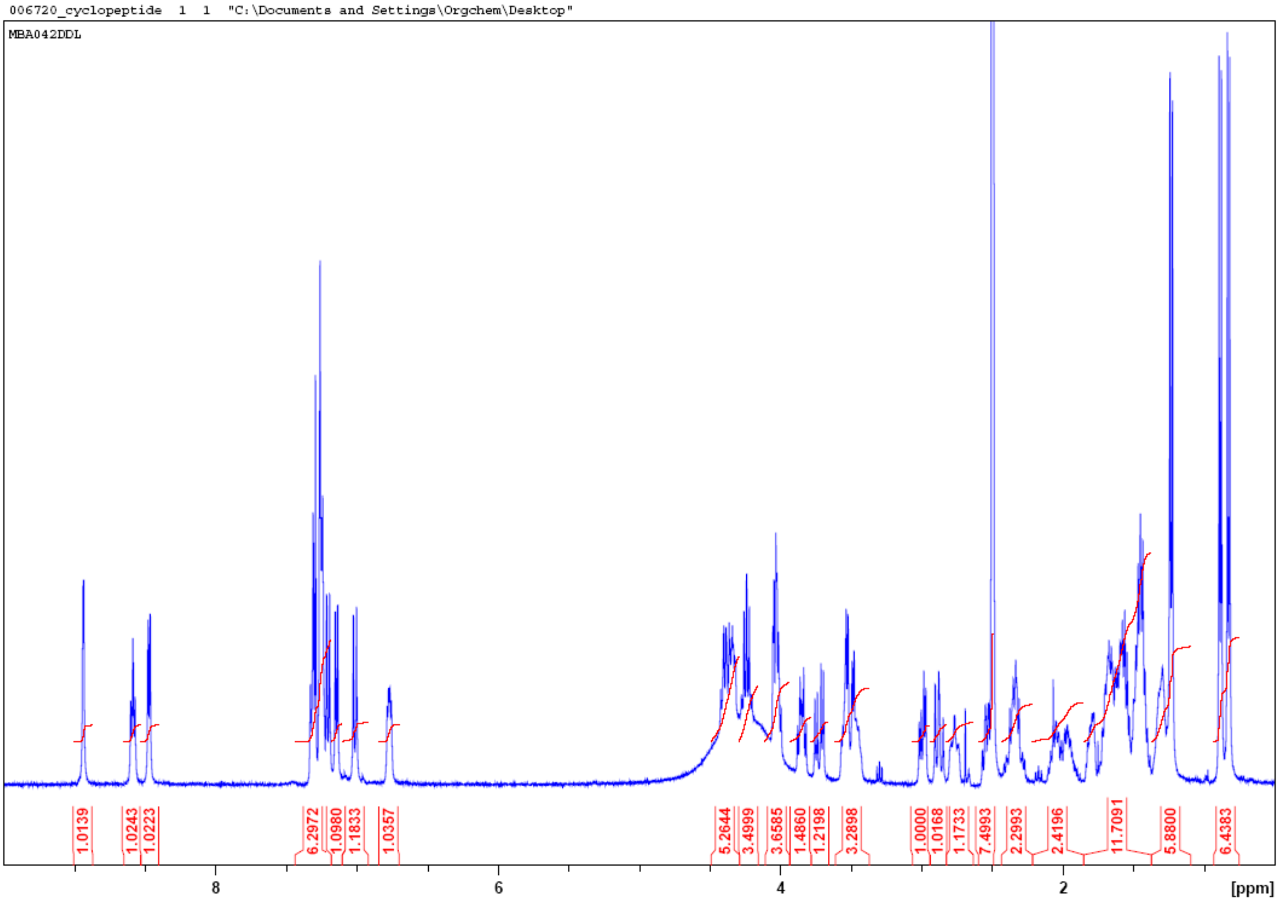

^{1}H-NMR (400 MHz, DMSO-d_6)

residue	*proton*	*δ (ppm)*	*J (Hz)*
Leu[1]	NH	8.47	^3J(NH–αCH) = 5.79
	αCH	4.02	
	βCH_2	1.45	
	γCH	1.55	
	δ'CH_3	0.88	
	δ"CH_3	0.83	
Gly[2]	NH	8.59	^3J(NH–α'CH) = 5.10
	α'CH	3.73	^3J(NH–α"CH) = 7.20
	α"CH	3.51	
lys[3]	αNH	7.01	^3J(NH–αCH) = 9.04
	αCH	4.40	
	β'CH	1.65	
	β"CH	1.31	
	γCH_2	1.47	
	δ'CH	1.60	
	δ"CH	1.30	
	ε'CH	3.47	
	ε"CH	2.78	^3J(NH–ε'CH) = 3.82
	εNH	6.77	^3J(NH–ε"CH) = 7.42
phe[4]	NH	8.94	^3J(NH–αCH) = 2.15
	αCH	4.34	
	β'CH	2.99	
	β"CH	2.88	
	2,6H	7.25	
	3,4,5H	7.30	
Pro[5]	αCH	4.04	
	β'CH	1.79	
	β"CH	1.68	
	γCH_2	1.67	
	δ'CH	3.55	
	δ"CH	2.55	
Glu[6]	NH	7.14	^3J(NH–αCH) = 6.95
	αCH	3.85	
	β'CH	2.05	
	β"CH	1.97	
	γCH_2	2.35	
ala[7]	NH	7.21	^3J(NH–αCH) = 8.23
	αCH	4.24	
	βCH_3	1.24	

l^{1}G*k*^{2}FP*E*^{1}A^2 (5).

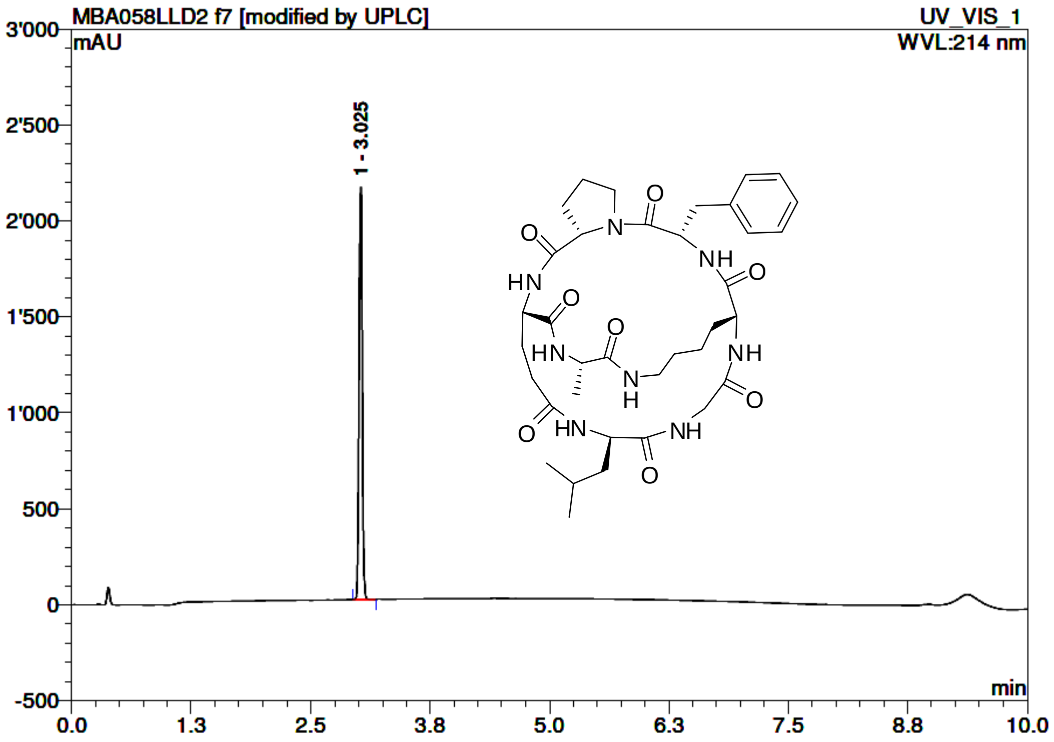

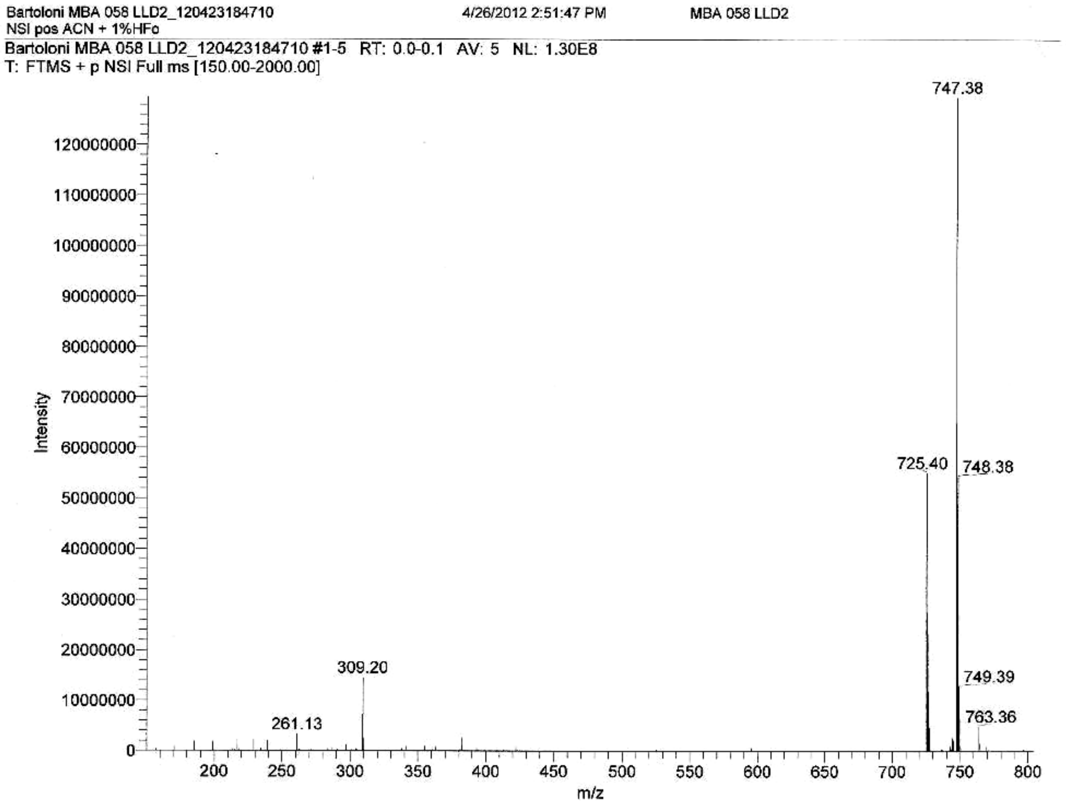

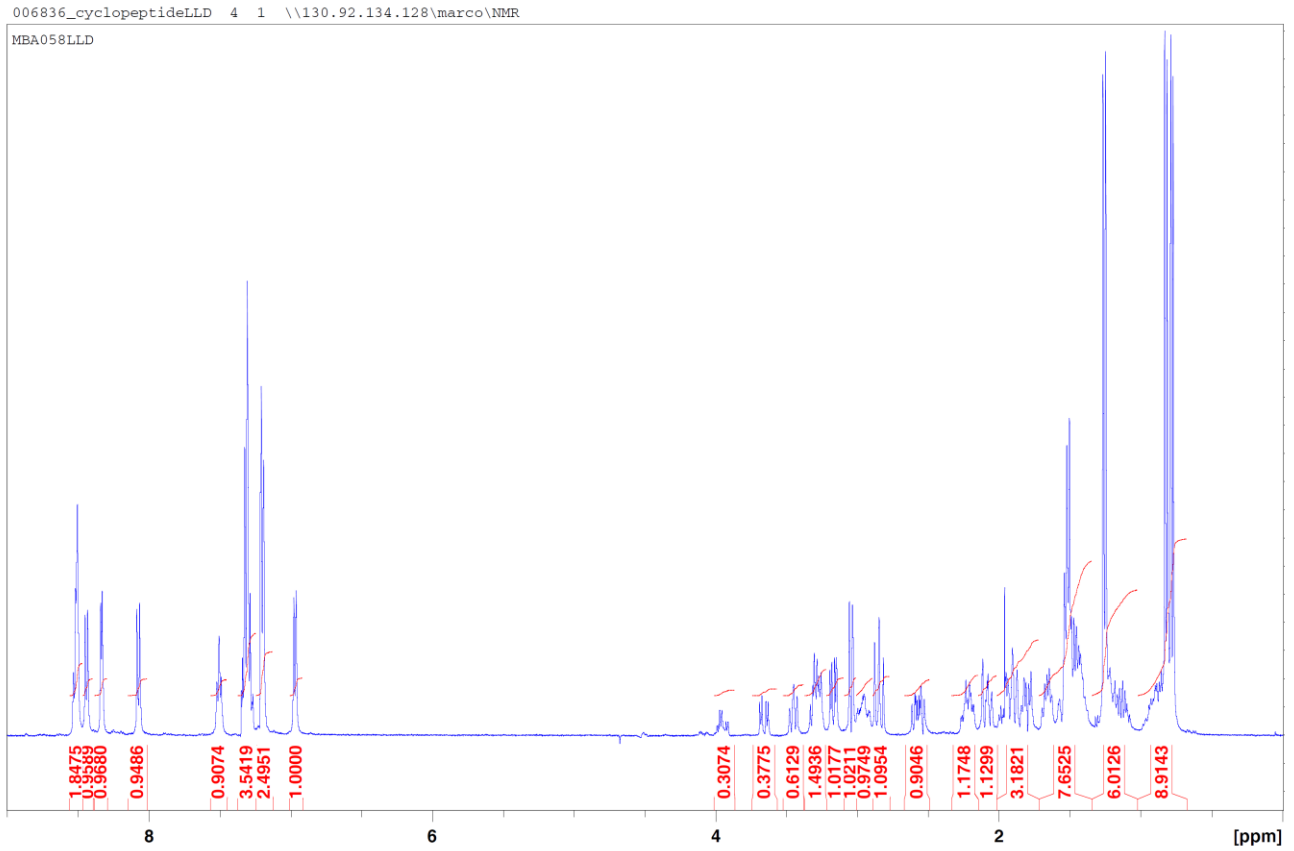

^{1}H-NMR (500 MHz, H_2O/D_2O 9:1)

residue	***proton***	***δ (ppm)***	***J (Hz)***
leu[1]	NH	8.06	^3J(NH–αCH) = 7.49
	αCH	4.36	
	$βCH_2$	1.51	
	γCH	1.47	
	$δ'CH_3$	0.82	
	$δ''CH_3$	0.78	
Gly[2]	NH	8.52	^3J(NH–αCH) = 6.01
	α'CH	3.94	
	α''CH	3.77	
lys[3]	αNH	6.97	^3J(NH–αCH) = 6.53
	αCH	4.53	
	β'CH	1.95	
	β''CH	1.53	
	$γCH_2$	0.87	
	δ'CH	1.45	
	δ''CH	1.23	
	ε'CH	3.27	
	ε''CH	2.95	
	εNH	7.50	^3J(NH–εCH) = 6.29
Phe[4]	NH	8.50	
	αCH	4.50	
	β'CH	3.16	
	β''CH	2.85	
	2,6H	7.20	
	3,4,5H	7.31	
Pro[5]	αCH	3.05	
	β'CH	1.79	
	β''CH	1.11	
	γ'CH	1.66	
	γ''CH	1.45	
	δ'CH	3.44	
	δ''CH	3.29	
Glu[6]	NH	8.44	^3J(NH–αCH) = 7.17
	αCH	4.09	
	β'CH	2.21	
	β''CH	1.88	
	γ'CH	2.57	
	γ''CH	2.08	
Ala[7]	NH	8.33	^3J(NH–αCH) = 4.45
	αCH	3.98	
	$βCH_3$	1.25	

L^{1}G*k*^{2}fP*E*^{1}A^{2} (6).

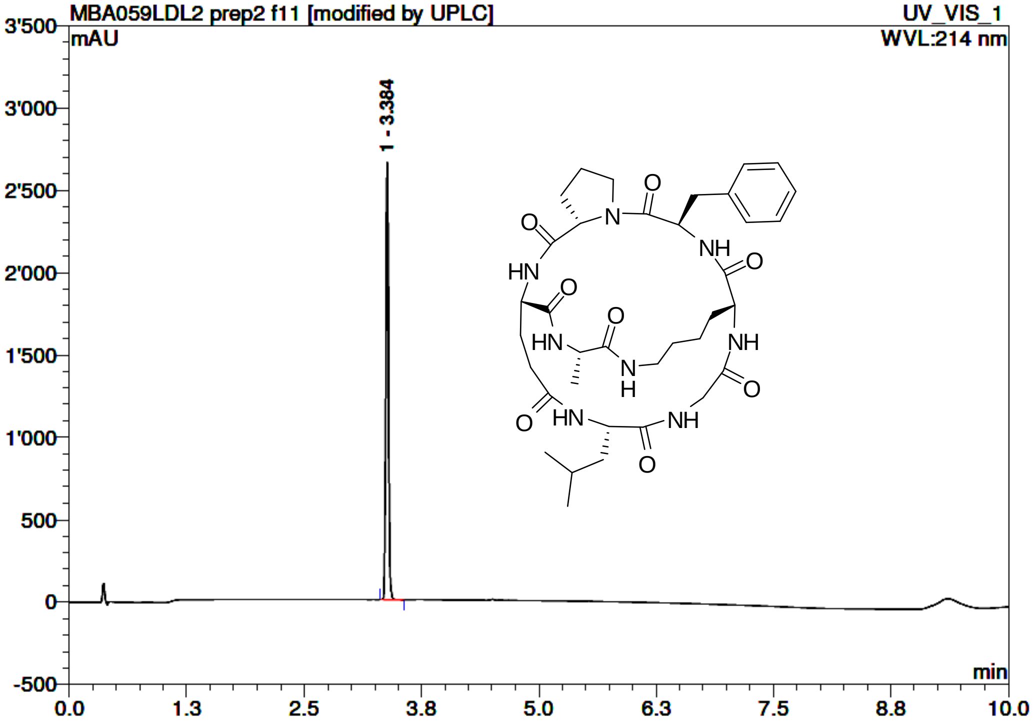

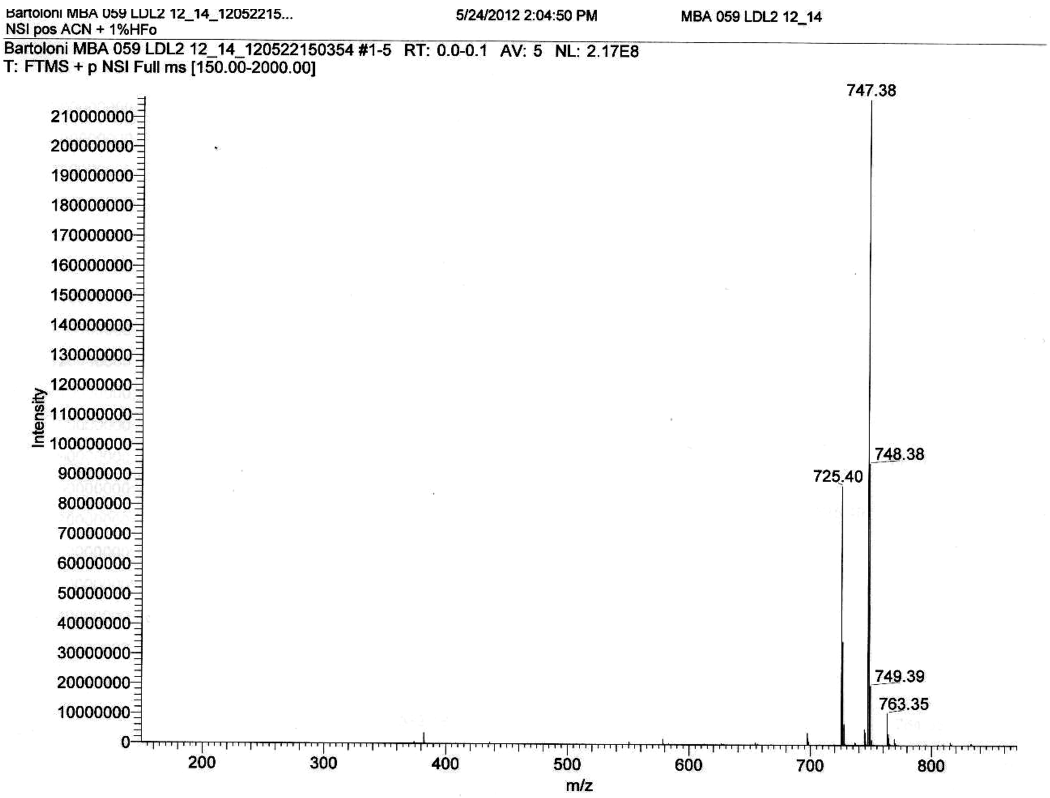

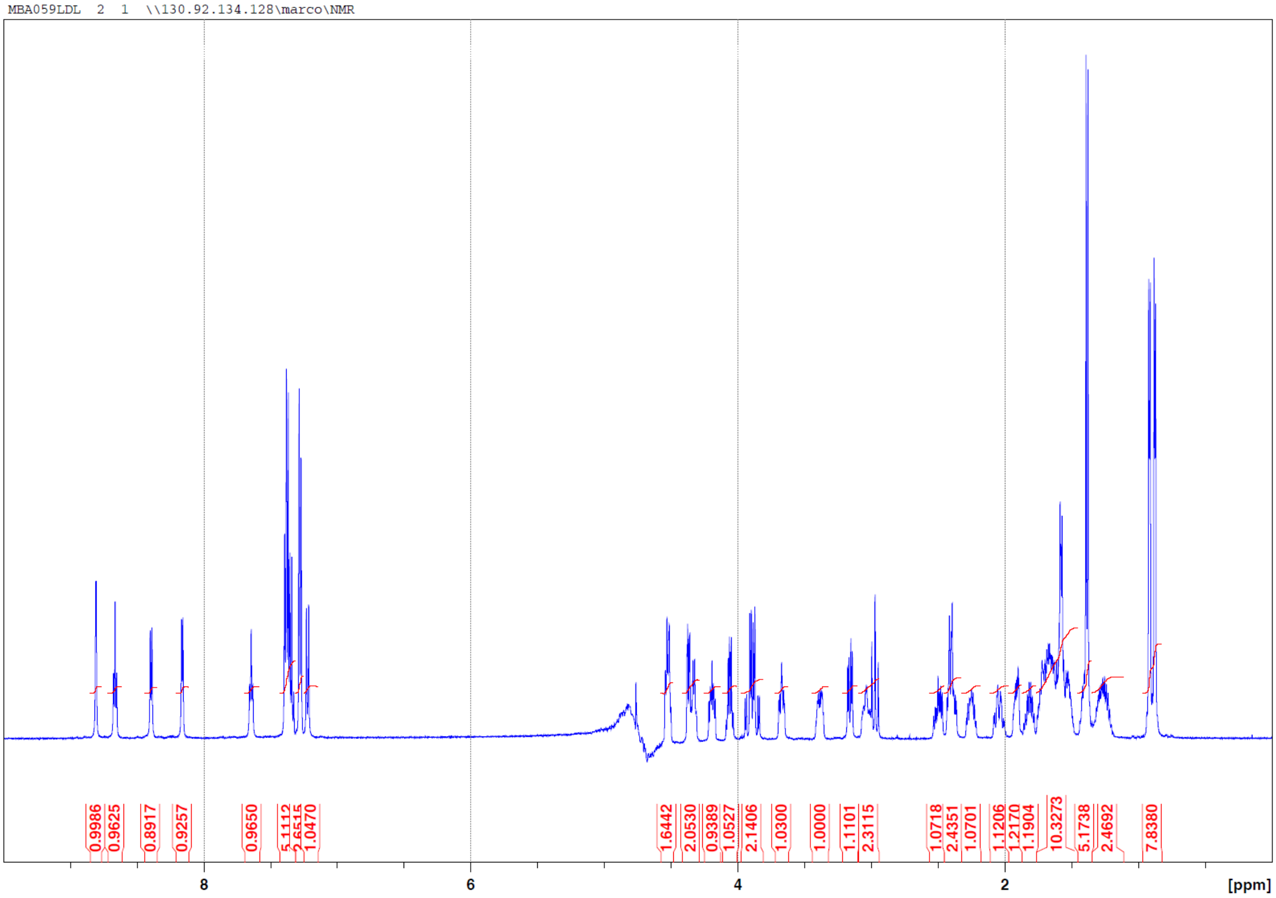

^{1}H-NMR (500 MHz, H_2O/D_2O 9:1)

residue	***proton***	***δ (ppm)***	***J (Hz)***
Leu[1]	NH	8.39	^{3}J(NH–αCH) = 6.26
	αCH	4.23	
	$βCH_2$	1.47	
	γCH	1.48	
	$δ'CH_3$	0.81	
	$δ''CH_3$	0.78	
Gly[2]	NH	8.67	^{3}J(NH–α'CH) = 5.08
	$αCH_2$	3.78	^{3}J(NH–α"CH) = 7.09
lys[3]	αNH	7.22	^{3}J(NH–αCH) = 8.45
	αCH	4.42	
	β'CH	1.55	
	β"CH	1.31	
	$γCH_2$	1.16	
	δ'CH	1.70	
	δ"CH	1.62	
	ε'CH	3.28	
	ε"CH	2.95	^{3}J(NH– ε'CH) = 4.24
	εNH	7.65	^{3}J(NH– ε"CH) = 7.65
phe[4]	NH	8.81	^{3}J(NH–αCH) = 1.70
	αCH	4.44	
	β'CH	3.07	
	β"CH	2.88	
	2,6H	7.19	
	3,5H	7.28	
	4H	7.26	
Pro[5]	αCH	4.27	
	β'CH	1.80	
	β"CH	1.58	
	γ'CH	1.54	
	γ"CH	1.44	
	δ'CH	3.57	
	δ"CH	2.32	
Glu[6]	NH	7.38	^{3}J(NH–αCH) = 7.56
	αCH	4.09	
	β'CH	2.15	
	β"CH	1.94	
	γ'CH	2.39	
	γ"CH	2.29	
Ala[7]	NH	8.16	^{3}J(NH–αCH) = 5.01
	αCH	3.97	
	$βCH_3$	1.29	

L[1]G*k*[2]FP*E*[1]a[2] (7).

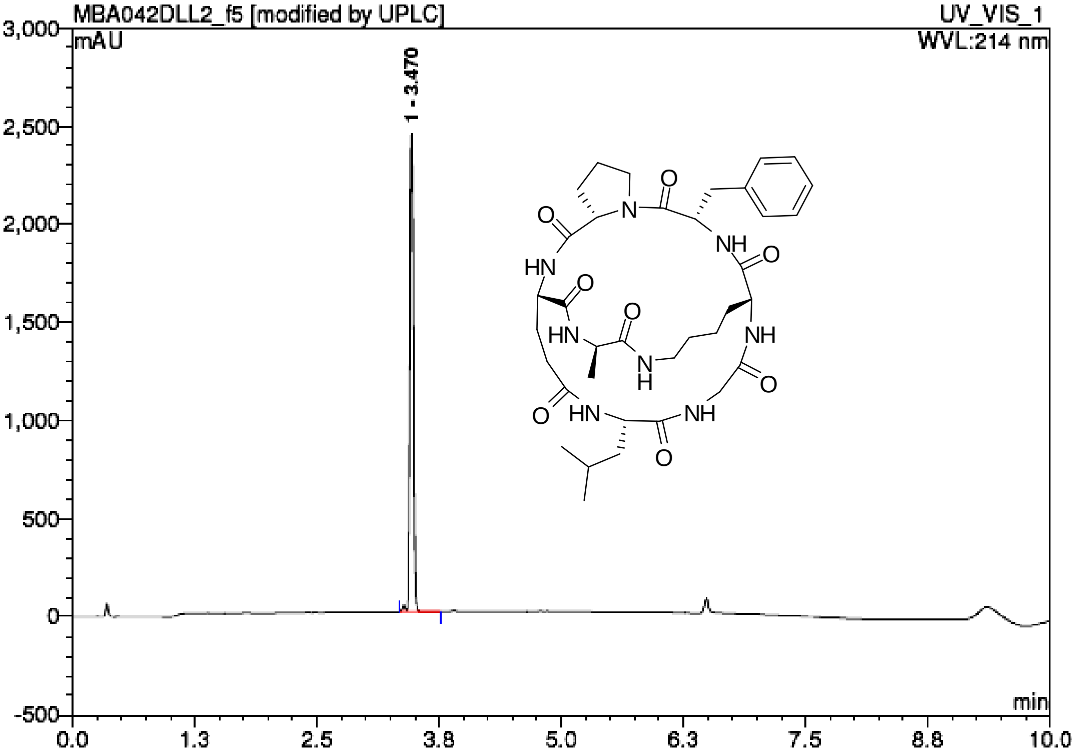

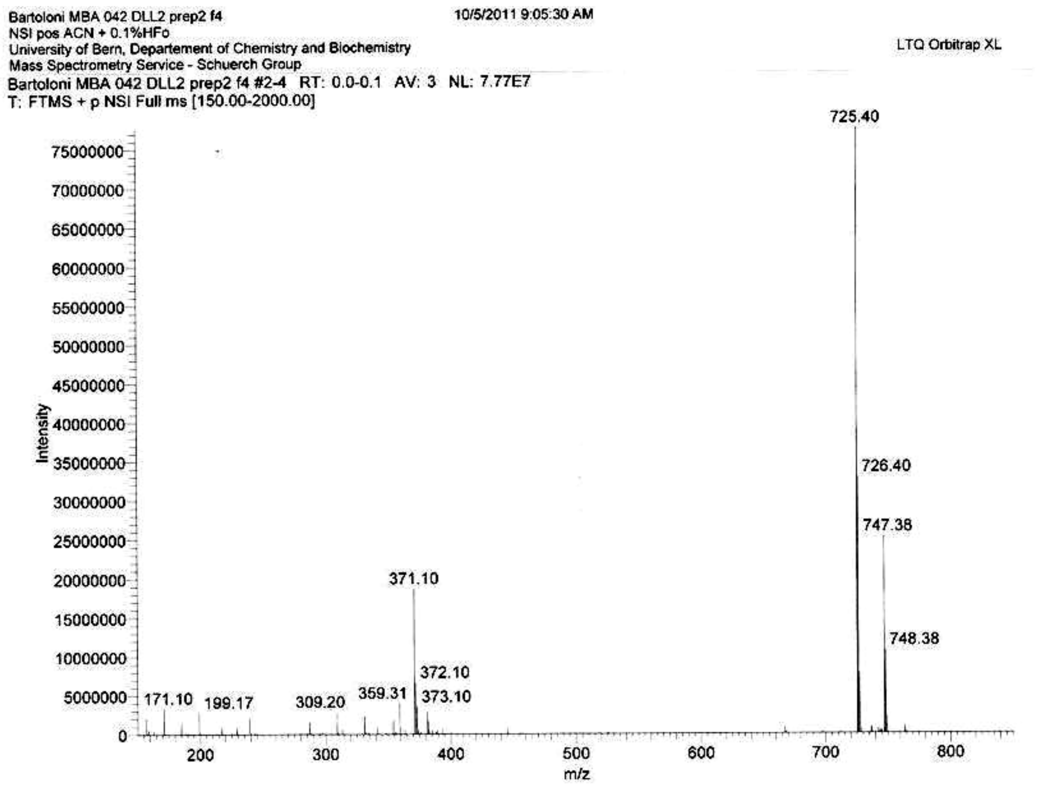

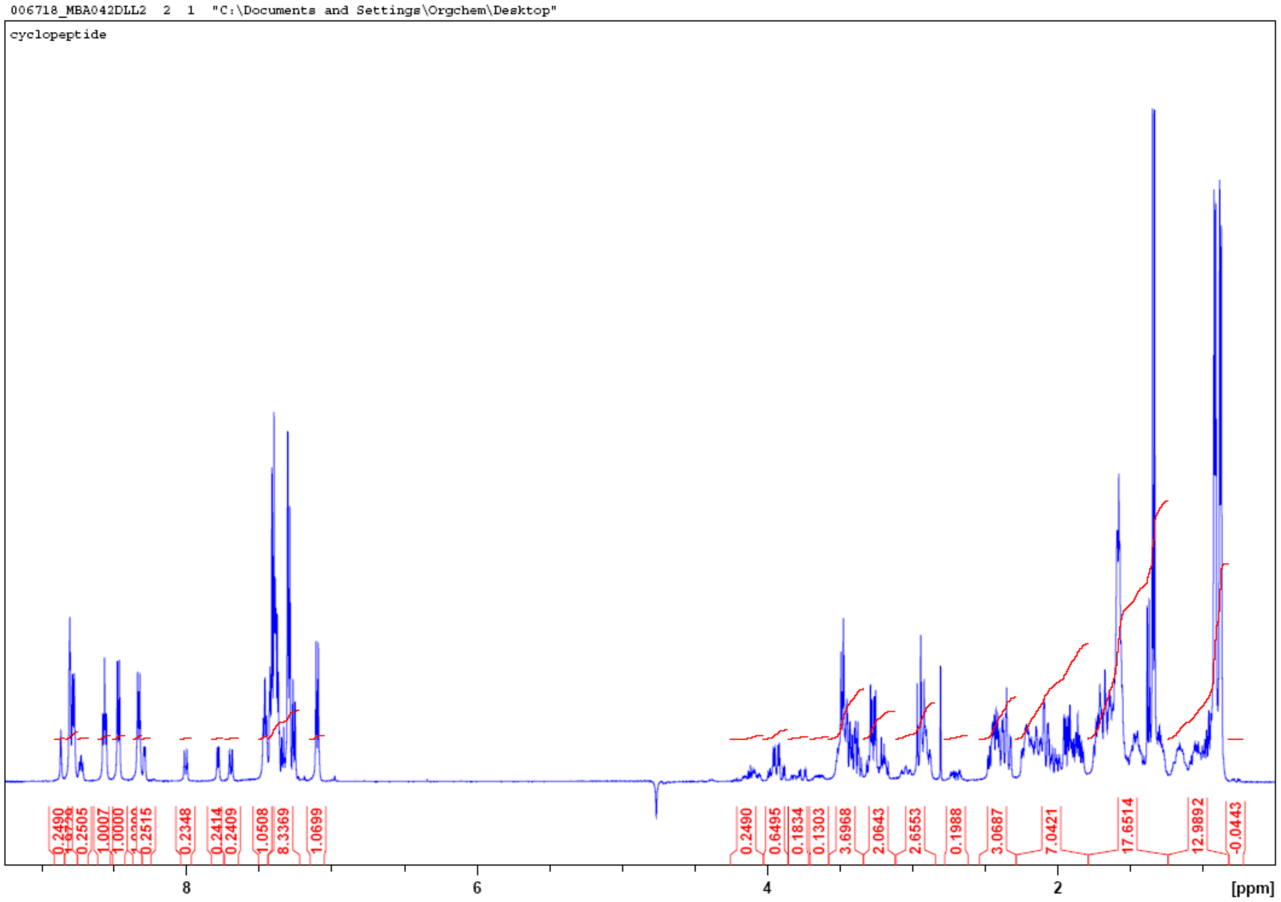

**^{1}H-NMR (500 MHz, H_2O/D_2O 9:1)**				
residue	***proton***	***δ (ppm) conform. A***	***δ (ppm) conform. B***	***J (Hz)***
Leu[1]	NH	8.38	8.20	^3J(NH–αCH) = 7.04 (A)
				^3J(NH–αCH) = 4.04 (B)
Gly[2]	NH	8.48	8.64	^3J(NH–α'CH) = 5.38 (A)
				^3J(NH–α''CH) = 6.58 (A)
				^3J(NH–α'CH) = 5.54 (B)
				^3J(NH–α''CH) = 7.04 (B)
lys[3]	αNH	7.01	7.92	^3J(NH–αCH) = 7.78 (A)
				^3J(NH–αCH) = 8.83 (B)
	εNH	7.37		^3J(NH–ε'CH) = 4.79 (A)
				^3J(NH–ε''CH) = 7.48 (A)
Phe[4]	NH	8.72	8.78	^3J(NH–αCH) = 1.80 (A)
Glu[6]	NH	8.69	7.61	^3J(NH–αCH) = 6.29 (A)
				^3J(NH–αCH) = 9.28 (B)
ala[7]	NH	8.24	7.60	^3J(NH–αCH) = 7.48 (A)
				^3J(NH–αCH) = 6.14 (B)

L[1]G*k*[2]FP*E*[1]A[2] (8).

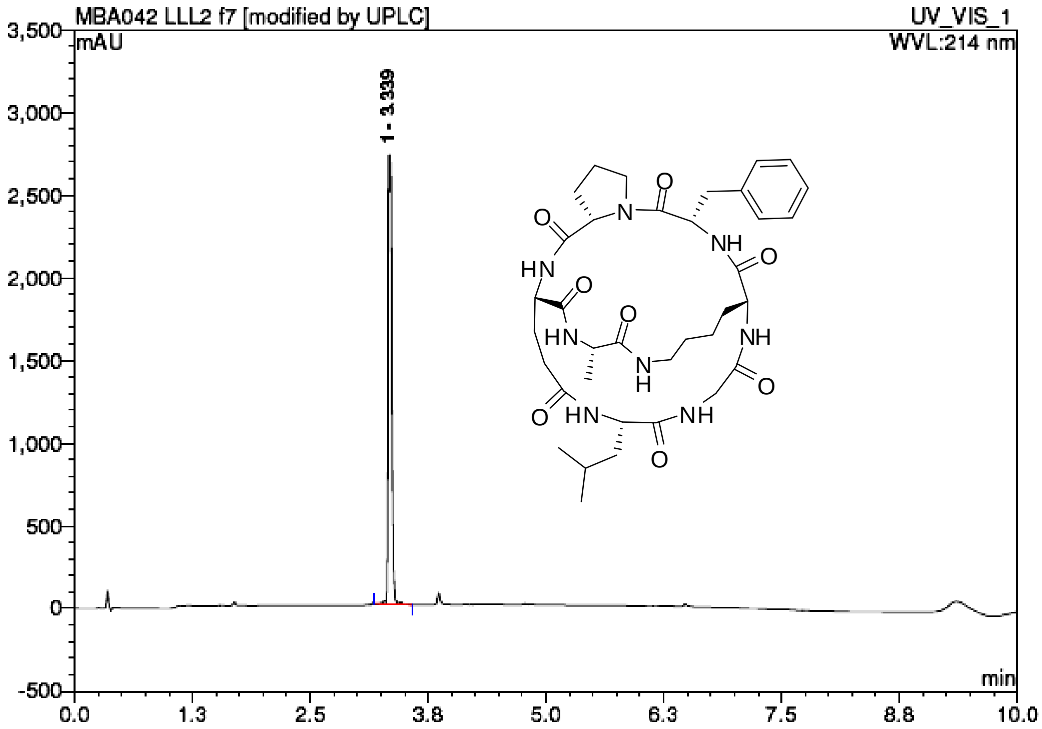

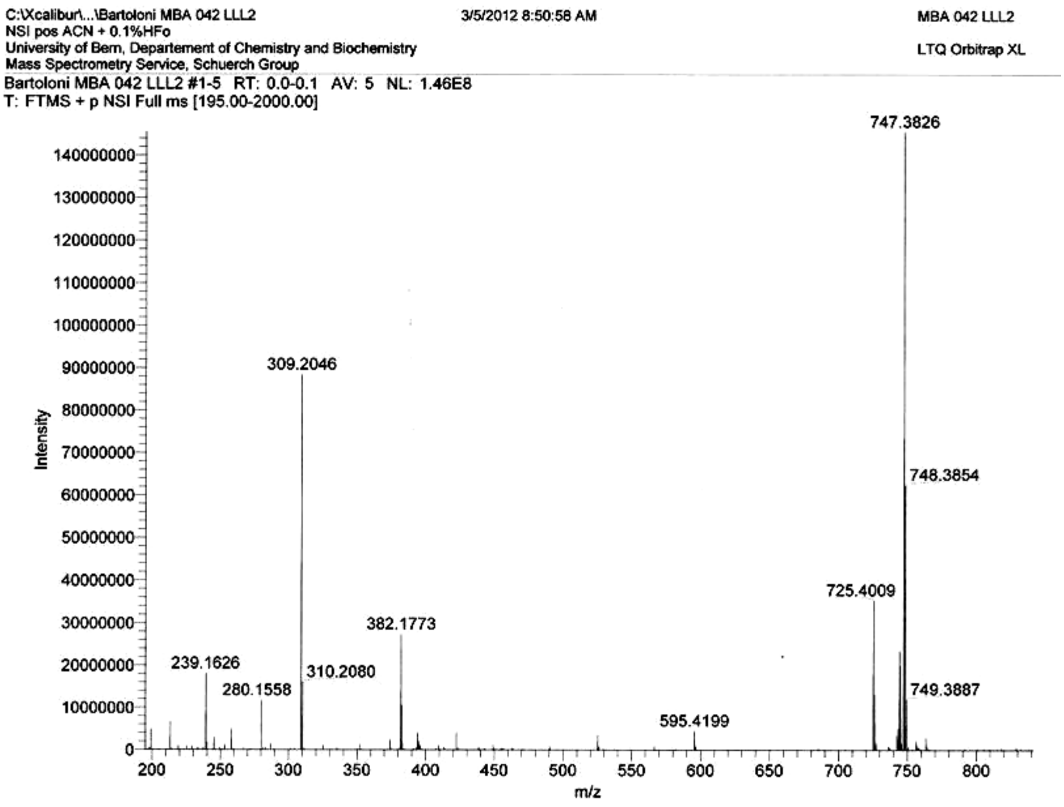

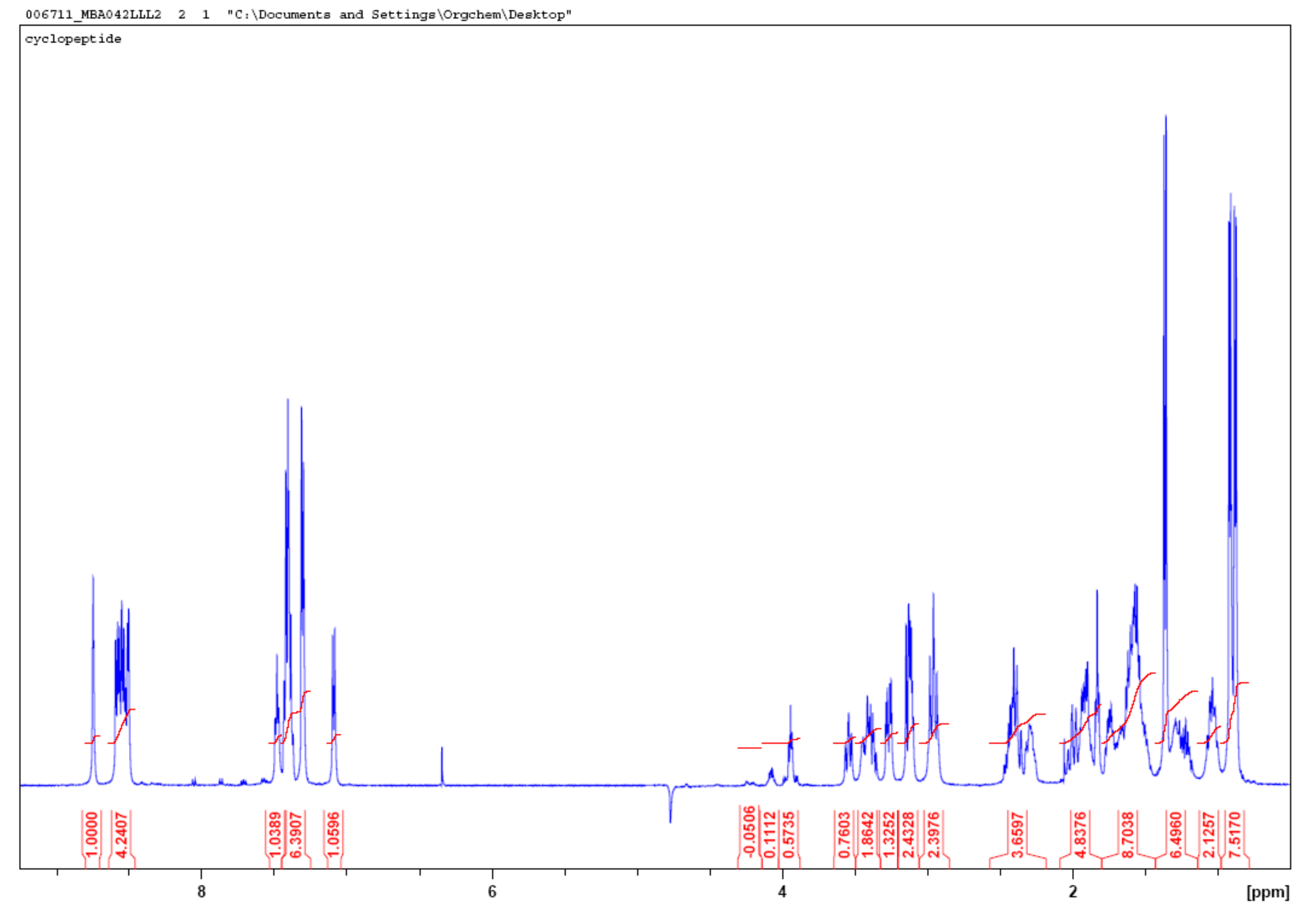

^{1}H-NMR (500 MHz, H_2O/D_2O 9:1)			
residue	***proton***	***δ (ppm)***	***J (Hz)***
Leu^{1}	NH	8.55	^{3}J(NH–αCH) = 8.00
	αCH	4.42	
	$βCH_2$	1.58	
	γCH	1.56	
	$δ'CH_3$	0.89	
	$δ''CH_3$	0.85	
Gly^{2}	NH	8.54	^{3}J(NH–α'CH) = 4.22
	$αCH_2$	3.92	^{3}J(NH–α"CH) = 7.94
lys^{3}	αNH	7.11	^{3}J(NH–αCH) = 7.50
	αCH	4.74	
	β'CH	1.87	
	β"CH	1.65	
	$γCH_2$	1.01	
	δ'CH	1.57	
	δ"CH	1.27	
	ε'CH	3.39	
	ε"CH	2.93	^{3}J(NH–ε'CH) = 4.22
	εNH	7.49	^{3}J(NH–ε"CH) = 7.94
Phe^{4}	NH	8.75	
	αCH	4.62	
	β'CH	3.23	
	β"CH	2.94	
	2,6H	7.29	
	3,4,5H	7.39	
Pro^{5}	αCH	3.12	
	β'CH	1.90	
	β"CH	1.20	
	γ'CH	1.72	
	γ"CH	1.49	
	δ'CH	3.51	
	δ"CH	3.35	
Glu^{6}	NH	8.61	^{3}J(NH–αCH) = 7.25
	αCH	4.19	
	β'CH	2.26	
	β"CH	1.97	
	$γCH_2$	2.38	
Ala^{7}	NH	8.51	^{3}J(NH–αCH) = 4.40
	αCH	4.06	
	$βCH_3$	1.34	

$L^1GK^2FPE^1A^2$ (9).

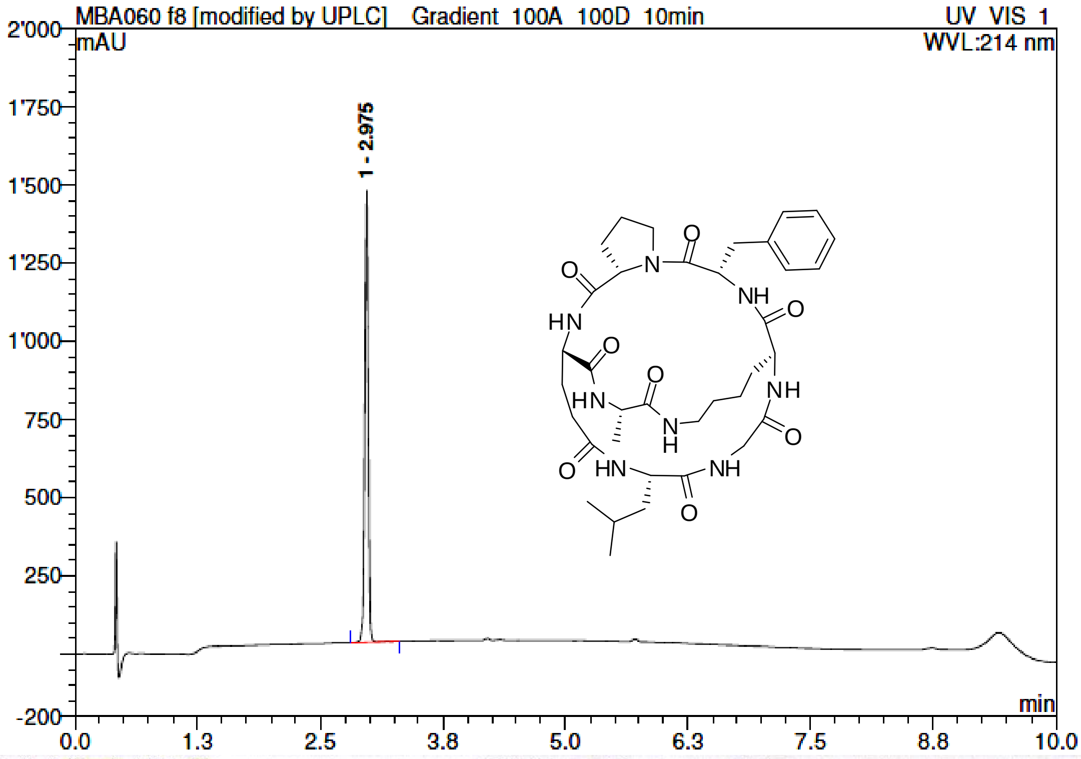

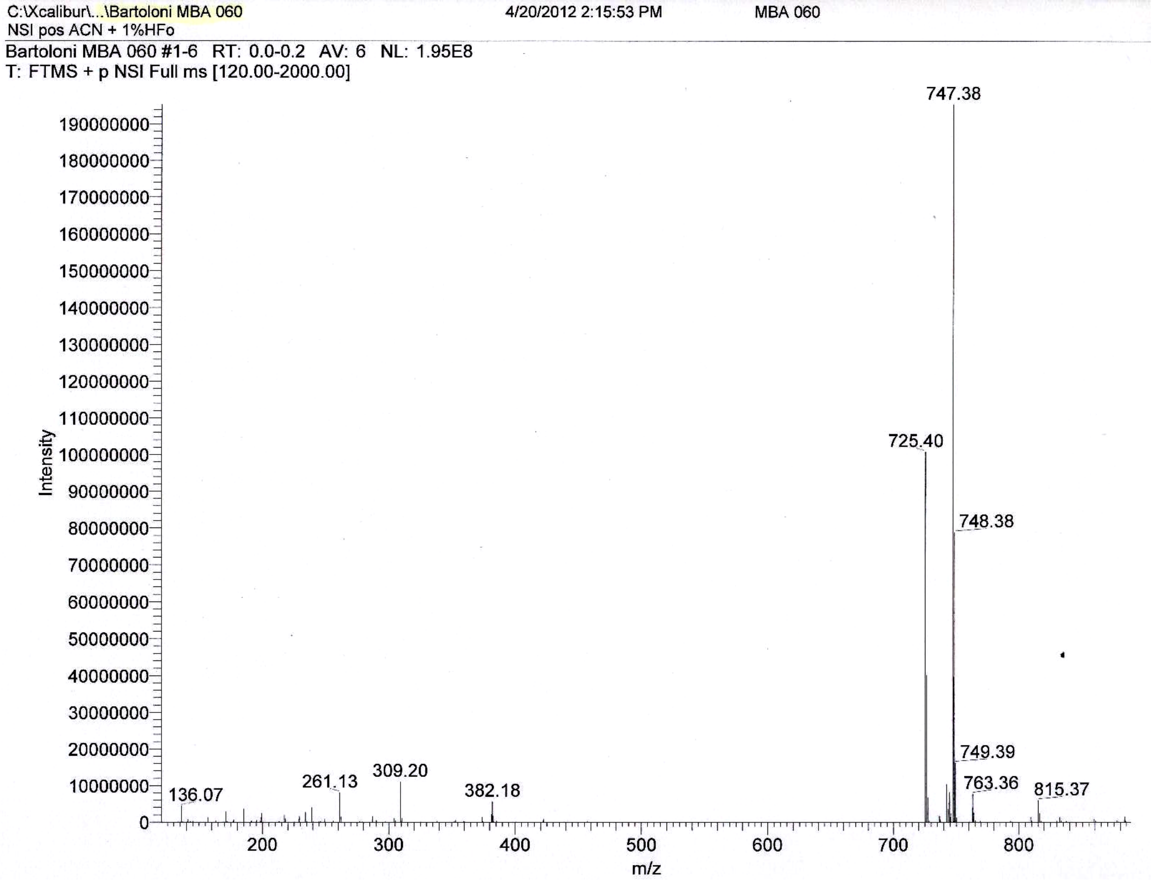

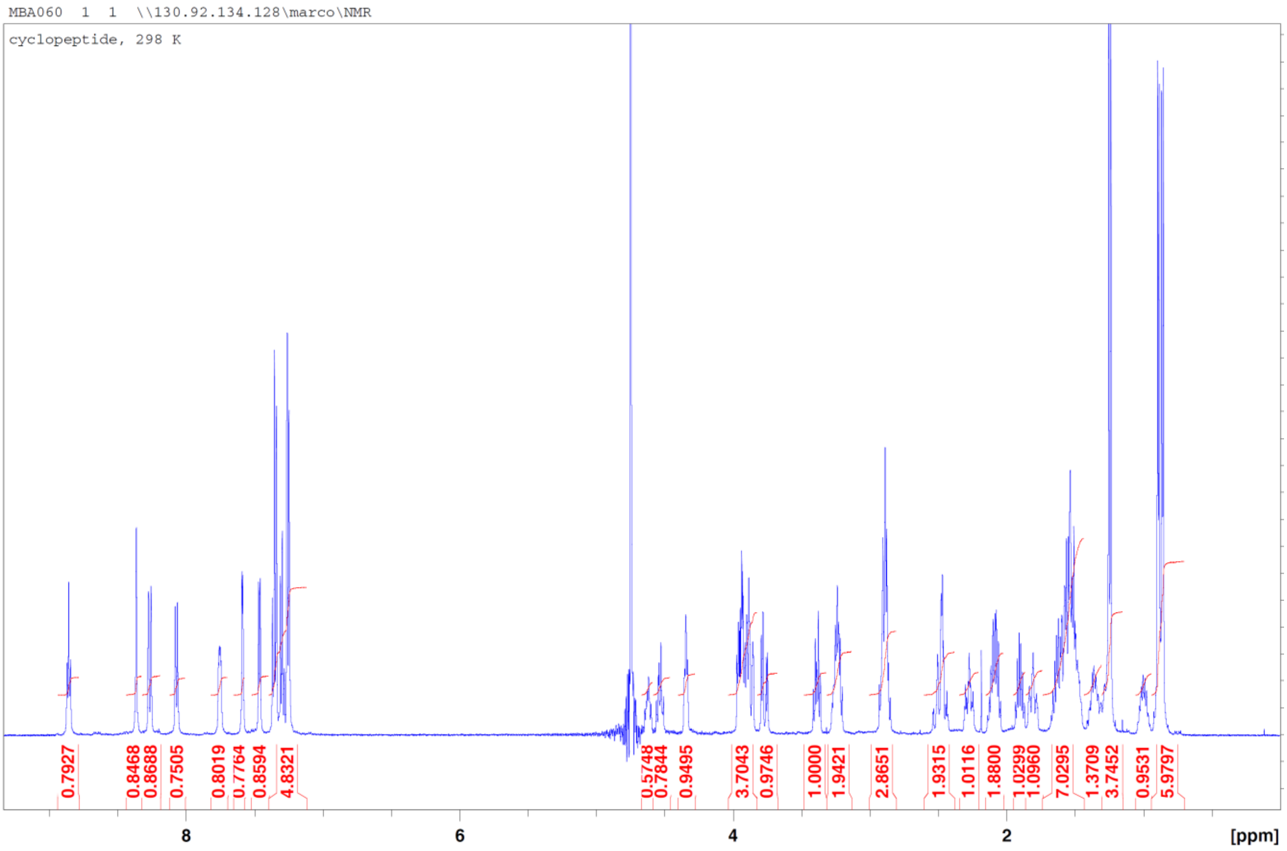

^{1}H-NMR (500 MHz, 90% H_2O/D_2O)			
residue	***proton***	***δ (ppm)***	***J (Hz)***
Leu[1]	NH	8.07	^3J(NH–αCH) = 8.25
	αCH	4.63	
	$βCH_2$	1.54	
	γCH	1.58	
	$δ'CH_3$	0.89	
	$δ''CH_3$	0.86	
Gly[2]	NH	8.86	^3J(NH–αCH) = 6.10
	α'CH	3.95	
	α''CH	3.77	
Lys[3]	αNH	7.46	^3J(NH–αCH) = 6.00
	αCH	4.35	
	β'CH	2.09	
	β''CH	1.54	
	γ'CH	1.27	
	γ''CH	1.01	
	δ'CH	1.48	
	δ''CH	1.36	
	ε'CH	3.25	
	ε''CH	2.89	
	εNH	7.75	
Phe[4]	NH	8.27	^3J(NH–αCH) = 9.60

l[1]G*k*[2]yP*E*[1]a[2] (10).

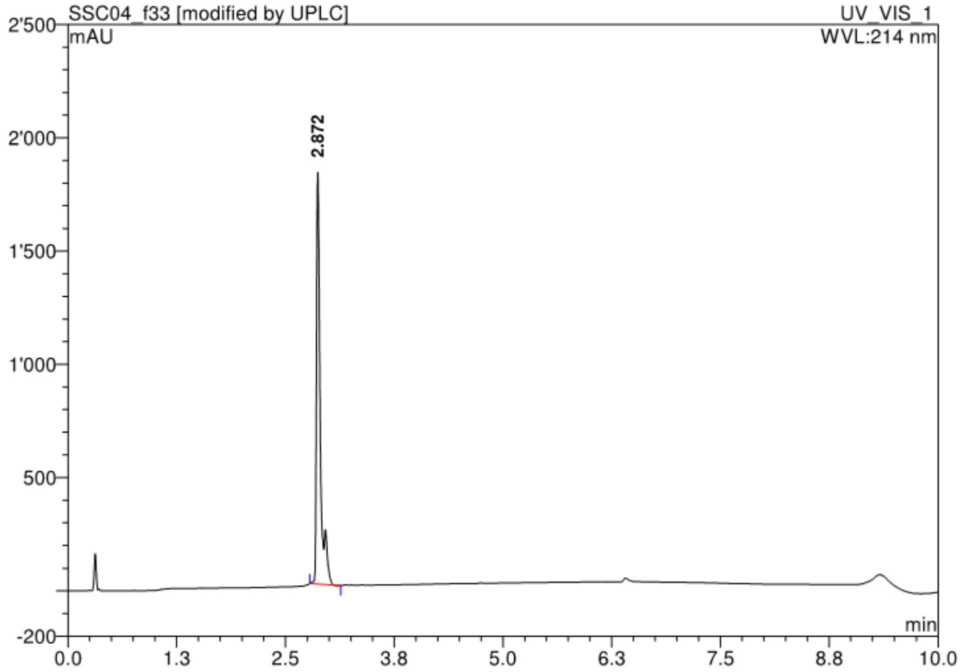

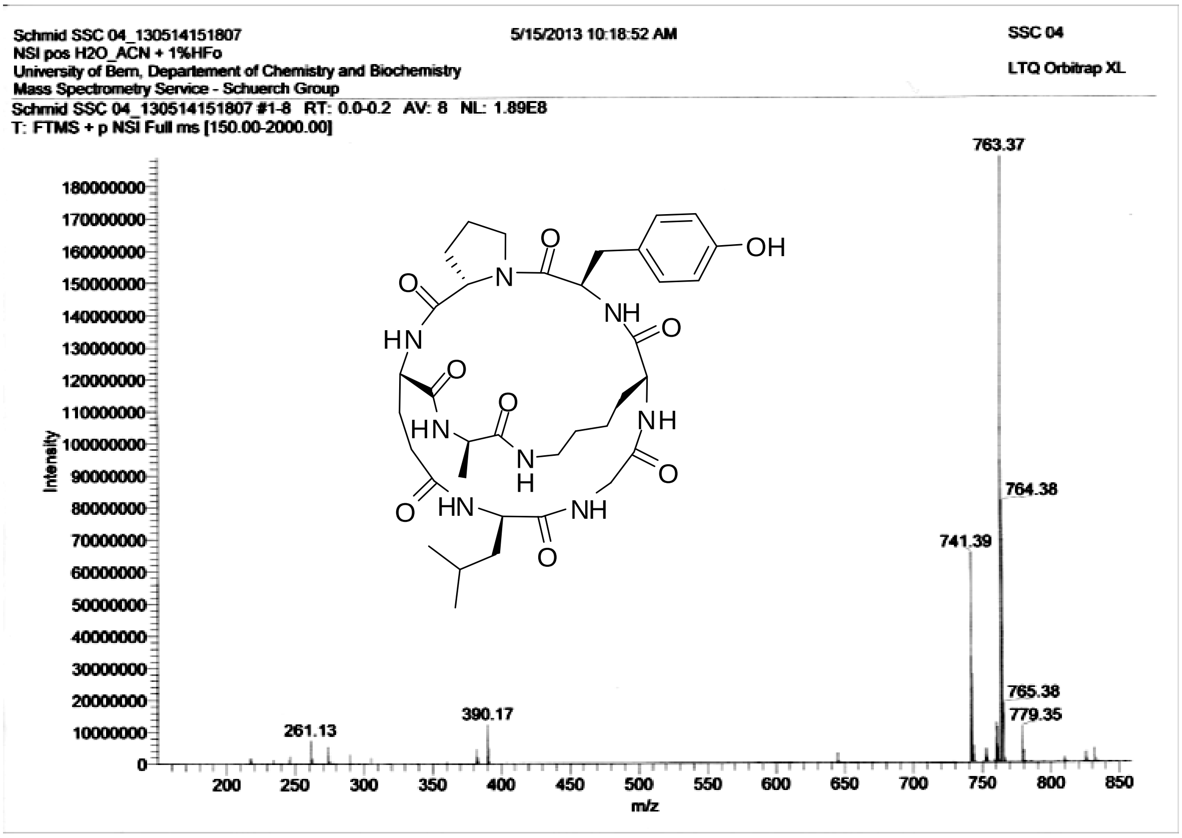

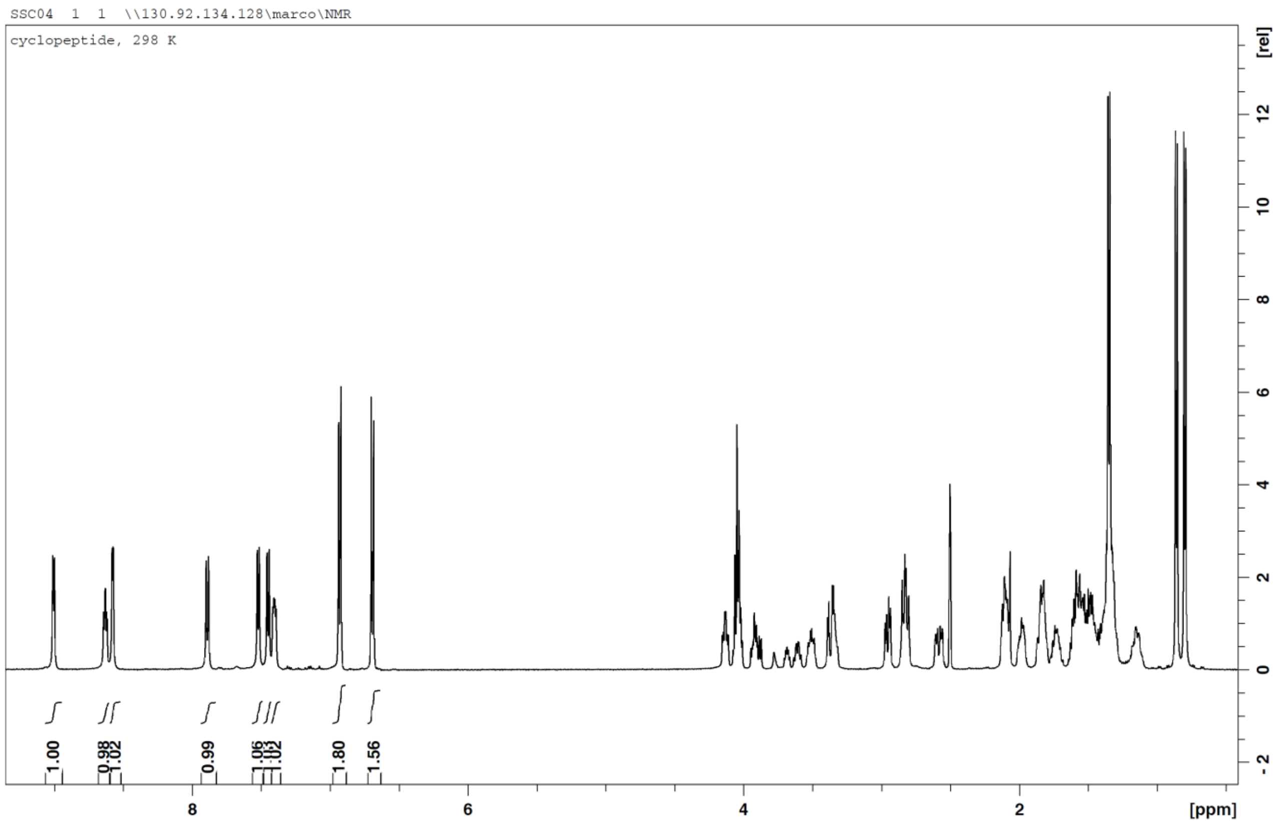

^{1}H-NMR (400 MHz, DMSO-d_6)

residue	***proton***	***δ (ppm)***	***J (Hz)***
leu[1]	NH	8.58	^3J(NH–αCH) = 4.10
	αCH	3.68	
	β'CH	1.50	
	β"CH	1.34	
	γCH	1.60	
	δ'CH_3	0.85	
	δ"CH_3	0.79	
Gly[2]	NH	8.63	^3J(NH–α'CH) = 5.47
	α'CH	3.88	^3J(NH–α"CH) = 6.73
	α"CH	3.37	
lys[3]	αNH	7.89	^3J(NH–αCH) = 8.38
	αCH	4.13	
	β'CH	1.57	
	β"CH	1.35	
	γ'CH	1.46	
	γ"CH	1.14	
	δ'CH	1.54	
	δ"CH	1.31	
	ε'CH	3.50	
	ε"CH	2.82	^3J(NH–ε'CH) = 3.99
	εNH	7.40	^3J(NH–ε"CH) = 7.73
tyr[4]	NH	9.01	^3J(NH–αCH) = 5.58
	αCH	4.03	
	β'CH	2.96	
	β"CH	2.82	
	2,6H	6.93	
	3,5H	6.69	
Pro[5]	αCH	4.05	
	β'CH	2.08	
	β"CH	1.41	
	γ'CH	1.82	
	γ"CH	1.72	
	δ'CH	3.61	
	δ"CH	3.32	
Glu[6]	NH	7.45	^3J(NH–αCH) = 7.92
	αCH	3.92	
	β'CH	1.98	
	β"CH	1.84	
	γ'CH	2.58	
	γ"CH	2.08	
ala[7]	NH	7.52	^3J(NH–αCH) = 7.07
	αCH	4.05	
	βCH_3	1.35	

l^{1}G*k*^{2}yP*E*^{1}A^2 (11).

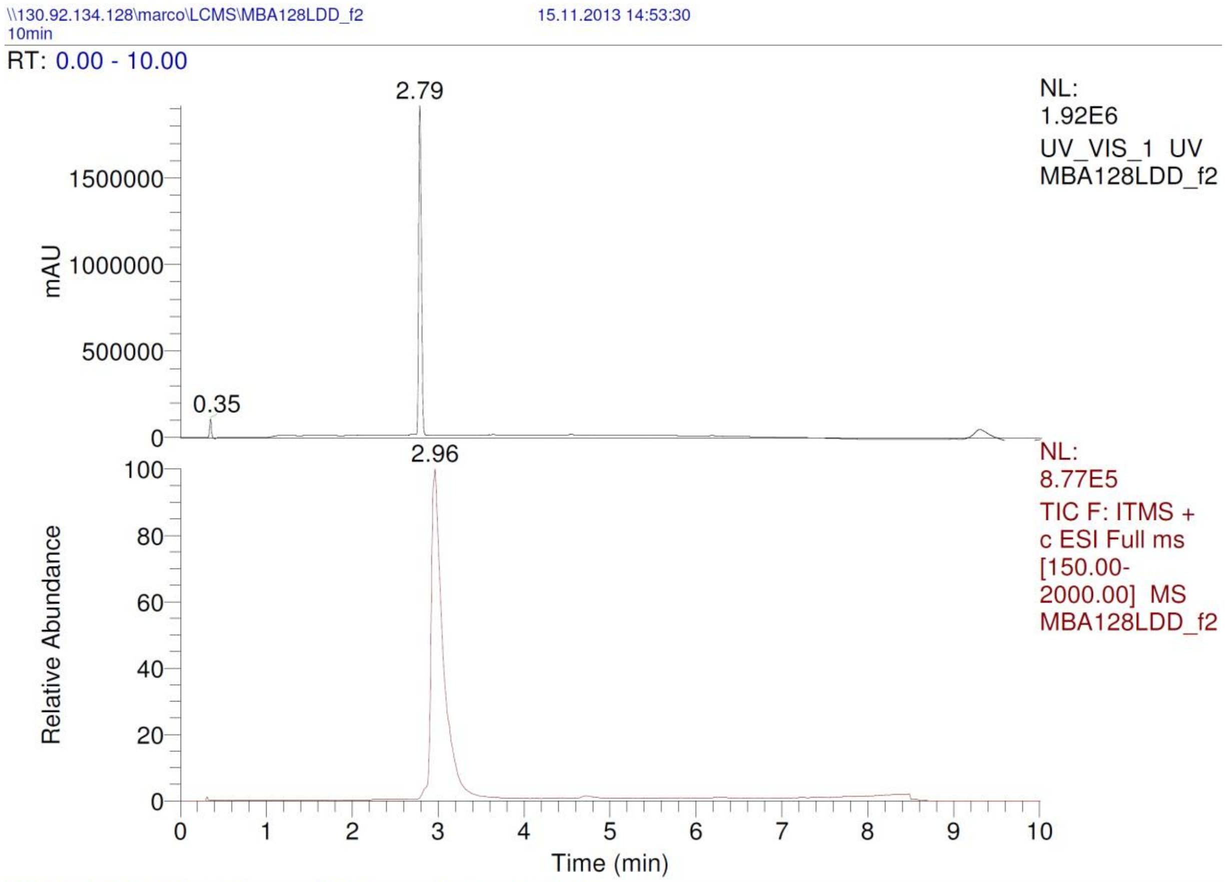

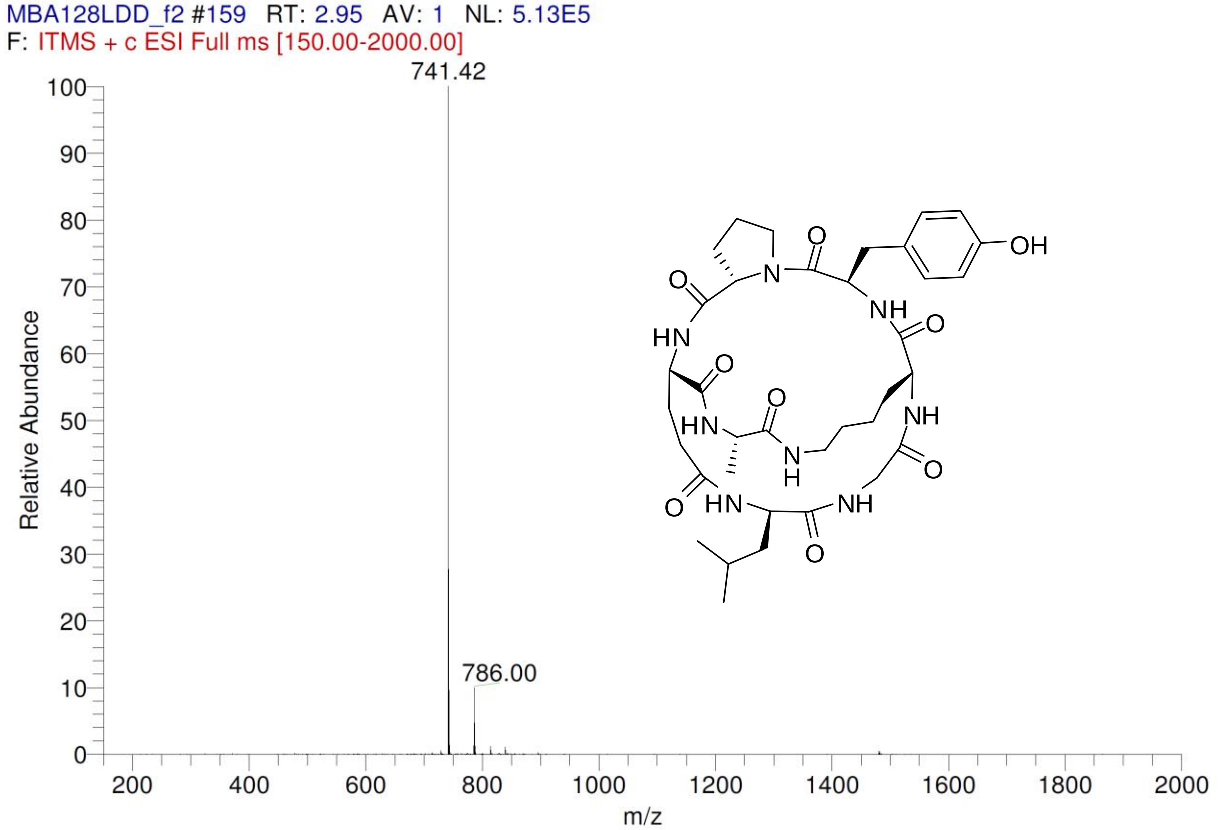

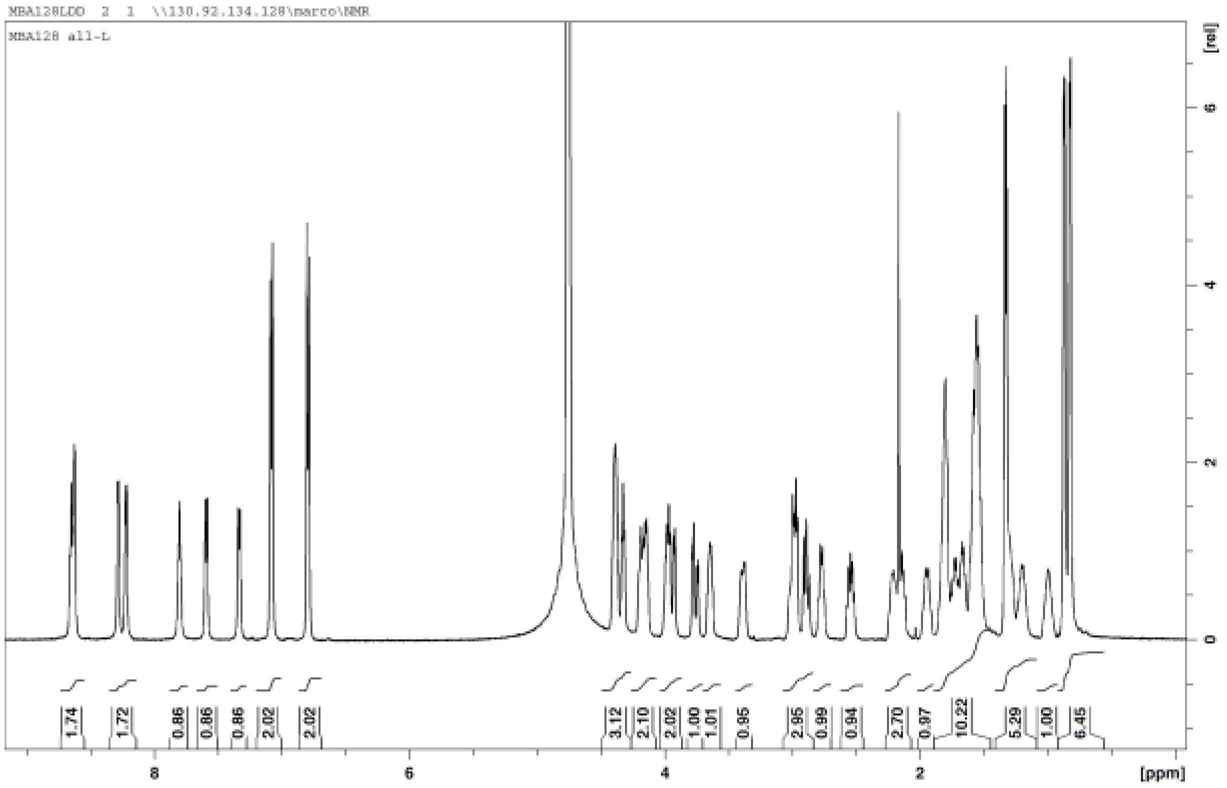

^{1}H-NMR (500 MHz, 90% H_2O/D_2O)			
residue	***proton***	***δ (ppm)***	***J (Hz)***
leu[1]	NH	8.23	^3J(NH–αCH) = 5.41
	αCH	4.15	
	βCH_2	1.55	
	γCH	1.55	
	$\delta' CH_3$	0.87	
	$\delta'' CH_3$	0.83	
Gly[2]	NH	8.65	
	α'CH	3.95	
	α''CH	3.76	
lys[3]	αNH	7.34	^3J(NH–αCH) = 7.19
	αCH	4.38	
	β'CH	1.82	
	β''CH	1.59	
	γ'CH	1.21	
	γ''CH	1.00	
	δ'CH	1.57	
	δ''CH	1.30	
	ε'CH	3.39	
	ε''CH	3.01	
	εNH	7.80	
tyr[4]	NH	8.63	^3J(NH–αCH) = 2.86
	αCH	4.40	
	β'CH	2.98	
	β''CH	2.89	
	2,6H	7.09	
	3,5H	6.80	
Pro[5]	αCH	4.33	
	βCH_2	1.81	
	γ'CH	1.73	
	γ''CH	1.67	
	δ'CH	3.65	
	δ''CH	2.78	
Glu[6]	NH	7.60	^3J(NH–αCH) = 7.59
	αCH	4.19	
	β'CH	2.22	
	β''CH	1.95	
	γ'CH	2.55	
	γ''CH	2.15	
Ala[7]	NH	8.29	^3J(NH–αCH) = 4.36
	αCH	3.99	
	βCH_3	1.33	

l[1]G*k*[2]YP*E*[1]a[2] (12)

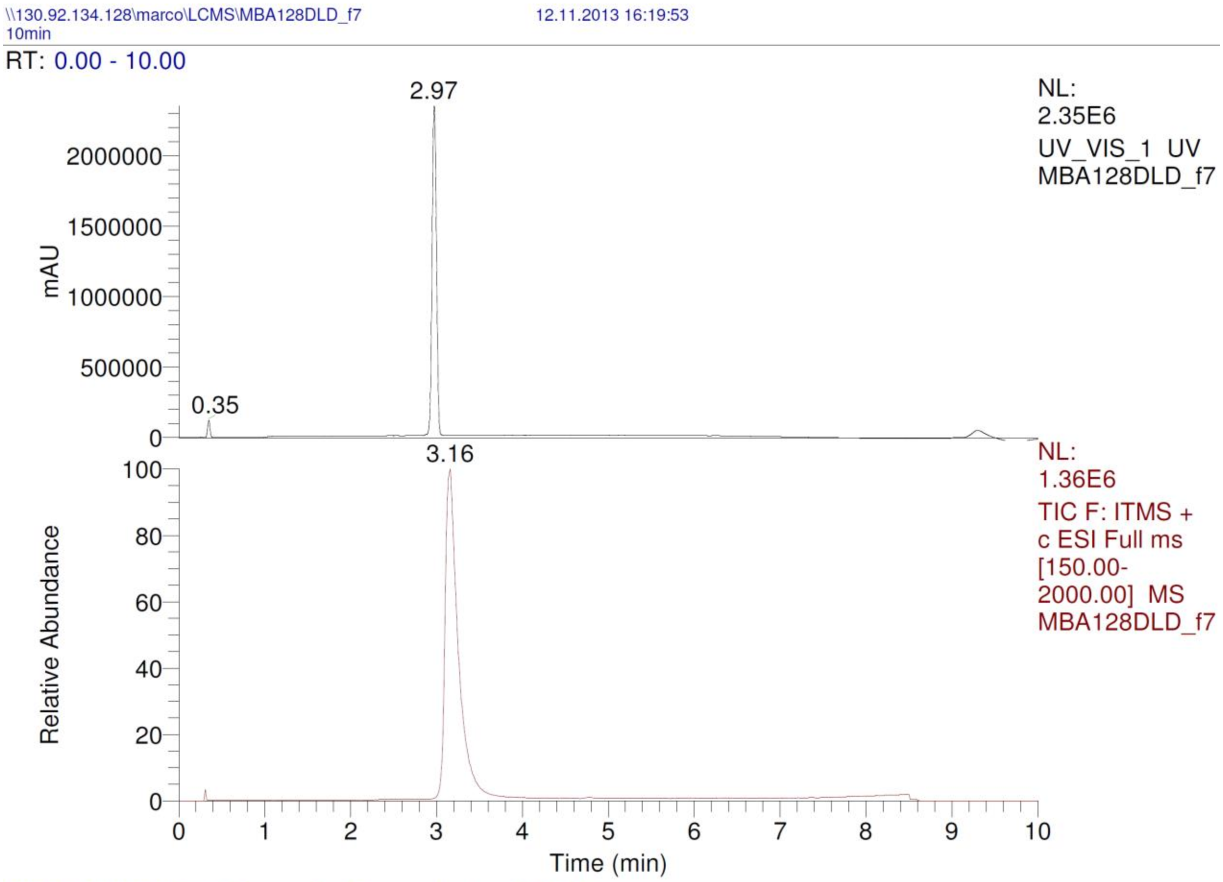

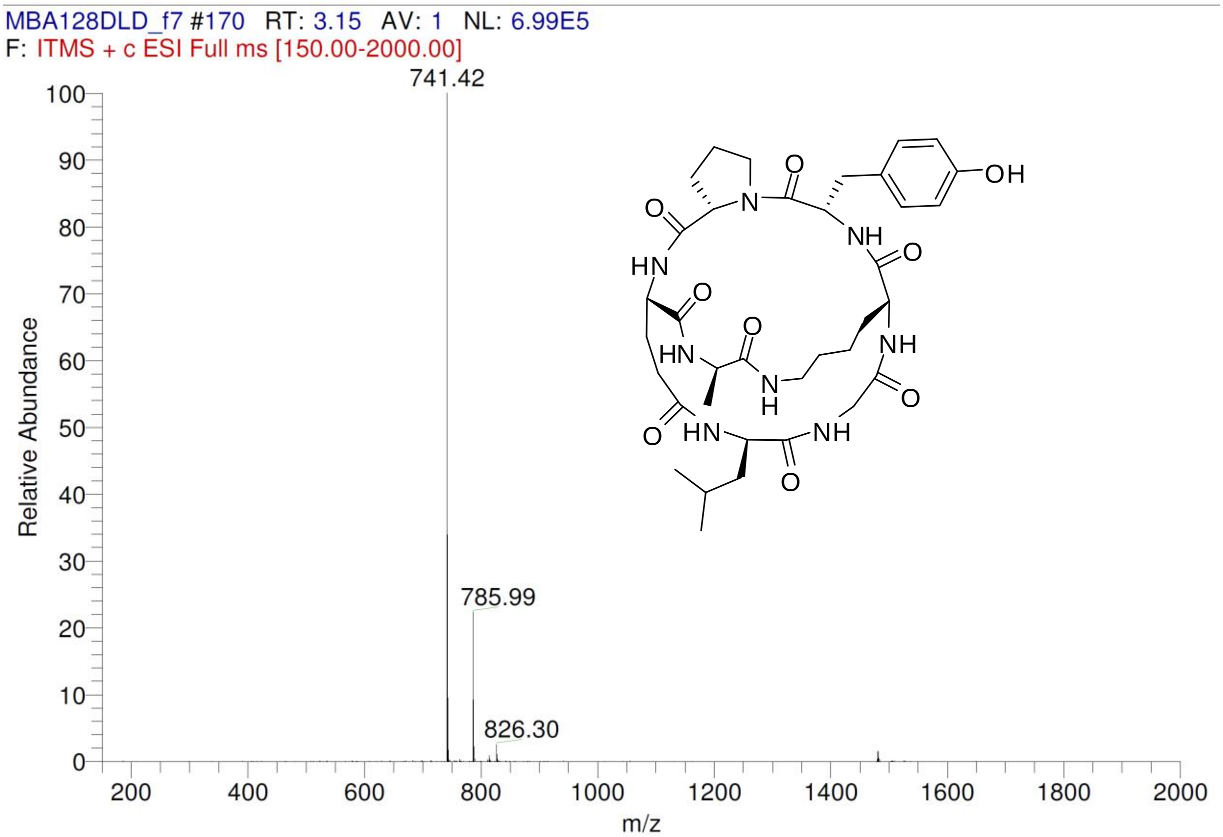

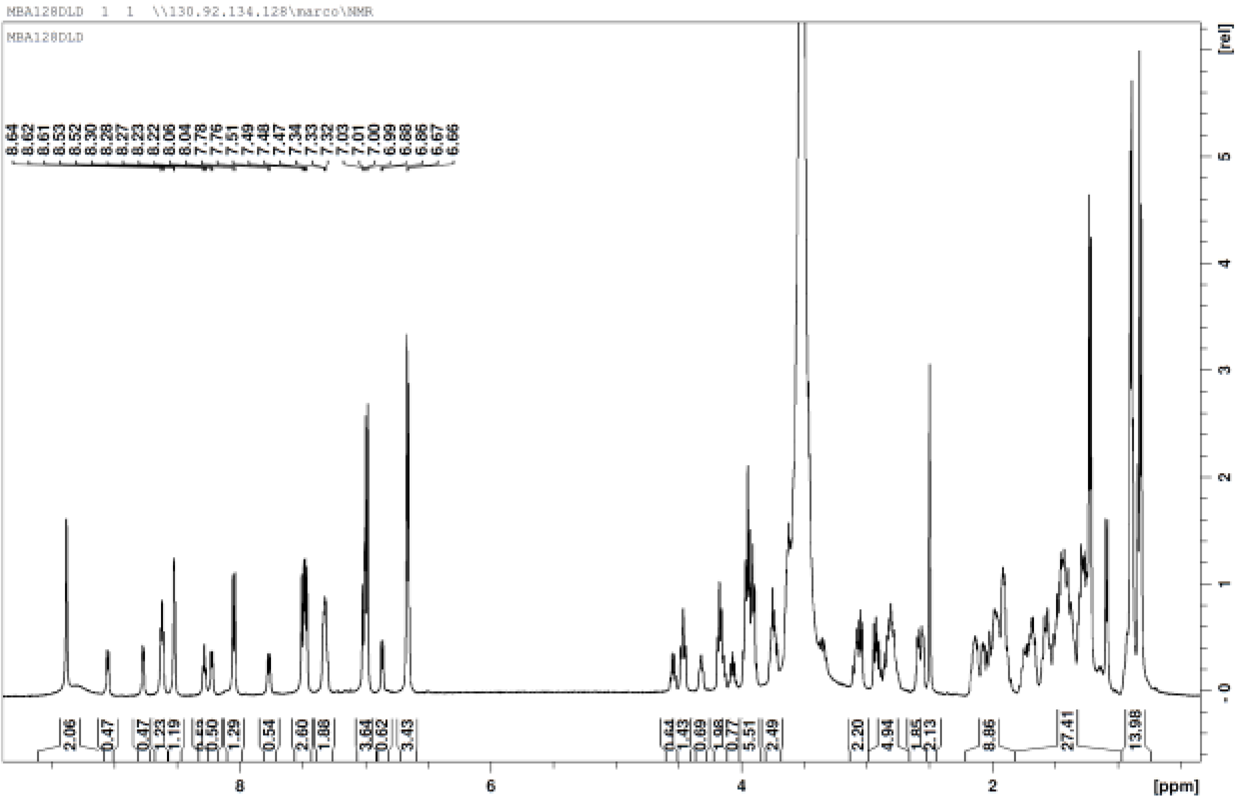

^{1}H-NMR (400 MHz, DMSO-d_6)				
residue	***proton***	***δ (ppm)*** **trans *conf.* (A)**	***δ (ppm)*** **cis *conf.* (B)**	***J (Hz)***
leu[1]	NH	8.53	8.23	^3J(NH–αCH) = 3.01 (A)<br>^3J(NH–αCH) = 6.40 (B)
Gly[2]	NH	8.62	8.28	
lys[3]	αNH	8.05	6.87	^3J(NH–αCH) = 8.26 (A)
	εNH	7.33	7.33	^3J(NH–αCH) = 7.06 (B)
Tyr[4]	NH	9.39	9.06	^3J(NH–αCH) = 1.11 (A)<br>^3J(NH–αCH) = 6.78 (B)
Glu[6]	NH	7.50	8.78	^3J(NH–αCH) = 8.62 (A)<br>^3J(NH–αCH) = 5.10 (B)
ala[7]	NH	7.48	7.77	^3J(NH–αCH) = 6.66 (A)<br>^3J(NH–αCH) = 8.22 (B)

L^{1}G*k*^{2}yP*E*^{1}a^2 (13).

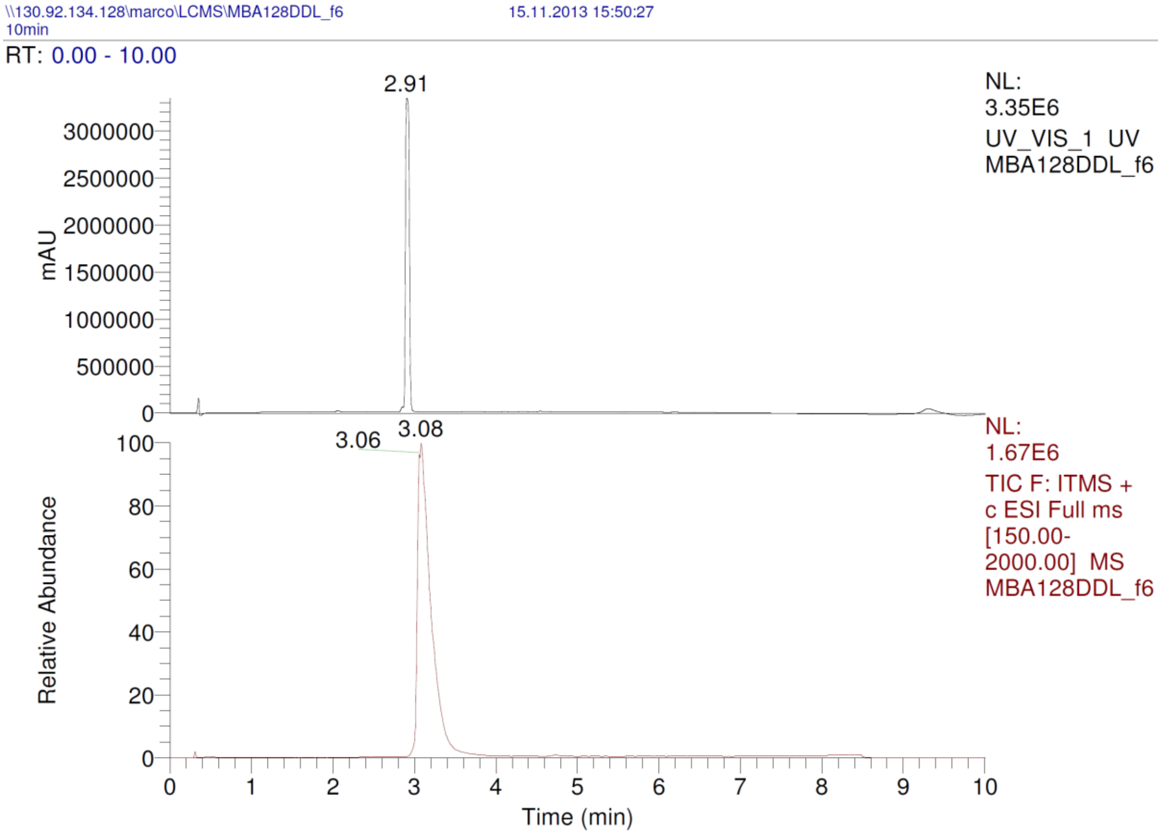

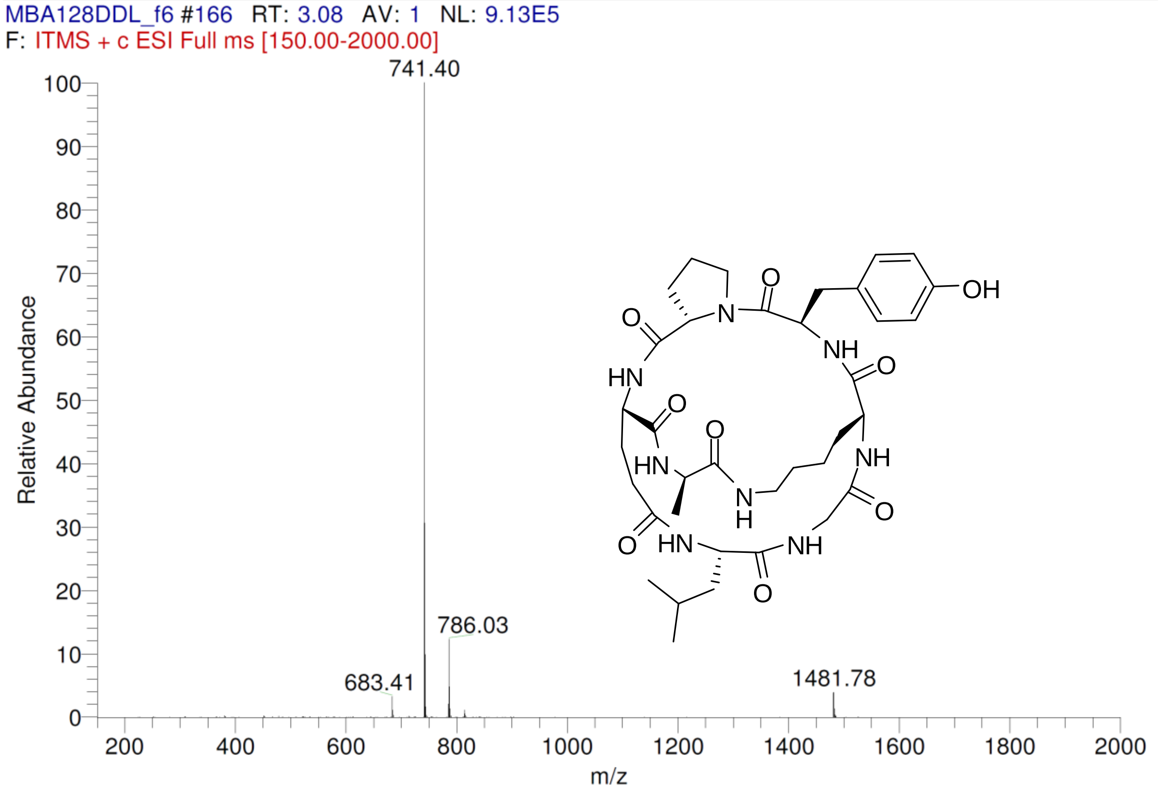

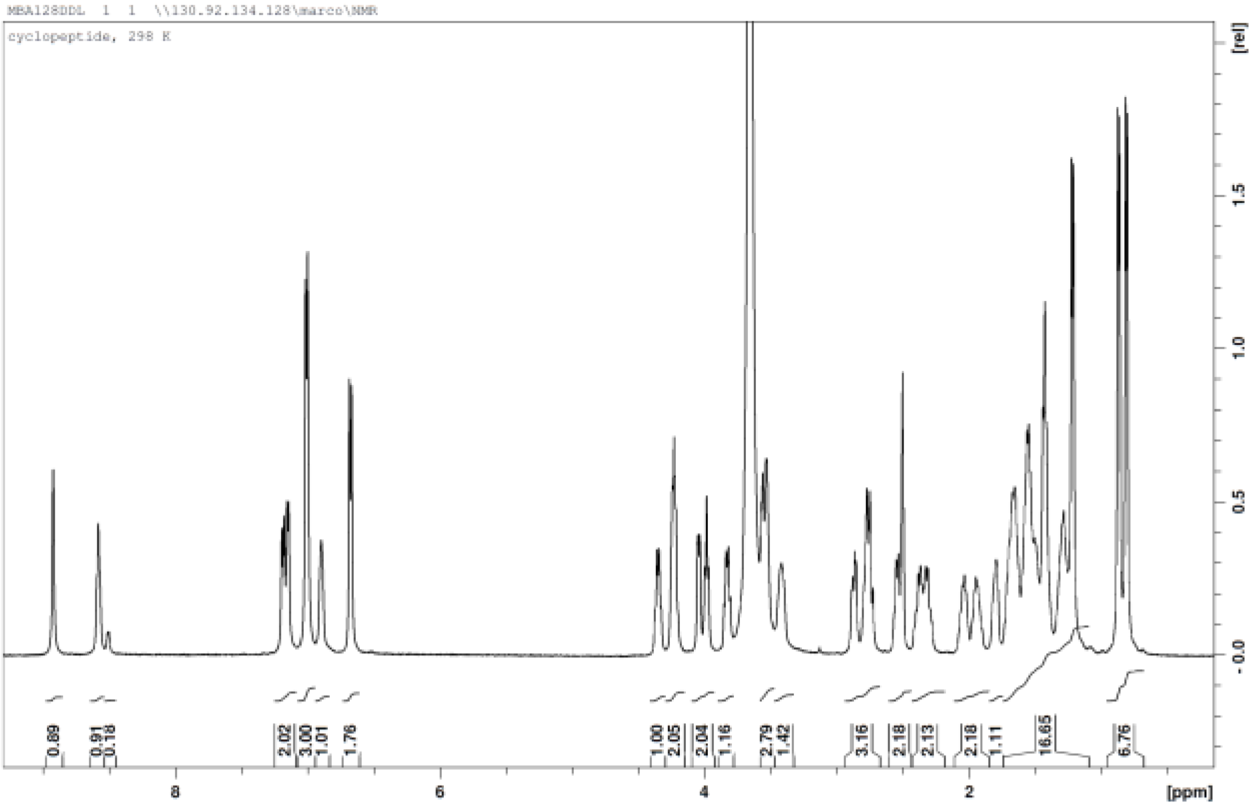

^{1}H-NMR (400 MHz, DMSO-d_6)

residue	***proton***	***δ (ppm)***	***J (Hz)***
Leu[1]	NH	8.51	^3J(NH–αCH) = 4.74
	αCH	3.99	
	βCH_2	1.43	
	γCH	1.49	
	$\delta' CH_3$	0.87	
	$\delta'' CH_3$	0.81	
Gly[2]	NH	8.58	
	α'CH	3.66	
	α"CH	3.55	
lys[3]	αNH	7.02	
	αCH	4.35	
	β'CH	1.57	
	β"CH	1.29	
	γCH_2	1.45	
	δ'CH	1.56	
	δ"CH	1.29	
	ε'CH	3.42	
	ε"CH	2.77	
	εNH	6.90	
tyr[4]	NH	8.93	
	αCH	4.24	
	β'CH	2.86	
	β"CH	2.75	
	2,6H	7.02	
	3,5H	6.68	
Pro[5]	αCH	4.04	
	β'CH	1.80	
	β"CH	1.70	
	γCH_2	1.65	
	δ'CH	3.54	
	δ"CH	2.54	
Glu[6]	NH	7.15	^3J(NH–αCH) = 6.39
	αCH	3.83	
	β'CH	2.04	
	β"CH	1.94	
	γCH_2	2.34	
ala[7]	NH	7.19	^3J(NH–αCH) = 8.20
	αCH	4.22	
	βCH_3	1.22	

l^{1}G*k*^{2}YP*E*^{1}A^2 (14).

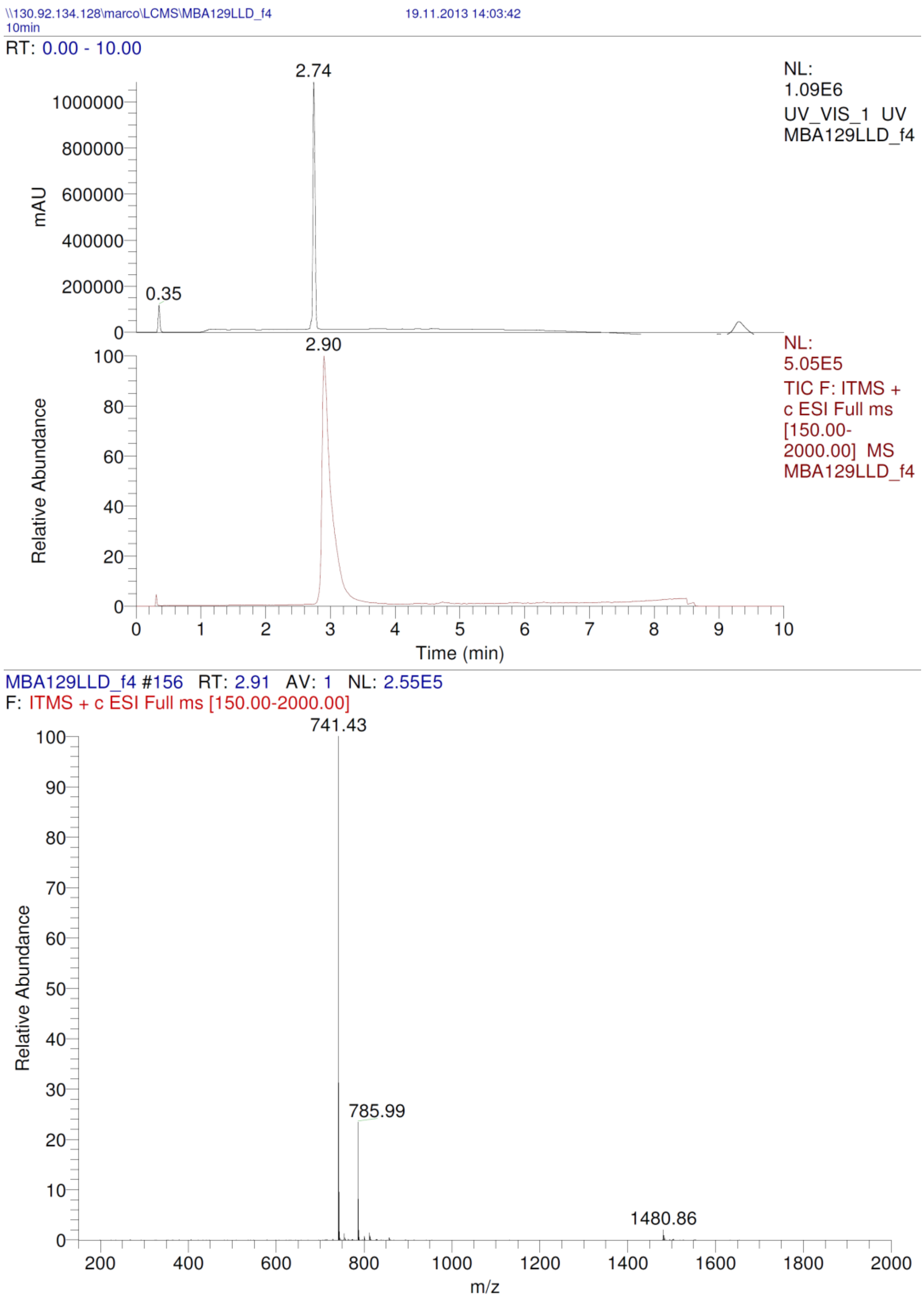

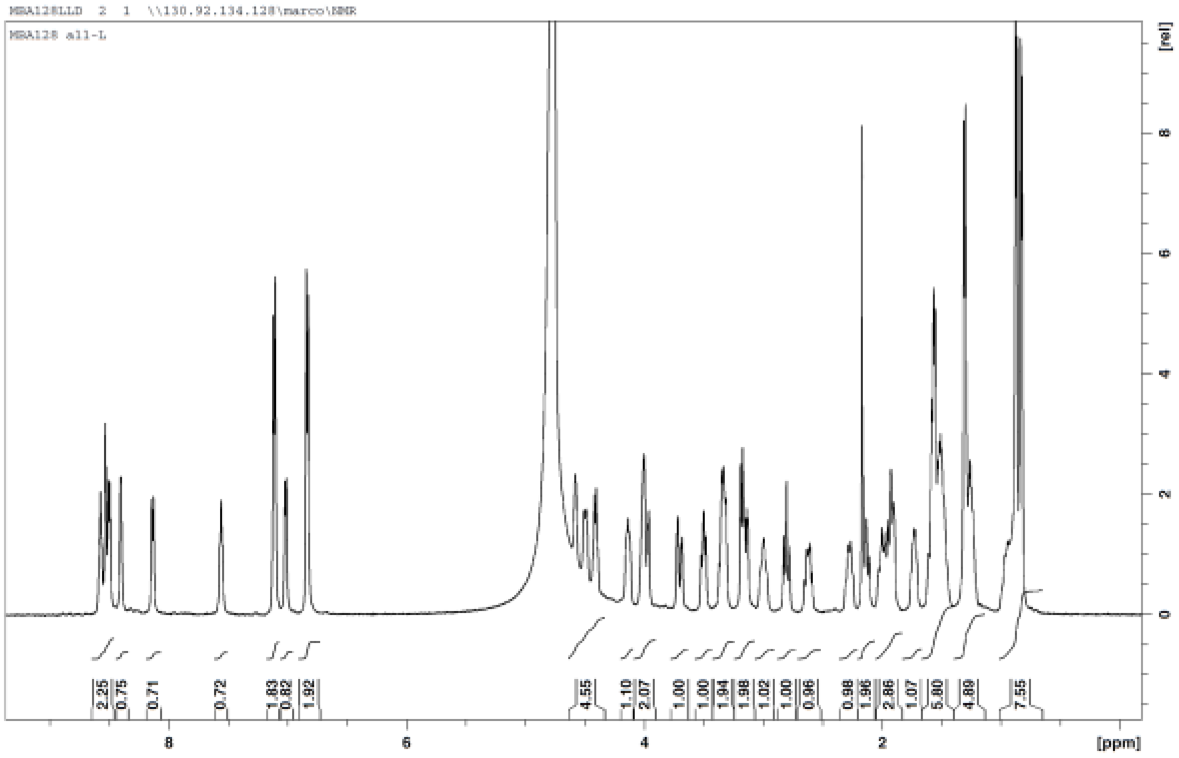

^{1}H-NMR (500 MHz, 90% H_2O/D_2O)			
residue	***proton***	***δ (ppm)***	***J (Hz)***
leu[1]	NH	8.14	^3J(NH–αCH) = 7.26
	αCH	4.41	
	βCH_2	1.56	
	γCH	1.52	
	$\delta' CH_3$	0.87	
	$\delta'' CH_3$	0.83	
Gly[2]	NH	8.58	
	α'CH	3.98	
	α''CH	3.70	
lys[3]	αNH	7.02	^3J(NH–αCH) = 6.08
	αCH	4.58	
	β'CH	1.99	
	β''CH	1.58	
	γCH_2	0.92	
	δ'CH	1.48	
	δ''CH	1.26	
	ε'CH	3.33	
	ε''CH	3.00	
	εNH	7.56	
Tyr[4]	NH	8.53	
	αCH	4.49	
	β'CH	3.14	
	β''CH	2.80	
	2,6H	7.11	
	3,5H	6.84	
Pro[5]	αCH	3.18	
	β'CH	1.91	
	β''CH	1.25	
	γ'CH	1.73	
	γ''CH	1.45	
	δ'CH	3.50	
	δ''CH	3.34	
Glu[6]	NH	8.50	^3J(NH–αCH) = 7.02
	αCH	4.13	
	β'CH	2.27	
	β''CH	1.94	
	γ'CH	2.62	
	γ''CH	2.13	
Ala[7]	NH	8.41	^3J(NH–αCH) = 3.35
	αCH	4.02	
	βCH_3	1.31	

L^{1}G*k*^{2}yP*E*^{1}A^{2} (15).

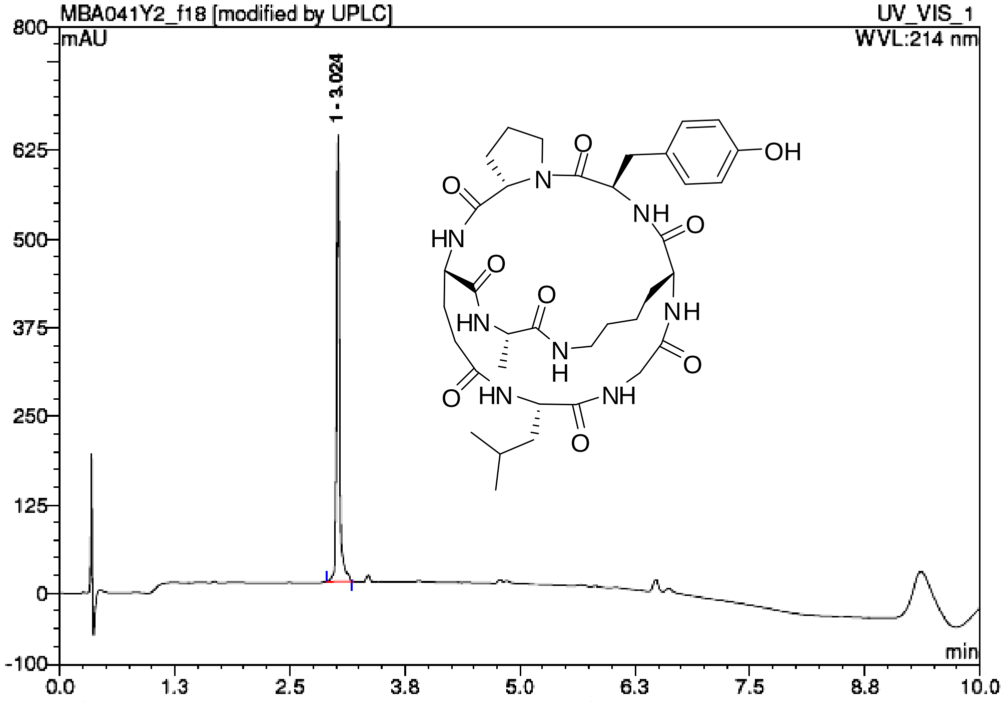

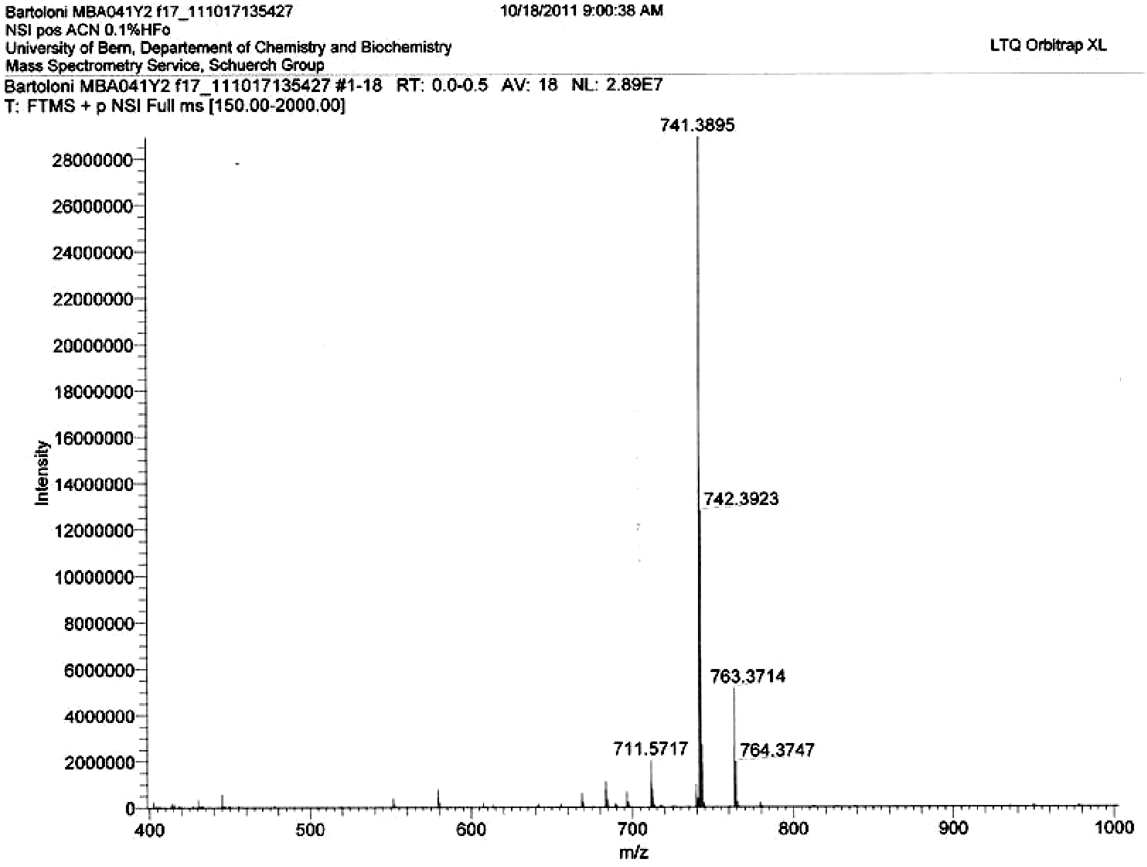

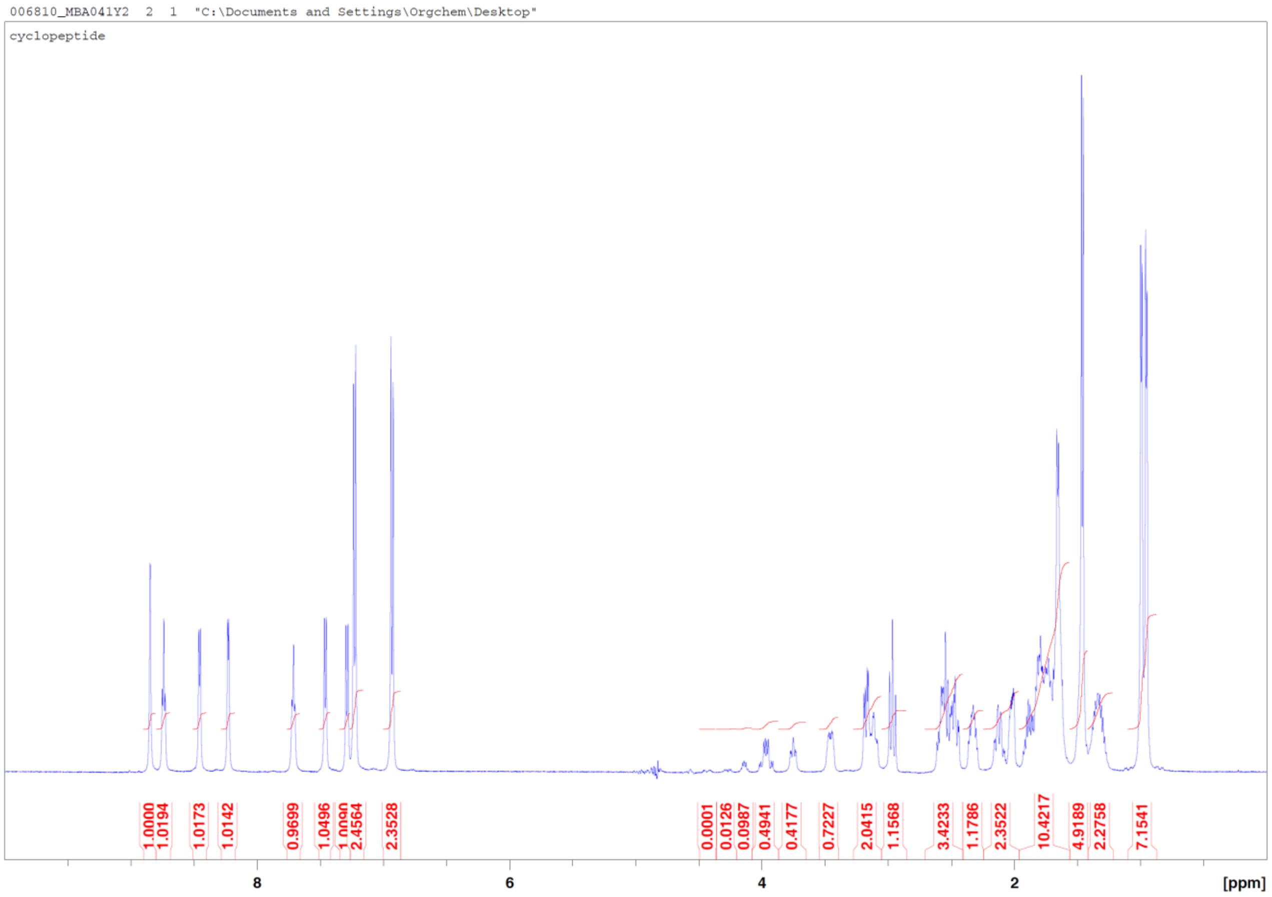

^{1}H-NMR (500 MHz, H_2O/D_2O 9:1)			
residue	***proton***	***δ (ppm)***	***J (Hz)***
Leu[1]	NH	8.39	^{3}J(NH–αCH) = 6.32
	αCH	4.34	
	$βCH_2$	1.58	
	γCH	1.58	
	$δ'CH_3$	0.91	
	$δ''CH_3$	0.87	
Gly[2]	NH	8.65	
	$αCH_2$	3.89	
lys[3]	αNH	7.21	^{3}J(NH–αCH) = 8.41
	αCH	4.52	
	β'CH	1.80	
	β"CH	1.72	
	$γCH_2$	1.26	
	δ'CH	1.65	
	δ"CH	1.40	
	ε'CH	3.39	
	ε"CH	3.04	
	εNH	7.64	
tyr[4]	NH	8.77	^{3}J(NH–αCH) = 1.45
	αCH	4.46	
	β'CH	3.08	
	β"CH	2.88	
	2,6H	7.13	
	3,5H	6.85	
Pro[5]	αCH	4.38	
	β'CH	1.94	
	β"CH	1.72	
	γ'CH	1.65	
	γ"CH	1.56	
	δ'CH	3.66	
	δ"CH	2.46	
Glu[6]	NH	7.38	^{3}J(NH–αCH) = 7.56
	αCH	4.20	
	β'CH	2.25	
	β"CH	2.04	
	γ'CH	2.49	
	γ"CH	2.39	
Ala[7]	NH	8.16	^{3}J(NH–αCH) = 4.95
	αCH	4.06	
	$βCH_3$	1.38	

L[1]G*k*[2]YP*E*[1]a[2] (16).

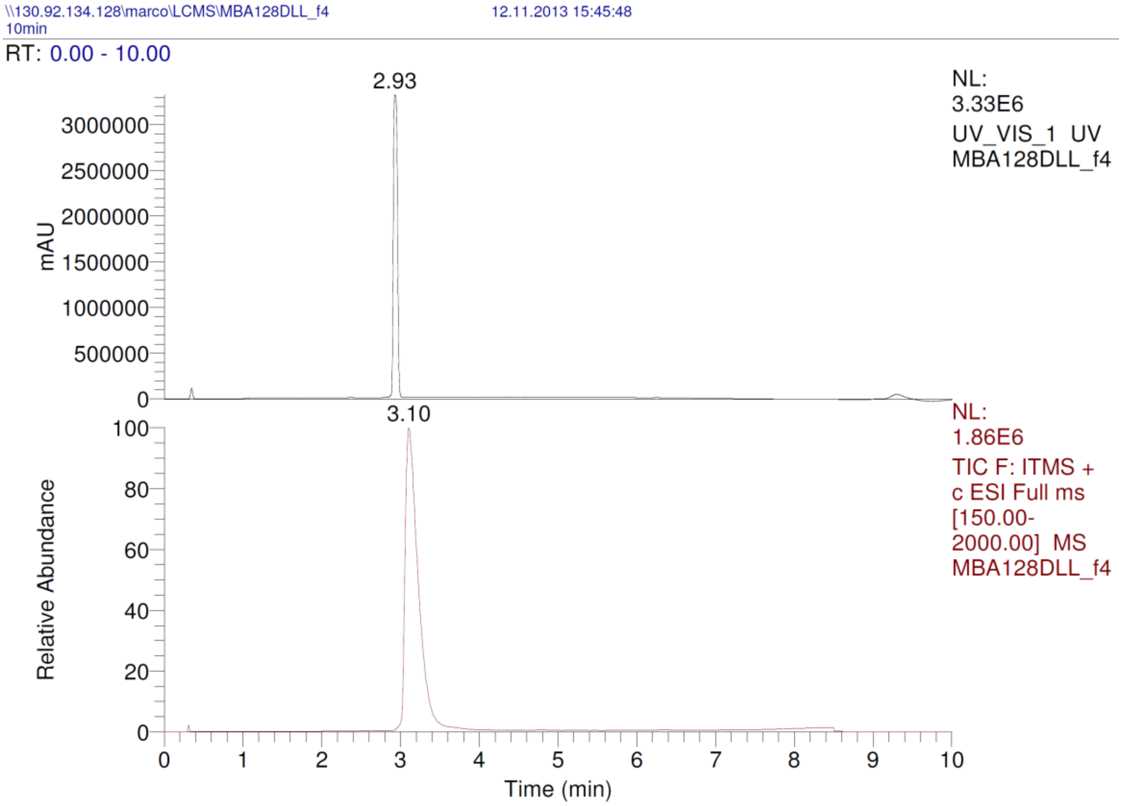

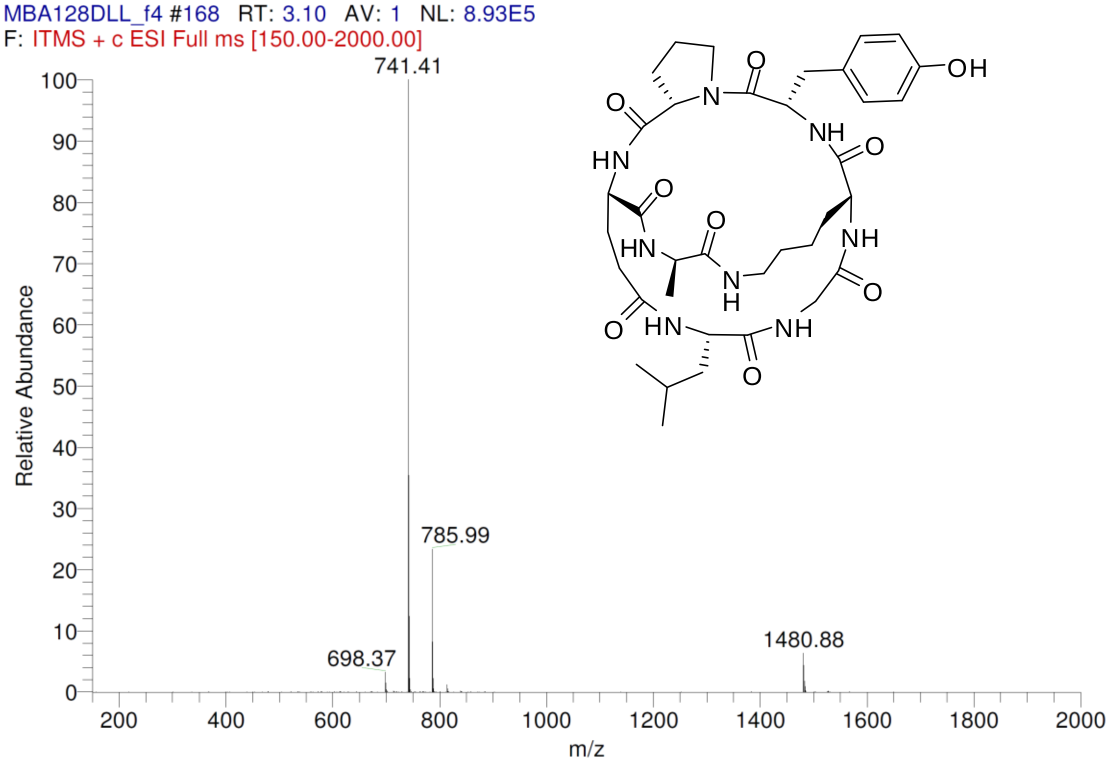

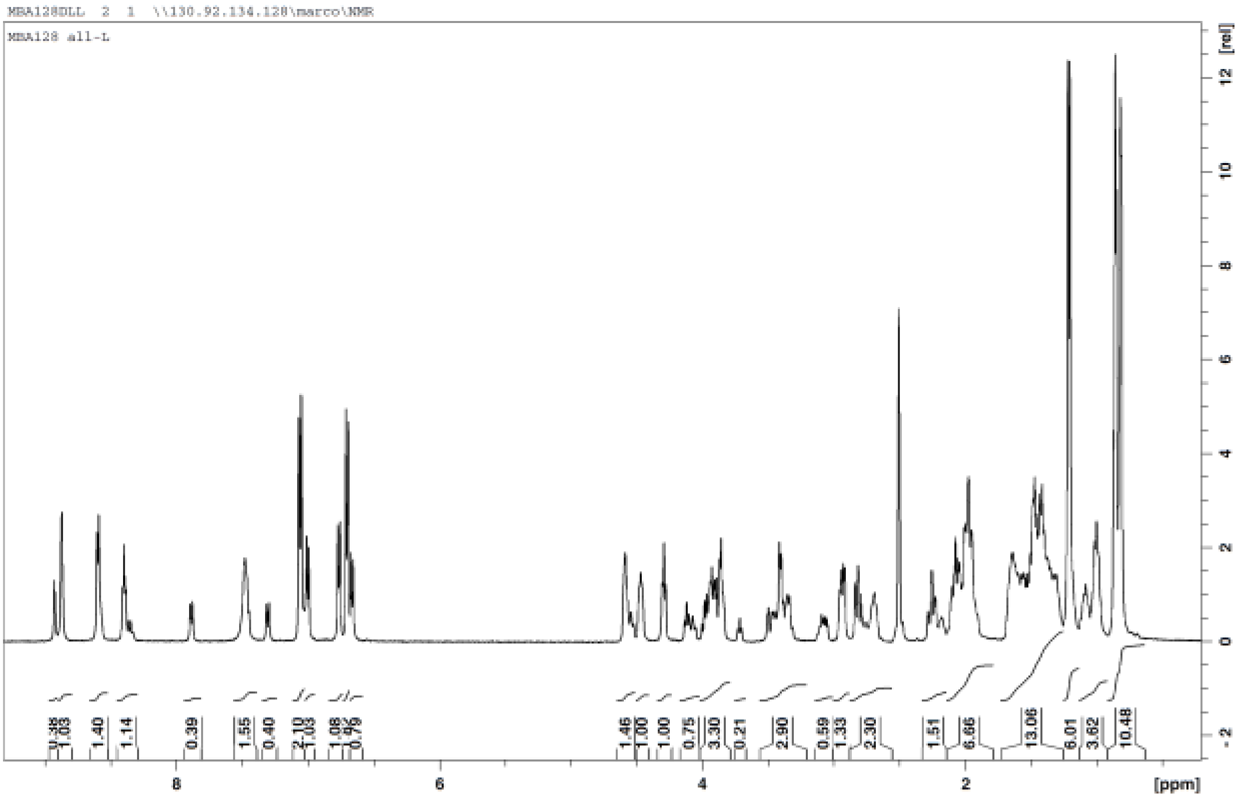

^{1}H-NMR (500 MHz, d_6-DMSO)				
residue	***proton***	***δ (ppm)*** **cis *conf.* (A)**	***δ (ppm)*** **trans *conf.* (B)**	***J (Hz)***
Leu[1]	NH	8.36	8.32	^3J(NH–αCH) = 8.15 (A)<br>^3J(NH–αCH) = 3.24 (B)
Gly[2]	NH	8.40	8.59	
lys[3]	αNH	6.77	7.89	^3J(NH–αCH) = 7.55 (A)<br>^3J(NH–αCH) = 8.48 (B)
	εNH	7.48	7.03	
Tyr[4]	NH	8.87	8.93	
Glu[6]	NH	8.60	7.31	^3J(NH–αCH) = 6.49 (A)<br>^3J(NH–αCH) = 9.10 (B)
ala[7]	NH	*n.d.*	7.46	^3J(NH–αCH) = *n.d.* (A)<br>^3J(NH–αCH) = 5.94 (B)

L^{1}G*k*^{2}YP*E*^{1}A^2 (17).

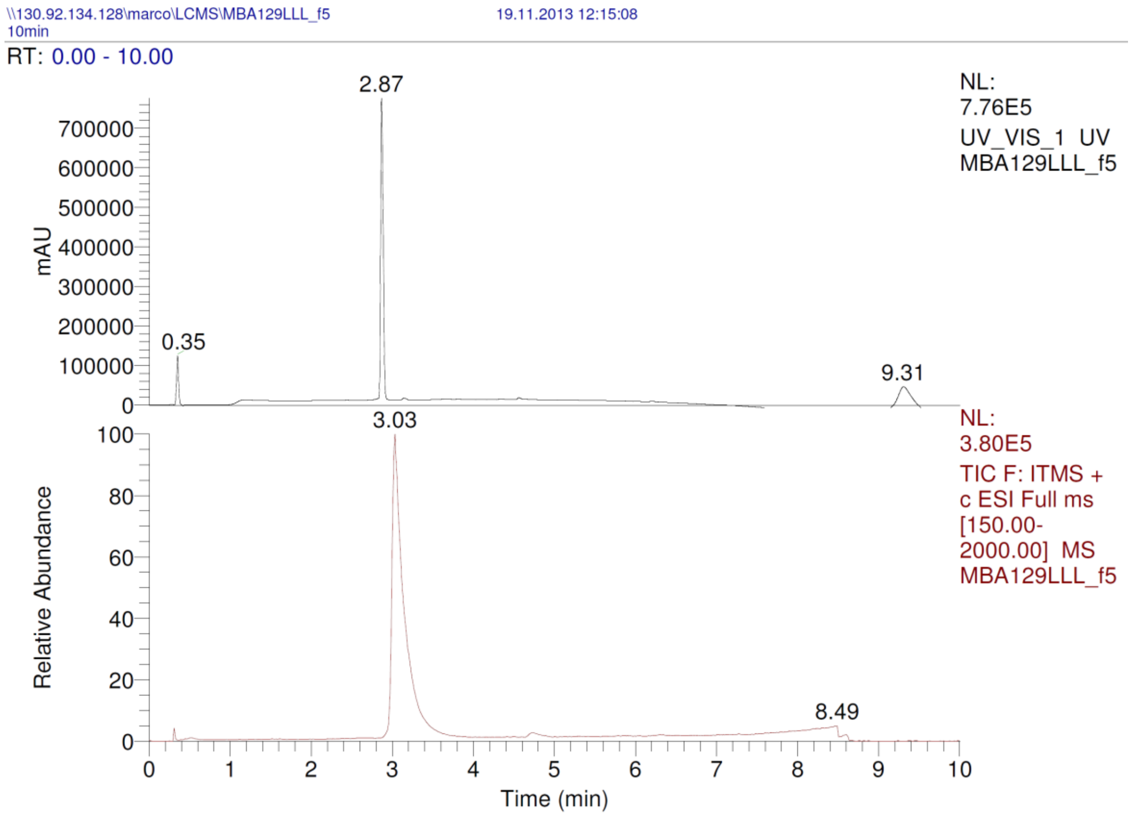

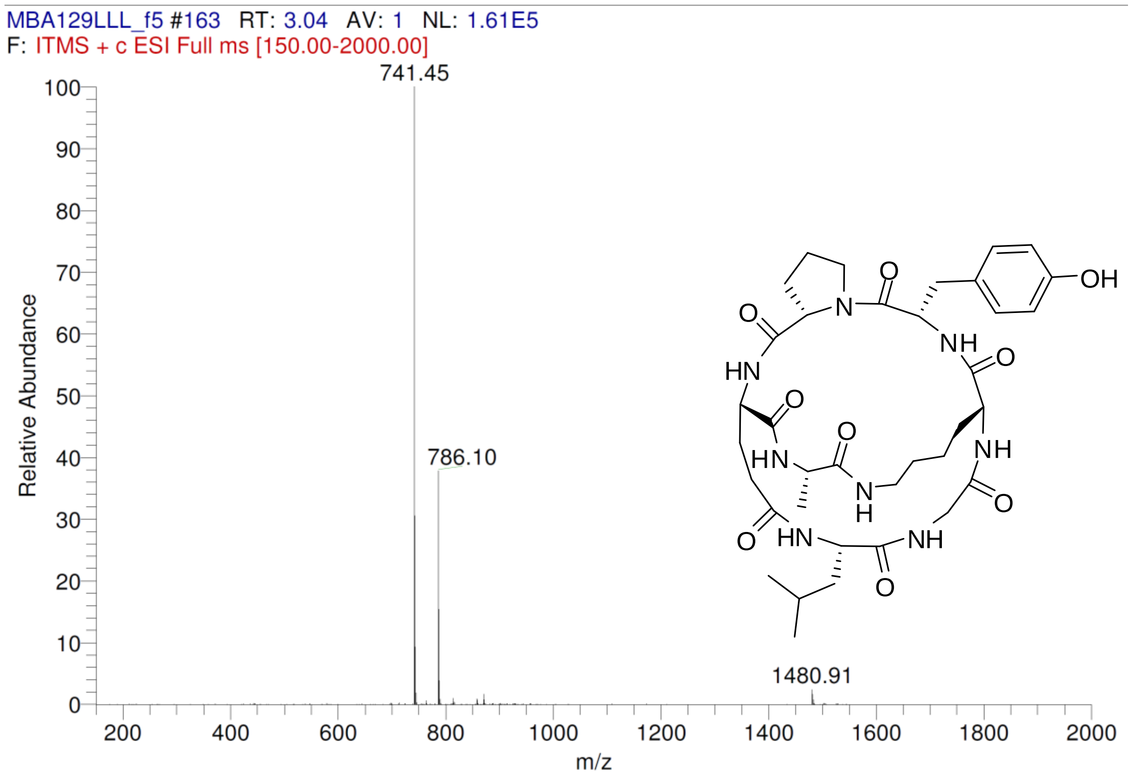

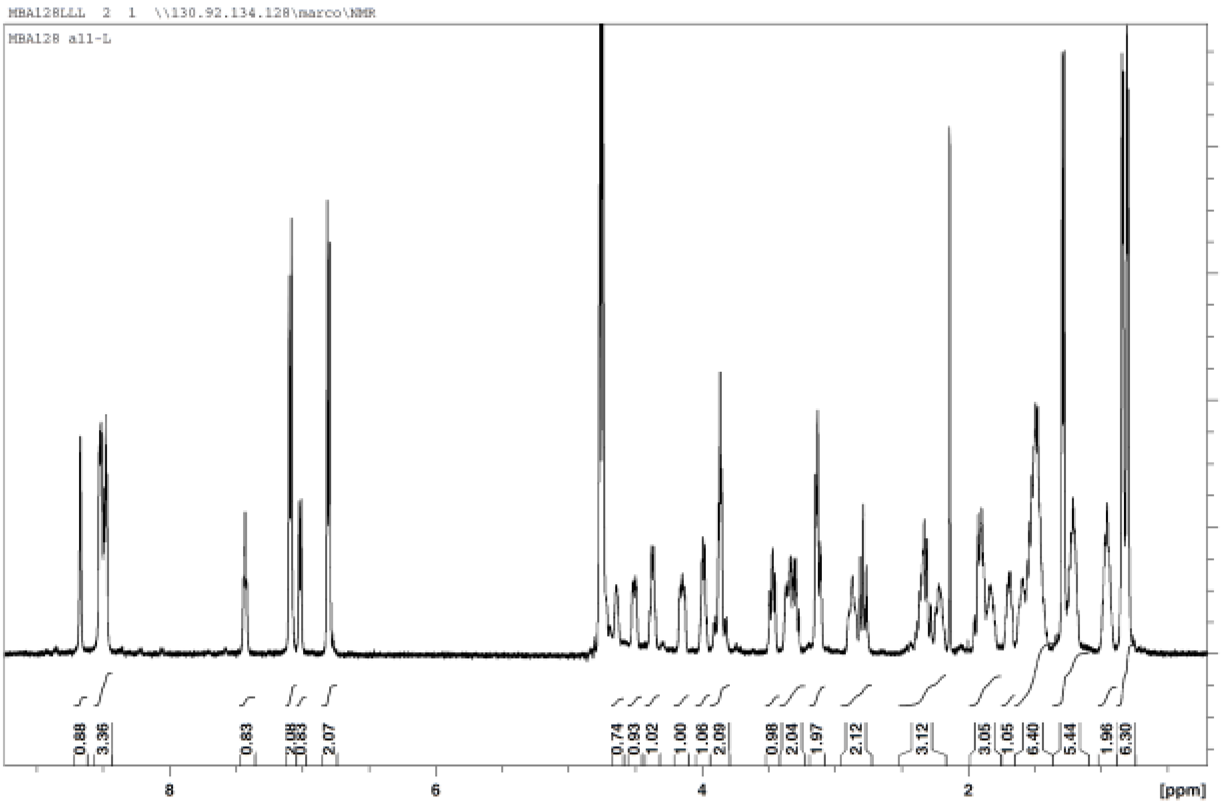

^{1}H-NMR (500 MHz, H_2O/D_2O 9:1)

residue	***proton***	***δ (ppm)***	***J (Hz)***
Leu[1]	NH	8.52	^3J(NH–αCH) = 3.50
	αCH	4.37	
	βCH_2	1.50	
	γCH		
	$\delta' CH_3$	0.84	
	$\delta'' CH_3$	0.80	
Gly[2]	NH	8.49	
	αCH_2	3.86	
lys[3]	αNH	7.02	^3J(NH–αCH) = 7.45
	αCH	4.64	
	β'CH		
	β"CH		
	γCH_2		
	δ'CH		
	δ"CH		
	ε'CH	3.35	
	ε"CH	2.87	
	εNH	7.43	
Tyr[4]	NH	8.67	
	αCH	4.51	
	β'CH	3.12	
	β"CH	2.79	
	2,6H	7.09	
	3,5H	6.81	
Pro[5]	αCH	3.14	
	β'CH		
	β"CH		
	γ'CH		
	γ"CH		
	δ'CH	3.47	
	δ"CH	3.30	
Glu[6]	NH	8.53	^3J(NH–αCH) = 2.57
	αCH	4.15	
	β'CH		
	β"CH		
	γCH_2		
Ala[7]	NH	8.48	^3J(NH–αCH) = 4.32
	αCH	3.99	
	βCH_3	1.28	

L[1]G*K*[2]YP*E*[1]A[2] (18).

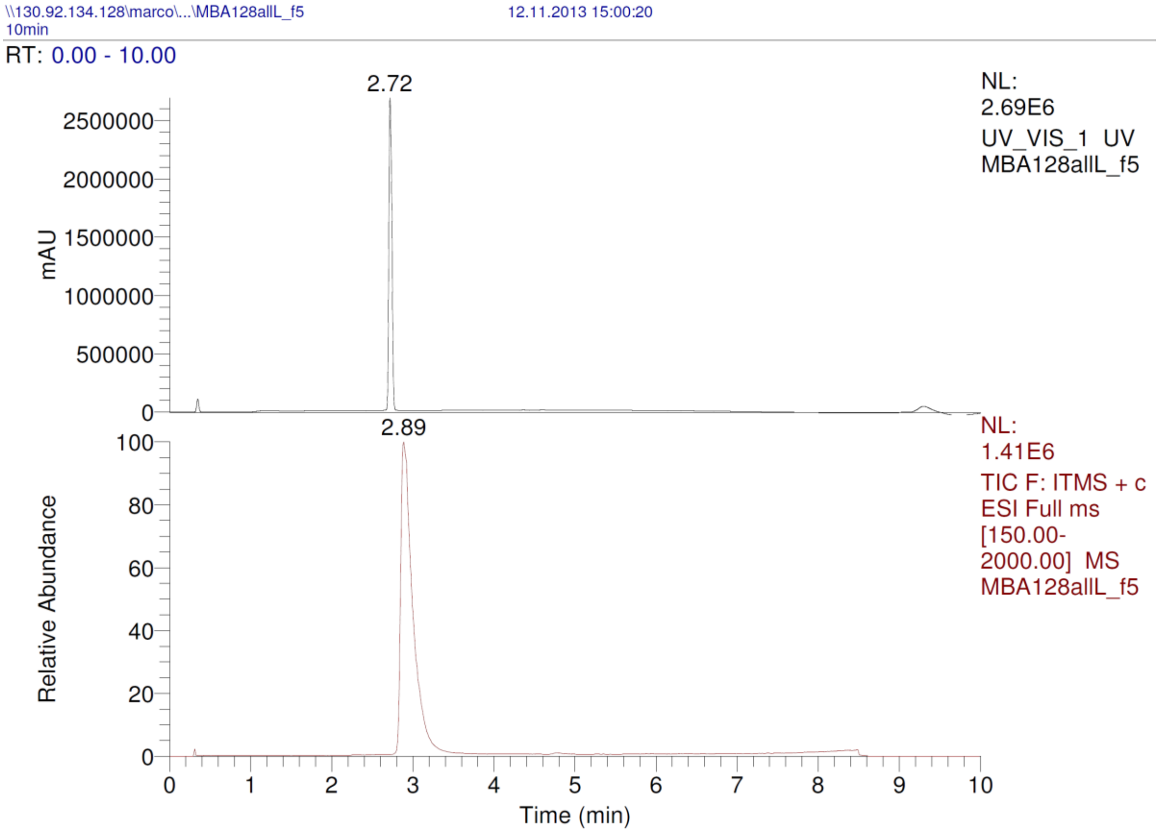

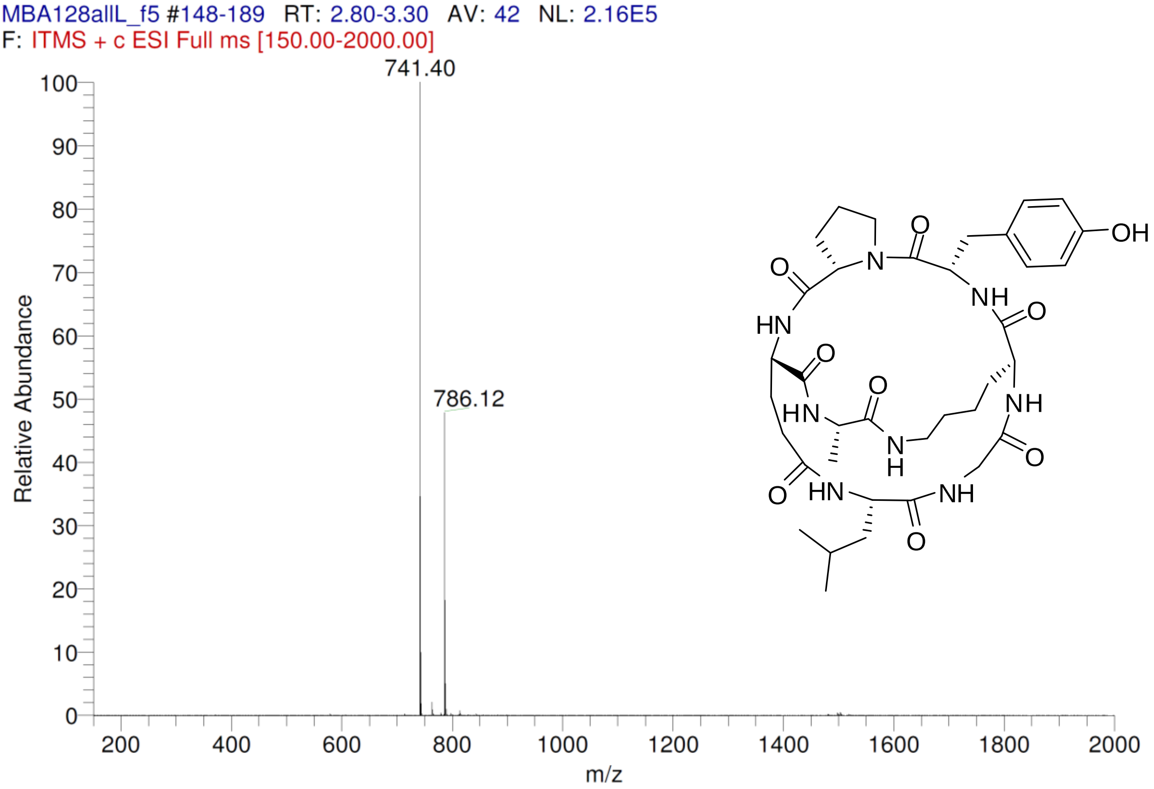

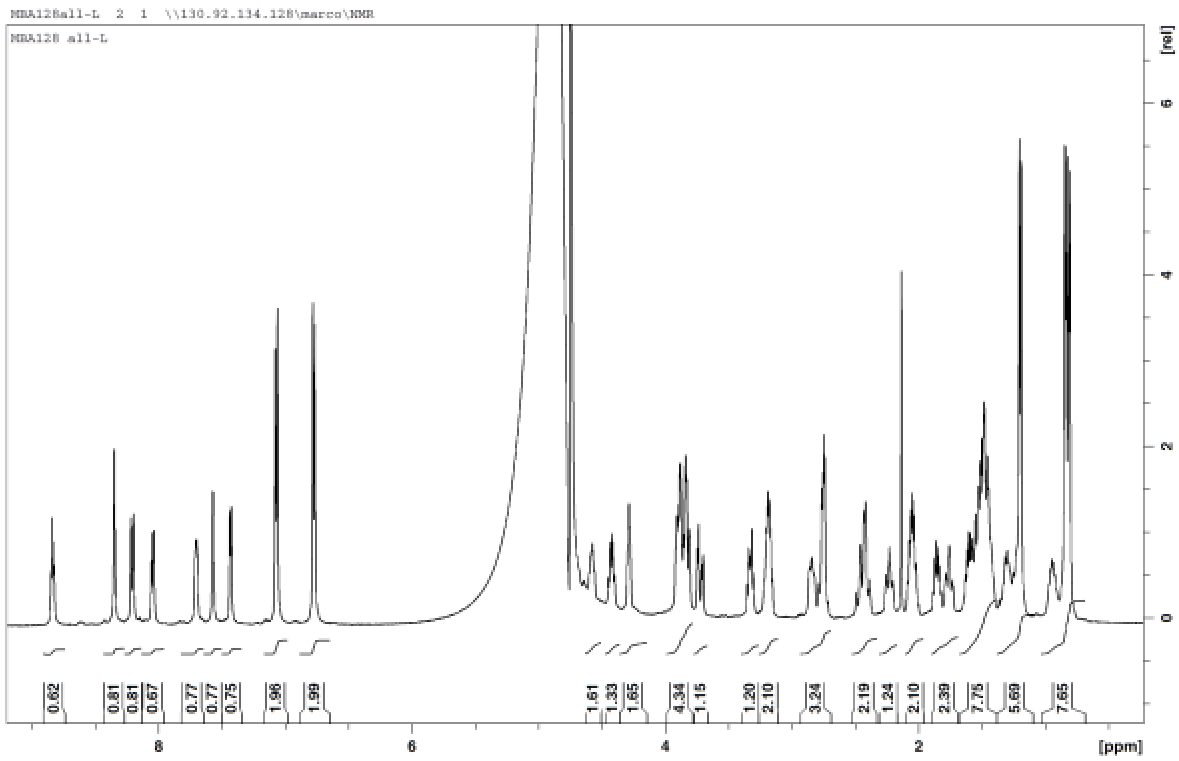

**^{1}H-NMR (500 MHz, 90% H_2O/D_2O)**			
residue	***proton***	***δ (ppm)***	***J (Hz)***
Leu^1	NH	8.05	^3J(NH–αCH) = 8.15
	αCH	4.57	
	βCH$_2$	1.51	
	γCH	1.52	
	δ'CH$_3$	0.84	
	δ''CH$_3$	0.81	
Gly^2	NH	8.84	
	α'CH	3.90	
	α''CH	3.72	
Lys^3	αNH	7.44	^3J(NH–αCH) = 5.86
	αCH	4.29	
	β'CH	2.06	
	β''CH	1.48	
	γ'CH	1.22	
	γ''CH	0.94	
	δ'CH	1.43	
	δ''CH	1.30	
	ε'CH	3.20	
	ε''CH	7.70	
	εNH	7.70	
Tyr^4	NH	8.21	^3J(NH–αCH) = 9.45
	αCH	4.43	
	βCH$_2$	2.75	
	2,6H	7.07	
	3,5H	6.78	
Pro^5	αCH	3.85	
	β'CH	2.05	
	β''CH	1.86	
	γ'CH	1.60	
	γ''CH	1.50	
	δ'CH	3.33	
	δ''CH	3.18	
Glu^6	NH	8.35	
	αCH	3.82	
	β'CH	2.23	
	β''CH	1.76	
	γCH$_2$	2.43	
Ala^7	NH	7.58	^3J(NH–αCH) = 2.44
	αCH	3.89	
	βCH$_3$	1.20	

k^{1}G*k*^{2}fP*E*^{1}a^2 (19).

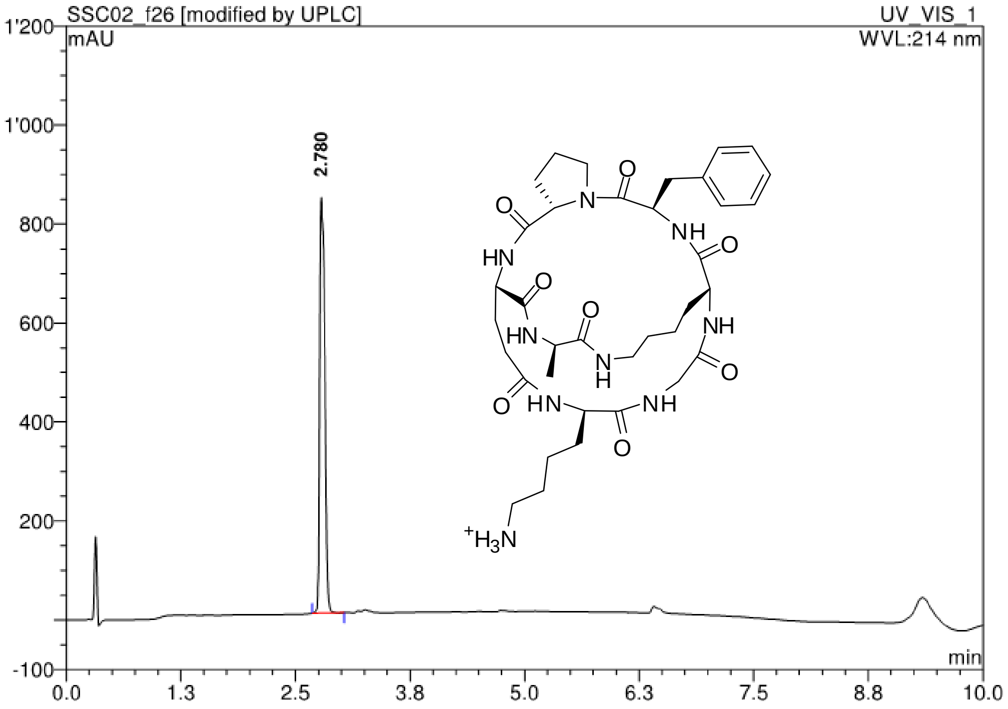

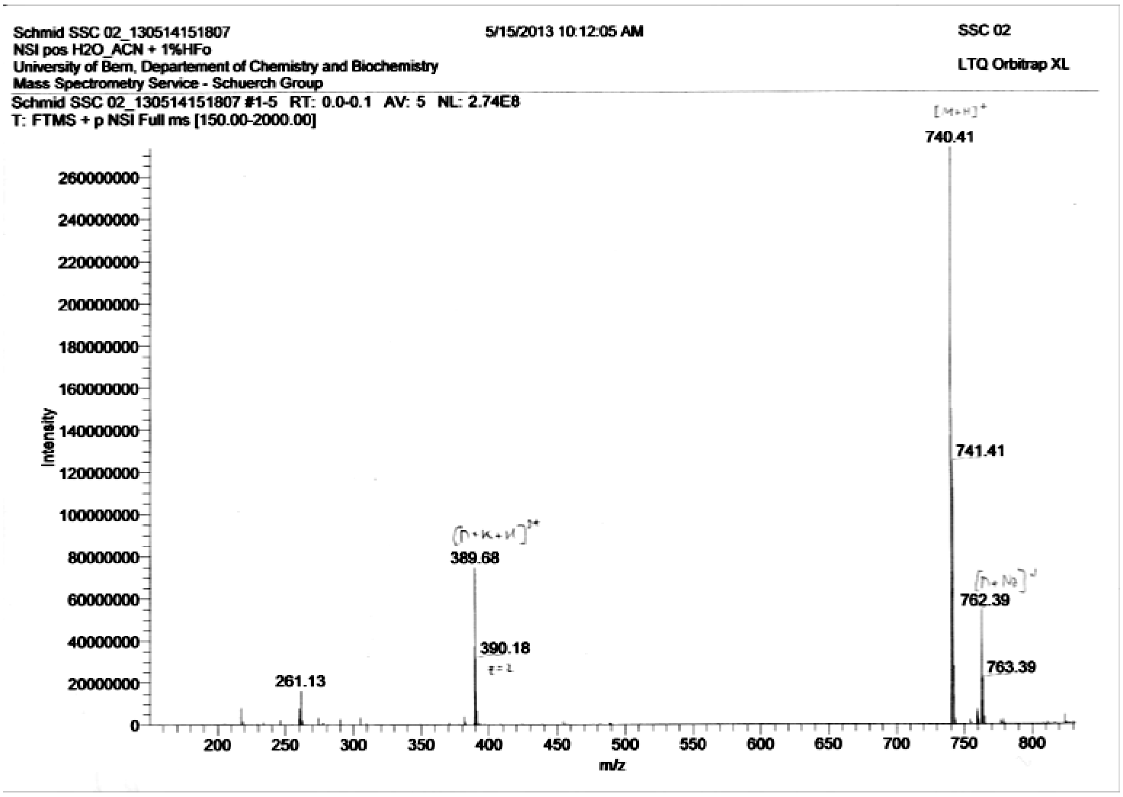

l[1]S*k*[2]fP*E*[1]a[2] (20).

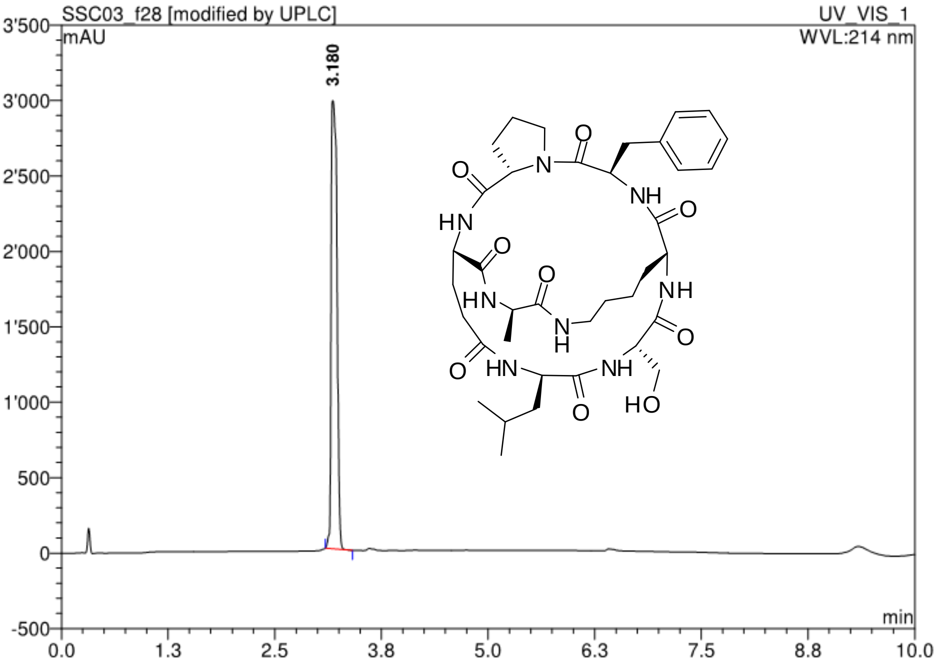

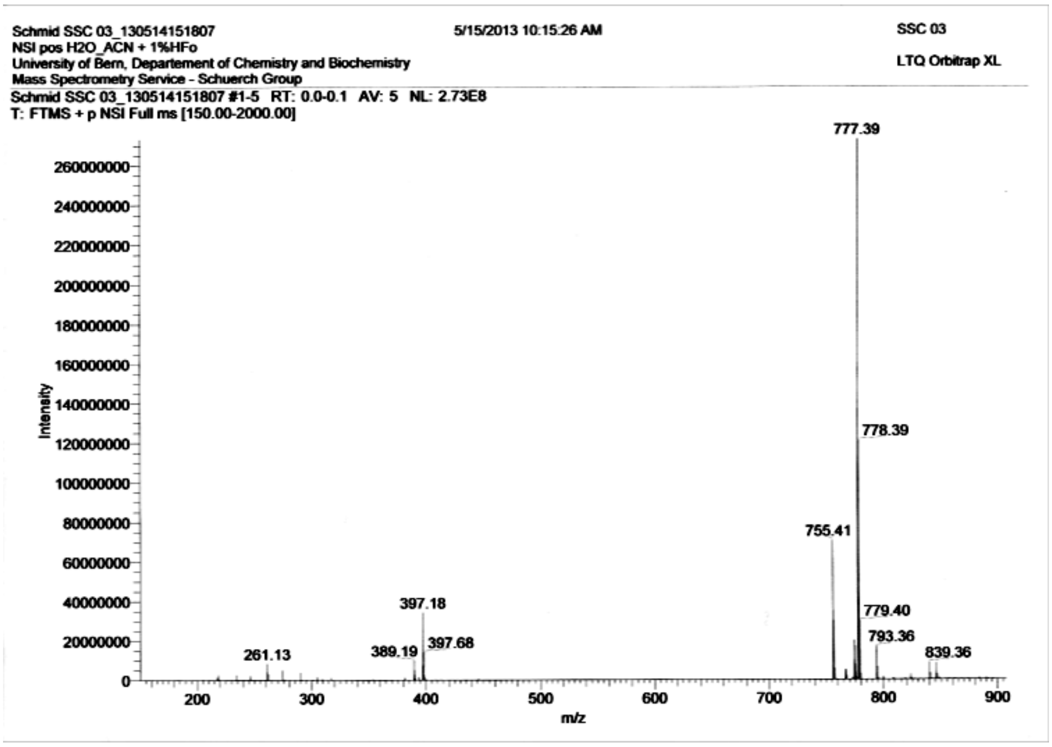

l[1]G*k*[2]fK*E*[1]a[2] (21).

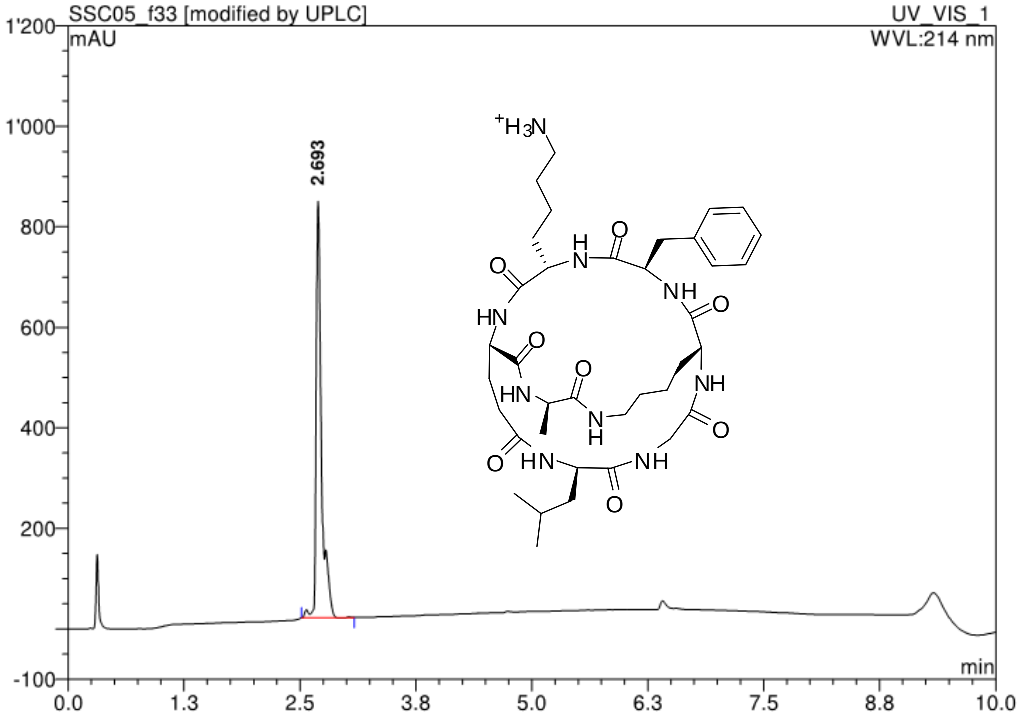

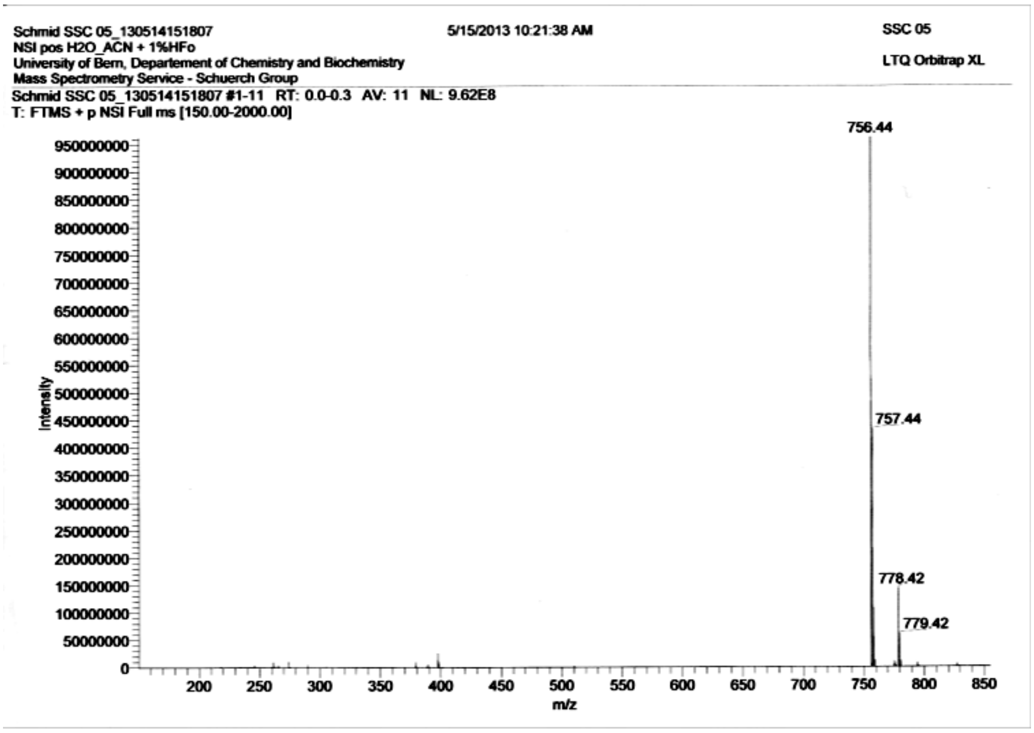

k[1]G*k*[2]eP*E*[1]c[2] (22). From 2CT-Cl resin (200 mg, 1.3 mmol/g), **22** was obtained as a white solid after preparative RP-HPLC (6.4 mg, 7.4 µmol, 2.3%). Analytical RP-HPLC: t_R = 1.92 min (A/D 100:0 to 0:100 in 7.5 min, λ = 214 nm). MS (ESI+): $C_{32}H_{51}N_9O_{10}S$ calc./obs. 754.36/754.35 $[M+H]^+$, 776.34/776.34 $[M+Na]^+$, 396.65/396.65 $[M+K+H]^{2+}$.

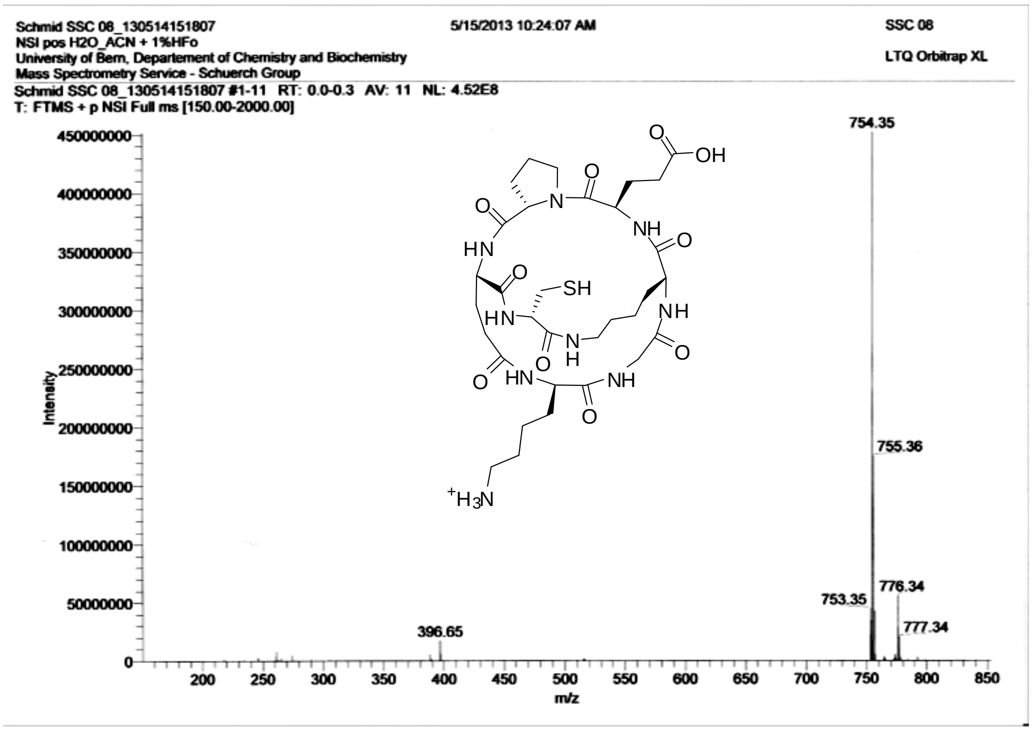

K[1]G*k*[2]fP*E*[1]A[2] (23).

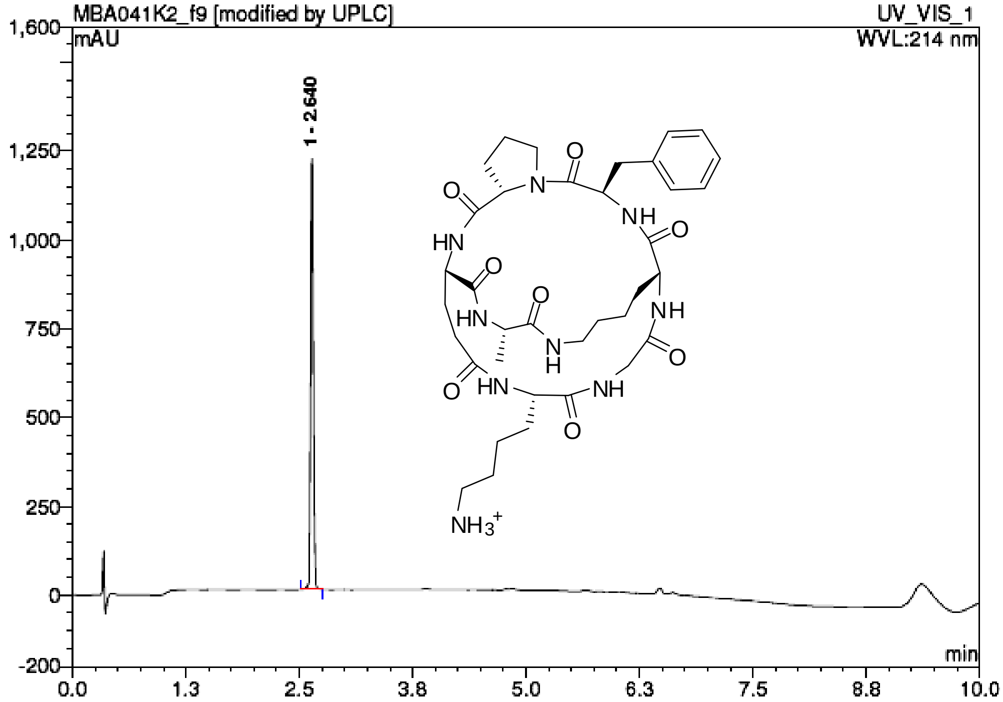

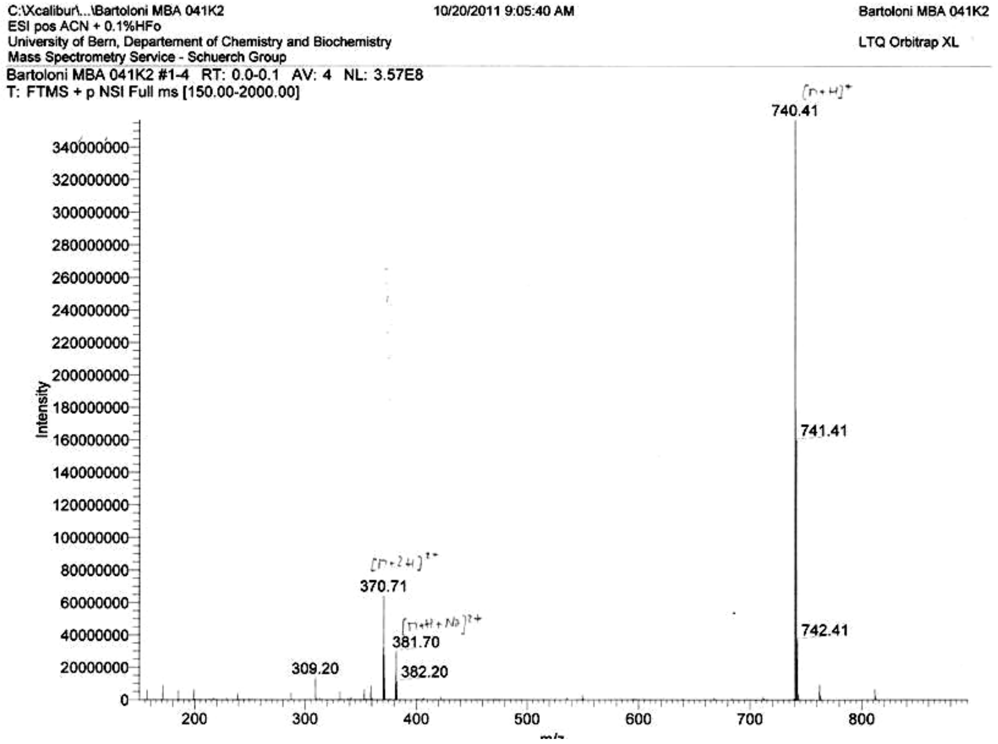

L[1]S*k*[2]fP*E*[1]A[2] (24).

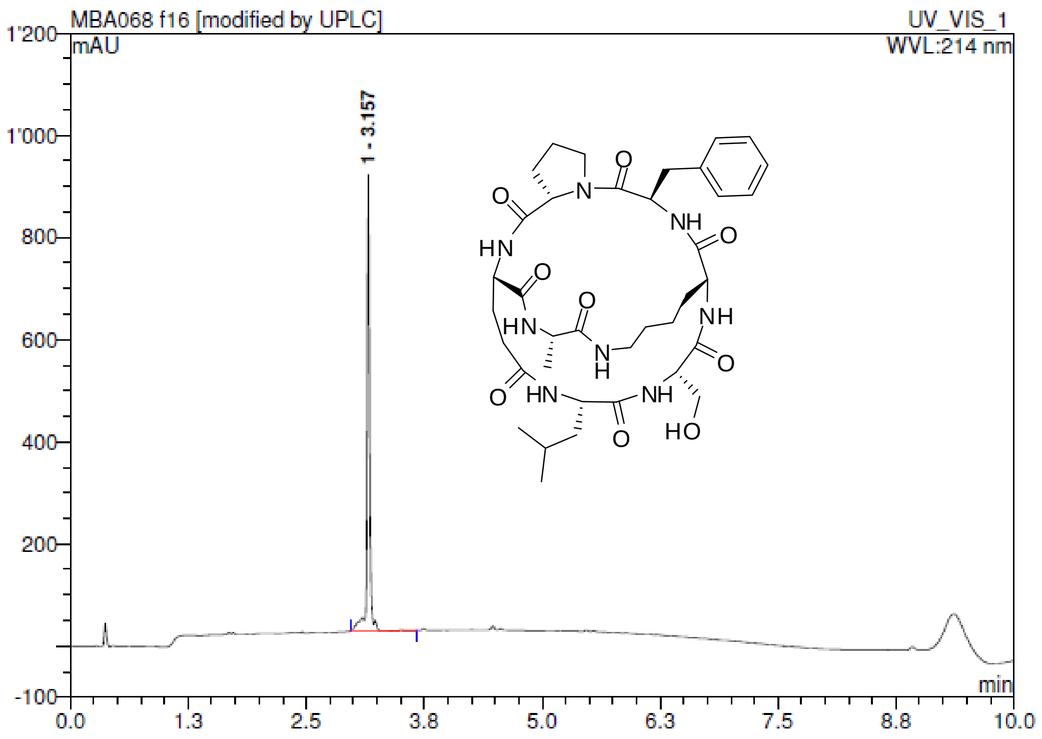

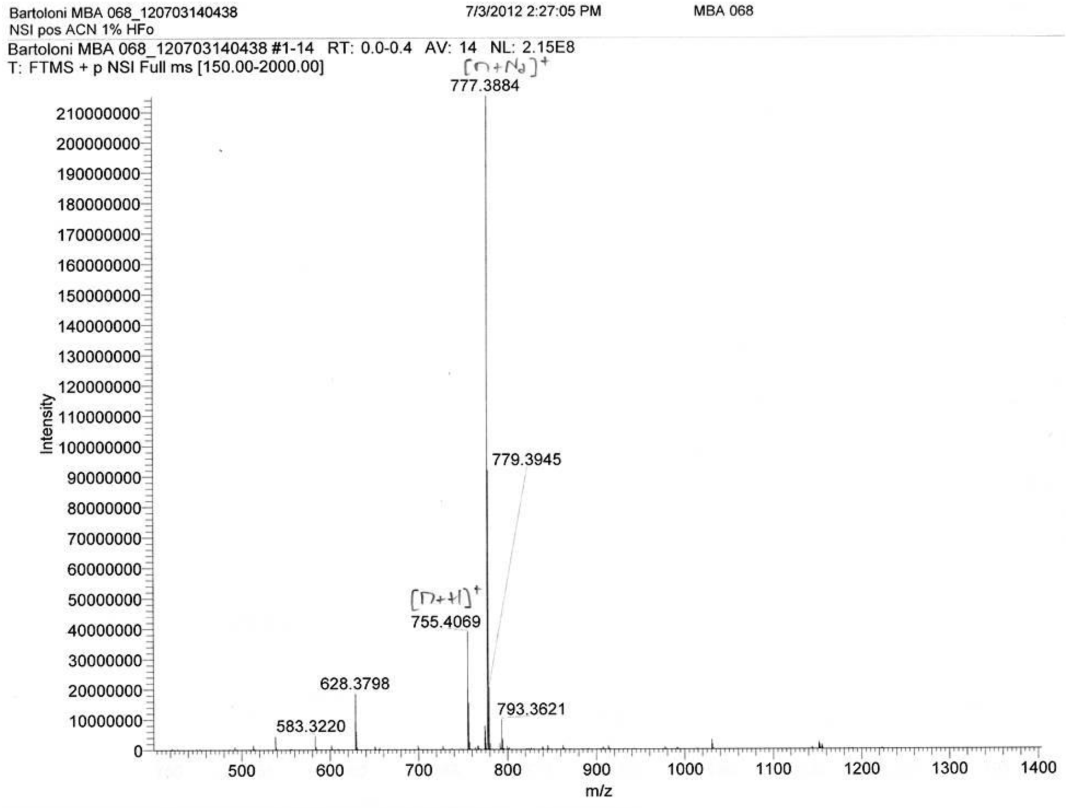

L[1]G*k*[2]fK*E*[1]A[2] (25).

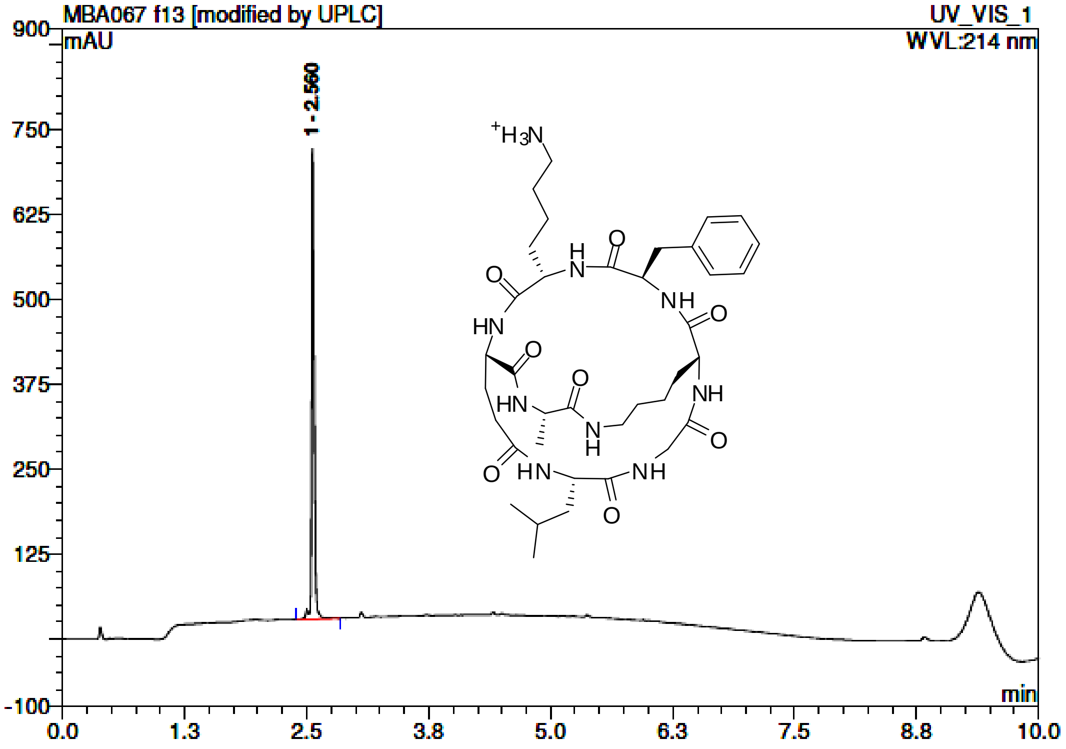

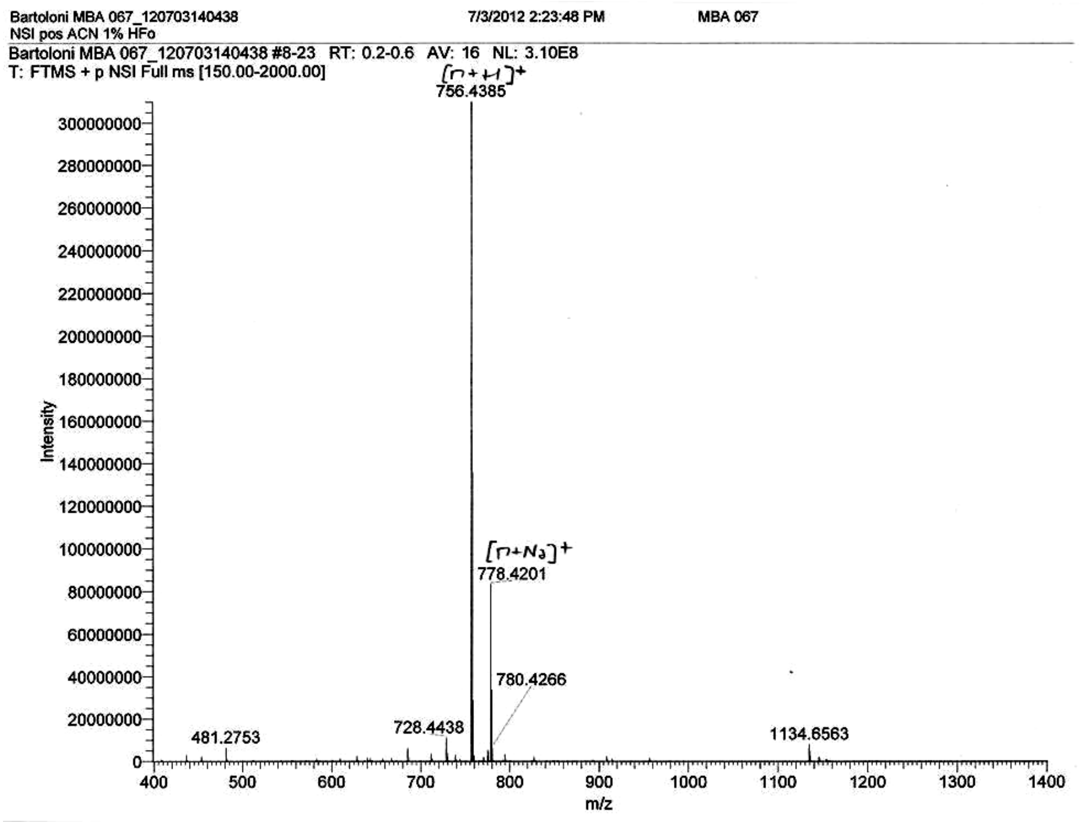

K^{1}G*k*^{2}eP*E*^{1}C^{2} (26).

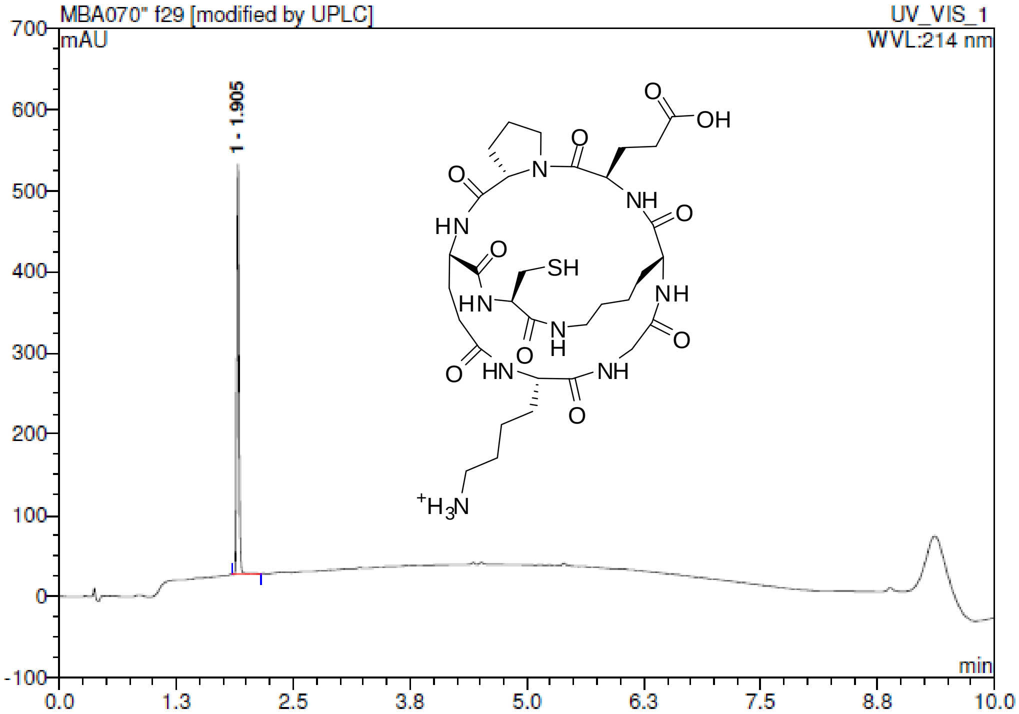

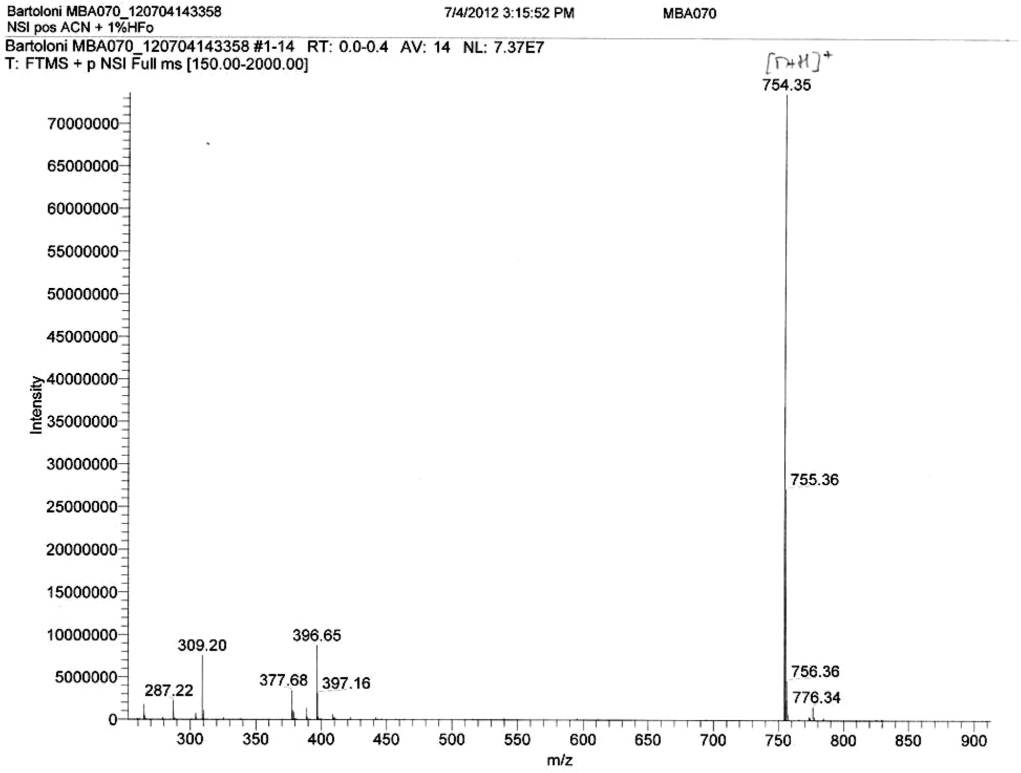

K^{2}*E*^{1}Kwz^{2}WK^{1}GBGGK$_{(biot)}$ (27x).

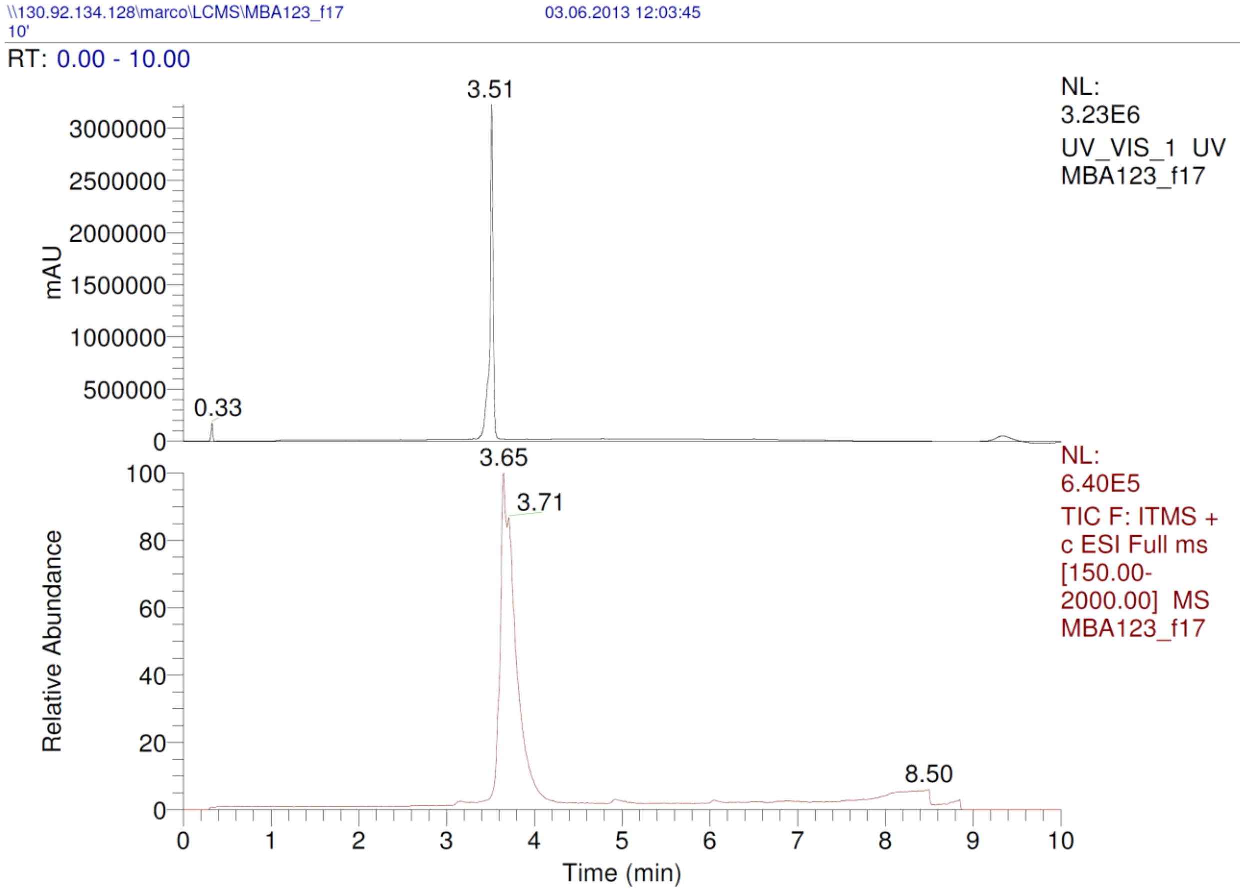

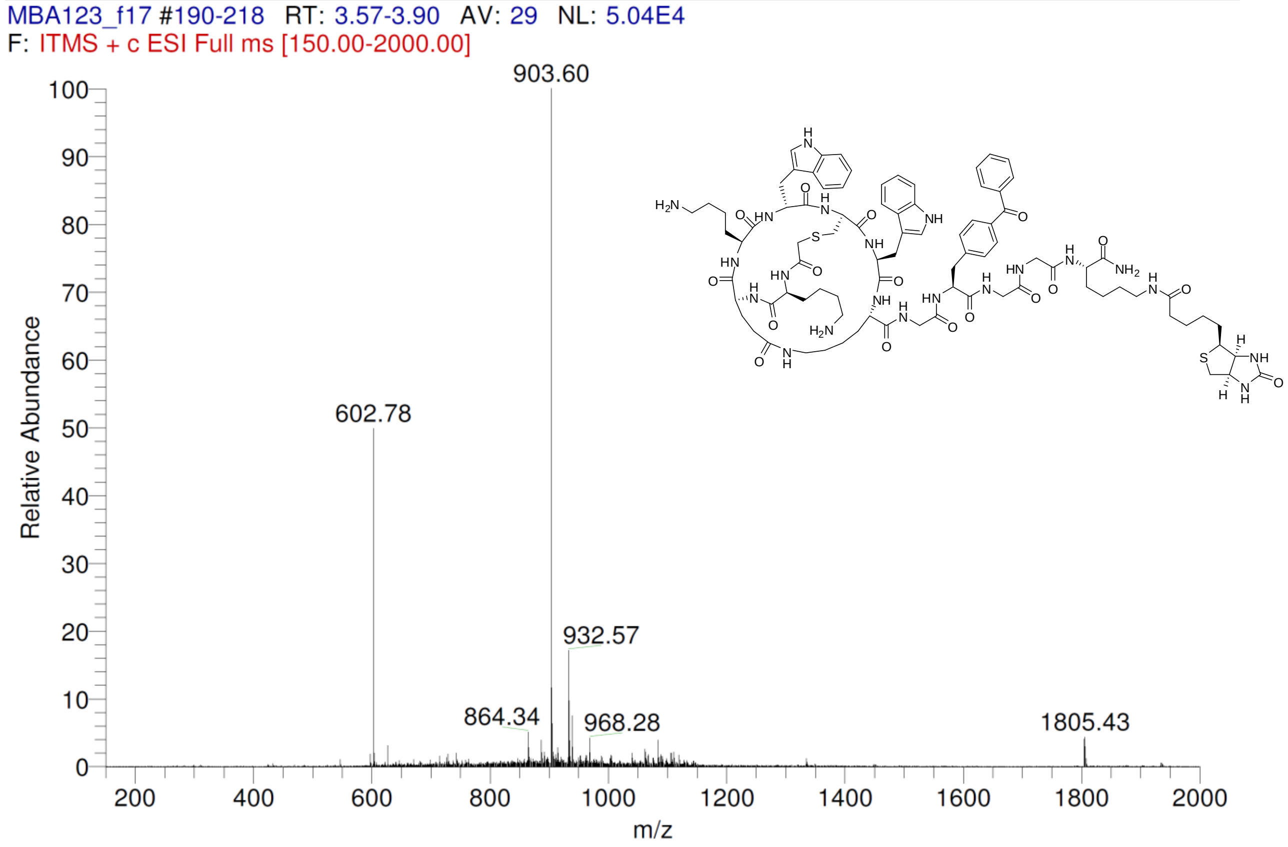

$K^{2}\mathit{E}^{1}Kwz^{2}WK^{1}GBGGK_{(biot)}$ (27s).

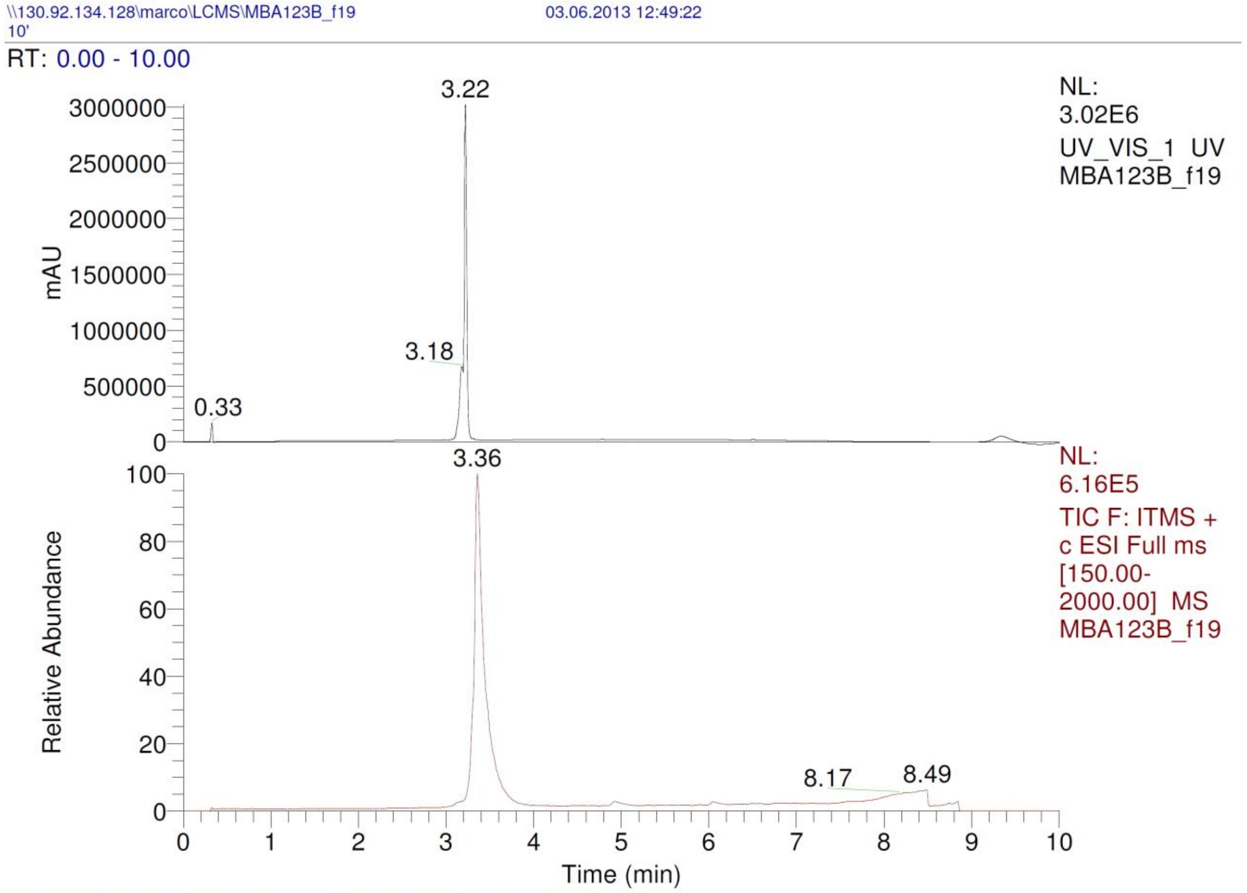

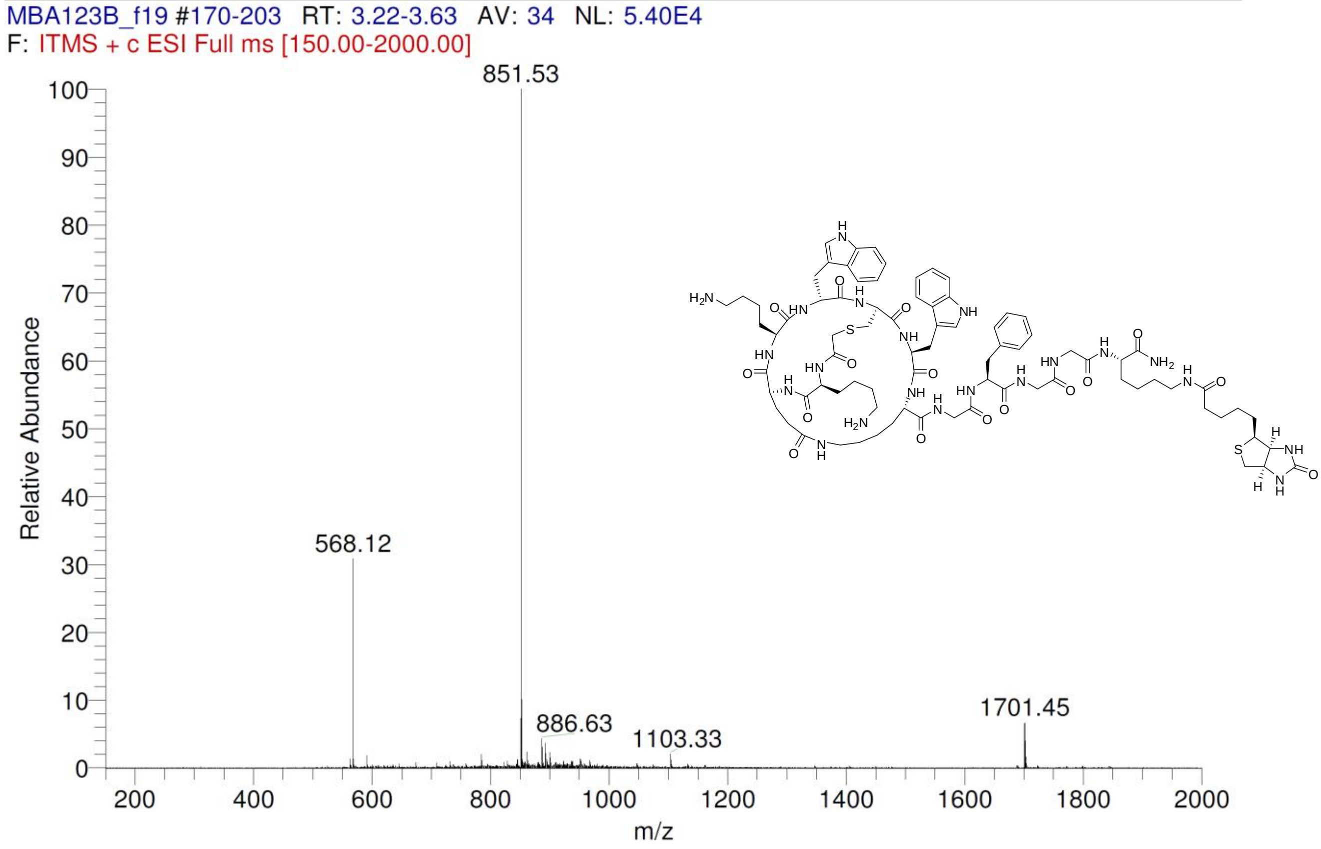

K^{2}*E*^{1}Kwz^{2}WK^{1}G (27c).

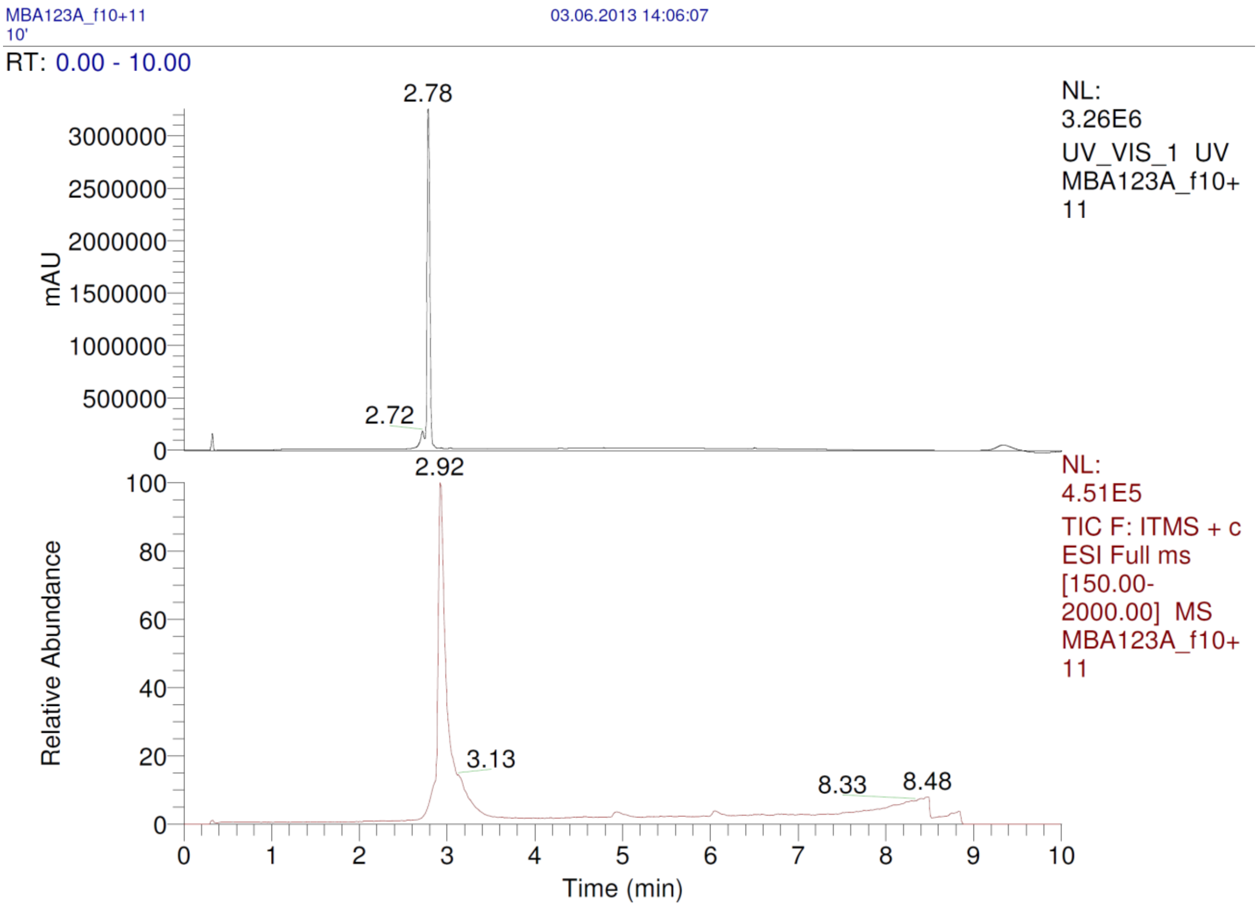

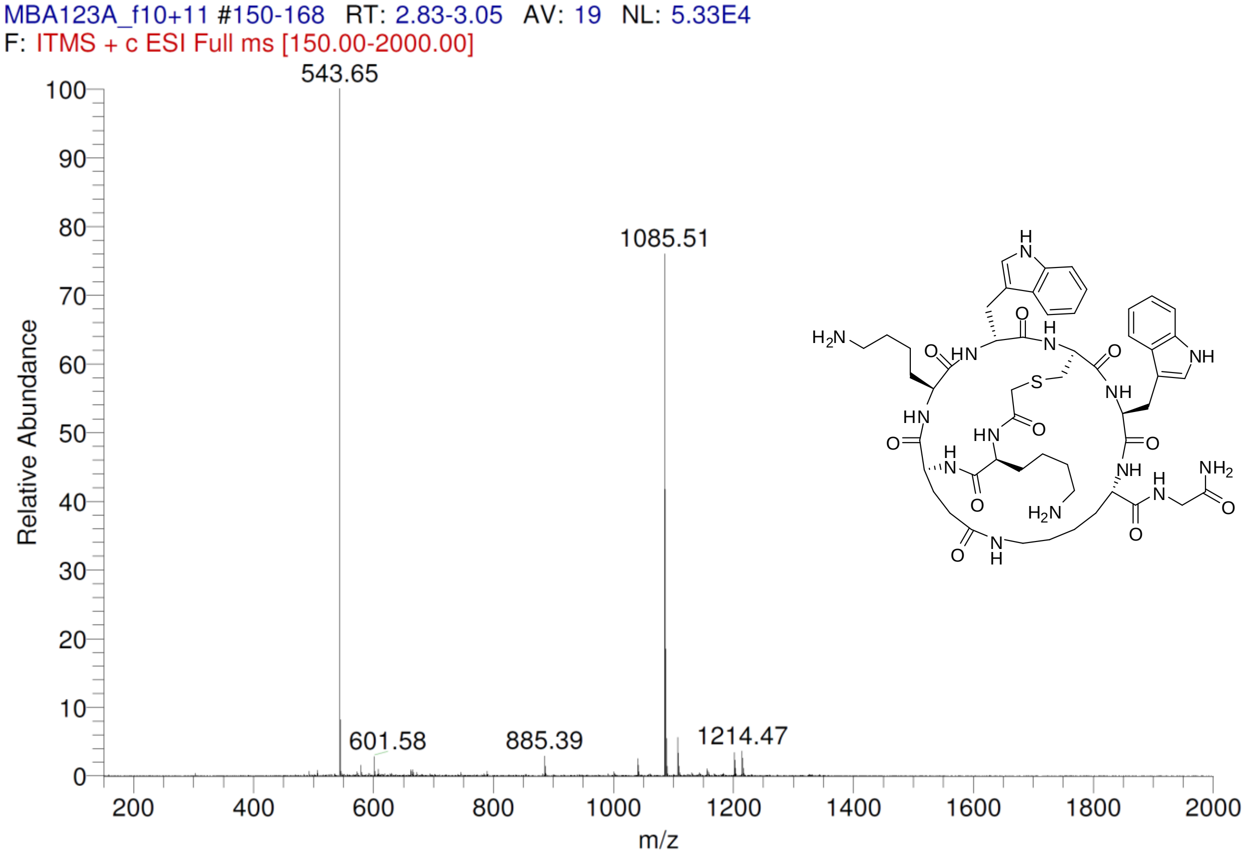

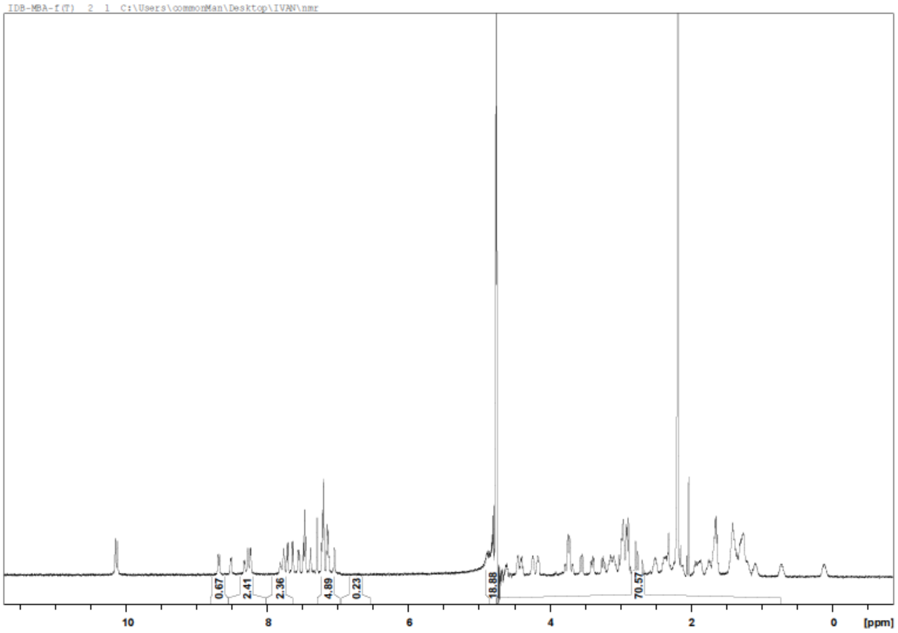

^{1}H-NMR (500 MHz, 90% H_2O/D_2O)

residue	***proton***	***δ(ppm)***	***J (Hz)***
Lys[1]	NH	8.27	^3J(NH–αCH) = 7.45
	αCH	4.26	
	β'CH	0.90	
	β''CH	0.50	
	γ'CH	1.48	
	γ''CH	1.12	
	δ'CH_3	1.61	
	δ''CH_3	1.60	
	ε'CH	2.90	
	ε''CH	2.82	
	εNH	7.70	
Glu[2]	NH	8.29	^3J(NH–αCH) = 7.57
	αCH	4.18	
	β'CH	1.21	
	β''CH	1.23	
	γ'CH	1.91	
	γ''CH	1.83	
Lys[3]	αNH	7.65	^3J(NH–αCH) = 7.38
	αCH	4.45	
	β'CH	1.59	
	β''CH	1.57	
	γ'CH	1.12	
	γ''CH	1.14	
	δ'CH	2.98	
	δ''CH	2.96	
	ε'CH	3.15	
	ε''CH	3.12	
	εNH	7.12	
Trp[4]	NH	8.49	
	αCH	4.23	
	β'CH	3.16	
	β''CH	3.12	
	δCH	7.73	
	ε'CH	10.19	
cys[5]	ε''CH	7.65	^{3}J(NH–αCH) = 7.98
	z'CH	7.31	
	z''CH	7.21	
	h'CH	7.07	
	NH	8.62	
	αCH	5.05	
	β'CH	3.88	
	β''CH	3.71	
	δ'CH	3.18	
Trp[6]	NH	7.68	
	αCH	4.68	
	β'CH	2.90	
	β''CH	2.85	
	δCH	6.98	
	ε'CH	10.19	
	ε''CH	7.65	
	z'CH	7.31	
	z''CH	7.21	
	h'CH	7.07	
Lys[7]	NH	8.32	^3J(NH–αCH) = 7.07
	αCH	4.15	
	β'CH	2.39	
	β''CH	2.37	
	γ'CH	1.96	
	γ''CH	1.98	
	δ'CH	2.15	
	δ''CH	2.22	
	ε'CH	3.22	
	ε''CH	3.12	
	εNH	7.76	
Gly[8]	NH	7.80	
	α'CH	3.98	
	α''CH	3.89	

R^{2}*E*^{1}**Rf***z*^{2}**F**K^{1}G**BGGK**$_{\mathbf{(biot)}}$ **(28x)**

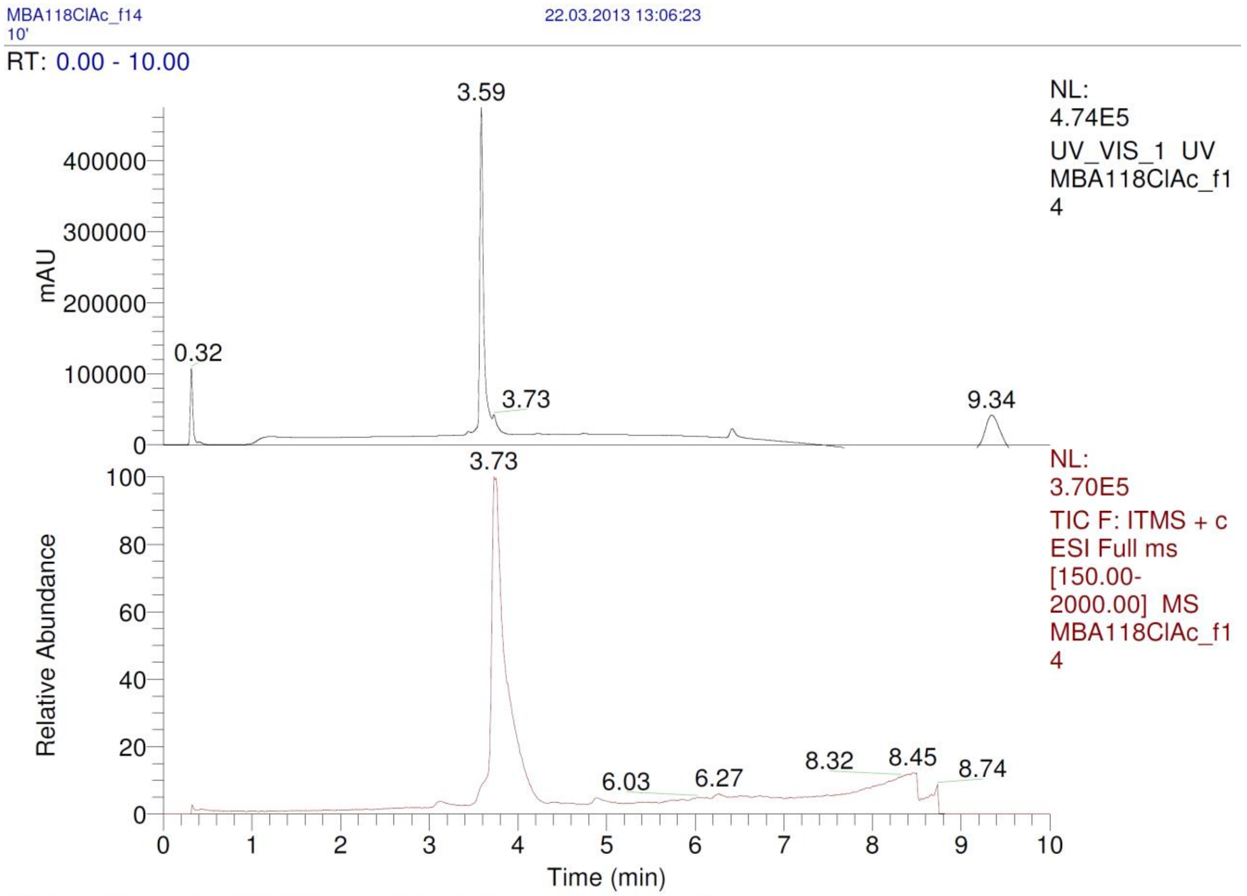

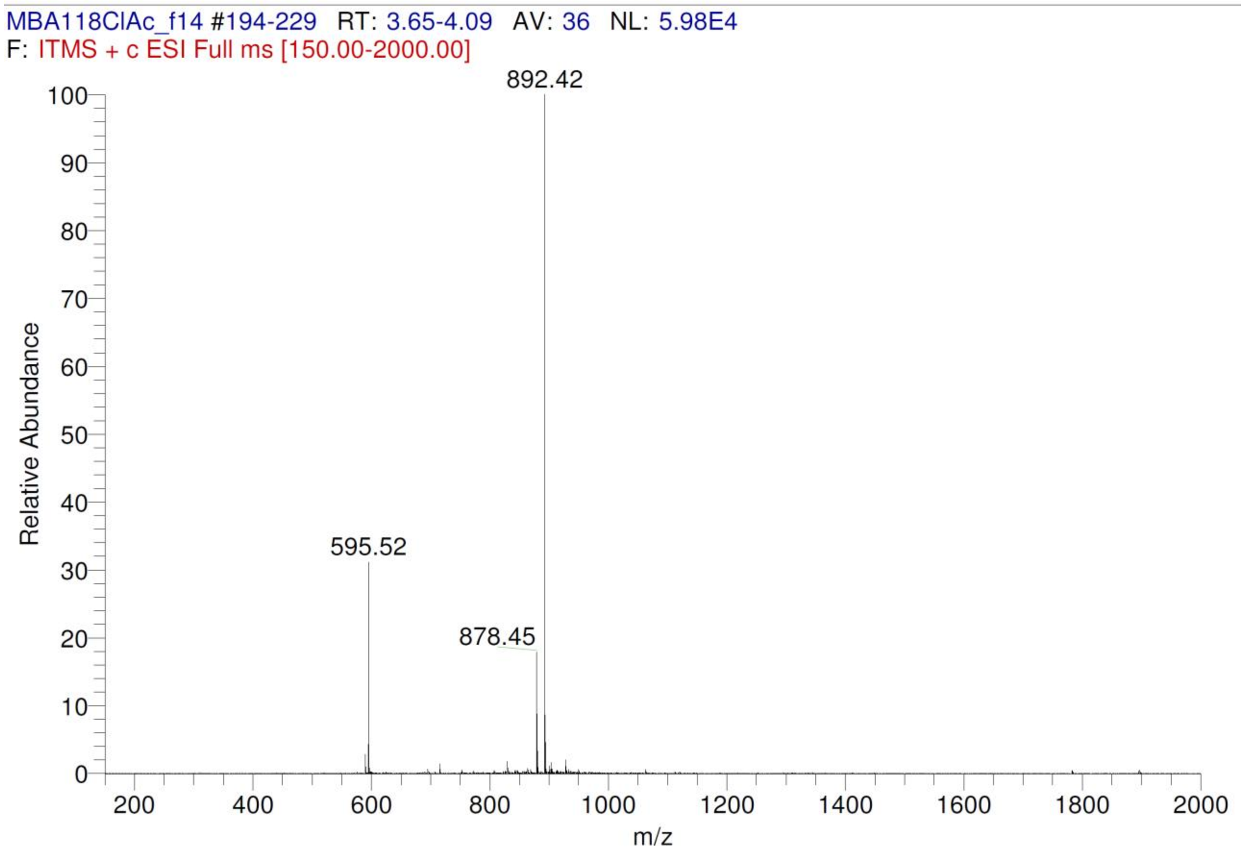

R^{2}*E*^{1}**Rf**z^{2}**F**K^{1}G**FGGK**$_{(biot)}$ **(28s)**

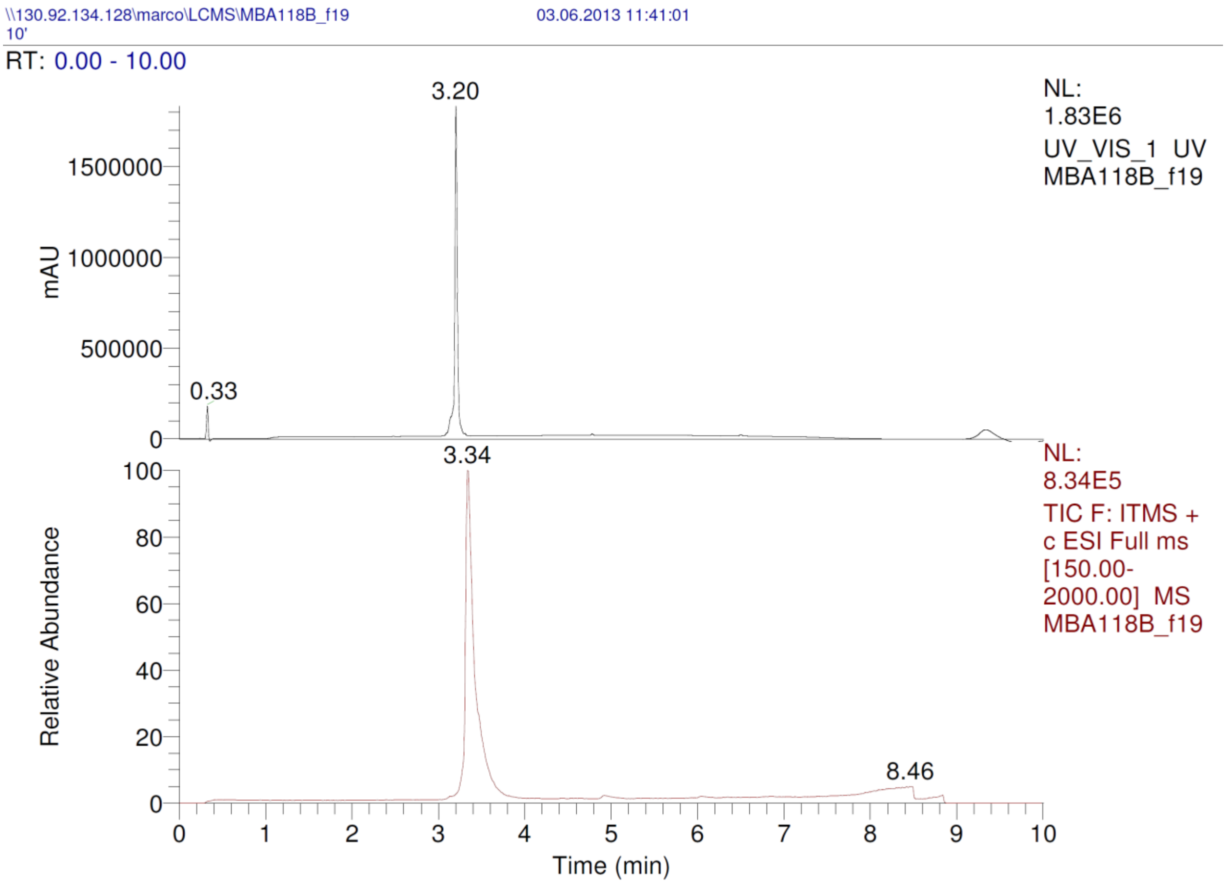

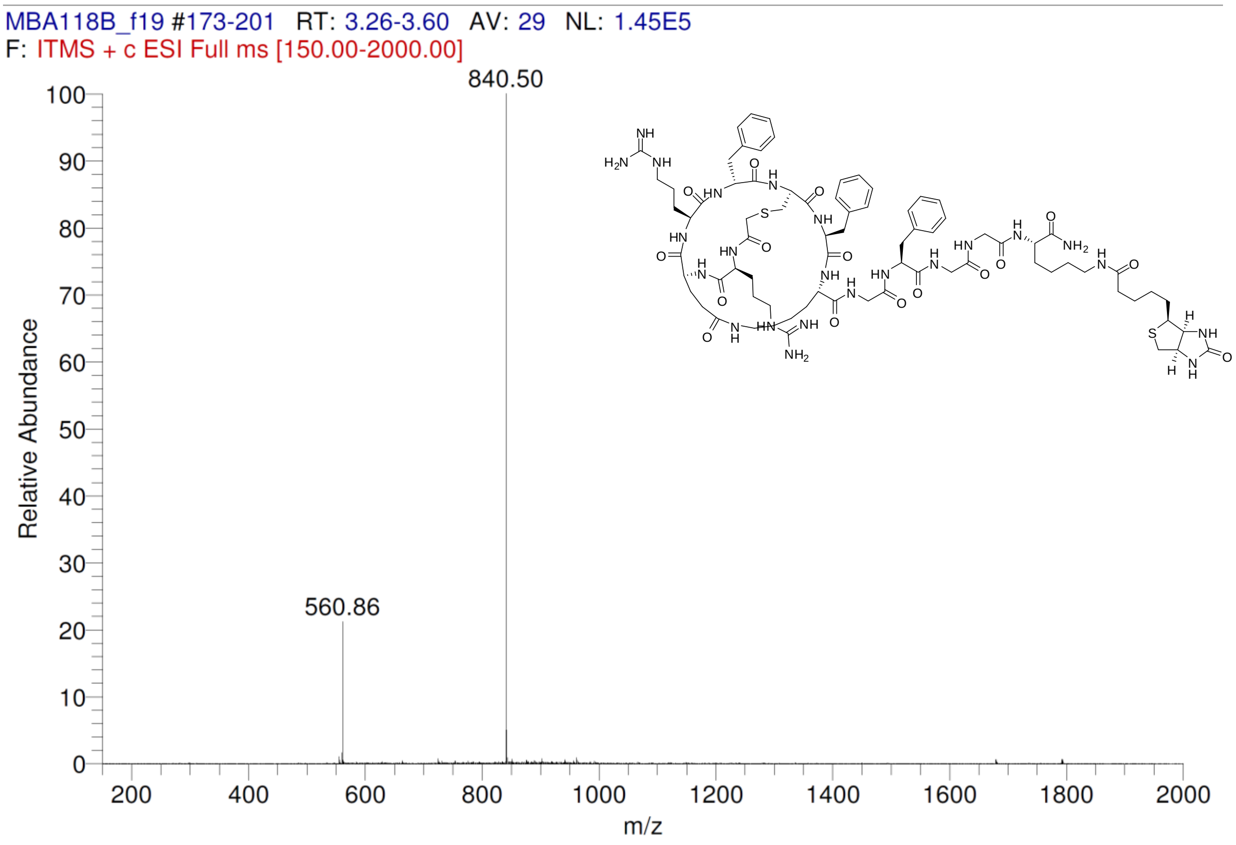

R^{2}*E*^{1}Rfz^{2}FK^{1}G (28c)

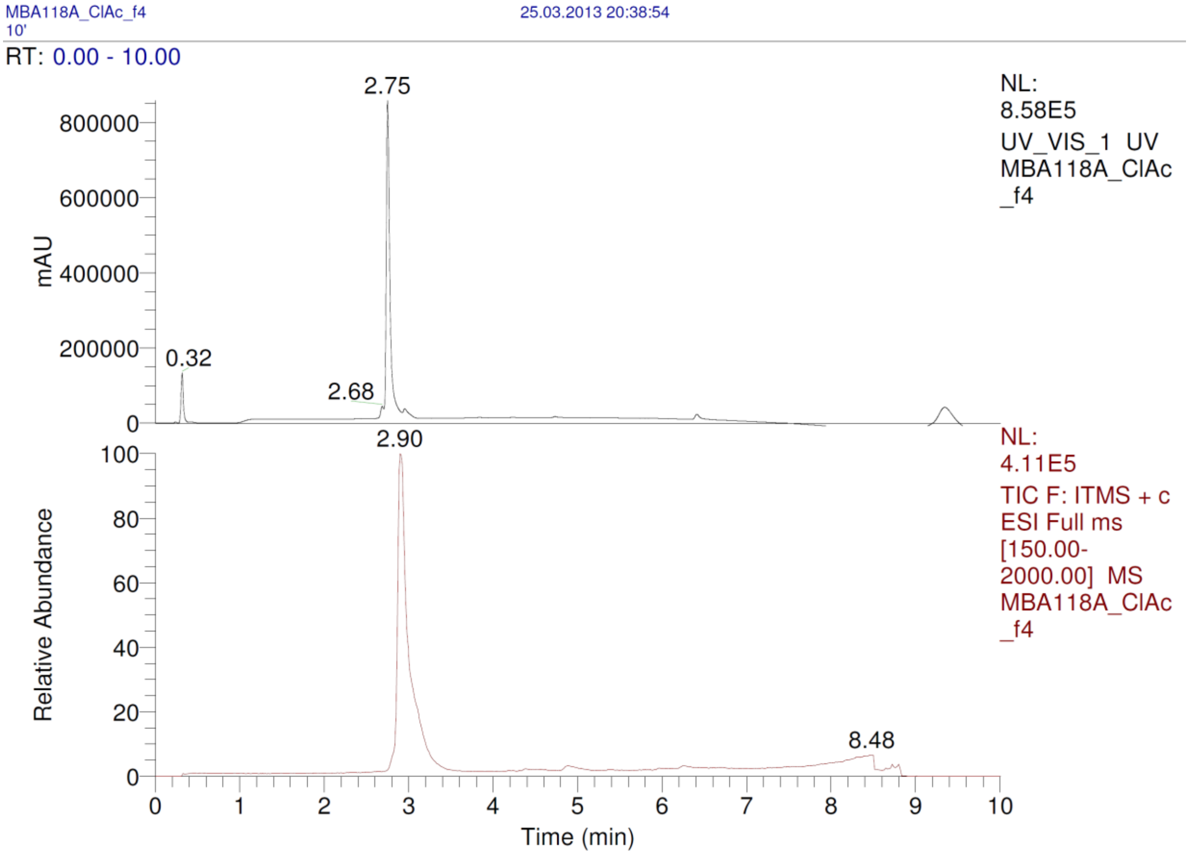

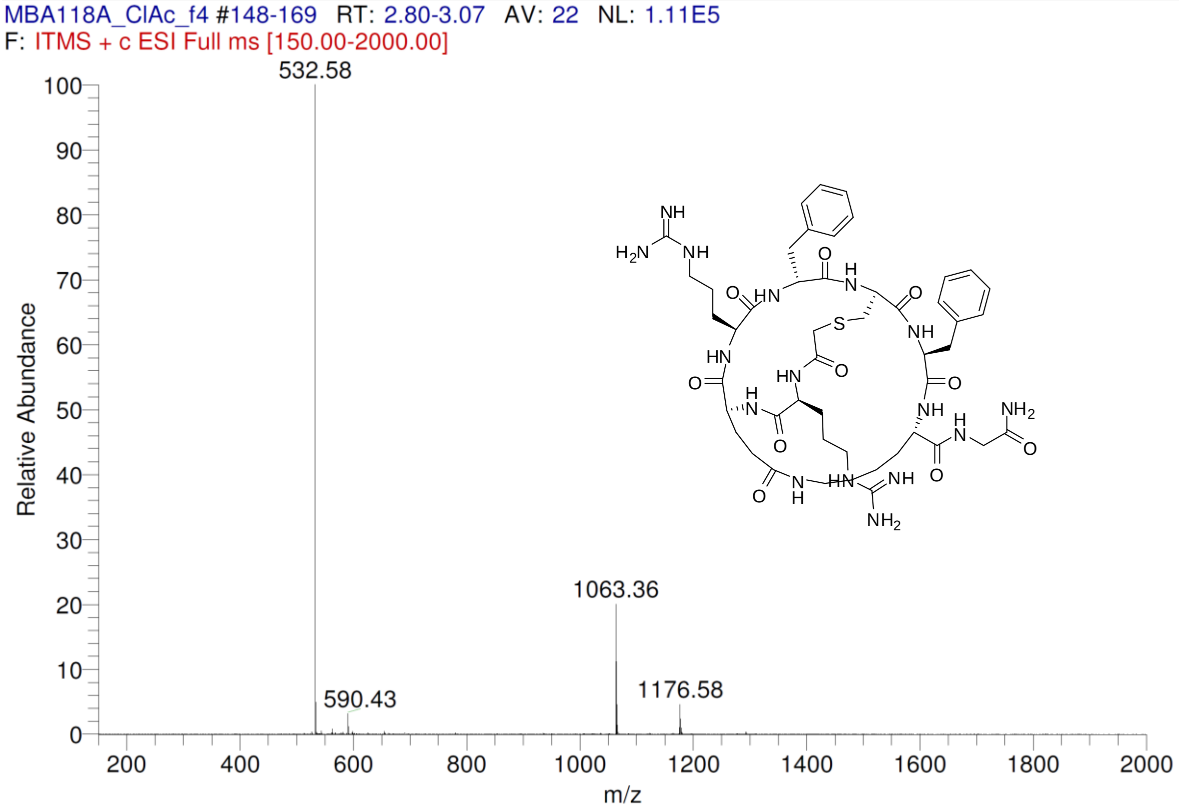

E^{2}*E*^{1}Efz^{2}FK^{1}GBGGK$_{(biot)}$ (29x)

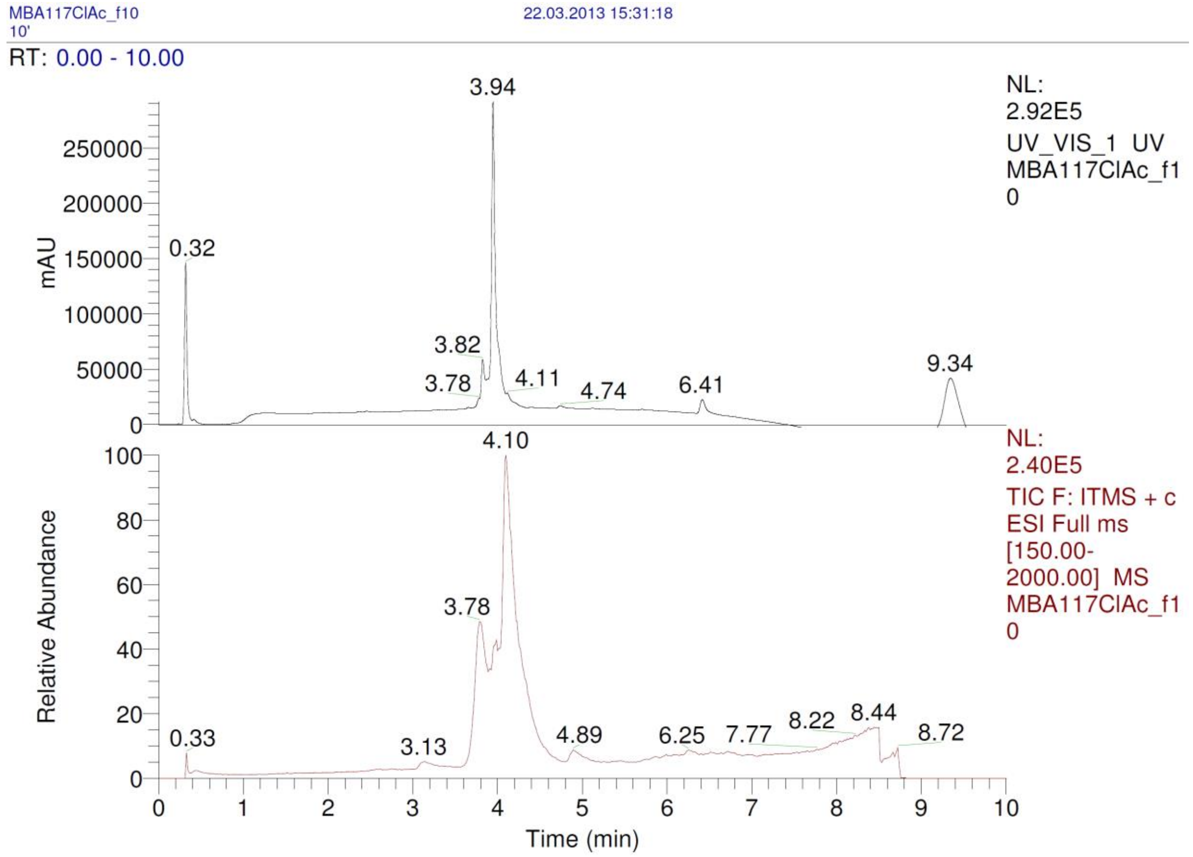

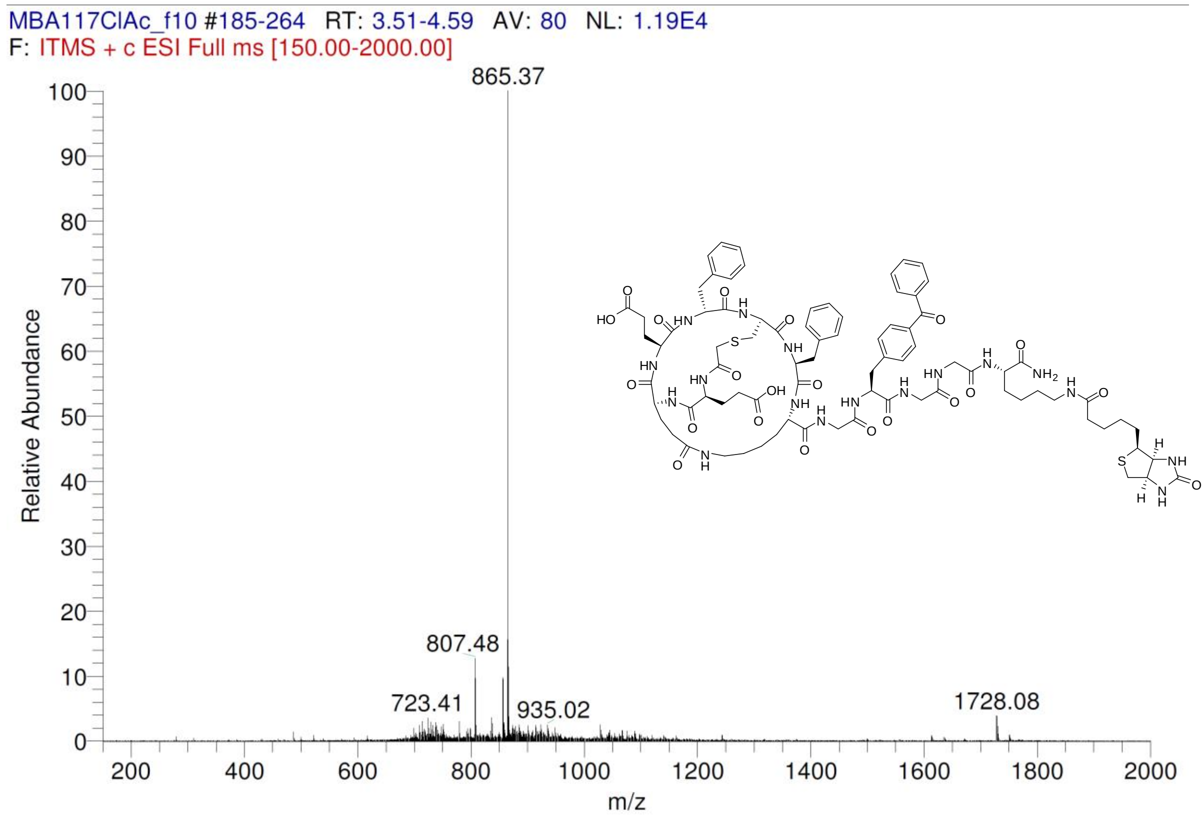

E^{2}*E*^{1}**Ef***z*^{2}FK^{1}G**FGGK**$_{(biot)}$ **(29s)**

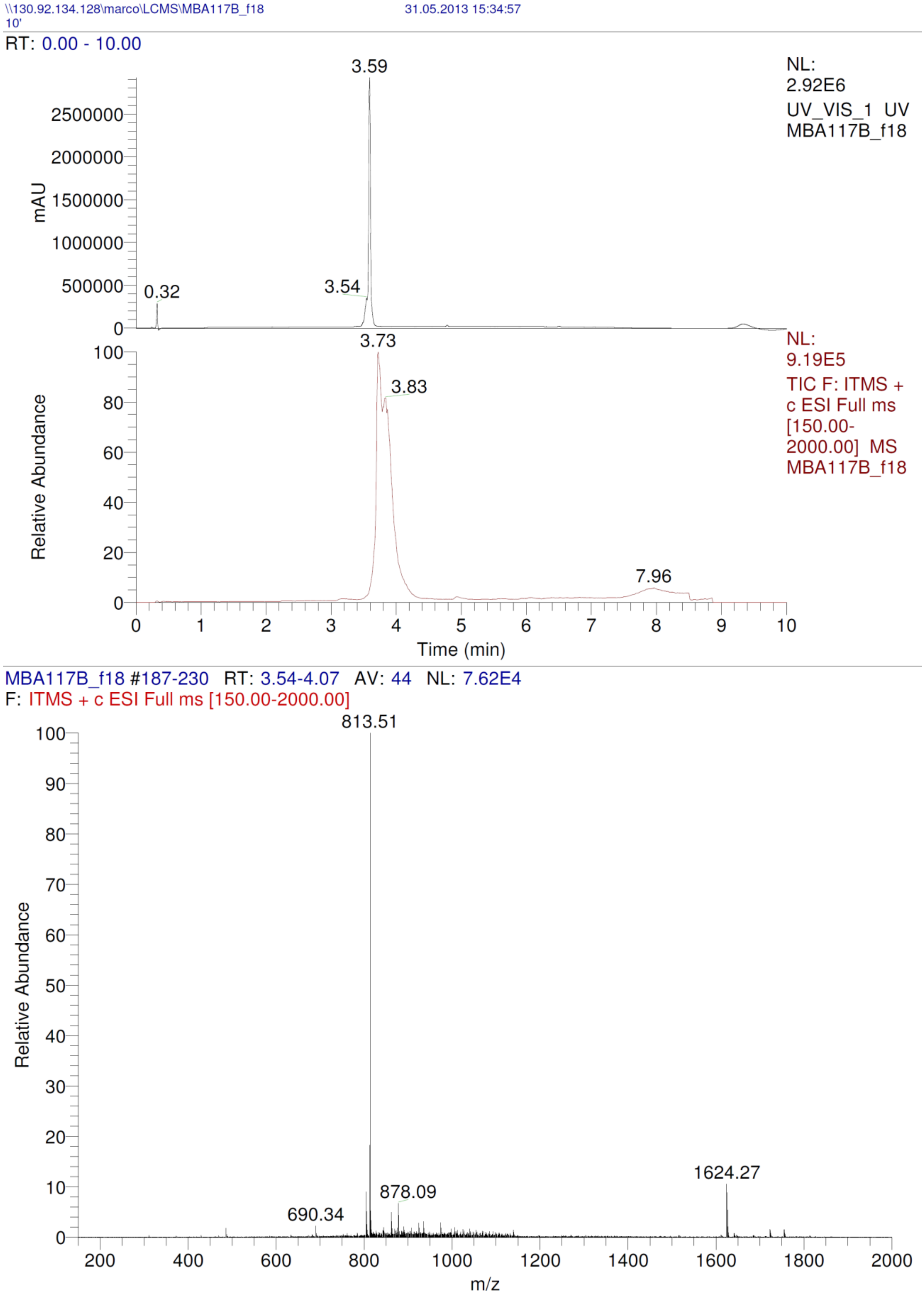

E^{2}*E*^{1}Efz^{2}FK^{1}G (29c)

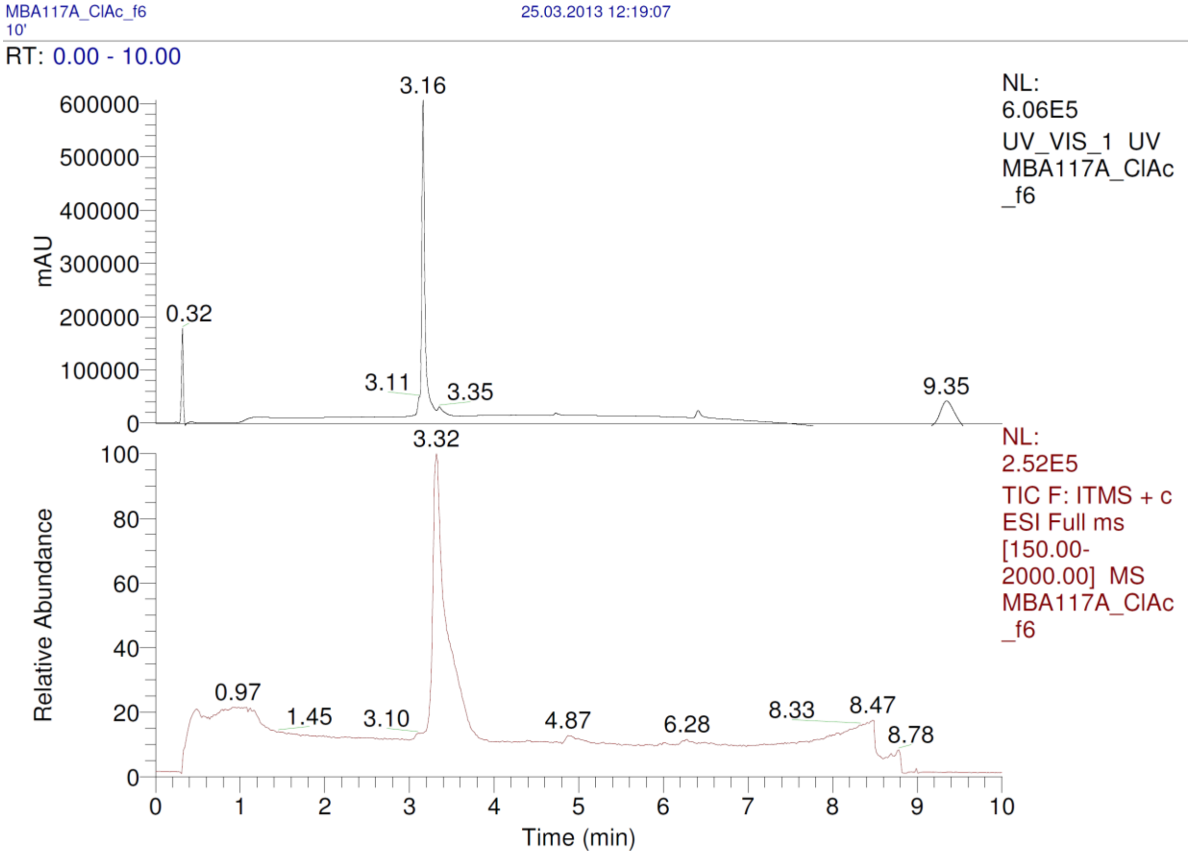

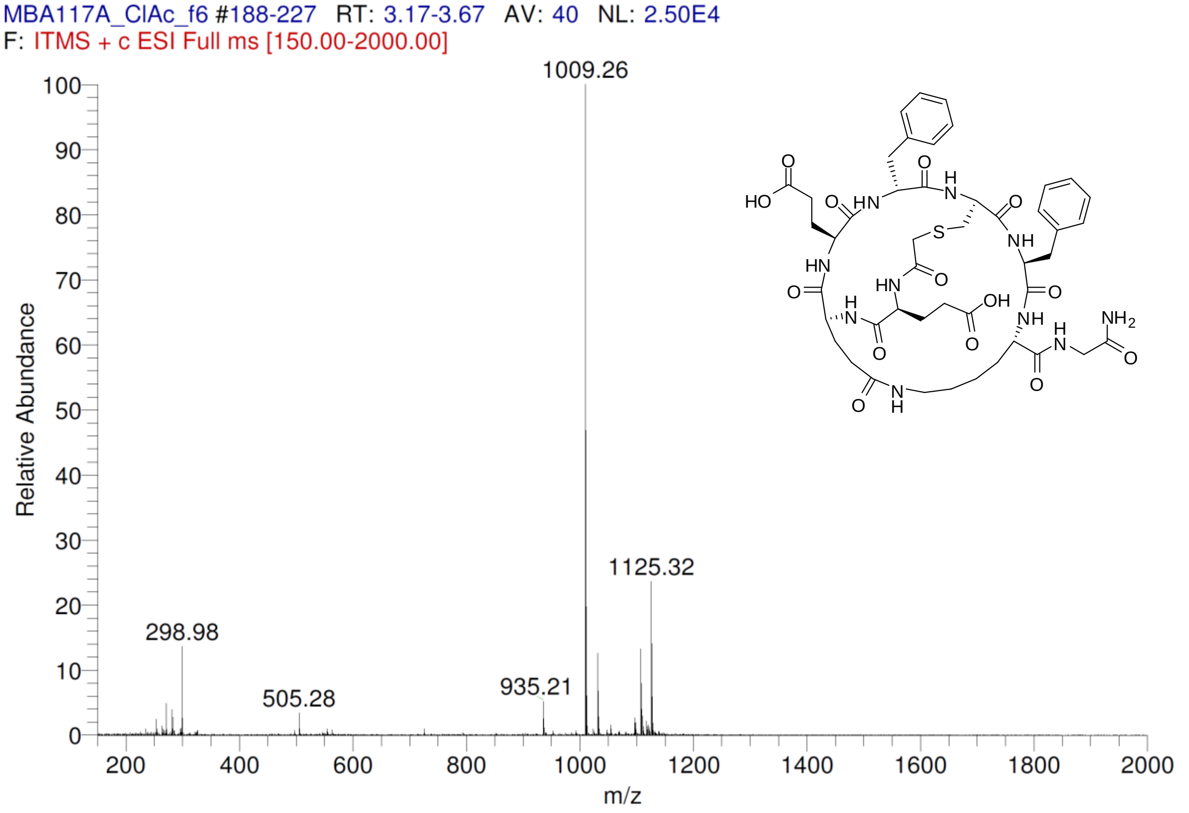

$_{Me}$l^{1}G*k*^{2}fP*E*^{1}a^{2} (30).

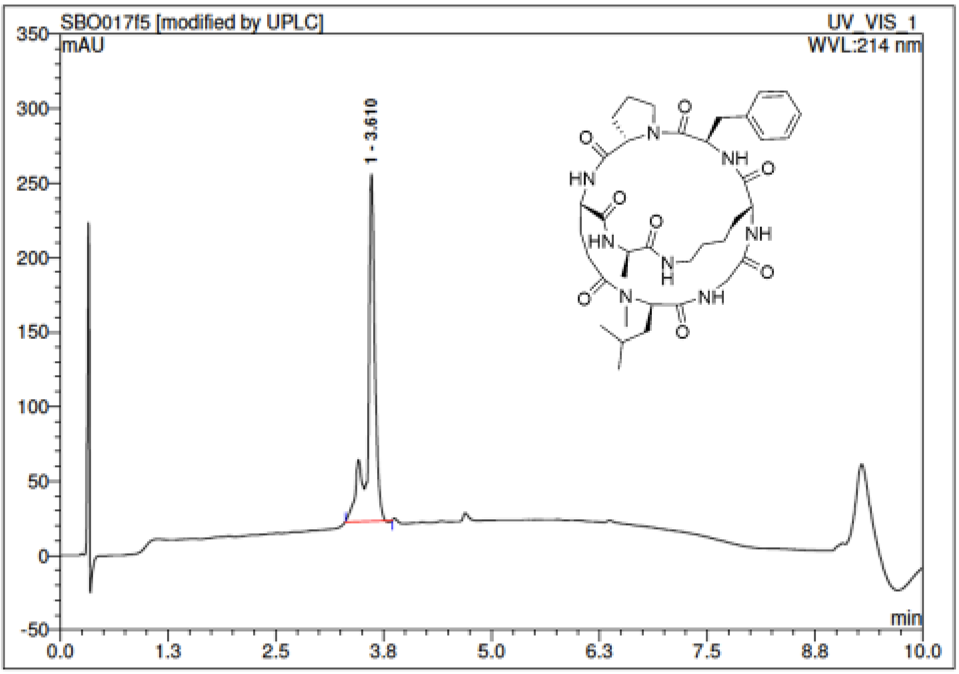

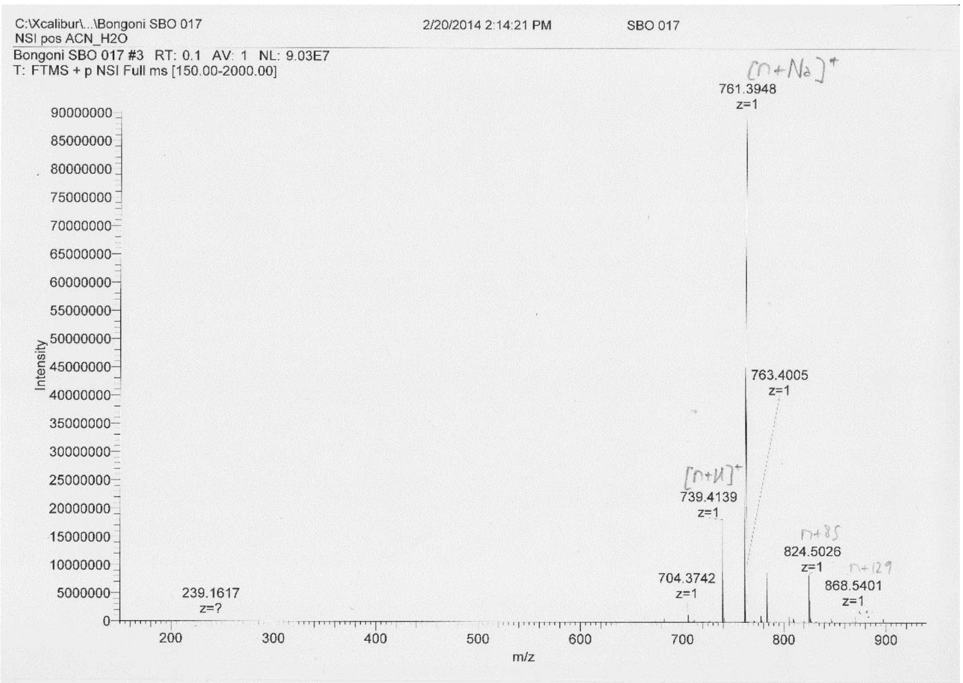

l$^{1}_{Me}$G*k*^{2}fP*E*^{1}a^{2}(31).

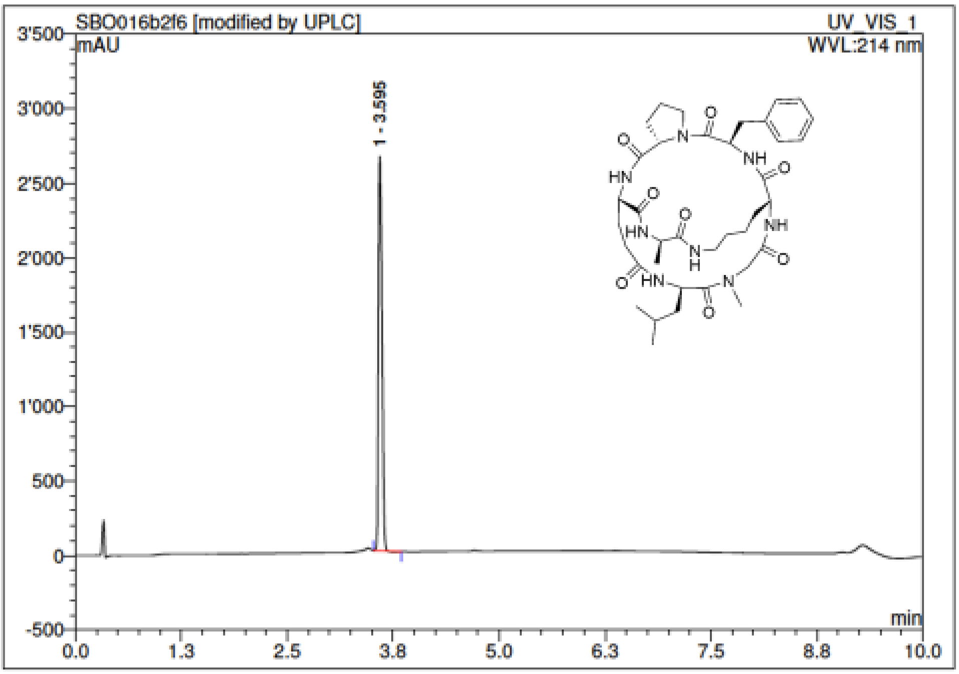

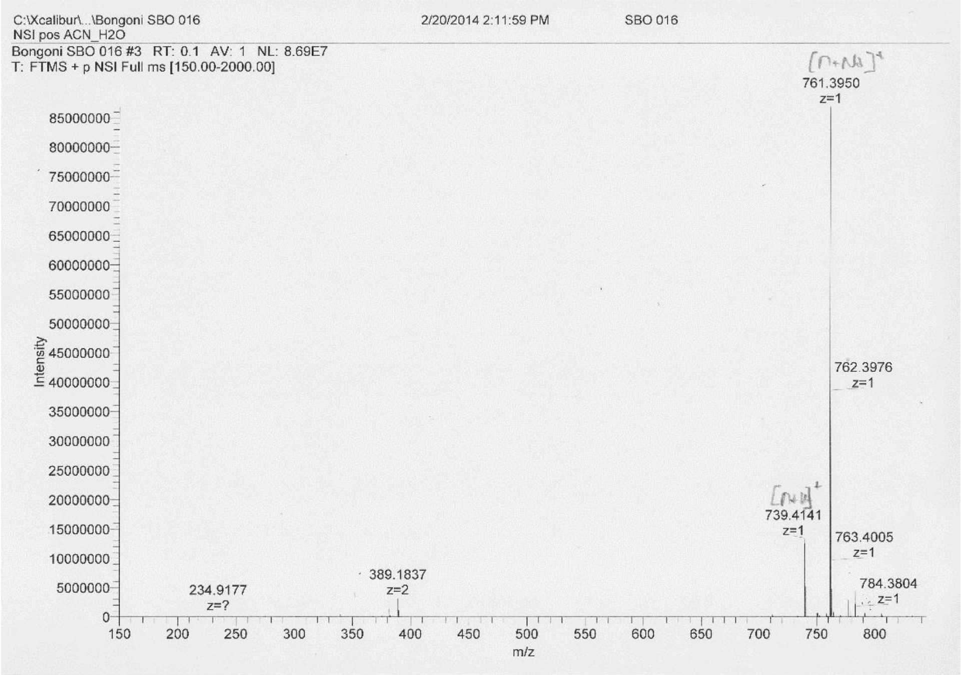

l[1]G*k*$^{2}_{Me}$fP*E*[1]a[2] (32).

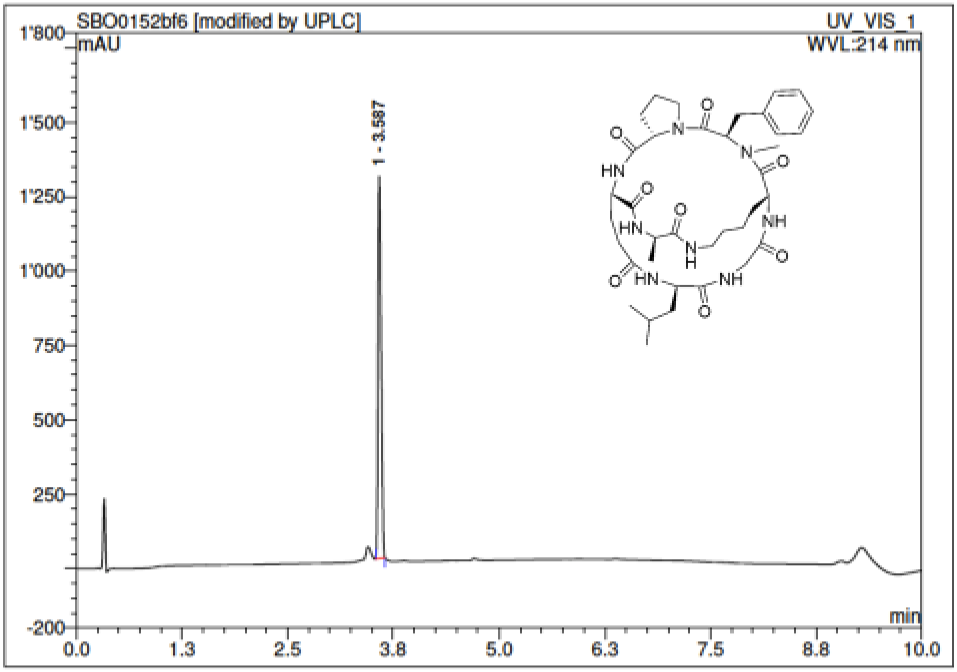

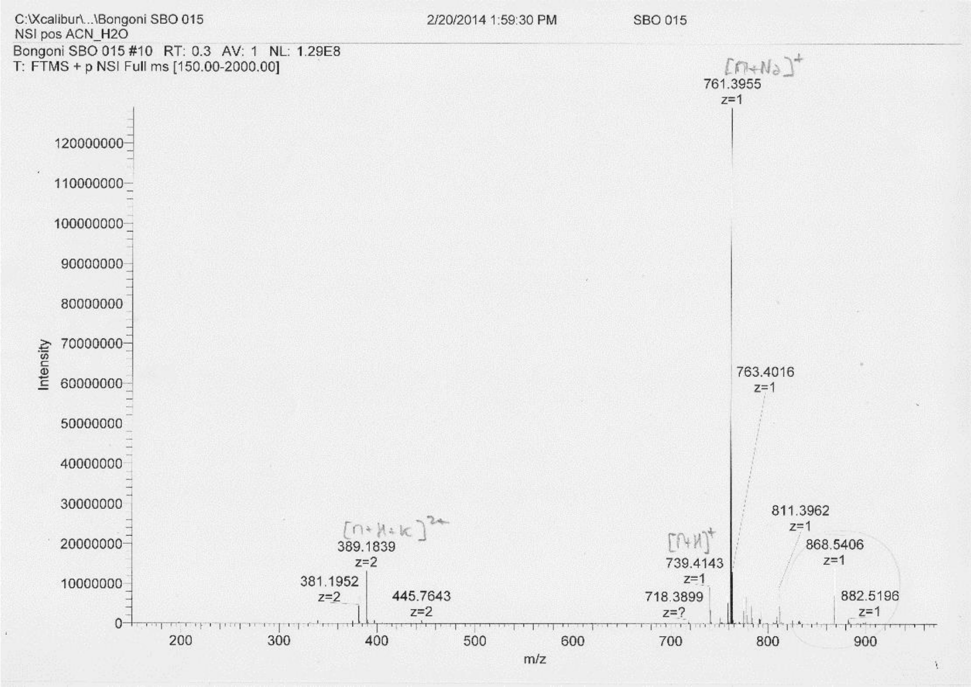

$_{Me}$l^{1}$_{Me}$G*k*^{2}fP*E*^{1}a^{2} (33).

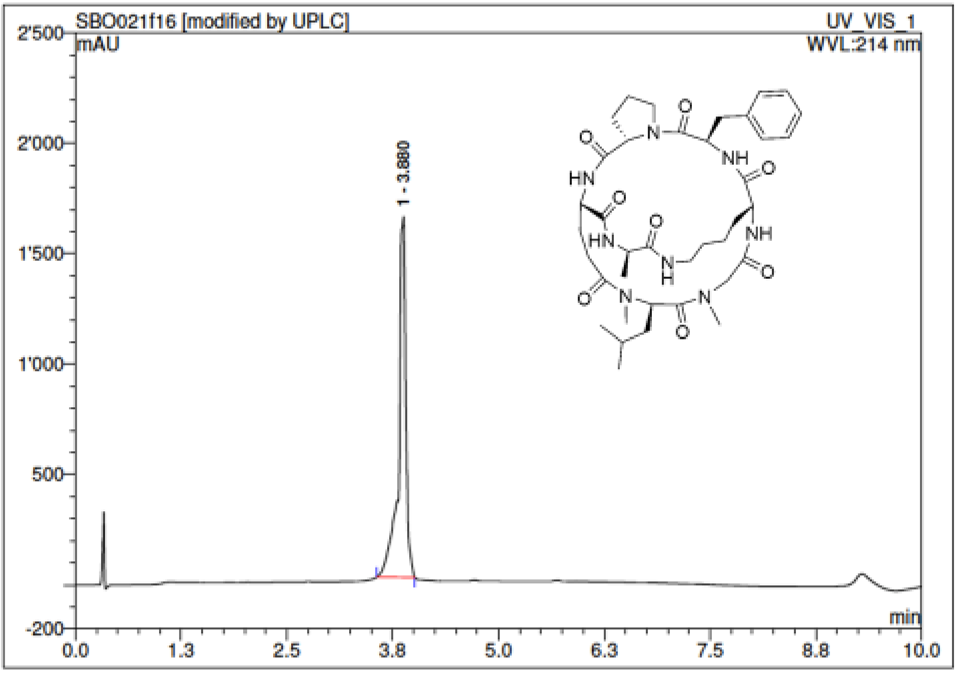

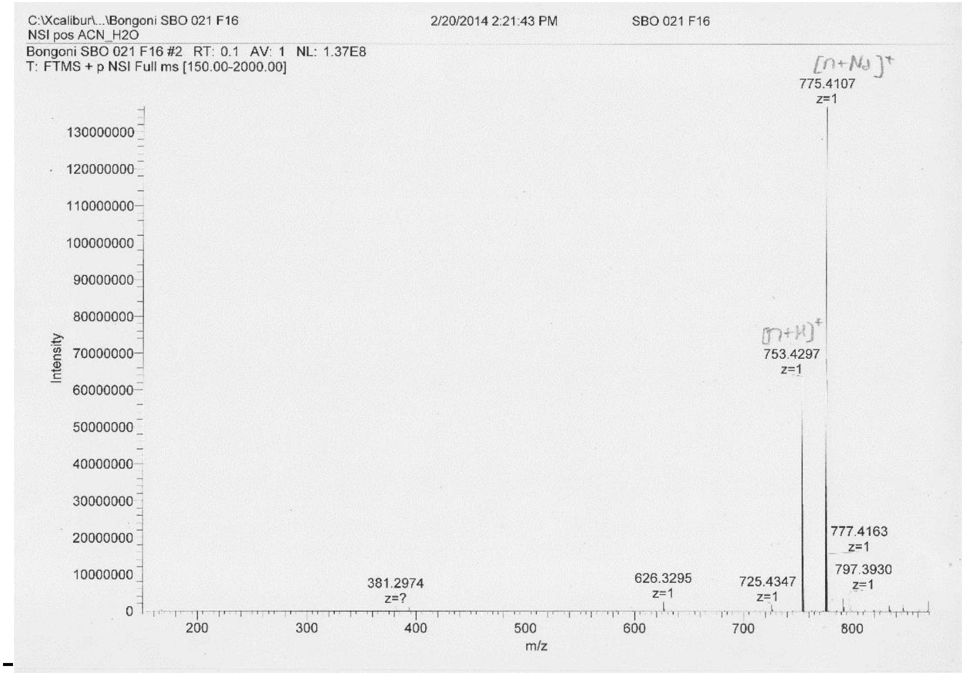

$_{Me}$l^{1}G*k*^{2}$_{Me}$fP*E*^{1}a^{2} (34).

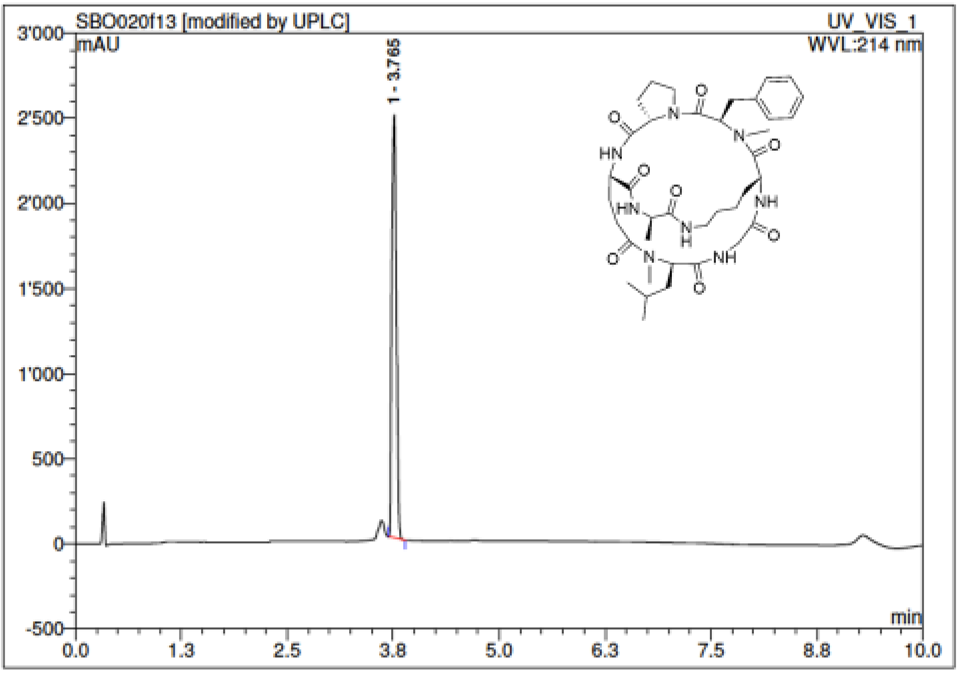

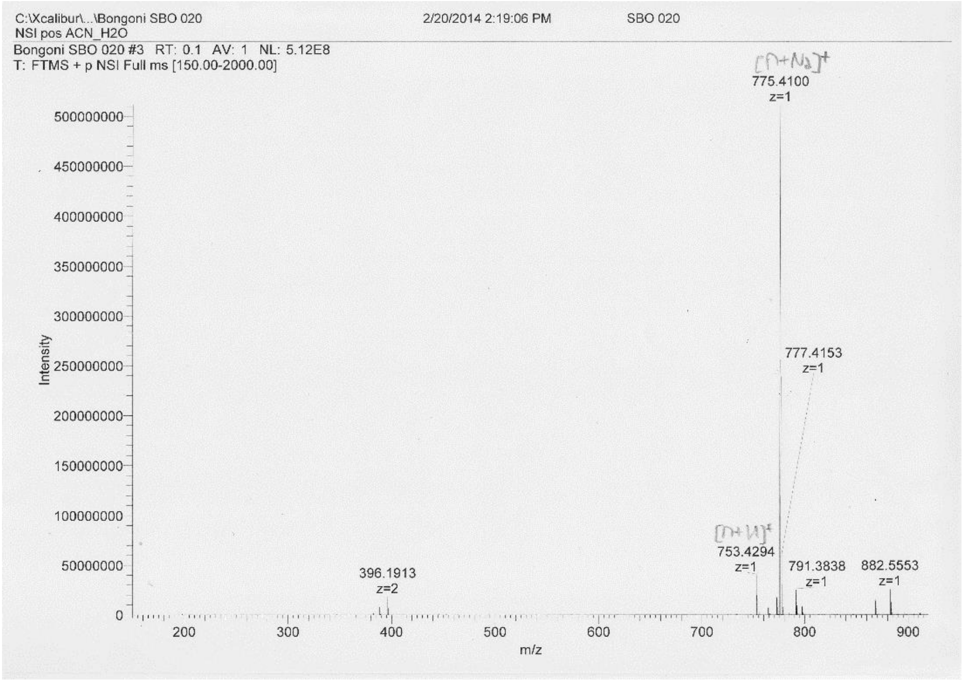

l[1]$_{Me}$G*k*[2]$_{Me}$fP*E*[1]a[2] (35).

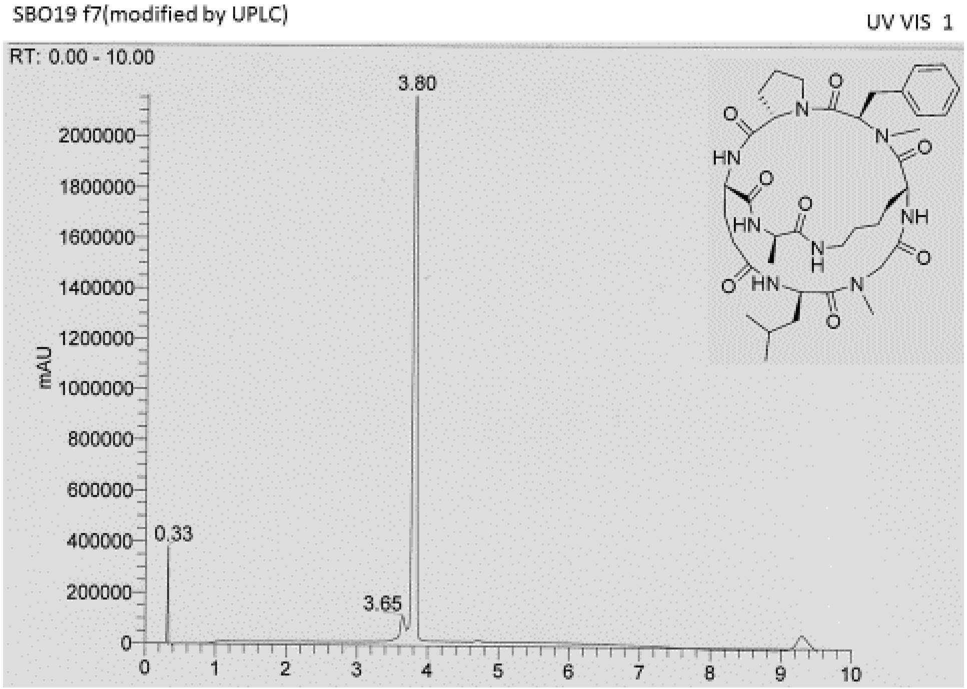

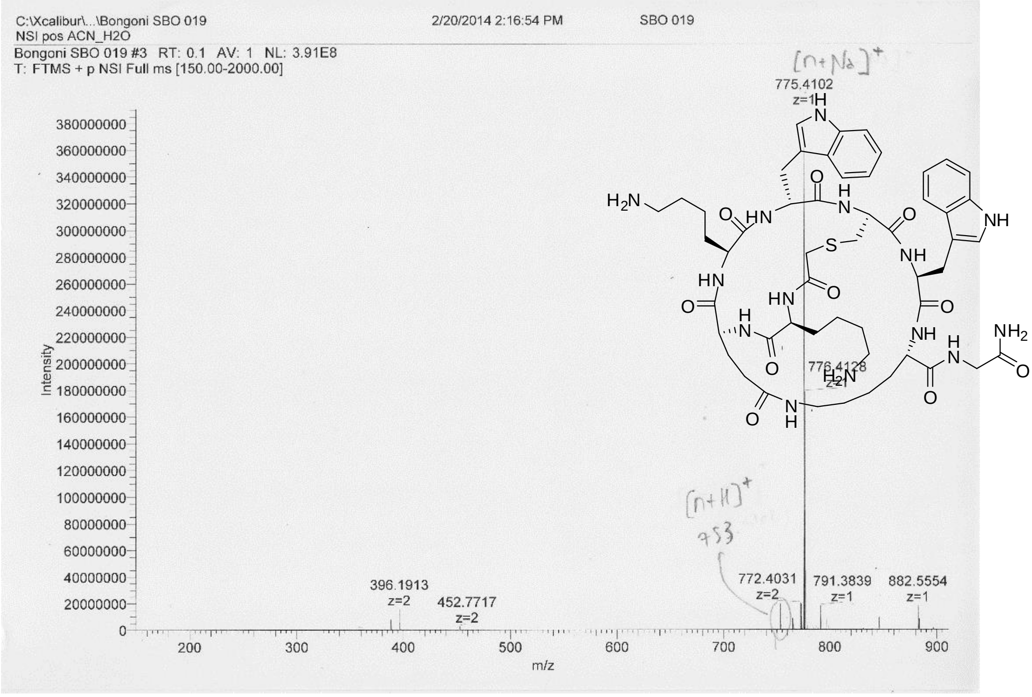

Supplement: Supplementary file 1 [file SC-006-C5SC01699A-s001.pdf]
